# Detection methods for stochastic gravitational-wave backgrounds: a unified treatment

**DOI:** 10.1007/s41114-017-0004-1

**Published:** 2017-04-04

**Authors:** Joseph D. Romano, Neil. J. Cornish

**Affiliations:** 1 Department of Physics and Astronomy, University of Texas Rio Grande Valley, Brownsville, TX 78520 USA; 2 Department of Physics, Montana State University, Bozeman, MT 59717 USA

**Keywords:** Gravitational waves, Data analysis, Stochastic backgrounds

## Abstract

We review detection methods that are currently in use or have been proposed to search for a stochastic background of gravitational radiation. We consider both Bayesian and frequentist searches using ground-based and space-based laser interferometers, spacecraft Doppler tracking, and pulsar timing arrays; and we allow for anisotropy, non-Gaussianity, and non-standard polarization states. Our focus is on relevant data analysis issues, and not on the particular astrophysical or early Universe sources that might give rise to such backgrounds. We provide a unified treatment of these searches at the level of detector response functions, detection sensitivity curves, and, more generally, at the level of the likelihood function, since the choice of signal and noise models and prior probability distributions are actually what define the search. Pedagogical examples are given whenever possible to compare and contrast different approaches. We have tried to make the article as self-contained and comprehensive as possible, targeting graduate students and new researchers looking to enter this field.

**Electronic supplementary material:**

The online version of this article (doi:10.1007/s41114-017-0004-1) contains supplementary material, which is available to authorized users.

## Introduction

The real voyage of discovery consists not in seeking new landscapes, but in having new eyes. *Marcel Proust*

It is an exciting time for the field of gravitational-wave astronomy. The observation, on September 14th, 2015, of gravitational waves from the inspiral and merger of a pair of black holes (Abbott et al. 2016e) has opened a radically new way of observing the Universe. The event, denoted GW150914, was observed simultaneously by the two detectors of the Laser Interferometer Gravitational-wave Observatory (LIGO) (Aasi et al. 2015). [LIGO consists of two 4 km-long laser interferometers, one located in Hanford, Washington, the other in Livingston, LA.] The merger event that produced the gravitational waves occured in a distant galaxy roughly 1.3 billion light years from Earth. The initial masses of the two black holes were estimated to be $36^{+5}_{-4}\ \mathrm{M}_\odot$ and $29^{+4}_{-4}\ \mathrm{M}_\odot$, and that of the post-merger black hole as $62^{+4}_{-4}\ \mathrm{M}_\odot$ (Abbott et al. 2016f). The difference between the initial and final masses corresponds to $3.0^{+0.5}_{-0.5}\ \mathrm{M}_\odot c^2$ of energy radiated in gravitational waves, with a peak luminosity of *more than ten times the combined luminosity of all the stars in all the galaxies in the visible universe*! The fact that this event was observed *only* in gravitational waves—and not in electromagnetic waves—illustrates the complementarity and potential for new discoveries that comes with the opening of the gravitational-wave window onto the universe.

GW150914 is just the first of many gravitational-wave signals that we expect to observe over the next several years. Indeed, roughly 3 months after the detection of GW150914, a second event, GW151226, was observed by the two LIGO detectors (Abbott et al. 2016d). This event also involved the inspiral and merger of a pair of stellar mass black holes, with initial component masses $14.2^{+8.3}_{-3.7}\ \mathrm{M}_\odot$ and $7.5^{+2.3}_{-2.3}\ \mathrm{M}_\odot$, and a final black hole mass of $20.8^{+6.1}_{-1.7}\ \mathrm{M}_\odot$. The source was at a distance of roughly 1.4 billion light-years from Earth, comparable to that of GW150914. Advanced LIGO will continue interleaving observation runs and commissioning activities to reach design sensivity around 2020 (Aasi et al. 2015), which will allow detections of signals like GW150914 and GW151226 with more than three times the signal-to-noise ratio than was observed for GW150914 (which was 24). In addition, the Advanced Virgo detector (Acernese et al. 2015) (a 3 km-long laser interferometer in Cascina, Italy) and KAGRA (Aso et al. 2013) (a 3 km-long cryogenic laser interferometer in Kamioka mine in Japan) should both be taking data by the end of 2016. There are also plans for a third LIGO detector in India (Iyer et al. 2011). A global network of detectors such as this will allow for much improved position reconstruction and parameter estimation of the sources (Abbott et al. 2016i).

### Motivation and context

GW150914 and GW151226 were single events—binary black hole mergers that were observed with both template-based searches for compact binary inspirals and searches for generic gravitational-wave transients in the two LIGO detectors (Abbott et al. 2016e, d). The network matched-filter signal-to-noise ratio (Owen and Sathyaprakash 1999) for these two events, using relativitistic waveform models for binary black holes, was 24 and 13, respectively. The probability that these detections were due to noise alone is ${<} 2\times 10^{-7}$, corresponding to a significance greater than $5\sigma$—the standard for so-called “gold-plated” detections. But for every loud event like GW150914 or GW151226, we expect many more quiet events that are too distant to be individually detected, since the associated signal-to-noise ratios are too low.

The total rate of merger events from the population of stellar-mass binary black holes of which GW150914 and GW151226 are members can be estimated^1^ by multiplying the local rate estimate of 9–240 $\mathrm{Gpc}^{-3}\, \mathrm{year}^{-1}$ (Abbott et al. 2016g) by the comoving volume out to some large redshift, e.g., $z\sim 6$. This yields a total rate of binary black hole mergers between ${\sim }1$ per minute and a few per hour. Since the duration of each merger signal in the sensitive band of a LIGO-like detector is of order a few tenths of a second to ${\sim } 1$ s, the *duty cycle* (the fraction of time that the signal is “on” in the data) is ${\ll } 1$. This means that the combined signal from such a population of binary black holes will be “popcorn-like”, with the majority of the individual signals being too weak to individually detect. Since the arrival times of the merger signals are randomly-distributed, the combined signal from the population of binary black holes is itself random—it is an example of a *stochastic background* of gravitational radiation.

More generally, a stochastic background of gravitational radiation is *any* random gravitational-wave signal produced by a large number of weak, independent, and unresolved sources. The background doesn’t have to be popcorn-like, like the expected signal from the population of binary black holes which gave rise to GW150914 and GW151226. It can be composed of individual deterministic signals that overlap in time (or in frequency) producing a “confusion” noise analogous to conversations at a cocktail party. Such a confusion noise is produced by the galactic population of compact white dwarf binaries. (For this case, the stochastic signal is so strong that it becomes a *foreground*, acting as an additional source of noise when trying to detect *other* weak gravitational-wave signals in the same frequency band). Alternatively, the signal can be *intrinsically* random, associated with stochastic processes in the early Universe or with unmodeled sources, like supernovae, which produce signals that are not described by deterministic waveforms.

The focus of this review article is on data analysis strategies (i.e., detection methods) that can be used to detect and ultimately characterize a stochastic gravitational-wave background. To introduce this topic and to set the stage for the more detailed discussions to follow in later sections, we ask (and start to answer) the following questions:

#### Why do we care about detecting a stochastic background?

Detecting a stochastic background of gravitational radiation can provide information about astrophysical source populations and processes in the very early Universe, which are inaccessible by any other means. For example, electromagnetic radiation cannot provide a picture of the Universe any earlier than the time of last of scattering (roughly 400,000 years after the Big Bang). Gravitational waves, on the other hand, can give us information all the way back to the onset of inflation, a mere ${\sim } 10^{-32}~\mathrm{s}$ after the Big Bang. (See Maggiore 2000 for a detailed discussion of both cosmological and astrophysical sources of a stochastic gravitational-wave background).

#### Why is detection challenging?

Stochastic signals are effectively another source of noise in a single detector. So the fundamental problem is how to distinguish between gravitational-wave “noise” and instrumental noise. It turns out that there are several ways to do this, as we will discuss in the later sections of this article.

#### What detection methods can one use?

Cross-correlation methods can be used whenever one has multiple detectors that respond to the common gravitational-wave background. For single detector analyses e.g., for the Laser Space Interferometer Antenna (LISA), one needs to take advantage of null combinations of the data (which act as instrument noise monitors) or use instrument noise modeling to try to distinguish the gravitational-wave signal from instrumental noise. Over the past 15 years or so, the number of detection methods for stochastic backgrounds has increased considerably. So now, in addition to the standard cross-correlation search for a “vanilla” (Gaussian-stationary, unpolarized, isotropic) background, one can search for non-Gaussian backgrounds, anisotropic backgrounds, circularly-polarized backgrounds, and backgrounds with polarization components predicted by alternative (non-general-relativity) theories of gravity. These searches are discussed in Sects. 7 and 8.

Table 1 summarizes the basic properties of various analysis methods that have been used (or proposed) for stochastic background searches. Despite apparent differences, *all* analyses use a likelihood function, e.g., for defining frequentist statistics or for calculating posterior distributions for Bayesian inference (as will be described in more detail in Sect. 3), and take advantage of cross-correlations if multiple detectors are available (as will be described in more detail in Sect. 4).

**Table 1:** Overview of analysis methods for stochastic gravitational-wave backgrounds

Early analyses (before 2000)	More recent analyses
Used frequentist statistics	Use both frequentist and Bayesian inference
Used cross-correlation methods	Use cross-correlation methods and stochastic templates; use null channels or knowledge about instrumental noise when cross-correlation is not available
Assumed Gaussian noise	Have allowed non-Gaussian noise
Assumed stationary, Gaussian, unpolarized, and isotropic gravitational-wave backgrounds	Have allowed non-Gaussian, polarized, and anisotropic gravitational-wave backgrounds
Were done primarily in the context of ground-based detectors (e.g., resonant bars and LIGO-like interferometers) where the small-antenna (i.e., long-wavelength) approximation was valid	Have been done in the context of space-based detectors (e.g., spacecraft tracking, LISA) and pulsar timing arrays for which the small-antenna approximation is not valid

The number and flexibility of the methods have increased considerably since the year 2000

#### What are the prospects for detection?

The prospects for detection depend on the source of the background (i.e., astrophysical or cosmological) and the type of detector being used. For example, a space-based interferometer like LISA is *guaranteed* to detect the gravitational-wave confusion noise produced by the galactic population of compact white dwarf binaries. Pulsar timing arrays, on the other hand, should be able to detect the confusion noise from supermassive black hole binaries (SMBHBs) at the centers of merging galaxies, provided the binaries are not affected by their environments in a way that severely diminishes the strength of the background (Shannon et al. 2015). Detection sensitivity curves are a very convenient way of comparing theoretical predictions of source strengths to the sensivity levels of the various detectors (as we will discuss in Sect. 10).

### Searches across the gravitational-wave spectrum

**Fig. 1:**
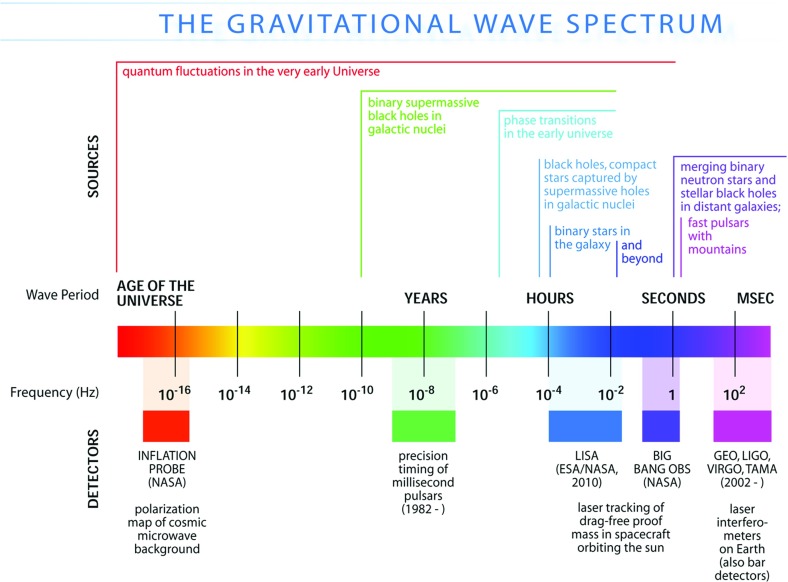
Gravitational-wave spectrum, together with potential sources and relevant detectors. *Image credit* Institute of Gravitational Research/University of Glasgow

The frequency band of ground-based laser interferometers like LIGO, Virgo, and KAGRA is between ${\sim }10~\mathrm{Hz}$ and a few kHz (gravity gradient and seismic noise are the limiting^2^ noise sources below 10 Hz, and photon shot noise above a couple of kHz). Outside this band there are several other experiments—both currently operating and planned—that should also be able to detect gravitational waves. An illustration of the gravitational-wave spectrum, together with potential sources and relevant detectors, is shown in Fig. 1. We highlight a few of these experiments below.

#### Cosmic microwave background experiments

At the extreme low-frequency end of the spectrum, corresponding to gravitational-wave periods of order the age of the Universe, the Planck satellite (ESA 2016c) and other cosmic microwave background (CMB) experiments, e.g., BICEP and Keck (BICEP/Keck 2016) are looking for evidence of relic gravitational waves from the Big Bang in the *B*-mode component of CMB polarization maps (Kamionkowski et al. 1997; Hu and White 1997; Ade et al. 2015a). In 2014, BICEP2 announced the detection of relic gravitational waves (Ade et al. 2014), but it was later shown that the observed *B*-mode signal was due to contamination by intervening dust in the galaxy (Flauger et al. 2014; Mortonson and Seljak 2014). So at present, these experiments have been able to only *constrain* (i.e., set upper limits on) the amount of gravitational waves in the very early Universe (Ade et al. 2015a). But these constraints severely limit the possibility of detecting the relic gravitational-wave background with any of the higher-frequency detection methods, unless its spectrum increases with frequency. [Note that standard models of inflation predict a relic background whose energy density is almost constant in frequency, leading to a strain spectral density that decreases with frequency.] Needless to say, the detection of a primordial gravitational-wave background is a “holy grail” of gravitational-wave astronomy.

#### Pulsar timing arrays

At frequencies between ${\sim }10^{-9}~\mathrm{Hz}$ and $10^{-7}~\mathrm{Hz}$, corresponding to gravitational-wave periods of order decades to years, pulsar timing arrays (PTAs) can be used to search for gravitational waves. This is done by carefully monitoring the arrival times of radio pulses from an array of galactic millisecond pulsars, looking for *correlated* modulations in the arrival times induced by a passing gravitational wave (Detweiler 1979; Hellings and Downs 1983). The most-likely gravitational-wave source for PTAs is a gravitational-wave background formed from the incoherent superposition of signals produced by the inspirals and mergers of SMBHBs in the centers of distant galaxies (Jaffe and Backer 2003). These searches continue to improve their sensitivity by upgrading instrument back-ends and discovering more millisecond pulsars that can be added to the array. These improvements have led to more constraining upper limits on the amplitude of the gravitational-wave background (Shannon et al. 2015; Arzoumanian et al. 2016), with a detection being likely before the end of this decade (Siemens et al. 2013; Taylor et al. 2016b).

#### Space-based interferometers

At frequencies between ${\sim }10^{-4}~\mathrm{Hz}$ and $10^{-1}~\mathrm{Hz}$, corresponding to gravitational-wave periods of order hours to minutes, proposed space-based interferometers like LISA can search for gravitational waves from a wide variety of sources (Gair et al. 2013). These include: (i) inspirals and mergers of SMBHBs with masses of order $10^6~\mathrm{M}_\odot$, (ii) captures of compact stellar-mass objects around supermassive black holes, and (iii) the stochastic confusion noise produced by compact white-dwarf binaries in our galaxy. In fact, hundreds of binary black holes that are individually resolvable by LISA will coalesce in the aLIGO band within a 10 year period, opening up the possibility of doing *multi-band* gravitational-wave astronomy (Sesana 2016).

The basic space-based interferometer configuration consists of three satellites (each housing two lasers, two telescopes, and two test masses) that fly in an equilateral-triangle formation, with arm lengths of order several million km. A variant of the original LISA design was selected in February 2017 by the European Space Agency (ESA) as the 3rd large mission in its Cosmic Vision Program (ESA 2016a). The earliest launch date for LISA would be around 2030. A technology-demonstration mission, called LISA Pathfinder (ESA 2016b), was launched in December 2015, meeting or exceeding all of the requirements for an important subset of the LISA technologies (Armano et al. 2016).

#### Other detectors

Finally, in the frequency band between ${\sim }0.1~\mathrm {Hz}$ and $10~\mathrm {Hz}$, there are proposals for both Earth-based detectors (Harms et al. 2013) and also second-generation space-based interferometers—the Big-Bang Observer (BBO) (Phinney et al. 2004) and the DECI-hertz interferometer Gravitational-wave Observatory (DECIGO) (Ando et al. 2010). Such detectors would be sensitive to gravitational waves with periods between ${\sim }10~\mathrm {s}$ and $0.1~\mathrm {s}$. The primary sources in this band are intermediate-mass ($10^3$–$10^4~M_\odot$) binary black holes, galactic and extra-galactic neutron star binaries, and a cosmologically-generated stochastic background.

### Goal of this article

Starting with the pioneering work of Grishchuk (1976), Detweiler (1979), Hellings and Downs (1983), and Michelson (1987), detection methods for gravitational-wave backgrounds have increased in scope and sophistication over the years, with several new developments occuring rather recently. As mentioned above, we have search methods now that target different properties of the background (e.g., isotropic or anisotropic, Gaussian or non-Gaussian, polarized or unpolarized, etc.). These searches are necessarily implemented differently for different detectors, since, for example, ground-based detectors like LIGO and Virgo operate in the *small-antenna* (or *long-wavelength*) limit, while pulsar timing arrays operate in the *short-wavelength* limit. Moreover, each of these searches can be formulated in terms of either Bayesian or frequentist statistics. *The goal of this review article is to discuss these different detection methods from a perspective that attempts to *unify* the different treatments, emphasizing the similarities that exist when viewed from this broader perspective.*

### Unification

The extensive literature describing stochastic background analyses leaves the reader with the impression that highly specialized techniques are needed for ground-based, space-based, and pulsar timing observations. Moreover, reviews of gravitational-wave data analysis leave the impression that the analysis of stochastic signals is somehow fundamentally different from that of any other signal type. Both of these impressions are misleading. The apparent differences are due to differences in terminology and perspective. By adopting a common analysis framework and notation, we are able to present a *unified* treatment of gravitational-wave data analysis across source classes and observation techniques.

We will provide a unified treatment of the various methods at the level of detector response functions, detection sensitivity curves, and, more generally, at the level of the likelihood function, since the choice of signal and noise models and prior probability distributions are actually what define the search. The same photon time-of-flight calculation underpins the detector response functions, and the choice of prior for the gravitational-wave template defines the search. A *matched-filter* search for binary mergers and a *cross-correlation* search for stochastic signals are both derived from the same likelihood function, the difference being that the former uses a parameterized, deterministic template, while the latter uses a stochastic template. Hopefully, by the end of this article, the reader will see that the plethora of searches for different types of backgrounds, using different types of detectors, and using different statistical inference frameworks are not all that different after all.

### Outline

The rest of the article is organized as follows: We begin in Sect. 2 by specifying the quantities that one uses to characterize a stochastic gravitational-wave background. In Sect. 3, we give an overview of statistical inference by comparing and contrasting how the Bayesian and frequentist formalisms address issues related to hypothesis testing, model selection, setting upper limits, parameter estimation, etc. We then illustrate these concepts in the context of a very simple toy problem. In Sect. 4, we introduce the key concept of correlation, which forms the basis for the majority of detection methods used for gravitational-wave backgrounds, and show how these techniques arise naturally from the standard template-based approach. We derive the frequentist cross-correlation statistic for a simple example. We also describe how a null channel is useful when correlation methods are not possible.

In Sect. 5, we go into more detail regarding the different types of detectors. In particular, we calculate single-detector response functions and the associated antenna patterns for ground-based and space-based laser interferometers, spacecraft Doppler tracking, and pulsar timing measurements. (We do not discuss resonant bar detectors or CMB-based detection methods in this review article. However, current bounds from CMB observations will be reviewed in Sect. 10). By correlating the outputs of two such detectors, we obtain expressions for the correlation coefficient (or *overlap reduction function*) for a Gaussian-stationary, unpolarized, isotropic background as a function of the separation and orientation of the two detectors. In Sect. 6, we discuss optimal filtering. Section 7 extends the analysis of the previous sections to *anisotropic* backgrounds. Here we describe several different analyses that produce maps of the gravitational-wave sky: (i) a frequentist gravitational-wave radiometer search, which is optimal for point sources, (ii) searches that decompose the gravitational-wave power on the sky in terms of spherical harmonics, and (iii) a phase-coherent search that can map both the amplitude and phase of a gravitational-wave background at each location on the sky. In Sect. 8, we discuss searches for: (i) non-Gaussian backgrounds, (ii) circularly-polarized backgrounds, and (iii) backgrounds having non-standard (i.e., non-general-relativity) polarization modes. We also briefly describe extensions of the cross-correlation search method to look for *non-stochastic-background-type* signals—in particular, long-duration unmodelled transients and continuous (nearly-monochromatic) gravitational-wave signals from sources like Sco X-1.

In Sect. 9, we discuss real-world complications introduced by irregular sampling, non-stationary and non-Gaussian detector noise, and correlated environmental noise (e.g., Schumann resonances). We also describe what one can do if one has only a single detector, as is the case for LISA. Finally, we conclude in Sect. 10 by discussing prospects for detection, including detection sensitivity curves and current observational results.

We also include several appendices: In Appendix A we discuss different polarization basis tensors, and a Stokes’ parameter characterization of gravitational-waves. In Appendices B and C, we summarize some standard statistical results for a Gaussian random variable, and then discuss how to define and test for non-stationarity and non-Gaussianity. In Appendix D we describe the relationship between continuous functions of time and frequency and their discretely-sampled counterparts. Appendices E, F, G are adapted from Gair et al. (2015), with details regarding spin-weighted scalar, vector, and tensor spherical harmonics. Finally, Appendix H gives a “Rosetta stone” for translating back and forth between different response function conventions for gravitational-wave backgrounds.

## Characterizing a stochastic gravitational-wave background

When you can measure what you are speaking about, and express it in numbers, you know something about it, when you cannot express it in numbers, your knowledge is of a meager and unsatisfactory kind; it may be the beginning of knowledge, but you have scarely, in your thoughts, advanced to the stage of science. *William Thomson, Baron Kelvin of Largs*

In this section, we define several key quantities (e.g., fractional energy density spectrum, characteristic strain, distribution of gravitational-wave power on the sky), which are used to characterize a stochastic background of gravitational radiation. The definitions are appropriate for both isotropic and anisotropic backgrounds. Our approach is similar to that found in Allen and Romano (1999) for isotropic backgrounds and for the standard polarization basis. For the plane-wave decomposition in terms of tensor spherical harmonics, we follow Gair et al. (2014, 2015). Detailed derivations can be found in those papers.

### When is a gravitational-wave signal stochastic?

The standard “textbook” definition of a stochastic background of gravitational radiation is *a random gravitational-wave signal produced by a large number of weak, independent, and unresolved sources*. To say that it is random means that it can be characterized only statistically, in terms of expectation values of the field variables or, equivalently, in terms of the Fourier components of a plane-wave expansion of the metric perturbations (Sect. 2.3.1). If the number of independent sources is sufficiently large, the background will be Gaussian by the central limit theorem. Knowledge of the first two moments of the distribution will then suffice to determine all higher-order moments (Appendix B). For non-Gaussian backgrounds, third and/or higher-order moments will also be needed.

Although there is general agreement with the above definition, there has been some confusion and disagreement in the literature (Rosado 2011; Regimbau and Mandic 2008; Regimbau and Hughes 2009; Regimbau 2011) regarding some of the defining properties of a stochastic background. This is because terms like *weak* and *unresolved* depend on details of the observation (e.g., the sensitivity of the detector, the total observation time, etc.), which are not intrinsic properties of the background. So the answer to the question “When is a gravitational-wave signal stochastic?” is not as simple or obvious as it might initially seem.

In Cornish and Romano (2015), we addressed this question in the context of searches for gravitational-wave backgrounds produced by a population of astrophysical sources. We found that it is best to give *operational* definitions for these properties, framed in the context of Bayesian inference. We will discuss Bayesian inference in more detail in Sect. 3, but for now the most important thing to know is that by using Bayesian inference we can calculate the probabilities of different signal-plus-noise models, given the observed data. The signal-plus-noise model with the largest probability is the preferred model, i.e., the one that is most consistent with the data. This is the essence of Bayesian model selection.

So we define a signal to be *stochastic* if a Bayesian model selection calculation prefers a stochastic signal model over any deterministic signal model. We also define a signal to be *resolvable* if it can be decomposed into *separate* (e.g., non-overlapping in either time or frequency) and *individually detectable* signals, again in a Bayesian model selection sense.^3^ If the background is associated with the superposition of signals from many astrophysical sources—as we expect for the population of binary black holes which gave rise to GW150914 and GW151226—then we should *subtract out* any bright deterministic signals that standout above the lower-amplitude background, leaving behind a residual non-deterministic signal whose statistical properties we would like to determine. In the context of Bayesian inference, this ‘subtraction’ is done by allowing *hybrid* signal models, which consist of both parametrized deterministic signals and non-deterministic backgrounds. By using such hybrid models we can investigate the statistical properties of the residual background without the influence of the resolvable signals.

We will return to these ideas in Sect. 8.1, when we discuss searches for non-Gaussian backgrounds in more detail.

### Plane-wave expansions

Gravitational waves are time-varying perturbations to the spacetime metric, which propagate at the speed of light. In transverse-traceless coordinates, the metric perturbations $h_{ab}(t,\vec {x})$ corresponding to a gravitational-wave background can be written as a superposition of sinusoidal plane waves having frequency *f*, and coming from different directions $\hat{n}$ on the sky:^4^

2.1 $$\begin{aligned} h_{ab}(t,{\vec {x}}) = \int _{-\infty }^\infty df\> \int d^2\Omega _{\hat{n}}\> h_{ab}(f,\hat{n}) e^{i2\pi f(t+\hat{n}\cdot {\vec {x}}/c)}. \end{aligned}$$

For a stochastic background, the metric perturbations $h_{ab}(t,{\vec {x}})$ and hence the Fourier coefficients $h_{ab}(f,\hat{n})$ are random variables, whose probability distributions define the statistical properties of the background.

#### Polarization basis

Typically, one expands the Fourier coefficients $h_{ab}(f,\hat{n})$ in terms of the standard $+$ and $\times$ polarization tensors:

2.2 $$\begin{aligned} h_{ab}(f,\hat{n}) =h_+(f,\hat{n}) e^+_{ab}(\hat{n}) + h_\times (f,\hat{n}) e^\times _{ab}(\hat{n}), \end{aligned}$$

where

**Fig. 2:**
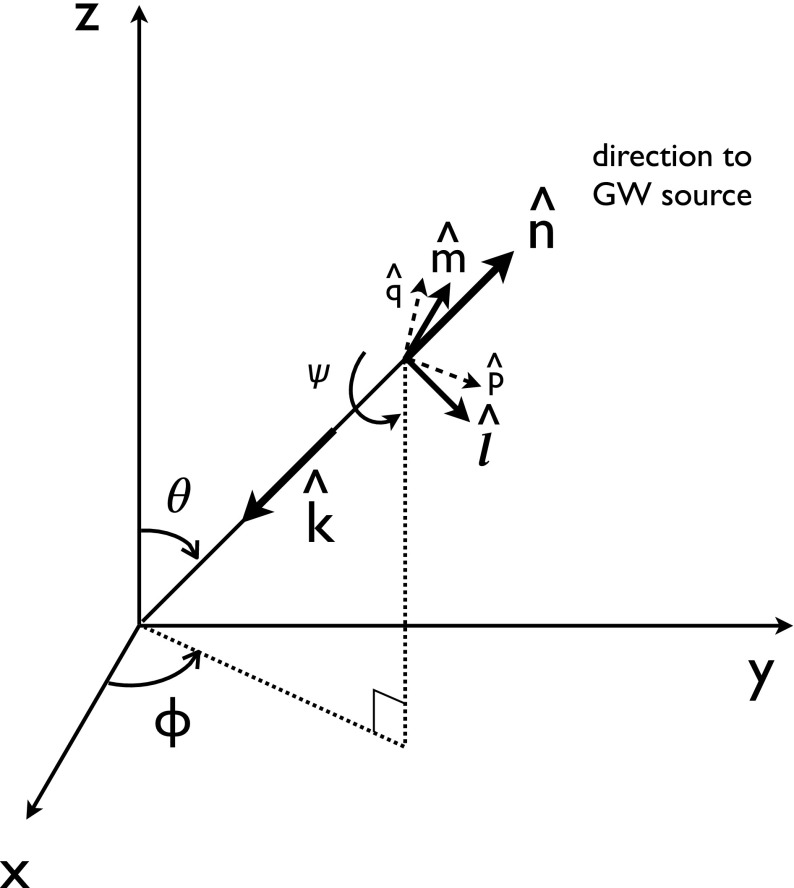
Our convention for the unit vectors $\{\hat{n}, \hat{l}, \hat{m}\}$ in terms of which the polarization basis tensors $e^+_{ab}(\hat{n})$ and $e^\times _{ab}(\hat{n})$ are defined. The unit vector $\hat{n}$ points in the direction of the gravitational-wave source (the gravitational wave propagates in direction $\hat{k}=-\hat{n}$); $\hat{l}=\hat{\theta }$ and $\hat{m}=\hat{\phi }$ are two unit vectors that lie in the plane perpendicular to $\hat{n}$. Another choice for the polarization basis tensors, defined in terms of the ‘rotated’ unit vectors $\hat{p}$ and $\hat{q}$, is given in Appendix A

2.3 $$\begin{aligned} \begin{aligned} e_{ab}^+(\hat{n})&=\hat{l}_a\hat{l}_b-\hat{m}_a\hat{m}_b, \\ e_{ab}^\times (\hat{n})&=\hat{l}_a\hat{m}_b+\hat{m}_a\hat{l}_b, \end{aligned} \end{aligned}$$

and $\hat{l}$, $\hat{m}$ are the standard angular unit vectors tangent to the sphere:

2.4 $$\begin{aligned} \begin{aligned} \hat{n}&=\sin \theta \cos \phi \,\hat{x} +\sin \theta \sin \phi \,\hat{y} +\cos \theta \,\hat{z} \equiv \hat{r}, \\ \hat{l}&=\cos \theta \cos \phi \,\hat{x} +\cos \theta \sin \phi \,\hat{y} -\sin \theta \,\hat{z} \equiv \hat{\theta }, \\ \hat{m}&=-\sin \phi \,\hat{x} +\cos \phi \,\hat{y} \equiv \hat{\phi }. \end{aligned} \end{aligned}$$

(See Fig. 2). Searches for stochastic backgrounds having alternative polarization modes, as predicted by modified (metric) theories of gravity, will be discussed in Sect. 8.3.

#### Tensor spherical harmonic basis

It is also possible to expand the Fourier coefficients $h_{ab}(f,\hat{n})$ in terms of the *gradient* and *curl* tensor spherical harmonics (Gair et al. 2014):

2.5 $$\begin{aligned} h_{ab}(f,\hat{n}) =\sum _{l=2}^\infty \sum _{m=-l}^l \left[ a^G_{(lm)}(f)Y^G_{(lm)ab}(\hat{n}) +a^C_{(lm)}(f)Y^C_{(lm)ab}(\hat{n})\right] , \end{aligned}$$

where

2.6 $$\begin{aligned} \begin{aligned} Y^G_{(lm)ab}&= {}^{(2)}\!N_l \left( Y_{(lm);ab} - \frac{1}{2} g_{ab} Y_{(lm);c}{}^{c} \right) ,\\ Y^C_{(lm)ab}&= \frac{{}^{(2)}\!N_l}{2} \left( Y_{(lm);ac}\epsilon ^c{}_b + Y_{(lm);bc} \epsilon ^c{}_a \right) . \end{aligned} \end{aligned}$$

In the above expressions, a semi-colon denotes covariant differentiation, $g_{ab}$ is the metric tensor on the sphere, and $\epsilon _{ab}$ is the Levi-Civita anti-symmetric tensor. In standard spherical coordinates $(\theta ,\phi )$,

2.7 $$\begin{aligned} g_{ab}=\left( \begin{array}{cc} 1&{}\quad 0\\ 0&{}\quad \sin ^2\theta \\ \end{array} \right) , \quad \epsilon _{ab} = \sqrt{g} \left( \begin{array}{cc} 0&{}\quad 1\\ -1&{}\quad 0\\ \end{array}\right) , \quad \sqrt{g}=\sin \theta . \end{aligned}$$

The normalization constant

2.8 $$\begin{aligned} {}^{(2)}\!N_l = \sqrt{\frac{2 (l-2)!}{(l+2)!}}, \end{aligned}$$

was chosen so that $\{Y^G_{(lm)ab}(\hat{n}), Y^C_{(lm)ab}(\hat{n})\}$ is a set of orthonormal functions (with respect to the multipole indices *l* and *m*) on the 2-sphere. Appendix G contains additional details regarding gradient and curl spherical harmonics.

Note that we have adopted the notational convention used in the CMB literature, e.g., Kamionkowski et al. (1997), by putting parentheses around the *lm* indices to distinguish them from the spatial tensor indices *a*, *b*, etc. In addition, summations over *l* and *m* start at $l=2$, and not $l=0$ as would be the case for the expansion of a scalar field on the 2-sphere in terms of ordinary (i.e., undifferentiated) spherical harmonics. In what follows, we will use $\sum _{(lm)}$ as shorthand notation for $\sum _{l=2}^\infty \sum _{m=-l}^l$ unless indicated otherwise.

#### Relating the two expansions

The gradient and curl spherical harmonics have been used extensively in the CMB community for decomposing CMB-polarization maps in terms of *E*-modes and *B*-modes (corresponding to the gradient and curl spherical harmonics). The most relevant property of the gradient and curl spherical harmonics is that they transform like combinations of spin-weight $\pm 2$ fields with respect to rotations of an orthonormal basis at points on the 2-sphere. Explicitly,

2.9 $$\begin{aligned} Y^G_{(lm)ab}(\hat{n}) \pm i Y^C_{(lm)ab}(\hat{n})&=\frac{1}{\sqrt{2}} \left( e_{ab}^+(\hat{n}) \pm i e_{ab}^\times (\hat{n})\right) \,{}_{\mp 2}Y_{lm}(\hat{n}), \end{aligned}$$

where ${}_{\pm 2}Y_{lm}(\hat{n})$ are the spin-weight $\pm 2$ spherical harmonics (Appendix E). Using this relationship between the tensor spherical harmonic and $(+,\times )$ polarization bases, one can show (Gair et al. 2014):

2.10 $$\begin{aligned} h_+(f,\hat{n})\pm i h_\times (f,\hat{n}) =\frac{1}{\sqrt{2}} \sum _{(lm)} \left( a^G_{(lm)}(f)\pm i a^C_{(lm)}(f)\right) \, {}_{\pm 2}Y_{lm}(\hat{n}), \end{aligned}$$

or, equivalently,

2.11 $$\begin{aligned} a^G_{(lm)}(f)\pm i a^C_{(lm)}(f) = \sqrt{2} \int \mathrm{d}^2\Omega _{\hat{n}}\> \left( h_+(f,\hat{n}) \pm i h_\times (f,\hat{n})\right) \, {}_{\pm 2}Y_{lm}^*(\hat{n}). \end{aligned}$$

These two expressions allow us to go back and forth between the expansion coefficients for the two different bases.

### Statistical properties

The statistical properties of a stochastic gravitational-wave background are specified in terms of the probability distribution or *moments* (Appendix B) of the metric perturbations:

2.12 $$\begin{aligned} \langle h_{ab}(t,{\vec {x}})\rangle , \quad \langle h_{ab}(t,{\vec {x}})h_{cd}(t', {\vec {x}}')\rangle , \quad \langle h_{ab}(t,{\vec {x}})h_{cd}(t', {\vec {x}}')h_{ef}(t'',{\vec {x}}'')\rangle , \ldots \qquad \end{aligned}$$

or similar expressions in terms of the Fourier coefficients $h_A(f,\hat{n})$, where $A\equiv \{+,\times \}$ labels the standard polarization modes of general relativity, or $a^P_{(lm)}(f)$, where $P\equiv \{G,C\}$ and (*lm*) label the multipole components for the gradient and curl tensor spherical harmonic decomposition. Without loss of generality we can assume that the background has zero mean:

2.13 $$\begin{aligned} \langle h_{ab}(t,{\vec {x}})\rangle =0 \quad {\Leftrightarrow }\quad \langle h_A(f,\hat{n})\rangle =0 \quad {\Leftrightarrow }\quad \langle a_{(lm)}^P(f)\rangle =0. \end{aligned}$$

We will also assume that the background is *stationary* (Appendix C). This means that all statistical quantities constructed from the metric perturbations at times *t*, $t'$, etc., depend only on the difference between times, e.g., $t-t'$, and not on the choice of time origin. We expect this to be true given that the age of the universe is roughly 9 orders of magnitude larger than realistic observation times, ${\sim }10~\mathrm {year}$. It is thus unlikely that a stochastic gravitational-wave background has statistical properties that vary over the time scale of the observation.

For Gaussian backgrounds we need only consider quadratic expectation values, since all higher-order moments are either zero or can be written in terms of the quadratic moments (Appendix B). For non-Gaussian backgrounds (Sect. 8.1), third and/or higher order moments will also be needed.

Beyond our assumption of stationarity, the specific form of the expectation values will depend, in general, on the source of the background. For example, a cosmological background produced by the superposition of a large number of independent gravitational-wave signals from the early Universe is expected to be Gaussian (via the central limit theorem), as well as isotropically-distributed on the sky. Contrast this with the superposition of gravitational waves produced by unresolved Galactic white-dwarf binaries radiating in the LISA band ($10^{-4}~\mathrm{Hz}$ to $10^{-1}~\mathrm{Hz}$). Although this confusion-limited astrophysical foreground is also expected to be Gaussian and stationary, it will have an *anisotropic distribution*, following the spatial distribution of the Milky Way. The anistropy will be encoded as a modulation in the LISA output, due to the changing antenna pattern of the LISA constellation in its yearly orbit around the Sun. Hence, different sources will give rise to different statistical distributions, which we will need to consider when formulating our data analysis strategies.

#### Quadratic expectation values for Gaussian-stationary backgrounds

The simplest type of stochastic background will be Gaussian-stationary, unpolarized, and spatially homogenous and isotropic. The quadratic expectation values for such a background are then

2.14 $$\begin{aligned} \left\langle h_A(f,\hat{n}) h_{A'}^*(f',\hat{n}')\right\rangle =\frac{1}{16\pi }S_h(f) \delta (f-f') \delta _{AA'} \delta ^2(\hat{n},\hat{n}'), \end{aligned}$$

or, equivalently,

2.15 $$\begin{aligned} \left\langle a^P_{(lm)}(f)a^{P'*}_{(l'm')}(f')\right\rangle =\frac{1}{8\pi } S_h(f)\delta (f-f')\delta ^{PP'}\delta _{ll'}\delta _{mm'}. \end{aligned}$$

The numerical factors out front have been included so that $S_h(f)$ has the interpretation of being the one-sided gravitational-wave *strain power spectral density* function (units of $\mathrm{strain}^2/\mathrm{Hz}$), summed over both polarizations and integrated over the sky. The factor of $\delta (f-f')$ arises due to our assumption of stationarity; the factor of $\delta _{AA'}$ (or $\delta ^{PP'}$) is due to our assumption that the polarization modes are statistically independent of one another and have no preferred component; and the factor of $\delta ^2(\hat{n},\hat{n}')$ (or $\delta _{ll'}\delta _{mm'}$) is due to our assumption of spatial homogeneity and isotropy.

Anisotropic, unpolarized, Gaussian-stationary backgrounds, whose radiation from different directions on the sky are uncorrelated with one another, are also simply represented in terms of the quadratic expectation values:

2.16 $$\begin{aligned} \left\langle h_A(f,\hat{n}) h_{A'}^*(f',\hat{n}')\right\rangle =\frac{1}{4}\mathcal{P}(f,\hat{n}) \delta (f-f') \delta _{AA'} \delta ^2(\hat{n},\hat{n}'). \end{aligned}$$

The function $\mathcal{P}(f,\hat{n})$ describes the spatial distribution of gravitational-wave power on the sky at frequency *f*. It is related to $S_h(f)$ via

2.17 $$\begin{aligned} S_h(f) = \int d^2\Omega _{\hat{n}}\> \mathcal{P}(f,\hat{n}). \end{aligned}$$

The corresponding expectation values in terms of the tensor spherical harmonic expansion coefficients $a^P_{(lm)}(f)$ are more complicated, since an individual mode in this basis corresponds to a gravitational-wave background whose radiation is correlated between different angular directions on the sky. (See Gair et al. (2014) for a discussion of backgrounds that have such correlations). We will discuss searches for anisotropic backgrounds in more detail in Sect. 7.

More general Gaussian-stationary backgrounds (e.g., polarized, statistically isotropic but with correlated radiation, etc.) can be represented by appropriately changing the right-hand-side of the quadratic expectation values. However, for the remainder of this section and for most of the article, we will consider “vanilla” isotropic backgrounds, whose quadratic expectation values (2.14) or (2.15) are completely specified by the power spectral density $S_h(f)$.

### Fractional energy density spectrum

The gravitational-wave strain power spectral density $S_h(f)$ is simply related to the fractional energy density spectrum in gravitational waves $\Omega _\mathrm{gw}(f)$, see e.g., Allen and Romano (1999):

2.18 $$\begin{aligned} S_h(f) = \frac{3 H_0^2}{2\pi ^2}\frac{\Omega _\mathrm{gw}(f)}{f^3}, \end{aligned}$$

where

2.19 $$\begin{aligned} \Omega _\mathrm{gw}(f) =\frac{1}{\rho _c}\frac{d\rho _\mathrm{gw}}{d\ln f}. \end{aligned}$$

Here $d\rho _\mathrm{gw}$ is the energy density in gravitational waves contained in the frequency interval *f* to $f+df$, and $\rho _c\equiv 3c^2 H_0^2/8\pi G$ is the critical energy density need to close the universe. The *total* energy density in gravitational waves normalized by the critical energy density is thus

2.20 $$\begin{aligned} \Omega _\mathrm {gw} = \int _{f=0}^{f_\mathrm {max}} d(\ln f)\>\Omega _\mathrm {gw}(f) , \end{aligned}$$

where $f_\mathrm {max}$ is some maximum cutoff frequency (e.g., associated with the Planck scale), beyond which our current understanding of gravity breaks down. $\Omega _\mathrm {gw}$ can be compared, for example, to the total fractional energy density $\Omega _\mathrm {b}$, $\Omega _{\Lambda }$, in baryons, dark energy, etc. Since $\rho _\mathrm {c}$ involves the Hubble constant, one sometimes writes $H_0=h_0\,100\mathrm {\ km\ s^{-1}\ Mpc^{-1}}$, and then absorbs a factor of $h_0^2$ in $\Omega _\mathrm {gw}(f)$. The quantity $h_0^2\,\Omega _\mathrm {gw}(f)$ is then *independent* of the value of the Hubble constant. However, since recent measurements by Planck (Ade et al. 2015b; ESA 2016c) have shown that $h_0=0.68$ to a high degree of precision, we have assumed this value in this review article and quote limits directly on $\Omega _\mathrm {gw}(f)$ (Sect. 10). The specific functional form for $\Omega _\mathrm {gw}(f)$ depends on the source of the background, as we shall see explicitly below.

### Characteristic strain

Although the fractional energy density spectrum $\Omega _\mathrm{gw}(f)$ completely characterizes the statistical properties of a Gaussian-stationary isotropic background, it is often convenient to work with the (dimensionless) characteristic strain amplitude $h_c(f)$ defined by

2.21 $$\begin{aligned} h_c(f) \equiv \sqrt{f S_h(f)}. \end{aligned}$$

It is related to $\Omega _\mathrm{gw}(f)$ via:

2.22 $$\begin{aligned} \Omega _\mathrm{gw}(f) = \frac{2\pi ^2}{3 H_0^2}f^2 h^2_c(f). \end{aligned}$$

Several theoretical models of gravitational-wave backgrounds predict characteristic strains that have a power-law form

2.23 $$\begin{aligned} h_c(f) =A_\alpha \left( \frac{f}{f_\mathrm{ref}}\right) ^\alpha , \end{aligned}$$

where $\alpha$ is spectral index and $f_\mathrm{ref}$ is typically set to $1/\mathrm{year}$. (There is no sum over $\alpha$ in the above expression, and no sum over $\beta$ in the following expression). Using Eqs. (2.22) and (2.23) it follows that

2.24 $$\begin{aligned} \Omega _\mathrm{gw}(f) = \Omega _\beta \left( \frac{f}{f_\mathrm{ref}}\right) ^\beta , \end{aligned}$$

where

2.25 $$\begin{aligned} \Omega _\beta = \frac{2\pi ^2}{3 H_0^2} f_\mathrm{ref}^2\,A_\alpha ^2, \quad \beta = 2\alpha +2. \end{aligned}$$

For inflationary backgrounds relevant for cosmology, it is often assumed that $\Omega _\mathrm{gw}(f)=\mathrm{const}$, for which $\beta =0$ and $\alpha =-1$. For a background arising from binary coalescence, $\Omega _\mathrm{gw}(f) \propto f^{2/3}$, for which $\beta = 2/3$ and $\alpha =-2/3$. This power-law dependence is applicable to super-massive black-hole binary (SMBHB) coalescences targeted by pulsar timing observations as well as to compact binary coalescences relevant for ground-based and space-based detectors.

## Statistical inference

If your experiment needs statistics, you ought to have done a better experiment. *Ernest Rutherford*

In this section, we review statistical inference from both the Bayesian and frequentist perspectives. Our discussion of frequentist and Bayesian upper limits, and the example given in Sect. 3.5 comparing Bayesian and frequentist analyses is modelled in part after Röver et al. (2011). Readers interested in more details about Bayesian statistical inference should see, e.g., Howson and Urbach (1991), Howson and Urbach (2006), Jaynes (2003), Gregory (2005) and Sivia and Skilling (2006). For a description of frequentist statistics, we recommend Helstrom (1968), Wainstein and Zubakov (1971) and Feldman and Cousins (1998).

### Introduction to Bayesian and frequentist inference

Statistical inference can be used to answer questions such as “Is a gravitational-wave signal present in the data?” and, if so, “What are the physical characteristics of the source?” These questions are addressed using the techniques of classical (also known as *frequentist*) inference and *Bayesian* inference. Many of the early theoretical studies and observational papers in gravitational-wave astronomy followed the frequentist approach, but the use of Bayesian inference is growing in popularity. Moreover, many contemporary analyses cannot be classified as purely frequentist or Bayesian.

The textbook definition states that the difference between the two approaches comes down to their different interpretations of probability: for frequentists, probabilities are fundamentally related to frequencies of events, while for Bayesians, probabilities are fundamentally related to our own knowledge about an event. For example, when inferring the mass of a star, the frequentist interpretation is that the star has a true, fixed (albeit unknown) mass, so it is meaningless to talk about a probability distribution for it. Rather, the uncertainty is in the data, and the relevant probability is that of observing the data *d*, given that the star has mass *m*. This probability distribution is the *likelihood*, denoted $p(d\vert m)$. In contrast, in the Bayesian interpretation the data are known (after all, it is what is measured!), and the mass of the star is what we are uncertain about,^5^ so the relevant probability is that the mass has a certain value, given the data. This probability distribution is the *posterior*, $p(m \vert d)$. The likelihood and posterior are related via Bayes’ theorem:

3.1 $$\begin{aligned} p( m \vert d) = \frac{ p( d \vert m) p(m)}{p(d)} , \end{aligned}$$

where *p*(*m*) is the prior probability distribution for *m*, and the normalization constant,

3.2 $$\begin{aligned} p(d) = \int p( d\vert m) p(m) \, dm , \end{aligned}$$

is the *marginalized likelihood*, or *evidence*. For uniform (flat) priors the frequentist confidence intervals for the parameters will coincide with the Bayesian credible intervals, but the interpretation remains quiet distinct.

The choice of prior probability distributions is a source of much consternation and debate, and is often cited as a weakness of the Bayesian approach. But the choice of probability distribution for the likelihood (which is also important for the frequentist approach) is often no less fraught. The prior quantifies what we know about the range and distribution of the parameters in our model, while the likelihood quantifies what we know about our measurement apparatus, and, in particular, the nature of the measurement noise. The choice of prior is especially problematic in a new field where there is little to guide the choice. For example, electromagnetic observations and population synthesis models give some guidance about black hole masses, but the mass range and distribution is currently not well constrained. The choice of likelihood can also be challenging when the measurement noise deviates from the stationary, Gaussian ideal. More details related to the choice of likelihood and choice of prior will be given in Sect. 3.6.

In addition to parameter estimation, statistical inference is used to select between competing models, or hypotheses, such as, “is there a gravitational-wave signal in the data or not?” Thanks to GW150914 and GW151226, we know that gravitational-wave signals *are* already present in existing data sets, but most are at levels where we are unable to distinguish them from noise processes. For detection we demand that a model for the data that includes a gravitational-wave signal be favored over a model having no gravitational-wave signal. In Bayesian inference a detection might be announced when the odds ratio between models with and without gravitational-wave signals gets sufficiently large, while in frequentist inference a detection might be announced when the *p*-value for some test statistic is less than some prescribed threshold. These different approaches to deciding whether or not to claim a detection (e.g., Bayesian model selection or frequentist hypothesis testing), as well as differences in regard to parameter estimation, are described in the following subsections. Table 2 provides an overview of the key similarities and differences between frequentist and Bayesian inference, to be described in detail below.

**Table 2:** Comparison of frequentist and Bayesian approaches to statistical inference

Frequentist	Bayesian
Probabilities assigned only to propositions about outcomes of repeatable experiments (i.e., random variables), not to hypotheses or parameters which have fixed but unknown values	Probabilities can be assigned to hypotheses and parameters since probability is degree of belief (or confidence, plausibility) in any proposition
Assumes measured data are drawn from an underlying probability distribution, which assumes the truth of a particular hypothesis or model (likelihood function)	Same
Constructs a statistic to estimate a parameter or to decide whether or not to claim a detection	Needs to specify prior degree of belief in a particular hypothesis or parameter
Calculates the probability distribution of the statistic (sampling distribution)	Uses Bayes’ theorem to update the prior degree of belief in light of new data (i.e., likelihood “plus” prior yields posterior)
Constructs confidence intervals and *p*-values for parameter estimation and hypothesis testing	Constructs posteriors and odds ratios for parameter estimation and hypothesis testing/model comparison

See Sects. 3.2 and 3.3 for details

### Frequentist statistics

As mentioned above, classical or *frequentist* statistics is a branch of statistical inference that interprets probability as the “long-run relative occurrence of an event in a set of identical experiments.” Thus, for a frequentist, probabilities can only be assigned to propositions about outcomes of (in principle) repeated experiments (i.e., *random variables*) and not to hypotheses or parameters describing the state of nature, which have fixed but unknown values. In this interpretation, the measured data are drawn from an underlying probability distribution, which assumes the truth of a particular hypothesis or model. The probability distribution for the data is just the likelihood function, which we can write as *p*(*d*|*H*), where *d* denotes the data and *H* denotes an hypothesis.

Statistics play an important role in the frequentist framework. These are random variables constructed from the data, which typically estimate a signal parameter or indicate how well the data fit a particular hypothesis. Although it is common to construct statistics from the likelihood function (e.g., the maximum-likelihood statistic for a particular parameter, or the maximum-likelihood ratio to compare a signal-plus-noise model to a noise-only model), there is no a priori restriction on the form of a statistic other than it be *some* function of the data. Ultimately, it is the goal of the analysis and the cleverness of the data analyst that dictate which statistic (or statistics) to use.

To make statistical inferences in the frequentist framework requires knowledge of the probability distribution (also called the *sampling distribution*) of the statistic. The sampling distribution can either be calculated analytically (if the statistic is sufficiently simple) or via Monte Carlo simulations, which effectively construct a histogram of the values of the statistic by simulating many independent realizations of the data. Given a statistic and its sampling distribution, one can then calculate either *confidence intervals* for parameter estimation or *p*-values for hypothesis testing. (These will be discussed in more detail below). Note that a potential problem with frequentist statistical inference is that the sampling distribution depends on data values that were *not* actually observed, which is related to how the experiment was carried out *or might have been* carried out. The so-called *stopping problem* of frequentist statistics is an example of such a problem (Howson and Urbach 2006).

#### Frequentist hypothesis testing

Suppose, as a frequentist, you want to test the hypothesis $H_1$ that a gravitational-wave signal, having some fixed but unknown amplitude $a>0$, is present in the data. Since you cannot assign probabilities to hypotheses or to parameters like *a* as a frequentist, you need to introduce instead an alternative (or *null*) hypothesis $H_0$, which, for this example, is the hypothesis that there is no gravitational-wave signal in the data (i.e., that $a=0$). You then argue for $H_1$ by arguing *against* $H_0$, similar to proof by contradiction in mathematics. Note that $H_1$ is a *composite* hypothesis since it depends on a range of values of the unknown parameter *a*. It can be written as the union, $H_1=\cup _{a>0} H_a$, of a set of simple hypotheses $H_a$ each corresponding to a single fixed value of the parameter *a*.

To rule either in favor or against $H_0$, you construct a statistic $\Lambda$, called a *test* or *detection statistic*, on which the statistical test will be based. As mentioned above, you will need to calculate analytically or via Monte Carlo simulations the sampling distribution for $\Lambda$ under the assumption that the null hypothesis is true, $p(\Lambda |H_0)$. If the observed value of $\Lambda$ lies far out in the tails of the distribution, then the data are most likely not consistent with the assumption of the null hypothesis, so you reject $H_0$ (and thus accept $H_1$) at the $p*100$% level, where

3.3 $$\begin{aligned} p\equiv \mathrm{Prob}(\Lambda >\Lambda _\mathrm{obs}|H_0) \equiv \int _{\Lambda _\mathrm{obs}}^\infty p(\Lambda |H_0)\,d\Lambda . \end{aligned}$$

**Fig. 3:**
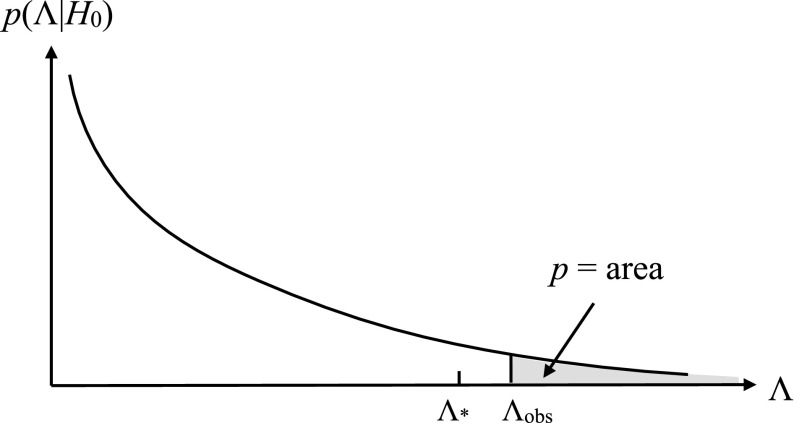
Definition of the *p*-value (or significance) for frequentist hypothesis testing. The value of *p* equals the area under the probability distribution $p(\Lambda |H_0)$ for $\Lambda \ge \Lambda _\mathrm{obs}$

This is the so-called *p*-value (or *significance*) of the test; it is illustrated graphically in Fig. 3. The *p*-value required to reject the null hypothesis determines a *threshold* $\Lambda _*$, above which you reject $H_0$ and accept $H_1$ (e.g., claim a detection). It is related to the *false alarm probability* for the test as we explain below.

The above statistical test is subject to two types of errors: (i) type I or *false alarm* errors, which arise if the data are such that you reject the null hypothesis (i.e., $\Lambda _\mathrm{obs}>\Lambda _*$) when it is actually true, and (ii) type II or *false dismissal* errors, which arise if the data are such that you accept the null hypothesis (i.e., $\Lambda _\mathrm{obs}<\Lambda _*$) when it is actually false. The false alarm probability $\alpha$ and false dismissal probability $\beta (a)$ are given explicitly by

3.4 $$\begin{aligned} \alpha&\equiv \mathrm{Prob}(\Lambda >\Lambda _*|H_0), \end{aligned}$$

3.5 $$\begin{aligned} \beta (a)&\equiv \mathrm{Prob}(\Lambda <\Lambda _*|H_a), \end{aligned}$$

where *a* is the amplitude of the gravitational-wave signal, assumed to be present under the assumption that $H_1$ is true. To calculate the false dismissal probability $\beta (a)$, one needs the sampling distribution of the test statistic assuming the presence of a signal with amplitude *a*.

Different test statistics are judged according to their false alarm and false dismissal probabilities. Ideally, you would like your statistical test to have false alarm and false dismissal probabilities that are both as small as possible. But these two properties compete with one another as setting a larger threshold value to minimize the false alarm probability will increase the false dismissal probability. Conversely, setting a smaller threshold value to minimize the false dismissal probability will increase the false alarm probability.

In the context of gravitational-wave data analysis, the gravitational-wave community is (at least initially) reluctant to falsely claim detections. Hence the false alarm probability is set to some very low value. The best statistic then is the one that minimizes the false dismissal probability (i.e., maximizes detection probability) for fixed false alarm. This is the *Neyman*–*Pearson criterion*. For medical diagnosis, on the other hand, a doctor is very reluctant to falsely dismiss an illness. Hence the false dismissal probability will be set to some very low value. The best statistic then is the one which minimizes the false alarm probability for fixed false dismissal.

#### Frequentist detection probability

**Fig. 4:**
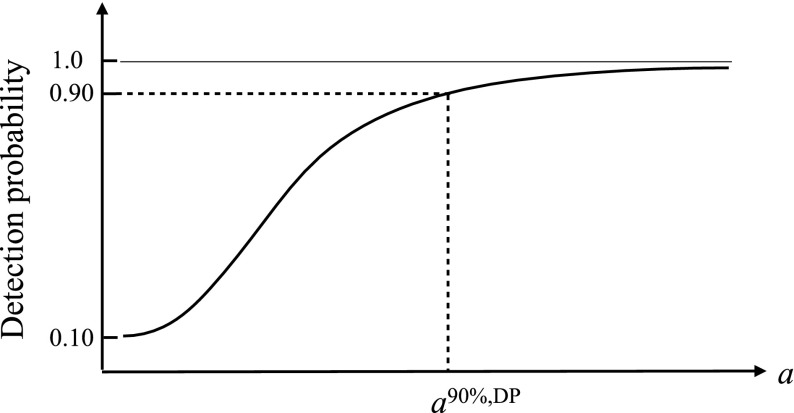
Detection probability as a function of the signal amplitude for a false alarm probability equal to $10\%$. The value of *a* needed for 90% detection probability is indicated by the *dashed vertical line* and is denoted by $a^{90\%,\mathrm{DP}}$

The value $1-\beta (a)$ is called the *detection probability* or *power* of the test. It is the fraction of times that the test statistic $\Lambda$ correctly identifies the presence of a signal of amplitude *a* in the data, for a fixed false alarm probability $\alpha$ (which sets the threshold $\Lambda _*$). A plot of detection probability versus signal strength is often used to show how strong a signal has to be in order to detect it with a certain probability. Since detection probability does not depend on the observed data—it depends only on the sampling distribution of the test statistic and a choice for the false alarm probability—detection probability curves are often used as a *figure-of-merit* for proposed search methods for a signal. Figure 4 shows a detection probability curve, with the value of *a* needed to be detectable with 90% frequentist probability indicated by the dashed vertical line. We will denote this value of *a* by $a^{90\%,\mathrm{DP}}$. Note that as the signal amplitude goes to zero, the detection probability reduces to the false alarm probability $\alpha$, which for this example was chosen to be 0.10.

#### Frequentist upper limits

In the absence of a detection (i.e., if the observed value of the test statistic is less than the detection threshold $\Lambda _*$), one can still set a bound (called an *upper limit*) on the strength of the signal that one was trying to detect. The upper limit depends on the observed value of the test statistic, $\Lambda _\mathrm{obs}$, and a choice of confidence level, $\mathrm {CL}$, interpreted in the frequentist framework as the long-run relative occurrence for a set of repeated identical experiments. For example, one defines the 90% confidence-level upper limit $a^{90\%,\mathrm{UL}}$ as the minimum value of *a* for which $\Lambda \ge \Lambda _\mathrm{obs}$ at least 90% of the time:

3.6 $$\begin{aligned} \mathrm{Prob}(\Lambda \ge \Lambda _\mathrm{obs}| a\ge a^{90\%,\mathrm{UL}}, H_a) \ge 0.90. \end{aligned}$$

**Fig. 5:**
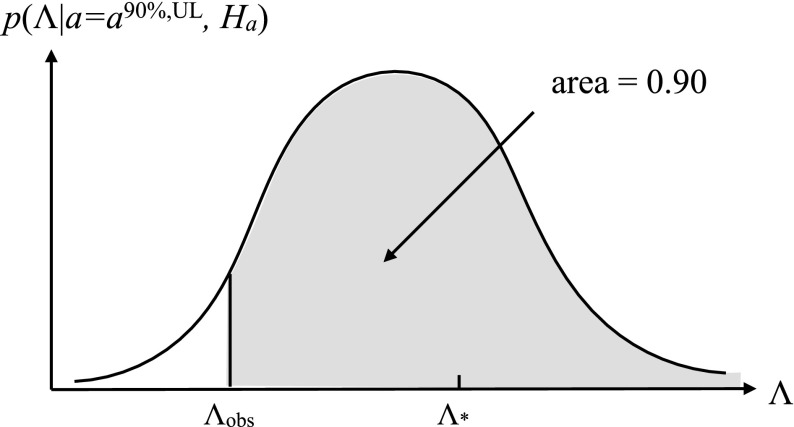
Graphical representation of a frequentist 90% confidence level upper limit. When $a=a^{90\%,\mathrm{UL}}$, the probability of obtaining a value of the detection statistic $\Lambda \ge \Lambda _\mathrm{obs}$ is equal to 0.90

In other words, if the signal has an amplitude $a^{90\%,\mathrm{UL}}$ or higher, we would have detected it in at least 90% of repeated observations. A graphical representation of a frequentist upper limit is given in Fig. 5.

#### Frequentist parameter estimation

The frequentist prescription for estimating the value of a particular parameter *a*, like the amplitude of a gravitational-wave signal, is slightly different than the method used to claim a detection. You need to first construct a statistic (called an *estimator*) $\hat{a}$ of the parameter *a* you are interested in. (This might be a maximum-likelihood estimator of *a*, but other estimators can also be used). You then calculate its sampling distribution $p(\hat{a}|a, H_a)$. Note that statements like

3.7 $$\begin{aligned} \mathrm{Prob}(a-\Delta< \hat{a} < a+\Delta )=0.95, \end{aligned}$$

which one constructs from $p(\hat{a}|a,H_a)$ make sense in the frequentist framework, since $\hat{a}$ is a random variable. Although the above inequality can be rearranged to yield

**Fig. 6:**
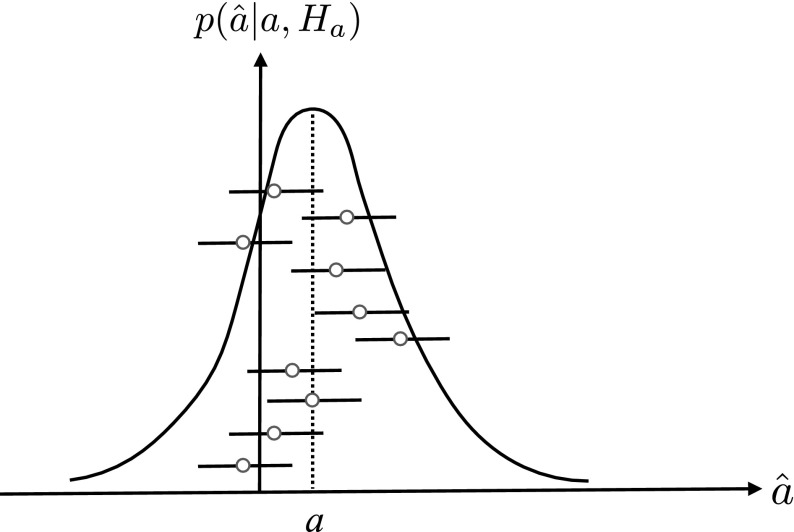
Definition of the frequentist confidence interval for parameter estimation. *Each circle* and *line* represents a measured interval $[\hat{a}-\Delta , \hat{a}+\Delta ]$. The set of all such intervals will contain the true value of the parameter *a* (indicated here by the *dotted vertical line*) $\mathrm {CL}*100\%$ of the time, where $\mathrm {CL}$ is the confidence level

3.8 $$\begin{aligned} \mathrm{Prob}(\hat{a}-\Delta< a < \hat{a}+\Delta )=0.95, \end{aligned}$$

this should *not* be interpreted as a statement about the probability of *a* lying within a particular interval $[\hat{a}-\Delta ,\hat{a}+\Delta ]$, since *a* is not a random variable. Rather, it should be interpreted as a probabilistic statement about the *set of intervals* $\{[\hat{a}-\Delta ,\hat{a}+\Delta ]\}$ for all possible values of $\hat{a}$. Namely, in a set of many repeated experiments, 0.95 is the fraction of the intervals that will contain the true value of the parameter *a*. Such an interval is called a $95\%$ *frequentist confidence interval*. This is illustrated graphically in Fig. 6.

It is important to point out that an estimator can sometimes take on a value of the parameter that is *not physically allowed*. For example, if the parameter *a* denotes the amplitude of a gravitational-wave signal (so physically $a\ge 0$), it is possible for $\hat{a} <0$ for a particular realization of the data. Note that there is nothing mathematically wrong with this result. Indeed, the sampling distribution for $\hat{a}$ specifies the probability of obtaining such values of $\hat{a}$. It is even possible to have a confidence interval $[\hat{a}-\Delta , \hat{a}+\Delta ]$ all of whose values are unphysical, especially if one is trying to detect a weak signal in noise. Again, this is mathematically allowed, but it is a little awkward to report a frequentist confidence interval that is completely unphysical. We shall see that within the Bayesian framework unphysical intervals and unphysical posteriors never arise, as a simple consequence of including a prior distribution on the parameter that requires $a > 0$.

#### Unified approach for frequentist upper limits and confidence intervals

Frequentists also have a way of avoiding unphysical or empty confidence intervals, which at the same time *unifies* the treatment of upper limits for null results and two-sided intervals for non-null results. This procedure, developed by Feldman and Cousins (1998), also solves the problem that the choice of an upper limit or two-sided confidence interval leads to intervals that do not have the proper coverage (i.e., the probability that an interval contains the true value of a parameter does not match the stated confidence level) if the choice of reporting an upper limit or two-sided confidence interval is *based on the data* and not decided upon before performing the experiment.

The basic idea underlying this unified approach to frequentist intervals is a new specification (or *ordering*) of the values of the random variable to include in the acceptance intervals for an unknown parameter. If we let *a* denote the parameter whose value we are trying to determine, and $\hat{a}$ be an estimator of *a* with sampling distribution $p(\hat{a}|a,H_a)$, then the choice of acceptance intervals becomes, for each value of *a*, how do we choose $[\hat{a}_1, \hat{a}_2]$ such that

3.9 $$\begin{aligned} \mathrm{Prob}(\hat{a}_1< \hat{a} < \hat{a}_2) \equiv \int _{\hat{a}_1}^{\hat{a}_2} p(\hat{a}|a, H_a)\,d\hat{a} =\mathrm {CL}, \end{aligned}$$

where $\mathrm {CL}$ is the confidence level, e.g., $\mathrm {CL}=0.95$. The ordering principle proposed by Feldman and Cousins (1998) is based on the ranking function

**Fig. 7:**
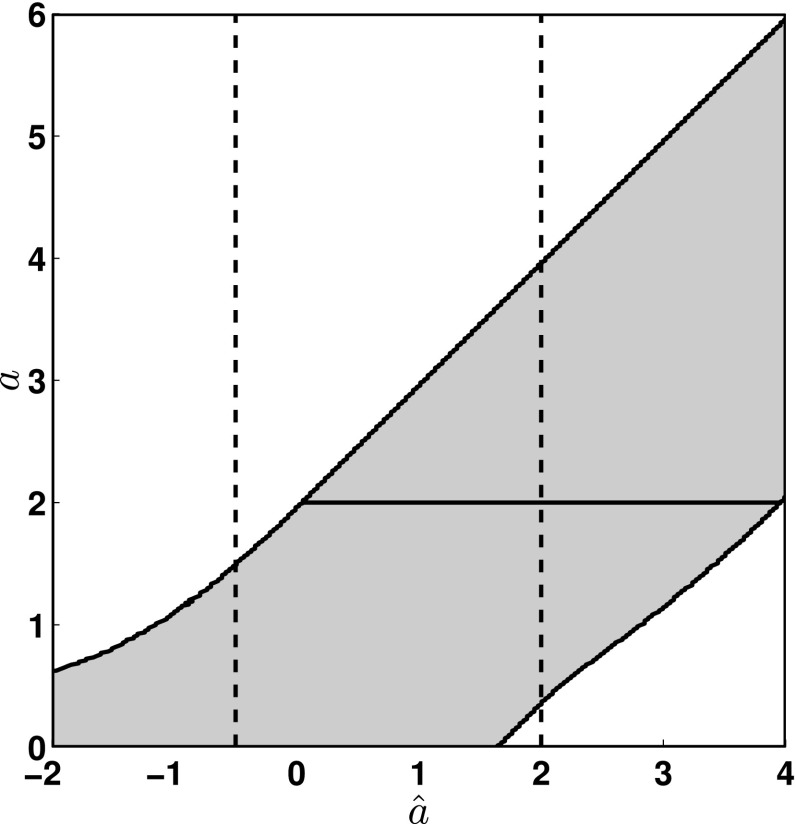
Confidence belt for 95% confidence-level intervals for a Gaussian distribution with mean $a> 0$. (The values for *a* and $\hat{a}$ are given here in units of $\sigma$). The *solid horizontal line* shows the acceptance interval for $a=2.0$. The *two dashed vertical lines* correspond to two different observed values for the estimator $\hat{a}$: $\hat{a}=-0.5$, which has a 95% confidence-level upper limit $a\le 1.5$; and $\hat{a}=2$, which has a 95% confidence-level two-sided interval $a\in [0.35, 3.95]$

3.10 $$\begin{aligned} R(\hat{a}|a) \equiv \frac{p(\hat{a}|a, H_a)}{p(\hat{a}|a, H_a)\big |_{a=a_\mathrm{best}}}, \end{aligned}$$

where $a_\mathrm{best}$ is the value of the parameter *a* that maximizes the sampling distribution $p(\hat{a}|a,H_a)$ for a given value of $\hat{a}$. The prescription then for constructing the acceptance intervals is to find, for each allowed value of *a*, values of $\hat{a}_1$ and $\hat{a}_2$ such that $R(\hat{a}_1|a)=R(\hat{a}_2|a)$ and for which (3.9) is satisfied. The set of all such acceptance intervals for different values of *a* forms a *confidence belt* in the $\hat{a}a$-plane, which is then used to construct an upper limit or a two-sided confidence interval for a particular observed value of the estimator $\hat{a}$, as explained below and illustrated in Fig. 7.

As a specific example, let us suppose that $\hat{a}$ is Gaussian-distributed about *a* with variance $\sigma ^2$:

3.11 $$\begin{aligned} p(\hat{a}|a,H_a) = \frac{1}{\sqrt{2\pi }\sigma } e^{-\frac{1}{2}\frac{(\hat{a}-a)^2}{\sigma ^2}}, \end{aligned}$$

and that the unknown parameter *a* represents the amplitude of a signal, so that $a > 0$. (Recall that it is possible, however, for the estimator $\hat{a}$ to take on negative values). Then $a_\mathrm{best}=\hat{a}$ if $\hat{a} > 0$, while $a_\mathrm{best} = 0$ if $\hat{a} \le 0$, for which

3.12 $$\begin{aligned} p(\hat{a}|a, H_a)\Big |_{a=a_\mathrm{best}} = \left\{ \begin{array}{l@{\quad }l} \frac{1}{\sqrt{2\pi }\sigma }, &{} \hat{a}> 0\\ \frac{1}{\sqrt{2\pi }\sigma } \exp \left[ -\frac{1}{2}\frac{\hat{a}^2}{\sigma ^2}\right] , &{} \hat{a} \le 0 \end{array} \right. \end{aligned}$$

and

3.13 $$\begin{aligned} R(\hat{a}|a) = \left\{ \begin{array}{l@{\quad }l} \exp \left[ -\frac{1}{2}\frac{(\hat{a}-a)^2}{\sigma ^2}\right] , &{} \hat{a}> 0\\ \exp \left[ -\frac{1}{2}\frac{(-2\hat{a} a + a^2)}{\sigma ^2}\right] , &{} \hat{a}\le 0 \end{array} \right. . \end{aligned}$$

The confidence belt constructed from this ranking function is shown in Fig. 7. The solid horizontal line at $a=2$ shows the corresponding 95% confidence-level acceptance interval for this ranking function. The two dashed vertical lines correspond to two different observed values for the estimator $\hat{a}$, leading to a 95% confidence-level upper limit and two-sided interval, respectively.

### Bayesian inference

In the following subsections, we again describe parameter estimation and hypothesis testing, but this time from the perspective of Bayesian inference.

#### Bayesian parameter estimation

In Bayesian inference, a parameter, e.g., *a*, is estimated in terms of its posterior distribution, *p*(*a*|*d*), in light of the observed data *d*. As discussed in the introduction to this section, the posterior *p*(*a*|*d*) can be calculated from the likelihood *p*(*d*|*a*) and the prior probability distribution *p*(*a*) using Bayes’ theorem

3.14 $$\begin{aligned} p(a\vert d) = \frac{p(d\vert a)p(a)}{p(d)}. \end{aligned}$$

The posterior distribution tells you everything you need to know about the parameter, although you might sometimes want to reduce it to a few numbers—e.g., its mode, mean, standard deviation, etc.

**Fig. 8:**
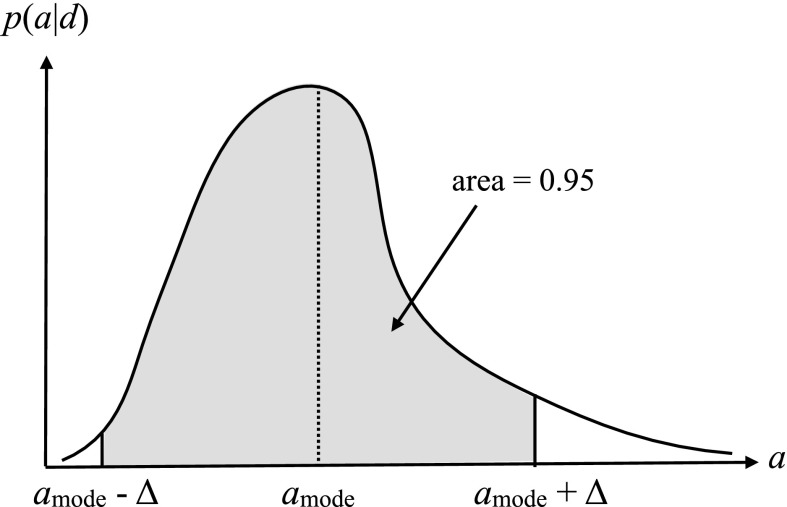
Definition of a Bayesian credible interval for parameter estimation. Here we construct a symmetric 95% credible interval centered on the mode of the distribution

Given a posterior distribution *p*(*a*|*d*), a Bayesian confidence interval (often called a *credible interval* given the Bayesian interpretation of probability as degree of belief, or state of knowledge, about an event) is simply defined in terms of the area under the posterior between one parameter value and another. This is illustrated graphically in Fig. 8, for the case of a 95% symmetric credible interval, centered on the mode of the distribution $a_\mathrm{mode}$. If the posterior distribution depends on two parameters *a* and *b*, but you really only care about *a*, then you can obtain the posterior distribution for *a* by marginalizing the joint distribution *p*(*a*, *b*|*d*) over *b*:

3.15 $$\begin{aligned} p(a\vert d) = \int db\>p(a,b\vert d) = \int db\>p(a\vert b,d) p(b), \end{aligned}$$

where the second equality follows from the relationship between joint probabilities and conditional probabilities, e.g., $p(a|b,d) p(b) = p(a,b|d)$. Variables that you don’t particularly care about (e.g., the variance of the detector noise as opposed to the strength of a gravitational-wave signal) are called *nuisance parameters*. Although nuisance parameters can be handled in a straight-forward manner using Bayesian inference, they are problematic to deal with (i.e., they are a nuisance!) in the context of frequentist statistics. The problem is that marginalization doesn’t make sense to a frequentist, for whom parameters cannot be assigned probability distributions.

The interpretation of Bayes’ theorem (3.14) is that our prior knowledge is updated by what we learn from the data, as measured by the likelihood, to give our posterior state of knowledge. The amount learned from the data is measured by the information gain

3.16 $$\begin{aligned} I = \int da\> p( a \vert d) \log \left( \frac{p( a \vert d)}{p(a)}\right) . \end{aligned}$$

Using a natural logarithm gives the information in *nats*, while using a base 2 logarithm gives the information in *bits*. If the data tells us nothing about the parameter, then $p(d\vert a) = \mathrm{constant}$, which implies $p(a\vert d)=p(a)$ and thus $I=0$.

#### Bayesian upper limits

**Fig. 9:**
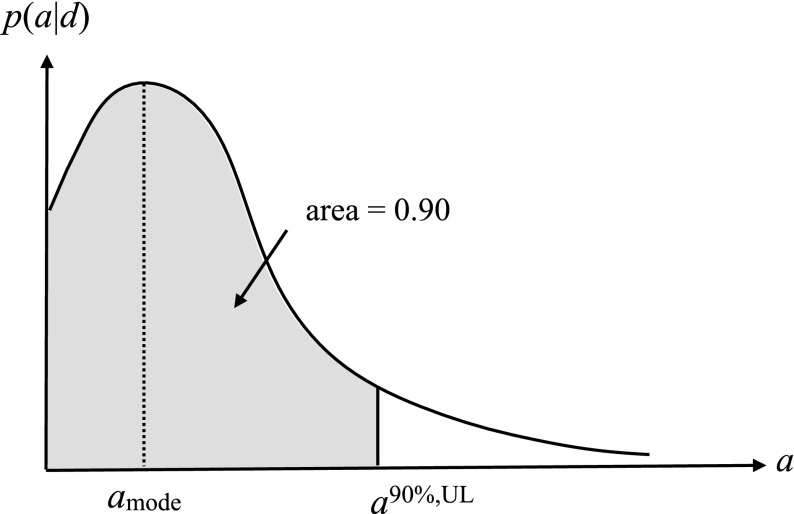
Bayesian 90% credible upper limit for the parameter *a*

A Bayesian upper limit is simply a Bayesian credible interval for a parameter with the lower end point of the interval set to the smallest value that the parameter can take. For example, the Bayesian 90% upper limit on a parameter $a> 0$ is defined by:

3.17 $$\begin{aligned} \mathrm{Prob}(0< a < a^{90\%,\mathrm{UL}}| d) = 0.90, \end{aligned}$$

where probability is interpreted as degree of belief, or state of knowledge, that the parameter *a* has a value in the indicated range. One usually sets an upper limit on a parameter when the mode of the distribution for the parameter being estimated is not sufficiently displaced from zero, as shown in Fig. 9.

#### Bayesian model selection

Bayesian inference can easily be applied to multiple models or hypotheses, each with a different set of parameters. In what follows, we will denote the different models by $\mathcal{M}_\alpha$, where the index $\alpha$ runs over the different models, and the associated set of parameters by the vector $\mathbf {\theta }_\alpha$. The joint posterior distribution for the parameters $\mathbf {\theta }_\alpha$ is given by

3.18 $$\begin{aligned} p( \mathbf {\theta }_\alpha \vert d, \mathcal{M}_\alpha ) = \frac{ p( d \vert \mathbf {\theta }_\alpha , \mathcal{M}_\alpha ) p(\mathbf {\theta }_\alpha \vert \mathcal{M}_\alpha )}{p(d \vert \mathcal{M}_\alpha )} , \end{aligned}$$

and the model evidence is given by

3.19 $$\begin{aligned} p(d \vert \mathcal{M}_\alpha ) = \int p( d\vert \mathbf {\theta }_\alpha , \mathcal{M}_\alpha ) p(\mathbf {\theta }_\alpha \vert \mathcal{M}_\alpha ) \, d\mathbf {\theta }_\alpha , \end{aligned}$$

where we marginalize over the parameter values associated with that model. The posterior probability for model $\mathcal{M}_\alpha$ is given by Bayes’ theorem as

3.20 $$\begin{aligned} p(\mathcal{M}_\alpha \vert d ) = \frac{p(d \vert \mathcal{M}_\alpha ) p(\mathcal{M}_\alpha )}{p(d)}, \end{aligned}$$

where the normalization constant *p*(*d*) involves a sum over all possible models:

3.21 $$\begin{aligned} p(d ) = \sum _\alpha p(d \vert \mathcal{M}_\alpha ) p(\mathcal{M}_\alpha ). \end{aligned}$$

Since the space of all possible models is generally unknown, the sum is usually taken over the subset of models being considered. The normalization can be avoided by considering the posterior odds ratio between two models:

3.22 $$\begin{aligned} \mathcal{O}_{\alpha \beta }(d) = \frac{p(\mathcal{M}_\alpha \vert d )}{p(\mathcal{M}_\beta \vert d )} = \frac{p(\mathcal{M}_\alpha )}{p(\mathcal{M}_\beta )}\, \frac{ p(d \vert \mathcal{M}_\alpha )}{p(d \vert \mathcal{M}_\beta )}. \end{aligned}$$

The first ratio on the right-hand side of the above equation is the *prior* odds ratio for models $\alpha ,\beta$, while the second term is the evidence ratio, or *Bayes factor*,

3.23 $$\begin{aligned} \mathcal{B}_{\alpha \beta }(d) \equiv \frac{p(d \vert \mathcal{M}_\alpha )}{p(d \vert \mathcal{M}_\beta )} . \end{aligned}$$

The prior odds ratio is often taken to equal unity, but this is not always justified. For example, the prior odds that a signal is described by general relativity versus some alternative theory of gravity should be much larger than unity given the firm theoretical and observational footing of Einstein’s theory.

While the foundations of Bayesian inference were laid out by Laplace in the 1700s, it did not see widespread use until the late twentieth century with the advent of practical implementation schemes and the development of fast electronic computers. Today, Monte Carlo sampling techniques, such as Markov Chain Monte Carlo (MCMC) and Nested Sampling, are used to sample the posterior and estimate the evidence (Skilling 2006; Gair et al. 2010). Successfully applying these techniques is something of an art, but in principle, once the likelihood and prior have been written down, the implementation of Bayesian inference is purely mechanical. Calculating the likelihood and choosing a prior will be discussed in some detail in Sect. 3.6.

### Relating Bayesian and frequentist detection statements

It is interesting to compare the Bayesian model selection calculation discussed above to frequentist hypothesis testing based on the *maximum-likelihood ratio*. For concreteness, let us assume that we have two models $\mathcal{M}_0$ (noise-only) and $\mathcal{M}_1$ (noise plus gravitational-wave signal), with parameters $\mathbf {\theta }_n$ and $\{\mathbf {\theta }_n,\mathbf {\theta }_h\}$, respectively. The frequentist detection statistic will be defined in terms of the ratio of the maxima of the likelihood functions for the two models:

3.24 $$\begin{aligned} \Lambda _\mathrm{ML}(d) \equiv \frac{\max _{\mathbf {\theta }_n} \max _{\mathbf {\theta }_h} p(d\vert \mathbf {\theta }_n,\mathbf {\theta }_h,\mathcal{M}_1)}{\max _{\mathbf {\theta }_n'} p(d\vert \mathbf {\theta }_n',\mathcal{M}_0)}. \end{aligned}$$

As described above, the Bayes factor calculation also involves a ratio of two quantities, the model evidences $p(d\vert \mathcal{M}_1)$ and $p(d\vert \mathcal{M}_0)$, but instead of maximizing over the parameters, we marginalize over the parameters:

3.25 $$\begin{aligned} \mathcal{B}_{10}(d) =\frac{\int d\mathbf {\theta }_n\int d\mathbf {\theta }_h\> p(d\vert \mathbf {\theta }_n,\mathbf {\theta }_h,\mathcal{M}_1) p(\mathbf {\theta }_n,\mathbf {\theta }_h\vert \mathcal{M}_1)}{\int d\mathbf {\theta }_n'\> p(d\vert \mathbf {\theta }_n',\mathcal{M}_0)p(\mathbf {\theta }_n'\vert \mathcal{M}_0)}. \end{aligned}$$

These two expressions can be related using Laplace’s approximation to individually approximate the model evidences $p(d\vert \mathcal{M}_1)$ and $p(d\vert \mathcal{M}_0)$. This approximation is valid when the data are *informative*—i.e., when the likelihood functions are peaked relative to the joint prior probability distributions of the parameters. For an arbitrary model $\mathcal{M}$ with parameters $\mathbf {\theta }$, the Laplace approximation yields:

3.26 $$\begin{aligned} \int d\mathbf {\theta }\> p(d|\mathbf {\theta }, \mathcal{M})p(\mathbf {\theta }|\mathcal{M}) \simeq p(d|\mathbf {\theta }_\mathrm{ML},\mathcal{M}) \frac{\Delta V_\mathcal{M}}{V_\mathcal{M}}, \end{aligned}$$

where $\mathbf {\theta }_\mathrm{ML}\equiv \mathbf {\theta }_\mathrm{ML}(d)$ maximizes the likelihood with respect to variations of $\mathbf {\theta }$ given the data *d*; $\Delta V_\mathcal{M}$ is the characteristic spread of the likelihood function around its maximum (the volume of the uncertainty ellipsoid for the parameters); and $V_\mathcal{M}$ is the total parameter space volume of the model parameters. Applying this approximation to models $\mathcal{M}_0$ and $\mathcal{M}_1$ in (3.25), we obtain

3.27 $$\begin{aligned} \mathcal{B}_{10}(d) \simeq \Lambda _\mathrm{ML}(d)\frac{\Delta V_1/V_1}{\Delta V_0/V_0}, \end{aligned}$$

or, equivalently,

3.28 $$\begin{aligned} 2\ln \mathcal{B}_{10}(d) \simeq 2\ln \left( \Lambda _\mathrm{ML}(d)\right) + 2\ln \left( \frac{\Delta V_1/V_1}{\Delta V_0/V_0}\right) . \end{aligned}$$

The second term on the right-hand side of the above equation is negative and penalizes models that require a larger parameter space volume than necessary to fit the data. This is basically an *Occam penalty factor*, which prefers the simpler of two models that fit the data equally well. The first term has the interpretation of being the squared signal-to-noise ratio of the data, assuming an additive signal in Gaussian-stationary noise, and it can be used as an alternative frequentist detection statistic in place of $\Lambda _\mathrm{ML}$.

Table 3 from Kass and Raftery (1995) gives a range of Bayes factors and their interpretation in terms of the strength of the evidence in favor of one model relative to another. The precise levels at which one considers the evidence to be “strong” or “very strong” is rather subjective. But recent studies (Cornish and Sampson 2016; Taylor et al. 2016a) in the context of pulsar timing have been trying to make this correspondence a bit firmer, using *sky* and *phase scrambles* to effectively destroy signal-induced spatial correlations between pulsars while retaining the statistical properties of each individual dataset. This is similar to doing time-slides for LIGO analyses, which are used to assess the significance of a detection.

**Table 3:** Bayes factors and their interpretation in terms of the strength of the evidence in favor of one model relative to the other

$\mathcal{B}_{\alpha \beta }(d)$	$2\ln \mathcal{B}_{\alpha \beta }(d)$	Evidence for model $\mathcal{M}_\alpha$ relative to $\mathcal{M}_\beta$
${<}1$	${<}0$	Negative (supports model $\mathcal{M}_\beta$)
1–3	0–2	Not worth more than a bare mention
3–20	2–6	Positive
20–150	6–10	Strong
${>}150$	${>}10$	Very strong

Adapted from Kass and Raftery (1995)

Taylor et al. (2016a) even go so far as to perform a *hybrid* frequentist-Bayesian analysis, doing Monte Carlo simulations: (i) over different noise-only realizations, and (ii) over different sky and phase scrambles, which null the correlated signal. These simulations produce different null *distributions* for the Bayes factor, similar to a null-hypothesis distribution for a frequentist detection statistic (in this case, the log of the Bayes factor). The significance of the measured Bayes factor is then its corresponding *p*-value with respect to one of these null distributions. The utility of such a hybrid analysis is its ability to better assess the significance of a detection claim, especially when there might be questions about the suitability of one of the models (e.g., the noise model) used in the construction of a likelihood function.

### Simple example comparing Bayesian and frequentist analyses

To further illustrate the relationship between Bayesian and frequentist analyses, we consider in this section a very simple example—a constant signal with amplitude $a>0$ in white, Gaussian noise (zero mean, variance $\sigma$):

3.29 $$\begin{aligned} d_i = a + n_i, \quad i=1,2,\ldots , N, \end{aligned}$$

where the index *i* labels the individual samples of the data. The likelihood functions for the noise-only and signal-plus-noise models $\mathcal{M}_0$ and $\mathcal{M}_1$ are thus simple Gaussians:

3.30 $$\begin{aligned} p(d|\mathcal{M}_0)&= \frac{1}{(2\pi )^{N/2}\sigma ^N} e^{-\frac{1}{2\sigma ^2}\sum _{i=1}^N d_i^2}, \end{aligned}$$

3.31 $$\begin{aligned} p(d|a, \mathcal{M}_1)&= \frac{1}{(2\pi )^{N/2}\sigma ^N} e^{-\frac{1}{2\sigma ^2}\sum _{i=1}^N (d_i - a)^2}. \end{aligned}$$

We will assume that the value of $\sigma$ is known a priori. Thus, the noise model has no free parameters, while the signal model has just one parameter, which is the amplitude of the signal that we are trying to detect. We will choose our prior on *a* to be flat over the interval $(0,a_\mathrm{max}]$, so $p(a)=1/a_\mathrm{max}$.

It is straight-forward exercise to check that the maximum-likelihood estimator of the amplitude *a* is given by the sample mean of the data:

3.32 $$\begin{aligned} \hat{a} \equiv a_\mathrm{ML}(d) = \frac{1}{N}\sum _{i=1}^N d_i \equiv \bar{d}. \end{aligned}$$

This is an unbiased estimator of *a* and has variance $\sigma _{\hat{a}}^2 =\sigma ^2/N$ (the familiar variance of the sample mean). Thus, the sampling distribution of $\hat{a}$ is simply

3.33 $$\begin{aligned} p(\hat{a}|a, \mathcal{M}_1) = \frac{1}{\sqrt{2\pi }\sigma _{\hat{a}}} e^{-\frac{1}{2\sigma _{\hat{a}}^2}(\hat{a}-a)^2}, \end{aligned}$$

where $\hat{a}$ can take on either positive or negative values (even though $a>0$).

To compute the posterior distribution $p(a|d,\mathcal{M}_1)$ for the Bayesian analysis, we first note that

3.34 $$\begin{aligned} \sum _{i=1}^N (d_i - a)^2 = N( \mathrm{Var}[d] + (a-\hat{a})^2 ). \end{aligned}$$

The model evidence $p(d | \mathcal{M}_1)$ is then given by

3.35 $$\begin{aligned} p(d| \mathcal{M}_1) =\frac{e^{- \frac{\mathrm{Var}[d]}{2 \sigma _{\hat{a}}^2 }} \left[ \mathrm{erf}\left( \frac{a_\mathrm{max}-\hat{a}}{\sqrt{2} \sigma _{\hat{a}}}\right) + \mathrm{erf}\left( \frac{\hat{a}}{\sqrt{2} \sigma _{\hat{a}}}\right) \right] }{2 a_\mathrm{max} \sqrt{N} (2\pi )^{(N-1)/2} \sigma ^{(N-1)}}, \end{aligned}$$

and the posterior distribution is given by

3.36 $$\begin{aligned} p(a|d,\mathcal{M}_1) = \frac{1}{\sqrt{2\pi } \sigma _{\hat{a}}} e^{-\frac{(a-\hat{a})^2}{2\sigma _{\hat{a}}^2}} 2\left[ \mathrm{erf}\left( \frac{a_\mathrm{max}-\hat{a}}{\sqrt{2} \sigma _{\hat{a}}}\right) + \mathrm{erf}\left( \frac{\hat{a}}{\sqrt{2} \sigma _{\hat{a}}}\right) \right] ^{-1}. \end{aligned}$$

Note that this is simply a *truncated* Gaussian on the interval $a\in (0,a_\mathrm{max}]$, with mean $\hat{a}$ and variance $\sigma _{\hat{a}}^2$.

The above calculation shows that $\hat{a}$ is a *sufficient statistic* for *a*. This means that the posterior distribution for *a* can be written simply in terms of $\hat{a}$, in lieu of the individual samples $d\equiv \{d_1, d_2, \ldots , d_N\}$. The Bayes factor

3.37 $$\begin{aligned} \mathcal{B}_{10}(d) = \frac{p(d| \mathcal{M}_1)}{p(d| \mathcal{M}_0)}, \end{aligned}$$

is given by

3.38 $$\begin{aligned} \mathcal{B}_{10}(d) = e^{\frac{ \hat{a}^2}{2 \sigma _{\hat{a}}^2}} \left( \frac{\sqrt{2\pi } \sigma _{\hat{a}}}{a_\mathrm{max}}\right) \frac{1}{2} \left[ \mathrm{erf}\left( \frac{a_\mathrm{max}-\hat{a}}{\sqrt{2} \sigma _{\hat{a}}}\right) + \mathrm{erf}\left( \frac{\hat{a}}{\sqrt{2} \sigma _{\hat{a}}}\right) \right] . \end{aligned}$$

In the limit where $\hat{a}$ is tightly peaked away from 0 and $a_\mathrm{max}$, the Bayes factor simplifies to

3.39 $$\begin{aligned} \mathcal{B}_{10}(d) \simeq e^{\frac{ \hat{a}^2}{2 \sigma _{\hat{a}}^2} } \left( \frac{ \sqrt{2\pi } \sigma _{\hat{a}} }{ a_\mathrm{max} } \right) . \end{aligned}$$

If we take the frequentist detection statistic to be twice the log of the maximum-likelihood ratio, $\Lambda (d) \equiv 2\ln \Lambda _\mathrm{ML}(d)$, then

3.40 $$\begin{aligned} \Lambda (d) = \frac{ \hat{a}^2}{\sigma _{\hat{a}}^2} = \frac{\bar{d}^2}{\sigma ^2/N} \equiv \rho ^2, \end{aligned}$$

which is just the squared signal-to-noise ratio of the data. Furthermore, taking twice the log of the approximate Bayes factor in (3.39) gives

3.41 $$\begin{aligned} 2\ln \mathcal{B}_{10}(d) \simeq \Lambda (d) + 2\ln \left( \frac{ \sqrt{2\pi } \sigma _{\hat{a}} }{ a_\mathrm{max} } \right) , \end{aligned}$$

where the first term is just the frequentist detection statistic and second term expresses the Occam penalty. This last result is consistent with the general relation (3.28) discussed in the previous subsection.

The statistical distribution of the frequentist detection statistic can be found in closed form for this simple example. Since a linear combination of Gaussian random variables is also Gaussian-distributed, $\Lambda$ is the *square* of a (single) Gaussian random variable $\rho =\bar{d}\sqrt{N}/\sigma$. Moreover, since $\rho$ has mean $\mu \equiv a\sqrt{N}/\sigma$ and unit variance, the sampling distribution for $\Lambda$ in the presence of a signal is a *noncentral chi-squared* distribution with one degree of freedom and non-centrality parameter $\lambda \equiv \mu ^2 = a^2 N/\sigma ^2$:

3.42 $$\begin{aligned} p(\Lambda |a, \mathcal{M}_1) =\frac{1}{2} e^{-(\Lambda +\lambda )/2}\left( \frac{\Lambda }{\lambda }\right) ^{-1/4} I_{-1/2}(\sqrt{\lambda \Lambda }), \end{aligned}$$

where $I_{-1/2}$ is a modified Bessel function of the first kind of order $-1/2$. In the absence of a signal (i.e., when *a* and hence $\lambda$ are equal to zero), $\Lambda$ is given by an (ordinary) chi-squared distribution with one degree of freedom:

3.43 $$\begin{aligned} p(\Lambda |\mathcal{M}_0) =\frac{1}{\sqrt{2}\Gamma (1/2)}\Lambda ^{-1/2}e^{-\Lambda /2}, \end{aligned}$$

where $\Gamma$ is the gamma function. Substituting explicit expressions for $I_{-1/2}(\sqrt{\lambda \Lambda })$ and $\Gamma (1/2)$, we find:

3.44 $$\begin{aligned} p(\Lambda |\mathcal{M}_0)&=\frac{1}{\sqrt{2\pi \Lambda }}e^{-\Lambda /2}, \end{aligned}$$

3.45 $$\begin{aligned} p(\Lambda |a, \mathcal{M}_1)&=\frac{1}{\sqrt{2\pi \Lambda }} \frac{1}{2}\left[ e^{-\frac{1}{2}(\sqrt{\Lambda }-\sqrt{\lambda })^2} +e^{-\frac{1}{2}(\sqrt{\Lambda }+\sqrt{\lambda })^2} \right] . \end{aligned}$$

**Fig. 10:**
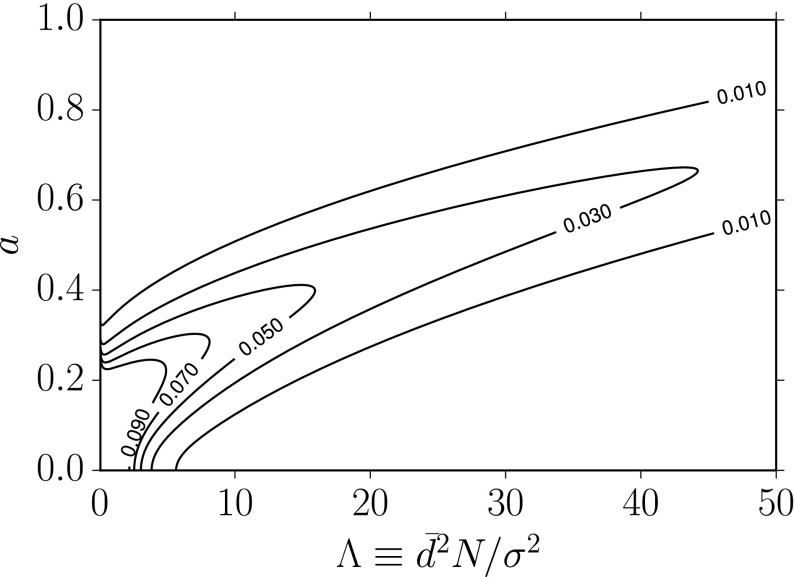
Equal-probability contour plot for the frequentist detection statistic $\Lambda \equiv \bar{d}^2 N/\sigma ^2$ for a signal with amplitude $a>0$. The *contours* correspond to the values $p(\Lambda |a, \mathcal{M}_1)=0.01$, 0.03, 0.05, 0.07, and 0.09

An equal-probability contour plot of the sampling distribution of the detection statistic is shown in Fig. 10. The fact that we are able to write down *analytic* expressions for the sampling distributions for the detection statistic $\Lambda$ is due to the simplicity of the signal and noise models. For more complicated real-world problems, these distributions would need to be generated *numerically* using fake signal injections and time-shifts to produce many different realizations of the data (signal plus noise) from which one can build up the distributions.

It is also important to point out that $\Lambda$ is *not* a sufficient statistic for *a*, due to the fact that $\Lambda$ involves the *square* of the maximum-likelihood estimate $\hat{a}$—i.e., $\Lambda = \hat{a}^2 N/\sigma ^2$. Thus, we cannot take $p(\Lambda |a,\mathcal{M}_1)$ conditioned on $\Lambda$ (assuming a flat prior on *a* from $[0,a_\mathrm{max}]$) to get the posterior distribution for *a* given *d*, since we would be missing out on data samples that give negative values for $\hat{a}$. Another way to see this is to start with $p(\Lambda |a,\mathcal{M}_1)$ given by (3.45), and then make a change of variables from $\Lambda$ to $\hat{a}$ using the general transformation relation

3.46 $$\begin{aligned} p_Y(y)\, dy = p_X(x)\, dx \quad \Rightarrow \quad p_X(x) = \left[ p_Y(y)\, |f'(x)|\right] _{y= f(x)}. \end{aligned}$$

This leads to

3.47 $$\begin{aligned} \tilde{p}(\hat{a}|a, \mathcal{M}_1) = \frac{1}{\sqrt{2\pi }\sigma _{\hat{a}}} \left[ e^{-\frac{1}{2\sigma _{\hat{a}}^2}(\hat{a}- a)^2} +e^{-\frac{1}{2\sigma _{\hat{a}}^2}(\hat{a} + a)^2} \right] , \end{aligned}$$

which is properly normalized for $\hat{a}>0$, but differs from (3.33) due to the second term involving $\hat{a}+a$. Thus, we need to construct *p*(*a*|*d*) from (3.33)—and *not* from (3.47)—if we want the posterior to have the proper dependence on *a*.

#### Simulated data

**Fig. 11:**
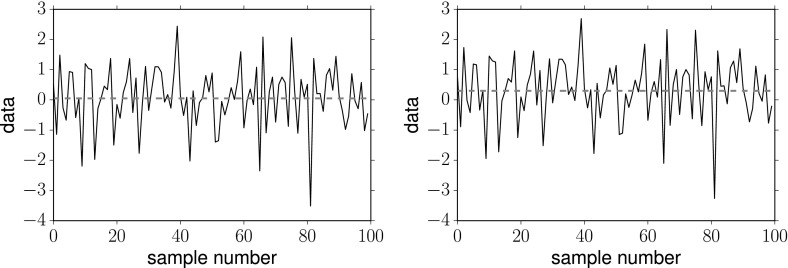
Examples of simulated data for weak (*left panel*) and strong (*right panel*) signals injected into the data—$a_0=0.05$ and 0.3, respectively

For our example, we will take $N=100$ samples, $\sigma =1$, and $a_\mathrm{max} =1.0$. We also simulate data with injected signals having amplitudes $a_0=0.05$ and 0.3, respectively. Since the expected signal-to-noise ratio, $a\sqrt{N}/\sigma$, is given by 0.5 and 3.0, these injections correspond to *weak* and (moderately) *strong* signals. Single realizations of the data for the two different injections are shown in Fig. 11. The noise realization is the same for the two injections.

#### Frequentist analysis

**Fig. 12:**
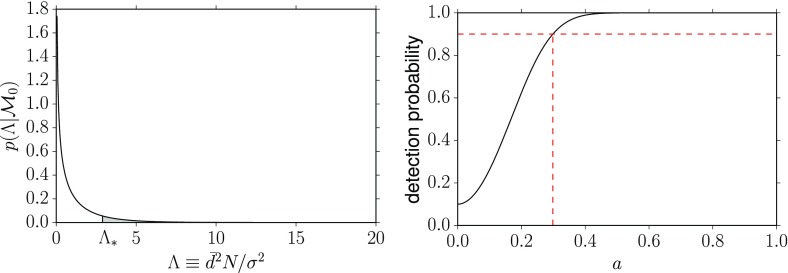
*Left panel* Probability distribution for the frequentist detection statistic $\Lambda$ for the noise-only model. The threshold value of the statistic for 10% false alarm probability is $\Lambda _*=2.9$. *Right panel* Detection probability as a function of the amplitude *a*. The value of the amplitude needed for 90% confidence detection probability with $10\%$ false alarm probability is $a^{90\%,\mathrm{DP}} = 0.30$

Given the values for *N*, $\sigma$, and the probability distributions (3.44) and (3.45) for the frequentist detection statistic $\Lambda$, we can calculate the detection threshold for fixed false alarm probability $\alpha$ (which we will take to equal 10%), and the corresponding detection probability as a function of the amplitude *a*. The detection threshold turns out to equal $\Lambda _* = 2.9$ (so 10% of the area under the probability distribution $p(\Lambda |\mathcal{M}_0)$ is for $\Lambda \ge \Lambda _*$). The value of the amplitude *a* needed for 90% confidence detection probability with 10% false alarm probability is given by $a^{90\%,\mathrm{DP}}=0.30$. (These results for the detection threshold and detection probability do *not* depend on the particular realizations of the simulated data). The corresponding curves are shown in Fig. 12.

**Fig. 13:**
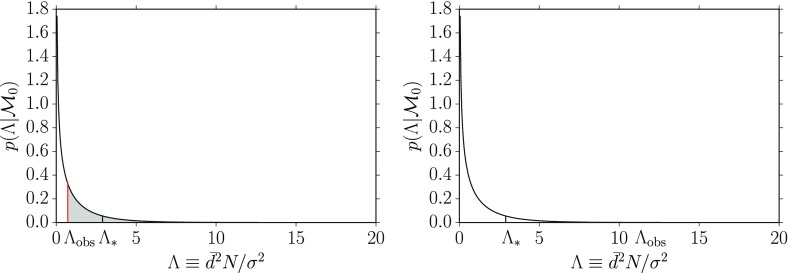
Graphical representation of the *p*-value calculation for the weak (*left panel*) and strong (*right panel*) injections. For the weak injection, $\Lambda _\mathrm{obs}=0.72$ is marked by the *red vertical line*, with corresponding *p*-value 0.45. For the strong injection, $\Lambda _\mathrm{obs}=11.2$ is sufficiently large that the corresponding *red vertical line* is not visible on this graph. The *p*-value for the strong injection is $9.0\times 10^{-4}$

The sample mean of the data for the two simulations are given by $\bar{d} = 0.085$ and 0.335, respectively. Since $\hat{a} = \bar{d}$, these are also the values of the maximum-likelihood estimator of the amplitude *a*. The corresponding values of the detection statistic are $\Lambda _\mathrm{obs} = 0.72$ and 11.2 for the two injections, and have *p*-values equal to 0.45 and $9.0\times 10^{-4}$, as shown in Fig. 13. The 95% frequentist confidence interval is given simply by $[\hat{a}-2\sigma _{\hat{a}},\hat{a}+2\sigma _{\hat{a}}]$, since $\hat{a}$ is Gaussian-distributed, and has values $[-0.11,0.29]$ and [0.14, 0.54], respectively. These intervals contain the true value of the amplitudes for the two injections, $a_0=0.05$ and 0.3.

**Fig. 14:**
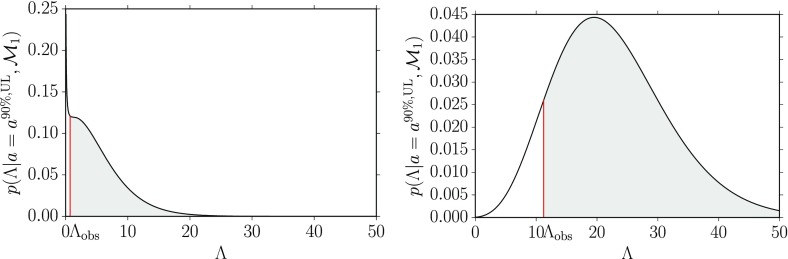
Probability distributions for the frequentist detection statistic $\Lambda$, conditioned on the value of the amplitude *a* for which the probability of obtaining $\Lambda \ge \Lambda _\mathrm{obs}$ is equal to 0.90. These define the 90% confidence-level frequentist upper limits $a^{90\%,\mathrm{UL}}= 0.20$ and 0.46, respectively. The *red vertical lines* mark the value of $\Lambda _\mathrm{obs}$ for the weak (*left panel*, $\Lambda _\mathrm{obs}=0.72$) and strong (*right panel*, $\Lambda _\mathrm{obs}=11.2$) injections

The 90% confidence-level frequentist upper limits are $a^{90\%,\mathrm{UL}}= 0.20$ and 0.46, respectively. Figure 14 shows the probability distributions for the detection statistic $\Lambda$ conditioned on these upper limit values for which the probability of obtaining $\Lambda \ge \Lambda _\mathrm{obs}$ is equal to 0.90.

#### Bayesian analysis

**Fig. 15:**
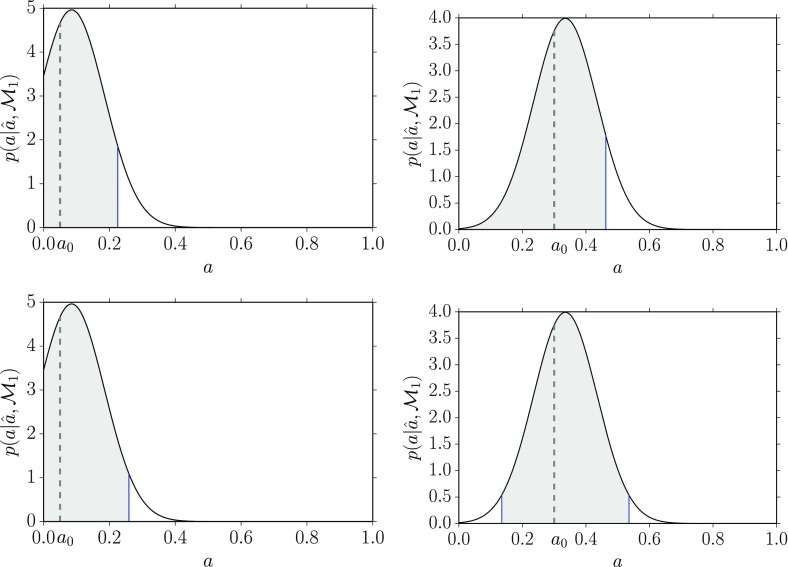
Posterior distributions for the amplitude *a* given the value of the maximum-likelihood estimator $\hat{a}$. The *left two panels* are for the weak injection; the *right two panels* are for the strong injection. The *top two plots* illustrate the graphical construction of Bayesian 90% upper limits for the two injections; the *bottom two plots* illustrate the graphical construction of the Bayesian 95% credible intervals. The *dashed vertical lines* indicate the values of the injected signal amplitude $a_0$, which equal 0.05 and 0.3, respectively

The results of the Bayesian analysis for the two different injections are summarized in Fig. 15. The plots show the posterior distribution for the amplitude *a* given the value of the maximum-likelihood estimator $\hat{a}$, which (as we discussed earlier) is a sufficient statistic for the data *d*. Recall that the posterior for *a* for this example is simply a truncated Gaussian from 0 to $a_\mathrm{max}$ centered on $\hat{a}$, which could be negative, see (3.36). The left two panels show the graphical construction of the Bayesian 90% upper limit and 95% credible interval for the amplitude *a* for the weak injection, $a^{90\%,\mathrm{UL}}=0.23$ and [0, 0.26]. The right two panels show similar plots for the strong injection, $a^{90\%,\mathrm{UL}}=0.46$ and [0.14, 0.54].

Finally, the Bayes factor for the signal-plus-noise model $\mathcal{M}_1$ relative to the noise-only model $\mathcal{M}_0$ can be calculated by taking the ratio of the marginalized likelihood $p(d|\mathcal{M}_1)$ given by (3.35) to $p(d|\mathcal{M}_0)$ given by (3.30). Doing this, we find 2 ln $B_{10} = -2.2$ and 9.2 for the weak and strong signal injections, respectively. The Laplace approximation to this quantity is given by (3.41), with values $-2.0$ and 8.5, respectively.

#### Comparison summary

Table 4 summarizes the numerical results for the frequentist and Bayesian analyses. We see that the frequentist and Bayesian 90% upper limits and 95% intervals numerically agree for the strong injection, but differ slightly for the weak injection. The interpretation of these results is different, of course, for a frequentist and a Bayesian, given their different definitions of probability. But for a moderately strong signal in noisy data, we expect both approaches to yield a confident detection as they have for this simple example.

**Table 4:** Tabular summary of the frequentist and Bayesian analysis results for the simulated data (both weak and strong injections)

	(Weak injection, $a_0=-0.05$)		(Strong injection, $a_0=0.3$)	
	Frequentist	Bayesian	Frequentist	Bayesian
Detection threshold ($\Lambda _*$)	2.9	–	2.9	–
Detection statistic ($\Lambda _\mathrm{obs}$)	0.72	–	11.2	–
*p*-value	0.45	–	$9.0\times 10^{-4}$	–
90% upper limit	0.20	0.23	0.46	0.46
95% interval	$[-0.11,0.29]$	[0, 0.26]	[0.14, 0.54]	[0.14, 0.54]
ML estimator ($\hat{a}$)	0.085	0.085	0.335	0.335
Bayes factor ($2\ln \mathcal{B}_{10}$)	–	$-2.2$	–	9.2
Laplace approximation	–	$-2.0$	–	8.5

A dash indicates that a particular quantity is not relevant for either the frequentist or Bayesian analysis

### Likelihoods and priors for gravitational-wave searches

To conclude this section on statistical inference, we discuss some issues related to calculating the likelihood and choosing a prior in the context of searches for gravitational-wave signals using a network of gravitational-wave detectors.

#### Calculating the likelihood

Defining the likelihood function (for either a frequentist or Bayesian analysis) involves understanding the instrument response and the instrument noise. The data collected by gravitational-wave detectors comes in a variety of forms. For ground-based interferometers such as LIGO and Virgo, the data comes from the error signal in the differential arm-length control system, which is non-linearly related to the laser phase difference, which in turn is linearly related to the gravitational-wave strain. For pulsar timing arrays, the data comes from the arrival times of radio pulses (derived from the folded pulse profiles), which must be corrected using a complicated timing model that takes into account the relative motion of the telescopes and the pulsars, along with the spin-down of the pulsars, in addition to a variety of propagation effects. The timing residuals formed by subtracting the timing model from the raw arrival times contain perturbations due to gravitational waves integrated along the line of sight to the pulsar. For future space-based gravitational-wave detectors such as LISA, the data will be directly read out from phase meters that perform a heterodyne measurement of the laser phase. Synthetic combinations of these phase read outs (chosen to cancel laser phase noise) are then linearly proportional to the gravitational-wave strain.

Since gravitational waves can be treated as small perturbations to the background geometry, the time delays or laser phase/frequency shifts caused by a gravitational wave can easily be computed. These idealized calculations have then to be related to the actual observations, either by propagating the effects through an instrument response model, or, alternatively, inverting the response model to convert the measured data to something proportional to the gravitational-wave strain. (For example, most LIGO analyses work with the calibrated strain, rather than the raw differential error signal). If we assume that the gravitational-wave signal and the instrument noise are linearly independent, then the data taken at time *t* can be written as

3.48 $$\begin{aligned} d(t) = h(t) + n(t), \end{aligned}$$

where *h*(*t*) is shorthand for the gravitational-wave metric perturbations $h_{ab}(t,{\vec {x}})$ convolved with the instrument response function and converted into the appropriate quantity—phase shift, time delay, differential arm length error, etc. (A detailed calculation of *h*(*t*) and the associated detector response functions will be given in Sect. 5.2). As mentioned above, the data *d*(*t*) may be the quantity that is measured directly, or, more commonly, some quantity that is derived from the measurements such as timing residuals or calibrated strain. In any analysis, it is important to marginalize over the model parameters used to make the conversion from the raw data.

The likelihood of observing *d*(*t*) is found by demanding that the residual

3.49 $$\begin{aligned} r(t) \equiv d(t) - \bar{h}(t), \end{aligned}$$

be consistent with a draw from the noise distribution $p_n(x)$:

3.50 $$\begin{aligned} p( d(t) \vert \bar{h}(t)) = p_n(r(t))=p_n(d(t) - \bar{h}(t) ). \end{aligned}$$

Here $\bar{h}(t)$ is our model^6^ for the gravitational-wave signal. The likelihood of observing a collection of discretely-sampled data $d \equiv \{ d_1, d_2, \ldots , d_N\}$, where $d_i\equiv d(t_i)$, is then given by $p( d\vert \bar{h}) = p_n(r)$, where $r\equiv \{r_1, r_2,\ldots , r_N\}$ with $r_i\equiv r(t_i)$. Since instrument noise is due to a large number of small disturbances combined with counting noise in the large-number limit, the central limit theorem suggests that the noise distribution can be approximated by a multi-variate normal (Gaussian) distribution:

3.51 $$\begin{aligned} p( d\vert \bar{h}) = \frac{1}{\sqrt{\mathrm{det} (2\pi C_n)}}\, e^{-\frac{1}{2}\sum _{i,j} r_i \left( C_n^{-1}\right) _{ij} r_j} , \end{aligned}$$

where $C_n$ is the noise correlation matrix, with components

3.52 $$\begin{aligned} (C_n)_{ij} = \langle n_i n_j\rangle -\langle n_i\rangle \langle n_j\rangle . \end{aligned}$$

If the noise is stationary, then the correlation matrix only depends on the lag $\vert t_i - t_j\vert$, and the matrix $C_n$ can be (approximately) diagonalized by transforming to the Fourier domain, where $r_i$ should then be interpreted as $\tilde{r}(f_i)$ (see Appendix D.6 for a more careful treatment of discrete probability distributions in the time and frequency domain). In practice, the noise observed in most gravitational-wave experiments is neither stationary nor Gaussian (Sect. 9 and Appendix C), but (3.51) still serves as a good starting point for more sophisticated treatments. The Gaussian likelihood (3.51) immediately generalizes for a network of detectors:

3.53 $$\begin{aligned} p( d\vert \bar{h}) = \frac{1}{\sqrt{\mathrm{det} (2\pi C_n)}} e^{-\frac{1}{2} \sum _{Ii,Jj}r_{Ii} \left( C_n^{-1}\right) _{Ii,Jj} r_{Jj}} , \end{aligned}$$

where *I*, *J* labels the detector, and *i*, *j* labels the discrete time or frequency sample for the corresponding detector. Note here that the parameters $\mathbf {\theta }$ appearing in (3.18) are the individual time or frequency samples $\bar{h}_i$.

#### Choosing a prior

For Bayesian inference, it is also necessary to define a model $\mathcal{M}$ for the gravitational-wave signal, which is done by placing a prior $p(\bar{h} \vert \mathcal{M})$ on the samples $\bar{h}_i$. In some cases, a great deal is known about the signal model, such as when approximate solutions to Einstein’s equations provide waveform templates. In that case the prior can be written as

3.54 $$\begin{aligned} p(\bar{h} \vert \mathcal{M}) = \delta (\bar{h} - \bar{h}(\mathbf {\theta }, \mathcal{M})) \, p(\mathbf {\theta } \vert \mathcal{M}) . \end{aligned}$$

Marginalizing over $\bar{h}$ converts the posterior $p(\bar{h} \vert d)$ to a posterior distribution for the signal parameters $p(\mathbf {\theta }\vert d, \mathcal{M})$. In other cases, such as for short-duration bursts associated with certain violent astrophysical events, much less is known about the possible signals and weaker priors have to be used. Models using wavelets, which have finite time-frequency support, and priors that favor connected concentrations of power in the time-frequency plane are commonly used for these “unmodeled burst” searches. At the other end of the spectrum from deterministic point sources are the statistically-isotropic stochastic backgrounds that are thought to be generated by various processes in the early Universe, or through the superposition of a vast number of weak astrophysical sources. In the case of Gaussian stochastic signals, the prior for a signal $\bar{h}=(\bar{h}{}_+(\hat{n}),\bar{h}{}_\times (\hat{n}))$ coming from direction $\hat{n}$ direction has the form

3.55 $$\begin{aligned} p(\bar{h}\vert \mathcal{M}) = \frac{1}{2\pi S_h} e^{-(\bar{h}{}^2_+(\hat{n})+\bar{h}{}^2_\times (\hat{n}))/2 S_h}, \end{aligned}$$

where $S_h$ is the power spectrum of the background. As we shall show in Sect. 4, marginalizing over $\bar{h}$ converts the posterior $p(\bar{h} \vert d)$ to a posterior $p(S_h \vert d, \mathcal{M})$ for $S_h$.

## Correlations

Correlation is not cause, it is just a ‘music of chance’. *Siri Hustvedt*

Stochastic gravitational waves are indistinguishable from unidentified instrumental noise in a single detector, but are correlated between pairs of detectors in ways that differ, in general, from instrumental noise. Cross-correlation methods basically use the random output of one detector as a template for the other, taking into account the physical separation and relative orientation of the two detectors. In this section, we introduce cross-correlation methods in the context of both frequentist and Bayesian inference, analyzing in detail a simple toy problem (the data are “white” and we ignore complications that come from the separation and relative orientation of the detectors—this we discuss in detail in Sect. 5). We also briefly discuss possible alternatives to cross-correlation methods, e.g., using a null channel as a noise calibrator.

The basic idea of using cross-correlation to search for stochastic gravitational-waves can be found in several early papers (Grishchuk 1976; Hellings and Downs 1983; Michelson 1987; Christensen 1990, 1992; Flanagan 1993). The derivation of the likelihood function in Sect. 4.2 follows that of Cornish and Romano (2013); parts of Sect. 4.4 are also discussed in Allen et al. (2003) and Drasco and Flanagan (2003).

### Basic idea

The key property that allows one to distinguish a stochastic gravitational-wave background from instrumental noise is that the gravitational-wave signal is correlated across multiple detectors while instrumental noise typically is not. To see this, consider the simplest possible example, i.e., a single sample of data from two colocated and coaligned detectors:

4.1 $$\begin{aligned} \begin{aligned} d_1&= h + n_1, \\ d_2&= h + n_2. \end{aligned} \end{aligned}$$

Here *h* denotes the common gravitational-wave signal and $n_1$, $n_2$ the noise in the two detectors. To cross correlate the data, we simply form the product of the two samples, $\hat{C}_{12}\equiv d_1 d_2$. The expected value of the correlation is then4.2since the gravitational-wave signal and the instrumental noise are uncorrelated. If the instrumental noise in the two detectors are also uncorrelated, then

4.3 $$\begin{aligned} \langle n_1 n_2\rangle =0, \end{aligned}$$

which implies

4.4 $$\begin{aligned} \langle \hat{C}_{12}\rangle = \langle h^2\rangle \equiv S_h. \end{aligned}$$

This is just the variance (or power) of the stochastic gravitational-wave signal. So by cross-correlating data in two (or more) detectors, we can extract the common gravitational-wave component.

We have assumed here that there is no cross-correlated noise (instrumental or environmental). If there is correlated noise, then the simple procedure describe above needs to be augmented. This will be discussed in more detail in Sect. 9.6.

### Relating correlations and likelihoods

The cross-correlation approach arises naturally from a standard likelihood analysis if we adopt a Gaussian stochastic template for the signal. Revisiting the example from the previous section, let’s assume that the detector noise is Gaussian-distributed with variances $S_{n_1}$ and $S_{n_2}$. Then the likelihood function for the data $d\equiv (d_1,d_2)$ for the noise-only model $\mathcal{M}_0$ is simply

4.5 $$\begin{aligned} p(d | S_{{n_1}}, S_{{n_2}}, \mathcal{M}_0) = \frac{1}{2\pi \sqrt{S_{{n_1}} S_{{n_2}}}} \, \exp \left[ -\frac{1}{2}\left( \frac{d_1^2}{S_{n_1}}+\frac{d_2^2}{S_{n_2}} \right) \right] . \end{aligned}$$

For the signal-plus-noise model $\mathcal{M}_1$, we have

4.6 $$\begin{aligned} p(d | S_{{n_1}}, S_{{n_2}}, \bar{h}, \mathcal{M}_1) = \frac{1}{2\pi \sqrt{S_{{n_1}} S_{{n_2}}}} \, \exp \left[ -\frac{1}{2}\left\{ \frac{(d_1-\bar{h})^2}{S_{n_1}}+\frac{(d_2-\bar{h})^2}{S_{n_2}} \right\} \right] , \end{aligned}$$

where the gravitational-wave signal $\bar{h}$ is assumed to be a Gaussian random deviate with probability distribution

4.7 $$\begin{aligned} p(\bar{h}|S_h, \mathcal{M}_1) = \frac{1}{\sqrt{2 \pi S_h}}\, \exp \left[ -\frac{1}{2}\frac{\bar{h}^2}{S_h}\right] . \end{aligned}$$

In most applications we are not interested in the value of $\bar{h}$, but rather the power $S_h$. Marginalizing over $\bar{h}$, the likelihood takes the form

4.8 $$\begin{aligned} p(d | S_{n_1}, S_{n_2}, S_h,\mathcal{M}_1) = \frac{1}{\sqrt{\mathrm{det}(2\pi C)}} e^{-\frac{1}{2} \sum _{I,J=1}^2 d_I \left( C^{-1}\right) _{IJ} d_J}, \end{aligned}$$

where

4.9 $$\begin{aligned} C = \left[ \begin{array}{cc} S_{n_1} +S_h &{}\quad S_h \\ S_h &{}\quad S_{n_2} +S_h \\ \end{array} \right] . \end{aligned}$$

Maximizing the likelihood with respect to $S_h$, $S_{n_1}$ and $S_{n_2}$ yields the maximum-likelihood estimators

4.10 $$\begin{aligned} \begin{aligned}&\hat{S}_h = d_1 d_2 = \hat{C}_{12}, \\&\hat{S}_{n_1} = d_{1}^2 - d_{1} d_{2}, \\&\hat{S}_{n_2} = d_{2}^2 - d_{1} d_{2}. \end{aligned} \end{aligned}$$

Thus, the cross-correlation statistic $\hat{C}_{12}$ is the maximum-likelihood estimator for a Gaussian stochastic gravitational wave template with zero mean and variance $S_h$.

### Extension to multiple data samples

The extension to multiple data samples

4.11 $$\begin{aligned} \begin{array}{l@{\quad }l} d_{1i} = h_i + n_{1i}, &{} i=1,2,\ldots , N, \\ d_{2i} = h_i + n_{2i},\ &{} i=1,2,\ldots , N, \end{array} \end{aligned}$$

is fairly straightforward. In the following two subsections, we consider the cases where the detector noise and stochastic signal are either: (i) both *white* (i.e., the data are uncorrelated between time samples) or (ii) both *colored* (i.e., allowing for correlations in time). The white noise example will be analyzed in more detail in Sects. 4.4–4.6.

#### White noise and signal

If the detector noise and stochastic signal are both white, then the likelihood functions for the data $d\equiv \{d_{1i};d_{2i}\}$, are simply *products* of the likelihoods (4.5) and (4.8) for the individual data samples. We can write these product likelihoods as single multivariate Gaussian distributions:

4.12 $$\begin{aligned} p(d|S_{n_1},S_{n_2},\mathcal{M}_0)= & {} \frac{1}{\sqrt{\det (2\pi C_n)}}\, e^{-\frac{1}{2} d^T C_n^{-1} d}, \end{aligned}$$

4.13 $$\begin{aligned} p(d|S_{n_1},S_{n_2},S_h,\mathcal{M}_1)= & {} \frac{1}{\sqrt{\det (2\pi C)}}\, e^{-\frac{1}{2} d^T C^{-1} d}, \end{aligned}$$

where

4.14 $$\begin{aligned} C_n= & {} \left[ \begin{array}{cc} S_{n_1}\,\mathbbm {1}_{N\times N} &{}\quad \mathbf {0}_{N\times N} \\ \mathbf {0}_{N\times N} &{}\quad S_{n_2}\,\mathbbm {1}_{N\times N} \\ \end{array} \right] , \end{aligned}$$

4.15 $$\begin{aligned} C= & {} \left[ \begin{array}{cc} (S_{n_1} +S_h)\,\mathbbm {1}_{N\times N} &{} S_h\,\mathbbm {1}_{N\times N} \\ S_h\,\mathbbm {1}_{N\times N} &{} (S_{n_2} +S_h)\,\mathbbm {1}_{N\times N} \\ \end{array} \right] . \end{aligned}$$

The arguments in the exponential have the form

4.16 $$\begin{aligned} d^T C_n^{-1} d = \sum _{I,J=1}^2\sum _{i,j=1}^N d_{Ii} \left( C^{-1}_n\right) _{Ii,Jj} d_{Jj}, \end{aligned}$$

and similarly for $d^T C^{-1} d$. The maximum-likelihood estimators for this case are:

4.17 $$\begin{aligned} \begin{aligned}&\hat{S}_h \equiv \frac{1}{N}\sum _{i=1}^N d_{1i} d_{2i}, \\&\hat{S}_{n_1} \equiv \frac{1}{N}\sum _{i=1}^N d_{1i}^2 - \frac{1}{N}\sum _{i=1}^N d_{1i} d_{2i}, \\&\hat{S}_{n_2} \equiv \frac{1}{N}\sum _{i=1}^N d_{2i}^2 - \frac{1}{N}\sum _{i=1}^N d_{1i} d_{2i}. \end{aligned} \end{aligned}$$

Note that these are just averages of the single-datum estimators (4.10) over the *N* independent data samples.

A couple of remarks are in order: (i) It is easy to show that the expectation values of the estimators are the true values of the parameters $S_h$, $S_{n_1}$, $S_{n_2}$. It is also fairly straightforward to calculate the variances of the estimators. In particular,

4.18 $$\begin{aligned} \mathrm{Var}(\hat{S}_h) \equiv \langle \hat{S}_h^2\rangle -\langle \hat{S}_h\rangle ^2 =\frac{1}{N}\left[ S_{n_1}S_{n_2} + S_h(S_{n_1} + S_{n_2}) + 2 S_h^2 \right] . \end{aligned}$$

Note that this expression reduces to $\mathrm{Var}(\hat{S}_h)\approx S_{n_1}S_{n_2}/N$ in the weak-signal limit, $S_h\ll S_{n_I}$, for $I=1,2$. (ii) If we simply maximized the likelihood with respect to variations of $S_h$, treating the noise variances $S_{n_1}$ and $S_{n_2}$ as *known* parameters, then the frequentist estimator of $S_h$ would also include *auto-correlation* terms for each detector:

4.19 $$\begin{aligned} \hat{S}_h= & {} \frac{1}{(S_{n_1}+S_{n_2})^2} \left[ 2S_{n_1}S_{n_2} \frac{1}{N}\sum _{i=1}^N d_{1i} d_{2i} \right. \nonumber \\&\left. +\,S_{n_2}\left( \frac{1}{N}\sum _{i=1}^N d_{1i}^2 - S_{n_1}\right) + S_{n_1}\left( \frac{1}{N}\sum _{i=1}^N d_{2i}^2 - S_{n_2}\right) \right] . \end{aligned}$$

In practice, however, the noise variances are not known well enough to be able to extract useful information from the auto-correlation terms; they actually worsen the performance of the simple cross-correlation estimator when the uncertainty in $S_{n_1}$ or $S_{n_2}$ is greater than or equal to $S_h$.

#### Colored noise and signal

For the case where the detector noise and stochastic signal are colored, it simplest to work in the frequency domain, since the Fourier components are *independent* of one another. (This assumes that the data are *stationary*, so that there is no preferred origin of time). Assuming multivariate Gaussian distributions as before, the variances $S_{n_1}$, $S_{n_2}$, and $S_h$ generalize to *power spectral densities*, which are functions of frequency defined by

4.20 $$\begin{aligned} \langle \tilde{n}_I(f)\tilde{n}^*_I(f')\rangle = \frac{1}{2}\delta (f-f')\, S_{n_I}(f), \quad \langle \tilde{h}(f)\tilde{h}^*(f')\rangle = \frac{1}{2}\delta (f-f')\, S_h(f),\quad \end{aligned}$$

where $I=1,2$ and tilde denotes Fourier transform.^7^ The factor of 1 / 2 in (4.20) is for *one-sided* power spectra, for which the integral of the power spectrum over *positive* frequencies equals the variance of the data:

4.21 $$\begin{aligned} \mathrm{Var}[h] =\int _0^\infty df\> S_h(f). \end{aligned}$$

This is just the continuous version of *Parseval’s theorem*, see e.g., (D.40). For *N* samples of discretely-sampled data from each of two detectors $I=1,2$ (total duration *T*), the likelihood function for a Gaussian stochastic signal template becomes (Allen et al. 2002; Cornish and Romano 2013):

4.22 $$\begin{aligned} p(d|S_{n_1}, S_{n_2}, S_h, \mathcal{M}_1) = \prod _{k=0}^{N/2-1} \frac{1}{\mathrm{det}(2\pi \tilde{C}(f_k))} e^{-\frac{1}{2}\sum _{I,J} \tilde{d}_I^*(f_k) \left( \tilde{C}(f_k)^{-1}\right) _{IJ}\tilde{d}_J(f_k)}, \end{aligned}$$

where

4.23 $$\begin{aligned} \tilde{C}(f) = \frac{T}{4}\left[ \begin{array}{cc} S_{n_1}(f) +S_h(f) &{} S_h(f) \\ S_h(f) &{} S_{n_2}(f) +S_h(f) \\ \end{array} \right] . \end{aligned}$$

Here $k=0,1,\ldots , N/2-1$ labels the discrete positive frequencies. There is no square root of the determinant in the denominator of (4.22) since the volume element for the probability density involves both the real and imaginary parts of the Fourier transformed data (Appendix D.6).

We do not bother to write down the maximum-likelihood estimators of the signal and noise power spectral densities for this particular example. We will return to this problem in Sect. 6, where we discuss the *optimally-filtered* cross-correlation statistic for isotropic stochastic backgrounds. There one assumes a particular spectral *shape* for the gravitational-wave power spectral density, and then simply estimates its overall amplitude. That simplifies the analysis considerably.

### Maximum-likelihood detection statistic

Let’s return to the example discussed in Sect. 4.3.1, which consists of *N* samples of data in each of two detectors, having uncorrelated white noise and a common white stochastic signal. As described in Sect. 3.4, one can calculate a frequentist detection statistic based on the *maximum-likelihood ratio*:

4.24 $$\begin{aligned} \Lambda _\mathrm{ML}(d) \equiv \frac{\max _{S_{n_1}, S_{n_2}, S_h} p(d\vert S_{n_1}, S_{n_2}, S_h, \mathcal{M}_1)}{\max _{S_{n_1}, S_{n_2}} p(d\vert S_{n_1}, S_{n_2}, \mathcal{M}_0)}. \end{aligned}$$

Substituting (4.12) and (4.13) for the likelihood functions and performing the maximizations yields

4.25 $$\begin{aligned} \Lambda _\mathrm{ML}(d) = \left[ 1-\frac{\hat{S}_h^2}{\hat{S}_1\hat{S}_2}\right] ^{-N/2}, \end{aligned}$$

where

4.26 $$\begin{aligned} \begin{aligned} \hat{S}_1&\equiv \frac{1}{N}\sum _{i=1}^N d_{1i}^2=\hat{S}_{n_1} + \hat{S}_h, \quad \hat{S}_2 \equiv \frac{1}{N}\sum _{i=1}^N d_{2i}^2=\hat{S}_{n_2} + \hat{S}_h. \end{aligned} \end{aligned}$$

Note that the these estimators involve only *autocorrelations* of the data. In the absence of a signal, they are maximum-likelihood estimators of the noise variances $S_{n_1}$ and $S_{n_2}$. But in the presence of a signal, they are maximum-likelihood estimators of the *combined* variances $S_1\equiv S_{n_1}+S_h$ and $S_2\equiv S_{n_2}+S_h$.

Recall that for comparison with Bayesian model selection calculations, it is convenient to define the frequentist statistic $\Lambda (d)$ as twice the logarithm of the maximum-likelihood ratio:

4.27 $$\begin{aligned} \Lambda (d) \equiv 2\ln \left( \Lambda _\mathrm{ML}(d)\right) = -N \ln \left[ 1-\frac{\hat{S}_h^2}{\hat{S}_1\hat{S}_2}\right] . \end{aligned}$$

In the limit that the stochastic gravitational-wave signal is *weak* compared to the detector noise—i.e., $S_h\ll S_{n_I}$, for $I=1,2$—the above expression reduces to

4.28 $$\begin{aligned} \Lambda (d) \simeq \frac{\hat{S}_h^2}{\hat{S}_1\hat{S}_2/N} \simeq \frac{\hat{S}_h^2}{\hat{S}_{n_1}\hat{S}_{n_2}/N}. \end{aligned}$$

This is just the squared signal-to-noise ratio of the cross-correlation statistic. Note also that $\hat{S}_h^2/\hat{S}_1\hat{S}_2$ is the normalized cross-correlation (i.e., *coherence*) of the data from the two detectors. It is a measure of how well the data in detector 2 *matches* that in detector 1.

From  (4.17), we see that $\Lambda (d)$ is a ratio of the square of a sum of products of Gaussian random variables to the product of a sum of squares of Gaussian random variables. This is a sufficiently complicated expression that we will estimate the distribution of $\Lambda (d)$ *numerically*, doing fake signal injections into many realizations of simulated noise to build up the sampling distribution. We do this explicitly in Sect. 4.6, when we compare the frequentist and Bayesian correlation methods for this example.

### Bayesian correlation analysis

Compared to the frequentist cross-correlation analysis described above, a Bayesian analysis is conceptually much simpler. One simply needs the likelihood functions $p(d|S_{n_1},S_{n_2},\mathcal{M}_0)$ and $p(d|S_{n_1},S_{n_2},S_h,\mathcal{M}_1)$ given by (4.12) and (4.13), and joint prior probability distributions for the signal and noise parameters. For our example, we will assume that the signal and noise parameters are *statistically independent* of one another so that the joint prior distributions factorize into a product of priors for the individual parameters. We use Jeffrey’s priors for the individual noise variances:

4.29 $$\begin{aligned} p_I(S_{n_I}) \propto 1/S_{n_I}, \quad I=1,2, \end{aligned}$$

and a flat^8^ prior for the signal variance:

4.30 $$\begin{aligned} p(S_h) = \mathrm{const}. \end{aligned}$$

Then, using Bayes’ theorem (3.18), we obtain the joint posterior distribution:

4.31 $$\begin{aligned} \begin{aligned} p(S_{n_1},S_{n_2},S_h|d,\mathcal{M}_1)&=\frac{p(d|S_{n_1},S_{n_2},S_h,\mathcal{M}_1) p(S_{n_1},S_{n_2},S_h|\mathcal{M}_1)}{p(d|\mathcal{M}_1)} \\&\propto p(d|S_{n_1},S_{n_2},S_h,\mathcal{M}_1) \frac{1}{S_{n_1}}\frac{1}{S_{n_2}}, \end{aligned} \end{aligned}$$

where $p(d|\mathcal{M}_1)$ is the evidence (or marginalized likelihood) for the signal-plus-noise model $\mathcal{M}_1$. (Similar expressions can be written down for the noise-only model $\mathcal{M}_0$). The marginalized posterior distributions for the signal and noise parameters are given by marginalizing over the other parameters. For example,

4.32 $$\begin{aligned} p(S_h|d,\mathcal{M}_1) \propto \int \frac{dS_{n_1}}{S_{n_1}}\> \int \frac{dS_{n_2}}{S_{n_2}}\> p(d|S_{n_1}, S_{n_2}, S_h, \mathcal{M}_1) \end{aligned}$$

for the signal variance $S_h$.

Correlations enter the Bayesian analysis via the covariance matrix *C* that appears in the likelihood function $p(d|S_{n_1},S_{n_2}, S_h,\mathcal{M}_1)$. The covariance matrix for the data includes the cross-detector signal correlations, as we saw in (4.15). So although one does not explicitly construct a cross-correlation statistic in the Bayesian framework, cross correlations do play an important role in the calculations.

### Comparing frequentist and Bayesian cross-correlation methods

**Fig. 16:**
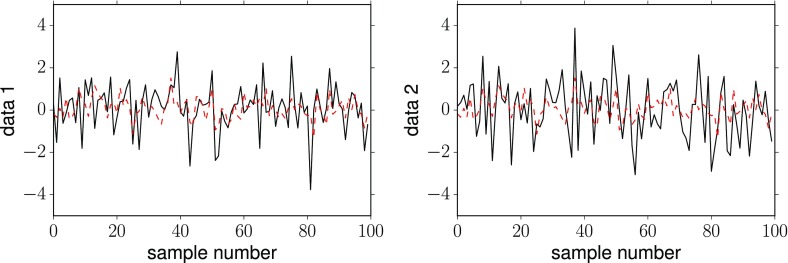
Simulated data in the two detectors. The detector output is shown by the *black curves*; the common stochastic signal is shown by the *red dashed curves*

To explicitly compare the frequentist and Bayesian methods for handling cross-correlations, we simulate data for the white noise, white signal example that we have been discussing in the previous subsections. The particular realization of data that we generate has $N=100$ samples with $S_{n_1} =1$, $S_{n_2}=1.5$, and $S_h=0.3$. Plots of the simulated data in the two detectors are given in Fig. 16.

#### Frequentist analysis

The frequentist maximum-likelihood estimators (4.17) are very easy to calculate. For the simulated data they have values:

4.33 $$\begin{aligned} \hat{S}_{n_1} = 0.78, \quad \hat{S}_{n_2} = 1.46, \quad \hat{S}_h = 0.40. \end{aligned}$$

In addition

4.34 $$\begin{aligned} \Lambda _\mathrm{ML}(d) = 44, \quad \Lambda (d)\equiv 2\ln \left( \Lambda _\mathrm{ML}(d)\right) = 7.6. \end{aligned}$$

The weak-signal approximation to $\Lambda (d)$, given by (4.28), is significantly larger (having a value of 14), since the injected stochastic signal for this case was relatively strong, with the injected $S_h$ equal to $0.3 S_{n_1}$ and $0.2 S_{n_2}$. In addition, for this realization of data, the signal variance was overestimated while both noise variances were underestimated, leading to a much larger value than the nominal squared signal-to-noise ratio of 6.

As mentioned previously, the form (4.27) of the detection statistic $\Lambda (d)$ is sufficiently complicated that it was simplest to resort to numerical simulations to estimate its sampling distribution, $p(\Lambda |S_{n_1}, S_{n_2}, S_h, \mathcal{M}_1)$. We took 50 values for each of $S_{n_1}$, $S_{n_2}$, and $S_h$ in the interval [0, 3], and then for each of the corresponding $50^3$ points in parameter space, we generated $10^4$ realizations of the data, yielding $10^4$ values of $\Lambda (d)$. By histogramming these values for each point in parameter space, we were able to estimate the probability density function (and also the cumulative distribution function) for $\Lambda$.

**Fig. 17:**
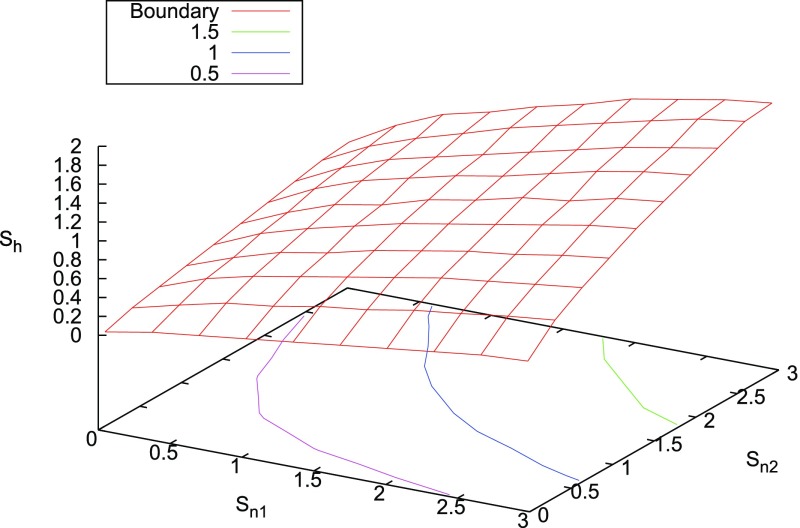
Frequentist 90% confidence-level exclusion and inclusion regions for the simulated data with $\Lambda _\mathrm{obs} = 7.6$. The 90% exclusion region $\mathcal{E}_{90\%}$ lies above the red surface; the 90% inclusion region $\mathcal{I}_{90\%}$ lies below the red surface. The *green*, *blue* and *magenta curves* are projections of the $S_h=1.5, 1.0, 0.5$ level surfaces of the boundary onto the $(S_{n_1}, S_{n_2})$ plane

Figure 17 shows the frequentist 90% confidence-level *exclusion* and *inclusion* regions for our simulated data with $\Lambda _\mathrm{obs}=7.6$. The 90% confidence-level exclusion region $\mathcal{E}_{90\%}$ lies *above* the red surface; it consists of points $(S_{n_1},S_{n_2},S_h)$ satisfying

4.35 $$\begin{aligned} \mathrm{Prob}\left( \Lambda \ge \Lambda _\mathrm{obs}| (S_{n_1}, S_{n_2}, S_h)\in \mathcal{E}_{90\%}\right) \ge 0.90. \end{aligned}$$

The region *below* the red surface is the 90% confidence-level inclusion region $\mathcal{I}_{90\%}$. Note that construction of these regions is such that the *true* values of the parameters $S_{n_1}$, $S_{n_2}$, and $S_h$ have a 90% frequentist probability of lying in $\mathcal{I}_{90\%}$. This generalizes, to multiple parameters, the definition of the frequentist 90% confidence-level upper-limit for a single parameter, which was discussed in detail in Sect. 3.2.3. Note that it is not correct to simply “cut” the surface using the maximum-likelihood point estimates $\hat{S}_{n_1}= 0.78$ and $\hat{S}_{n_2}=1.46$ to obtain a single value for $S_h^{90\%, \mathrm{UL}}$. One needs to include the whole region in order to get the correct frequentist coverage.

A similar procedure can be used to estimate sampling distributions for the frequentist maximum-likelihood estimators $\hat{S}_{n_1}$, $\hat{S}_{n_2}$, and $\hat{S}_h$. From these distributions, one can then calculate e.g., frequentist 95% confidence-level exclusion and inclusion *regions* for the given point estimates. For example, $(S_{n_1}, S_{n_2}, S_h)\in \mathcal{I}_{95\%}$ for the observed point estimate $\hat{S}_{h,\mathrm{obs}}$ if and only if $\hat{S}_{h,\mathrm{obs}}$ is contained in the symmetric 95% confidence-level interval centered on the mode of the probability distribution $p(\hat{S}_h|S_{n_1}, S_{n_2}, S_h, \mathcal{M}_1)$. These regions again generalize to multiple parameters the definition of a frequentist confidence *interval* for a single parameter, which was discussed in detail in Sect. 3.2.4. They will be different, in general, for the different maximum-likelihood estimators. But in order to move on to the Bayesian analysis for this example, we will leave the explicit construction of these regions to the interested reader.

**Fig. 18:**
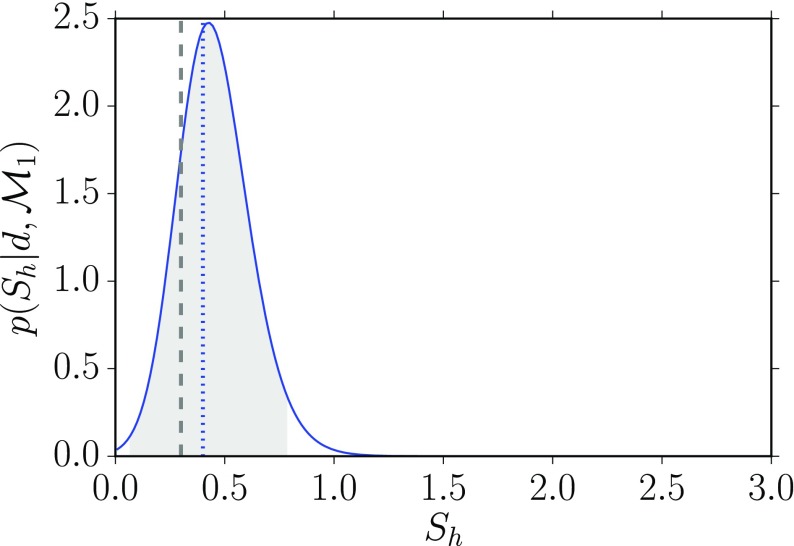
Marginalized posterior distribution for the stochastic signal variance $S_h$ for the signal-plus-noise model $\mathcal{M}_1$. The actual value of $S_h$ used for the simulation is shown by the *grey dashed vertical line*. The 95% Bayesian credible interval centered on the mode of the distribution is the *grey-shaded region*. For comparison, the frequentist maximum-likelihood estimator of $S_h$ is shown by the *blue dotted vertical line*

**Fig. 19:**
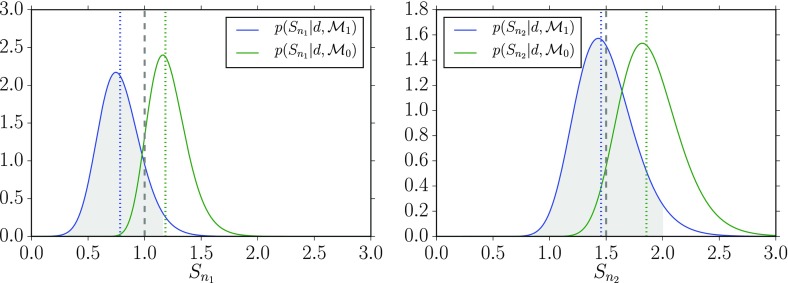
Marginalized posterior distributions for the detector noise variances $S_{n_1}$ (*left panel*) and $S_{n_2}$ (*right panel*) for the signal-plus-noise model $\mathcal{M}_1$ (*blue curves*) and the noise-only model $\mathcal{M}_0$ (*green curves*), respectively. The actual values of $S_{n_1}$ and $S_{n_2}$ used for the simulation are shown by the *grey dashed vertical lines*. The 95% Bayesian credible intervals for the signal-plus-noise model $\mathcal{M}_1$ are the *grey-shaded regions*. For comparison, the frequentist estimators of $S_{n_1}$ and $S_{n_2}$ for the two models are shown by the (*blue and green*) *dotted vertical lines*

#### Bayesian analysis

For the Bayesian analysis of this example, we limit ourselves to calculating the Bayes factor $2 \ln \mathcal{B}_{10}(d)$ comparing the noise-only and signal-plus-noise models $\mathcal{M}_0$ and $\mathcal{M}_1$, as well as the posterior distributions for the three parameters $S_h$, $S_{n_1}$, and $S_{n_2}$. Following the procedure described above in Sect. 4.5 we find, for this particular realization of data,

4.36 $$\begin{aligned} \mathcal{B}_{10}=10, \quad 2\ln \mathcal{B}_{10}(d) = 4.6. \end{aligned}$$

This Bayes factor corresponds to *positive* evidence (see Table 3) in favor of a correlated stochastic signal in the data.

Figure 18 shows the marginalized posterior $p(S_h|d,\mathcal{M}_1)$ for the stochastic signal variance given the data *d* and signal-plus-noise model $\mathcal{M}_1$. The peak of the posterior lies close the frequentist maximum-likelihood estimator $\hat{S}_h=0.40$ (blue dotted vertical line), and easily contains the injected value in its 95% Bayesian credible interval (grey shaded region). Figure 19 shows similar plots for the marginalized posteriors for the noise variances $S_{n_1}$ and $S_{n_2}$ for both the signal-plus noise model $\mathcal{M}_1$ (blue curves) and the noise-only model $\mathcal{M}_0$ (green curves). For comparison, the frequentist maximum-likelihood estimators $\hat{S}_{n_1}, \hat{S}_{n_2}=0.78, 1.46$ and 1.18, 1.86 for the two models are shown by the corresponding (blue and green) dotted vertical lines. Again, the peaks of the Bayesian posterior distributions lie close to these values. The 95% Bayesian credible intervals for $S_{n_1}$ and $S_{n_2}$ for the signal-plus-noise model $\mathcal{M}_1$ are also shown (grey shaded region). These intervals easily contain the injected values for these two parameters.

### What to do when cross-correlation methods aren’t available

Cross-correlation methods can be used whenever one has two or more detectors that respond to a common gravitational-wave signal. The beauty of such methods is that even though a stochastic background is another source of “noise” in a single detector, the common signal components in multiple detectors combine coherently when the data from pairs of detectors are multiplied together and summed, as described in Sect. 4.1. But with only a single detector, searches for a stochastic background need some other way to distinguish the signal from the noise—e.g., a difference between the spectra of the noise and the gravitational-wave signal, or the modulation of an anisotropic signal due to the motion of the detector (as is expected for the confusion-noise from galactic compact white dwarf binaries for LISA). Without some way of differentiating instrumental noise from gravitational-wave “noise”, there is no hope of detecting a stochastic background.

As a simple example, suppose that we have *N* samples of data from each of two detectors $I=1,2$ (which we will call *channels* in what follows), but let’s assume that the second channel is *insensitive* to the gravitational-wave signal:

4.37 $$\begin{aligned} \begin{array}{l@{\quad }l} d_{1i}=h_i + n_{1i}, &{} \quad i=1,2,\ldots , N, \\ d_{2i}=n_{2i}, &{} \quad i=1,2,\ldots , N. \end{array} \end{aligned}$$

Then if we make the same assumptions as before for the signal and the noise, it follows that the likelihood function for the data $d\equiv \{d_{1i};d_{2i}\}$ is given by

4.38 $$\begin{aligned} p(d|S_{n_1}, S_{n_2}, S_h, \mathcal{M}_1)= \frac{1}{\sqrt{\det (2\pi C)}} e^{-\frac{1}{2}d^T C^{-1} d}, \end{aligned}$$

where

4.39 $$\begin{aligned} C= \left[ \begin{array}{cc} (S_{n_1}+S_h)\,\mathbbm {1}_{N\times N} &{}\quad \mathbf {0}_{N\times N} \\ \mathbf {0}_{N\times N} &{}\quad S_{n_2}\,\mathbbm {1}_{N\times N} \\ \end{array} \right] \end{aligned}$$

is the covariance matrix of the data. Since the off-diagonal blocks of the covariance matrix are identically zero, it is clear that we will not be able to use the cross-correlation methods developed in the previous sections. So we need to do something else if we are going to extract the gravitational-wave signal from the noise.

#### Single-detector excess power statistic

If we knew $S_{n_1}$ a priori, then we could construct an *excess power* statistic from the autocorrelated data to estimate the signal variance:

4.40 $$\begin{aligned} \hat{S}_h \equiv \frac{1}{N}\sum _{i=1}^N d_{1i}^2-S_{n_1}. \end{aligned}$$

(This is effectively how Penzias and Wilson (1965) discovered the CMB; they observed excess antenna noise that they could not attribute to any other known source of noise). But as mentioned at the end of Sect. 4.3.1, typically we do not know the detector noise well enough to use such a statistic, since the uncertainty in $S_{n_1}$ is much greater than the variance of the gravitational-wave signal that we are trying to detect. This is definitely the case for ground-based detectors like LIGO, Virgo, etc. An exception to this “rule” will probably be the predicted *foreground* signal from galactic white-dwarf binaries in the LISA band. For frequencies below a few mHz, the gravitational-wave confusion noise from these binaries is expected to dominate the LISA instrument noise (Hils et al. 1990; Bender and Hils 1997; Hils and Bender 2000; Nelemans et al. 2001).

#### Null channel method

If it were possible to make an *off-source* measurement using detector 1, then we could estimate the noise variance $S_{n_1}$ directly from the detector output, free of contamination from gravitational waves. Using this noise estimate, $\hat{S}_{n_1}$, we could then define our excess power statistic as

4.41 $$\begin{aligned} \hat{S}_h \equiv \frac{1}{N}\sum _{i=1}^N d_{1i}^2 -\hat{S}_{n_1}. \end{aligned}$$

Unfortunately, such off-source measurements are not possible, since you cannot shield a gravitational-wave detector from gravitational waves. However, in certain cases one can construct a particular *combination* of the data (called a *null channel*) for which the response to gravitational waves is strongly suppressed. The *symmetrized Sagnac* combination of the data for LISA (Tinto et al. 2001; Hogan and Bender 2001) is one such example.

So let us assume that channel 2 for our example is such a null channel, and let us also assume that there is some relationship between the noise in the two channels—e.g., $S_{n_1} = aS_{n_2}$, with $a>0$. (For colored noise, the variances would be replaced by power spectra and *a* would be replaced by a function of frequency—i.e., a *transfer function* relating the noise in the two channels). To begin with, we will also assume that *a* is *known*. Then the data from the second channel can be used as a *noise calibrator* for the first channel. The frequentist estimators for this scenario are:

4.42 $$\begin{aligned} \begin{aligned}&\hat{S}_{n_2} = \frac{1}{N}\sum _{i=1}^N d_{2i}^2, \\&\hat{S}_{n_1} = a\hat{S}_{n_2}, \\&\hat{S}_h = \frac{1}{N}\sum _{i=1}^N d_{1i}^2 - \hat{S}_{n_1}. \end{aligned} \end{aligned}$$

These are the maximum-likelihood estimators of the signal and noise parameters, derived from the likelihood (4.38) with $S_{n_1}$ replaced by $aS_{n_2}$. In the Bayesian framework, the relation $S_{n_1}= a S_{n_2}$ is encoded in the joint prior probability distribution

4.43 $$\begin{aligned} p(S_{n_1},S_{n_2}) = \delta (S_{n_1}-aS_{n_2})p_2(S_{n_2}), \end{aligned}$$

which eliminates $S_{n_1}$ as an independent variable. The marginalized posterior distribution for the signal variance $S_h$, assuming a flat prior $p_h(S_h)=\mathrm{const}$, is then

4.44 $$\begin{aligned} p(S_h|d) \propto \int dS_{n_2}\> p(d|S_{n_1}=aS_{n_2},S_{n_2},S_h) p_2(S_{n_2}). \end{aligned}$$

In the more realistic scenario where the transfer function *a* is not known a priori, but is described by its own prior probability distribution $p_a(a)$, we have

4.45 $$\begin{aligned} p(S_{n_1}, S_{n_2}, a) = \delta (S_{n_1}- aS_{n_2})p_a(a)p_2(S_{n_2}) \end{aligned}$$

and

4.46 $$\begin{aligned} p(S_h|d) \propto \int da \int dS_{n_2}\> p(d|S_{n_1}=aS_{n_2},S_{n_2},S_h) p_a(a) p_2(S_{n_2}). \end{aligned}$$

This integral can be done numerically given priors for $S_{n_2}$ and *a*.

**Fig. 20:**
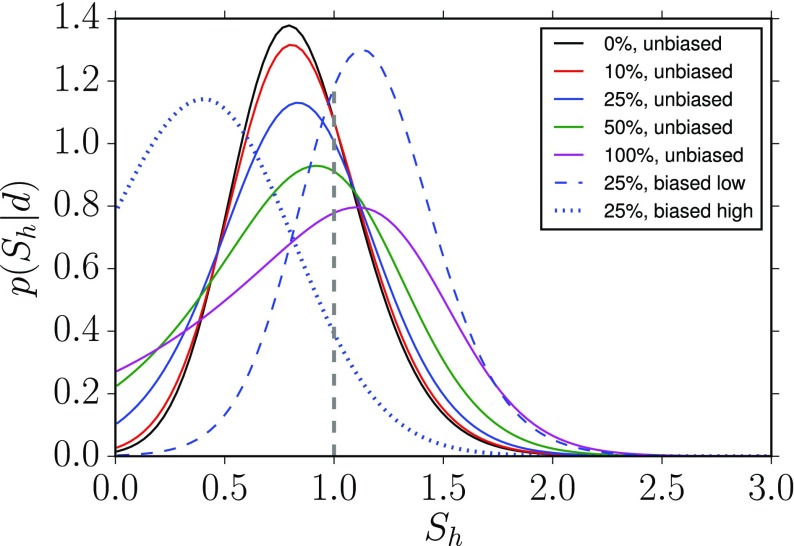
Posterior distributions for $S_h$ for the null channel analysis, corresponding to different priors for the parameter *a*, which relates the instrumental noise variances in the two channels. The labels “*p*%, unbiased” correspond to $A=a_0$ and $\Sigma =1+p/100$; the labels “25%, biased low (or high)” correspond to $A=0.67 a_0$ (or $1.5 a_0$) and $\Sigma = 1.25$. The *vertical grey dashed line* corresponds to the injected value of $S_h$

To help illustrate the above discussion, Fig. 20 shows plots of several different posterior distributions for $S_h$, corresponding to different choices for the prior distribution $p_a(a)$. For these plots, we chose a *Jeffrey’s prior* for $S_{n_2}$:

4.47 $$\begin{aligned} p_2(S_{n_2})\propto 1/S_{n_2}, \end{aligned}$$

and a *log-normal* prior for *a*:

4.48 $$\begin{aligned} p(a|\mu ,\sigma ) = \frac{1}{a}\frac{1}{\sqrt{2\pi }\sigma } e^{-\frac{1}{2}\frac{(\ln a-\mu )^2}{\sigma ^2}}. \end{aligned}$$

The different curves correspond to different values of $\mu$ and $\sigma$:

4.49 $$\begin{aligned} \begin{aligned}&\mu \equiv \ln A ,\quad A = a_0, 0.67a_0, 1.5a_0, \\&\sigma \equiv \ln \Sigma , \quad \Sigma = 1, 1.1, 1.25, 1.5, 2, \end{aligned} \end{aligned}$$

where $a_0$ denotes the nominal (true) value of *a*. Note that $A=0.67a_0$ and $1.5a_0$ correspond to priors for *a* that are *biased* away from its true value $a=a_0$. Note also that 68% of the prior distribution is contained in the region $a\in [A/\Sigma , A\Sigma ]$ (so $\Sigma =1$ corresponds to a delta-function prior—i.e., no uncertainty in *a*). The particular realization that we used consisted of $N=100$ samples of data (4.37) with $S_h=1$, $S_{n_2}=1$, and $S_{n_1} = a_0 S_{n_2}$ with $a_0=1$. Note that for the biased priors for *a* (associated with the dashed and dotted curves in Fig. 20), an under (over) estimate in *a* corresponds to over (under) estimate in $S_h$, as $S_h$ is effectively the difference between the estimated variance in channel 1 and *a* times the estimated variance in channel 2. For this particular realization of the data, the mode of the “0%, unbiased” posterior for $S_h$ is about 20% less than the injected value, $S_h=1$. On average, they would agree.

## Geometrical factors

There is geometry in the humming of the strings, there is music in the spacing of the spheres. *Pythagoras*

In the previous sections, we ignored many details regarding detector response and detector geometry. We basically assumed that the detectors were *isotropic*, responding equally well to all gravitational waves, regardless of the waves’ directions of propagation, frequency content, and polarization. We also ignored any loss in sensitivity in the correlations between data from two or more detectors, due to the separation and relative orientation of the detectors. But these details *are* important if we want to design optimal (or near-optimal) data analysis algorithms to search for gravitational waves. To specify the likelihood function, for example, requires models not only for the gravitational-wave signal and instrument noise, but also for the response of the detectors to the waves that a source produces.

In this section, we fill in these details. We first discuss the response of a single detector to an incident gravitational wave. We then show how these non-trivial detector responses manifest themselves in the correlation between data from two or more detectors. The results are first derived in a general setting making no assumption, for example, about the wavelength of a gravitational wave to the characteristic size of a detector. The general results are then specialized, as appropriate, to the case of ground-based and space-based laser interferometers, spacecraft Doppler tracking, and pulsar timing arrays. We conclude this section by discussing how the motion of a detector relative to the gravitational-wave source affects the detector response.

The approach we take in this section is similar in spirit to that of Hellings (1991), attempting to unify the treatment of detector response functions and correlation functions across different gravitational-wave detectors. Readers interested in more details about the effect of detector geometry on the correlation of data from two or more detectors should see the original papers by Hellings and Downs (1983) for pulsar timing arrays, and Flanagan (1993) and Christensen (1990, 1992) for ground-based laser interferometers.

### Detector response

Gravitational waves are time-varying perturbations to the background geometry of spacetime. Since gravitational waves induce time-varying changes in the separation between two freely-falling objects (so-called test masses), gravitational-wave detectors are designed to be as sensitive as possible to this changing separation. For example, a resonant bar detector acts like a giant tuning fork, which is set into oscillation when a gravitational wave of the natural frequency of the bar is incident upon it. These oscillations produce a stress against the equilibrium electromagnetic forces that exist within the bar. The stress (or oscillation) is measured by a strain gauge (or accelerometer), indicating the presence of a gravitational wave. The response for a bar detector is thus the fractional change in length of the bar, $h(t) = \Delta l(t)/l$, induced by the wave. Since the length of the bar is typically much smaller than the wavelength of a gravitational wave at the bar’s resonant frequency, the response is most easily computed using the geodesic deviation equation (Misner et al. 1973) for the time-varying tidal field.

In this article, we will focus our attention on *beam* detectors, which use electromagnetic radiation to monitor the separation of two or more freely-falling objects. Spacecraft Doppler tracking, pulsar timing arrays, and ground- and space-based laser interferometers (e.g., LIGO-like and LISA-like detectors) are all examples of beam detectors, which can be used to search for gravitational waves (see, e.g., Section 4.2 in Sathyaprakash and Schutz 2009).

#### Spacecraft Doppler tracking

For spacecraft Doppler tracking, pulses of electromagnetic radiation are sent from one test mass (e.g., a radio transmitting tower on Earth) to another (e.g., the Cassini probe), and then bounced back (or coherently transponded) from the second test mass to the first. From the arrival times of the returning pulses, one can calculate the fractional change in the frequency of the emitted pulses induced by a gravitational wave. The detector response for such a measurement is thus

**Fig. 21:**
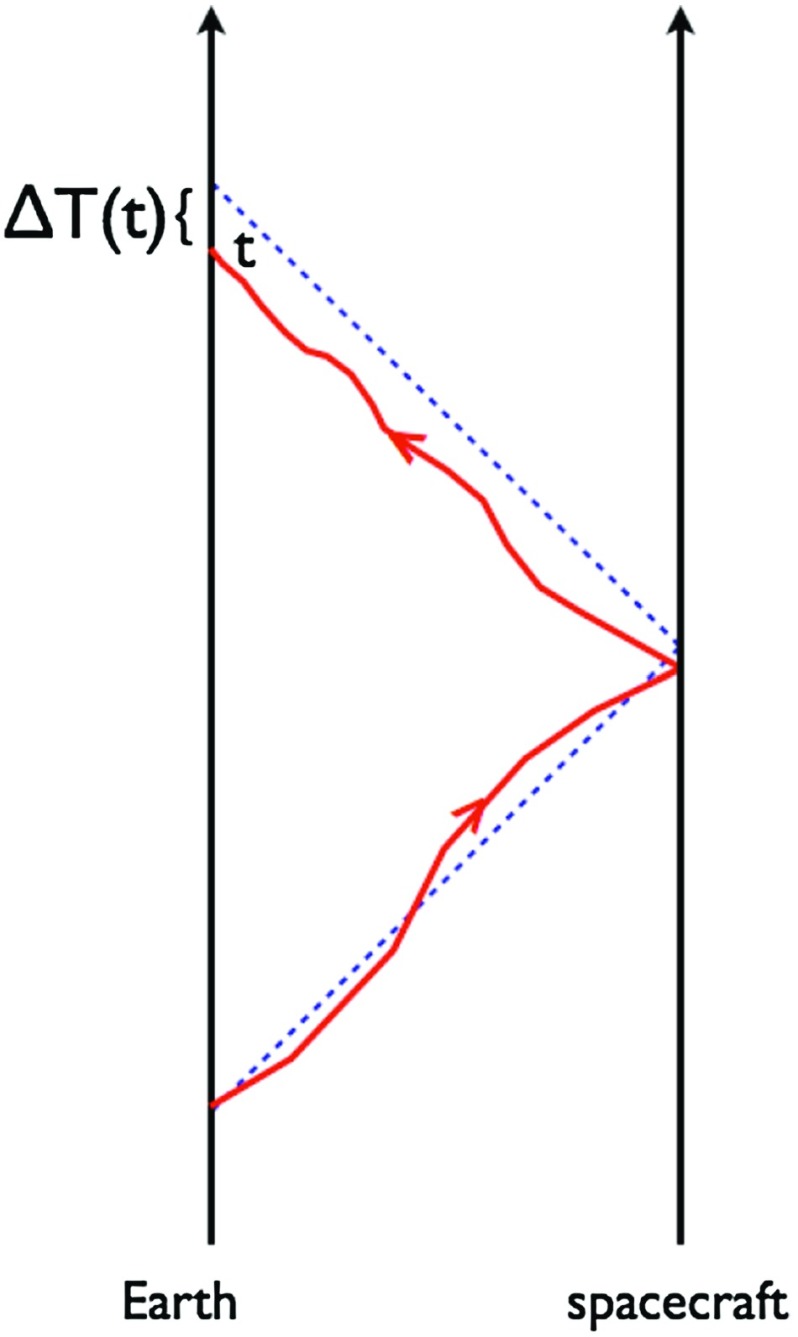
A spacetime diagram representation of $\Delta T(t)$ for a two-way spacecraft Doppler tracking measurement. Time increases vertically upward. The *vertical arrows* are spacetime worldlines for the Earth and a spacecraft. The measurement is made at time *t*. The *blue dotted line* shows the trajectory of a pulse of electromagnetic radiation in the absence of a gravitational wave; the *red solid line* shows the trajectory in the presence of a gravitational wave

5.1 $$\begin{aligned} h_\mathrm{doppler}(t)\equiv \frac{\Delta \nu (t)}{\nu _0}=\frac{d\Delta T(t)}{dt}, \end{aligned}$$

where $\Delta T(t)$ is the deviation of the round-trip travel time of a pulse away from the value it would have had at time *t* in the absence of the gravitational wave. A schematic representation of $\Delta T(t)$ for spacecraft Doppler tracking is given in Fig. 21.

#### Pulsar timing

Pulsar timing is even simpler in the sense that we only have *one-way* transmission of electromagnetic radiation (i.e., radio pulses are emitted by a pulsar and received by a radio antenna on Earth). The response for such a system is simply the timing residual

**Fig. 22:**
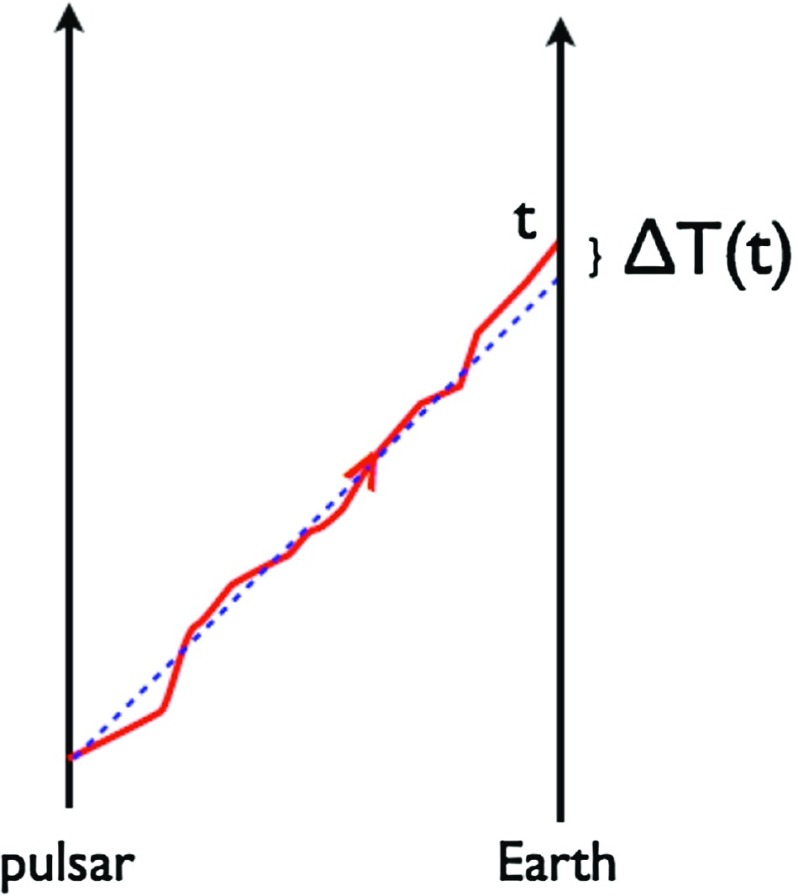
A spacetime diagram representation of $\Delta T(t)$ for a (one-way) pulsar timing residual measurement. Time increases vertically upward. The *vertical arrows* are spacetime worldlines for a pulsar and a detector on Earth. The measurement is made at time *t*. The *blue dotted line* shows the trajectory of the radio pulse in the absence of a gravitational wave; the *red solid line* shows the trajectory in the presence of a gravitational wave

5.2 $$\begin{aligned} h_\mathrm{timing}(t) = \Delta T(t), \end{aligned}$$

which is the difference between the measured time of arrival of a radio pulse and the expected time of arrival of the pulse (as determined from a detailed timing model for the pulsar) due to the presence of a gravitational wave. A schematic representation of $\Delta T(t)$ for a pulsar timing measurement is given in Fig. 22.

#### Laser interferometers

For laser interferometers like LIGO or LISA, the detector response is the phase difference in the laser light sent down and back the two arms of the interferometer. Again, the phase difference can be calculated in terms of the change in the round-trip travel time of the laser light from one test mass (e.g., the beam splitter) to another (e.g., one of the end test masses). If we consider an equal-arm Michelson interferometer with unit vectors $\hat{u}$ and $\hat{v}$ pointing from the beam splitter to the end masses in each of the arms, then

**Fig. 23:**
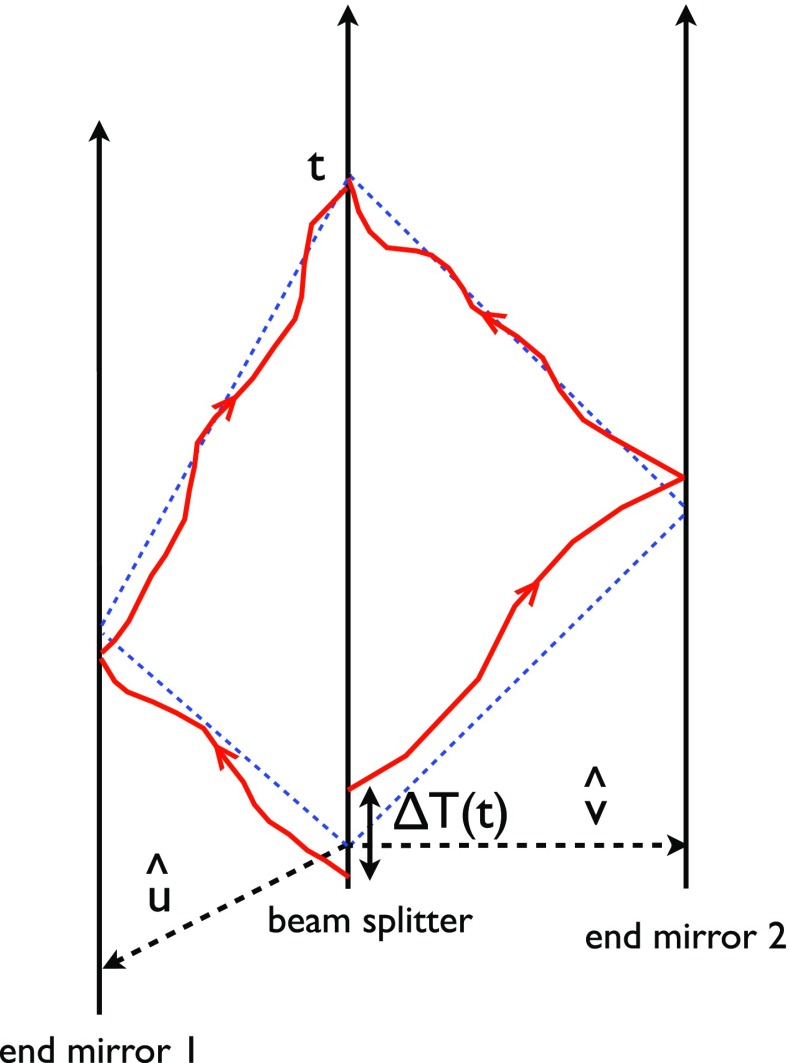
A spacetime diagram representation of $\Delta T(t)$ for an equal-arm Michelson interferometer. Time increases vertically upward. The *vertical arrows* are spacetime worldlines for the beam splitter and two end mirrors. The *blue dotted lines* show the trajectory of the laser light in the two arms of the interferometer in the absence of a gravitational wave; the *red solid lines* show the trajectory in the presence of a gravitational wave. The *black dotted arrows*, labeled $\hat{u}$ and $\hat{v}$, show the orientation of the two arms, from beam splitter to end mirrors, at $t=0$, assuming an opening angle of $90^\circ$

5.3 $$\begin{aligned} h_\mathrm{phase}(t) \equiv \Delta \Phi (t) = 2\pi \nu _0 \Delta T(t), \end{aligned}$$

where $\Delta T(t)\equiv T_{{\hat{u}},\mathrm{rt}}(t) -T_{{\hat{v}},\mathrm{rt}}(t)$ is the difference of the round-trip travel times, and $\nu _0$ is the frequency of the laser light. (See Fig. 23). Alternatively, one often writes the interferometer response as a *strain* measurement in the two arms

5.4 $$\begin{aligned} h_\mathrm{strain}(t) \equiv \frac{\Delta L(t)}{L} =\frac{\Delta T(t)}{2L/c}, \end{aligned}$$

where $\Delta L(t)\equiv L_{\hat{u}}(t)-L_{\hat{v}}(t)$ is the difference of the proper lengths of the two arms (having unperturbed length *L*), and $\Delta T(t)$ is the difference in round-trip travel times as before. Thus, interferometer phase and strain response are simply related to one another.

Calculation of $\Delta T(t)$ for beam detectors is most simply carried out in the transverse-traceless gauge^9^ (Misner et al. 1973; Schutz 1985; Hartle 2003) since the unperturbed separation *L* of the two test masses can be larger than or comparable to the wavelength $\lambda \equiv c/f$ of an incident gravitational wave having frequency *f*. This is definitely the case for pulsar timing where *L* is of order a few kpc, and for spacecraft Doppler tracking where *L* is of order tens of AU. It is also the case for space-based detectors like LISA ($L=5\times 10^6 \mathrm{\ km}$) for gravitational waves with frequencies around a tenth of a Hz. On the other hand, for Earth-based detectors like LIGO ($L=4~\mathrm{km}$), $L\ll \lambda$ is a good approximation below a few kHz. Thus, the approach that we will take in the following subsections is to calculate the detector response in general, not making any approximation a priori regarding the relative sizes of $\lambda =c/f$ and *L*. To recover the standard expressions (i.e., in the long-wavelength or small-antenna limit) for Earth-based detectors like LIGO will be a simple matter of taking the limit *fL* / *c* to zero. For reference, Table 5 summarizes the characteristic properties (i.e., size, characteristic frequency, sensitivity band, etc.) of different beam detectors.

**Table 5:** Characteristic properties of different beam detectors: column 2 is the arm length or characteristic size of the detector (tens of AU for spacecraft Doppler tracking; a few kpc for pulsar timing); column 3 is the frequency corresponding to the characteristic size of the detector, $f_*\equiv c/L$; columns 4 and 5 are the frequencies at which the detector is sensitive in units of Hz and units of $f_*$, respectively; and column 6 is the relationship between *f* and $f_*$

Beam detector	*L* (km)	$f_*$ (Hz)	*f* (Hz)	$f/f_*$	Relation
Ground-based interferometer	${\sim } 1$	${\sim } 10^5$	$10\,\,\hbox {to}\,10^4$	$10^{-4}\,\hbox {to}\,10^{-1}$	$f\ll f_*$
Space-based interferometer	${\sim } 10^6$	${\sim } 10^{-1}$	$10^{-4}\,\hbox {to}\,10^{-1}$	$10^{-3}\,\hbox {to}\, 1$	$f\lesssim f_*$
Spacecraft Doppler tracking	${\sim } 10^9$	${\sim } 10^{-4}$	$10^{-6}\,\hbox {to}\,10^{-3}$	$10^{-2}\,\hbox {to}\,10$	$f\sim f_*$
Pulsar timing	${\sim } 10^{17}$	${\sim } 10^{-12}$	$10^{-9}\,\hbox {to}\,10^{-7}$	$10^3\,\hbox {to}\,10^5$	$f\gg f_*$

### Calculation of response functions and antenna patterns

Gravitational waves are weak. Thus, the detector response is *linear* in the metric perturbations $h_{ab}(t,{\vec {x}})$ describing the wave, and can be written as the convolution of the metric perturbations $h_{ab}(t,{\vec {x}})$ with the *impulse response* $R^{ab}(t,{\vec {x}})$ of the detector:

5.5 $$\begin{aligned} h(t) = (\mathbf {R}*\mathbf {h})(t,{\vec {x}}) \equiv \int _{-\infty }^\infty d\tau \int d^3y\, R^{ab}(\tau ,\vec {y}) h_{ab}(t-\tau ,{\vec {x}}-\vec {y}), \end{aligned}$$

where ${\vec {x}}$ is the location of the measurement at time *t*. In terms of a plane-wave expansion (2.1) of the metric perturbations, we have

5.6 $$\begin{aligned} h(t) = \int _{-\infty }^\infty df \int d^2\Omega _{\hat{n}} R^{ab}(f,\hat{n}) h_{ab}(f,\hat{n}) e^{i2\pi f t}, \end{aligned}$$

or, in the frequency domain,

5.7 $$\begin{aligned} \tilde{h}(f) = \int d^2\Omega _{\hat{n}} R^{ab}(f,\hat{n}) h_{ab}(f,\hat{n}), \end{aligned}$$

where^10^

5.8 $$\begin{aligned} R^{ab}(f,\hat{n}) =e^{i2\pi f\hat{n}\cdot {\vec {x}}/c} \int _{-\infty }^\infty \mathrm{d}\tau \int \mathrm{d}^3 y\> R^{ab}(\tau ,\vec {y})\, e^{-i2\pi f(\tau +\hat{n}\cdot \vec {y}/c)}. \end{aligned}$$

Further specification of the response function depends on the choice of gravitational-wave detector as well as on the basis tensors used to expand $h_{ab}(f,\hat{n})$, as we shall see below and in the following subsections.

For example, if we work in the polarization basis, with expansion coefficients $h_A(f,\hat{n})$, where $A=\{+,\times \}$, then

5.9 $$\begin{aligned} \tilde{h}(f) = \int d^2\Omega _{\hat{n}} \sum _A R^A(f,\hat{n})h_A(f,\hat{n}), \end{aligned}$$

with

5.10 $$\begin{aligned} R^A(f,\hat{n}) = R^{ab}(f,\hat{n})e^A_{ab}(\hat{n}). \end{aligned}$$

If we work instead in the tensor spherical harmonic basis, with expansion coefficients $a^P_{(lm)}(f)$, where $P=\{G,C\}$, then

5.11 $$\begin{aligned} \tilde{h}(f) = \sum _{(lm)} \sum _P R^P_{(lm)}(f)a^P_{(lm)}(f), \end{aligned}$$

with

5.12 $$\begin{aligned} R^P_{(lm)}(f) = \int d^2\Omega _{\hat{n}}\> R^{ab}(f,\hat{n})Y^{P}_{(lm)ab}(\hat{n}). \end{aligned}$$

Note that in the polarization basis the response function $R^A(f,\hat{n})$ is the detector response to a sinusoidal plane-wave with frequency *f*, coming from direction $\hat{n}$, and having polarization $A=+,\times$. Plots of $|R^A(f,\hat{n})|$ for fixed frequency *f* are *antenna beam patterns* for gravitational waves with polarization *A*. A plot of

5.13 $$\begin{aligned} \mathcal{R}(f,\hat{n})\equiv \left( |R^+(f,\hat{n})|^2+ |R^\times (f,\hat{n})|^2\right) ^{1/2} \end{aligned}$$

for fixed frequency *f* is the beam pattern for an *unpolarized* gravitational wave—i.e., a wave having statistically equivalent $+$ and $\times$ polarization components.

Since the previous subsection showed that the response of all beam detectors can be written rather simply in terms of the change in the light-travel time of an electromagnetic wave propagating between two test masses, we now calculate $\Delta T(t)$ in various scenarios and use the resulting expressions to read-off the response functions $R^{ab}(f,\hat{n})$ for the different detectors. We also make plots of various antenna patterns.

**Fig. 24:**
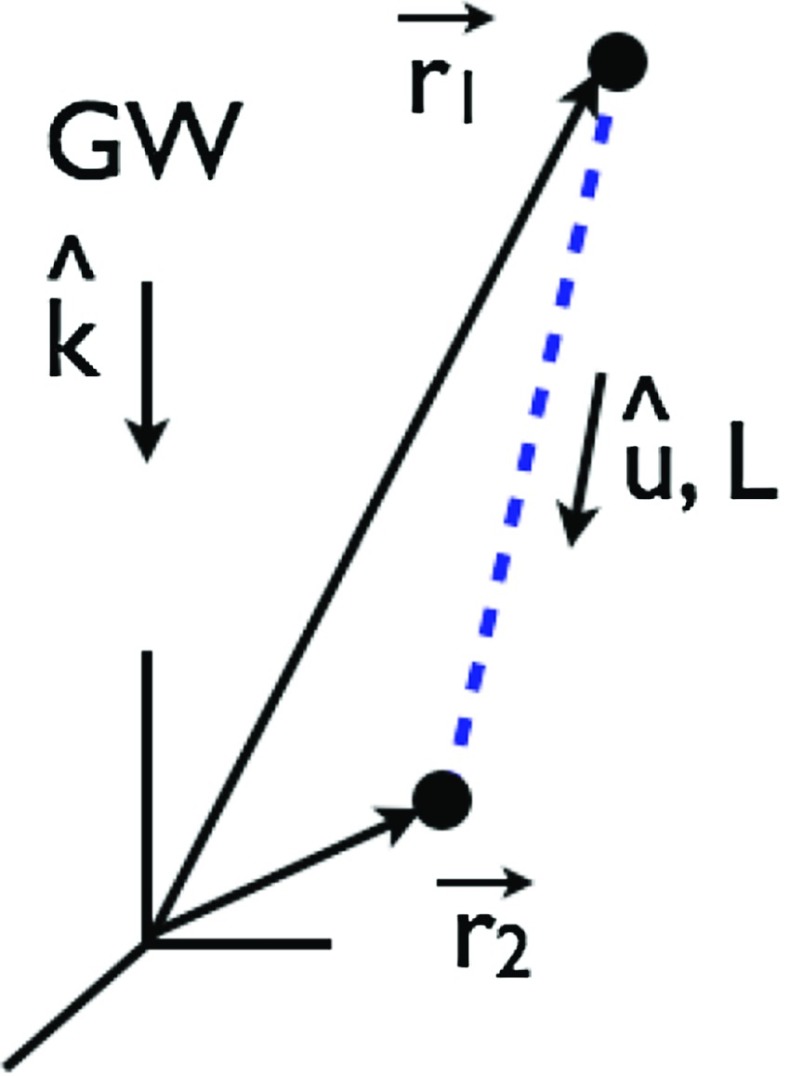
Geometry for calculating the change in the photon propagation time from $\vec {r}_1$ to $\vec {r}_2=r_1+L\hat{u}$ in the presence of a plane gravitational wave propagating in direction $\hat{k}$

#### One-way tracking

Consider two test masses located at position vectors $\vec {r}_1$ and $\vec {r}_2=\vec {r}_1+L\hat{u}$, respectively, in the presence of a plane gravitational wave propagating in direction $\hat{k} = -\hat{n}$, as shown in Fig. 24. Then the change in the light-travel time for a photon emitted at $\vec {r}_1$ and received at $\vec {r}_2$ at time *t* is given by Estabrook and Wahlquist (1975):

5.14 $$\begin{aligned} \Delta T(t) =\frac{1}{2c} u^a u^b \int _{s=0}^L ds\, h_{ab}(t(s),{\vec {x}}(s)), \end{aligned}$$

where the 0th-order expression for the photon trajectory can be used in $h_{ab}$:

5.15 $$\begin{aligned} t(s) = (t-L/c) + s/c, \quad {\vec {x}}(s) = \vec {r}_1 + s\hat{u}. \end{aligned}$$

Since $h_{ab}(t,{\vec {x}}) = h_{ab}(t+\hat{n}\cdot {\vec {x}}/c)$ for a plane wave, it is relatively easy to do the integral. The result is

5.16 $$\begin{aligned} {\Delta T(t)}= & {} \int _{-\infty }^\infty df \frac{1}{2} u^a u^b h_{ab}(f,\hat{n})\, \nonumber \\&\quad \quad \times \,\frac{1}{i 2\pi f}\, \frac{1}{1+\hat{n}\cdot \hat{u}}\, \left[ e^{i 2\pi f(t_2+\hat{n}\cdot \vec {r}_2/c)}- e^{i 2\pi f(t_1+\hat{n}\cdot \vec {r}_1/c)} \right] \end{aligned}$$

5.17 $$\begin{aligned}= & {} \int _{-\infty }^\infty df \frac{1}{2} u^a u^b h_{ab}(f,\hat{n})\, e^{i 2\pi f (t+\hat{n}\cdot \vec {r}_2/c)} \nonumber \\&\quad \quad \times \,\frac{1}{i 2\pi f}\, \frac{1}{1+\hat{n}\cdot \hat{u}}\, \left[ 1-e^{-\frac{i2\pi f L}{c}(1+\hat{n}\cdot \hat{u})}\right] , \end{aligned}$$

where we factored out $e^{i 2\pi f (t+\hat{n}\cdot \vec {r}_2/c)}$, corresponding to the time and location of the measurement, to get the last line. Note that the two terms in square brackets in (5.16) correspond to sampling the gravitational-wave phase at photon reception (location $\vec {r}_2$ at time $t_2\equiv t$) and photon emission (location $\vec {r}_1$ at time $t_1\equiv t-L/c$), respectively. In the context of pulsar timing, these two terms are called the *Earth term* and *pulsar term*, respectively.

From Eq. (5.17), we can read-off the response function for a timing residual measurement, $h_\mathrm{timing}(t)\equiv \Delta T(t)$. It is

5.18 $$\begin{aligned} R^{ab}_\mathrm{timing}(f,\hat{n}) =\frac{1}{2} u^a u^b\, \mathcal{T}_{{\vec {u}}}(f,\hat{n}\cdot \hat{u}) e^{i2\pi f\hat{n}\cdot \vec {r}_2/c}, \end{aligned}$$

where

**Fig. 25:**
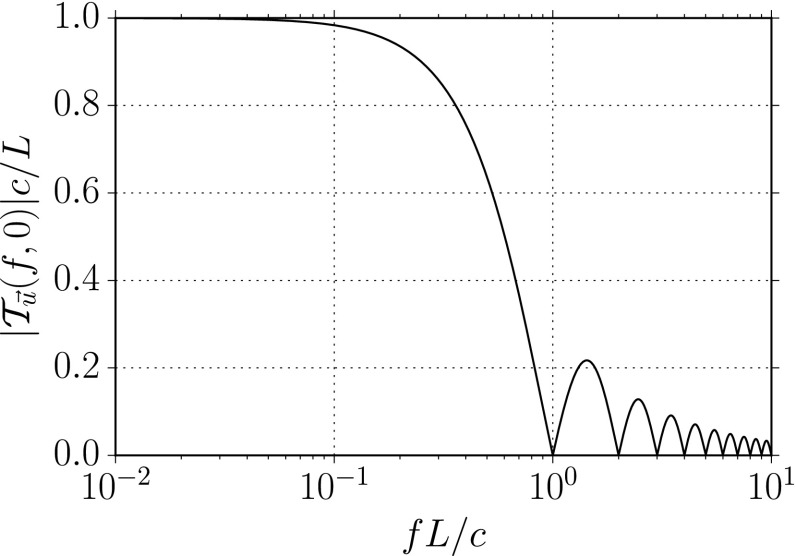
Magnitude of the one-way tracking timing transfer function $|\mathcal{T}_{{\vec {u}}}(f,0)|$ for normal incidence of the gravitational wave, plotted on a logarithmic frequency scale. Nulls in the transfer function occur at frequencies equal to integer multiples of *c* / *L*

5.19 $$\begin{aligned} \begin{aligned} \mathcal{T}_{{\vec {u}}}(f,\hat{n}\cdot \hat{u})&\equiv \frac{1}{i 2\pi f}\, \frac{1}{1+\hat{n}\cdot \hat{u}}\, \left[ 1-e^{-\frac{i2\pi f L}{c}(1+\hat{n}\cdot \hat{u})}\right] \\&= \frac{L}{c} e^{-\frac{i\pi fL}{c}(1+\hat{n}\cdot \hat{u})}\, \mathrm{sinc}\left( \frac{\pi fL}{c}[1+\hat{n}\cdot \hat{u}]\right) \end{aligned} \end{aligned}$$

is the *timing transfer function* for one-way photon propagation along ${\vec {u}}=L\hat{u}$. (Here $\mathrm{sinc}\,x \equiv \sin x/x$). If we choose $\vec {r}_2$ to be the origin of coordinates, then $\mathcal{T}_{{\vec {u}}}(f,\hat{n}\cdot \hat{u})$ contains all the frequency-dependence of the timing response. For example, for normal incidence of the gravitational wave ($\hat{n}\cdot \hat{u}=0$), $|\mathcal{T}_{{\vec {u}}}(f,0)| = (L/c)\,|\mathrm{sinc}(\pi fL/c)|$. Figure 25 is a plot of $|\mathcal{T}_{{\vec {u}}}(f,0)|$ versus frequency on a logarithmic frequency scale.

If we choose instead to measure the fractional Doppler frequency shift of the incoming photons, then we need to differentiate the timing response with respect to *t* as indicated in (5.1). This simply pulls-down a factor of $i2\pi f$ from the exponential in $\Delta T(t)$, leading to

5.20 $$\begin{aligned} R^{ab}_\mathrm{doppler}(f,\hat{n}) = i 2\pi f\, R^{ab}_\mathrm{timing}(f,\hat{n}). \end{aligned}$$

Thus, the frequency-dependence of the Doppler frequency response is $i2\pi f$ times the timing transfer function $\mathcal{T}_{{\vec {u}}}(f,\hat{n}\cdot \hat{u})$. All of the above remarks are relevant for pulsar timing and *one-way* spacecraft Doppler tracking.

**Fig. 26:**
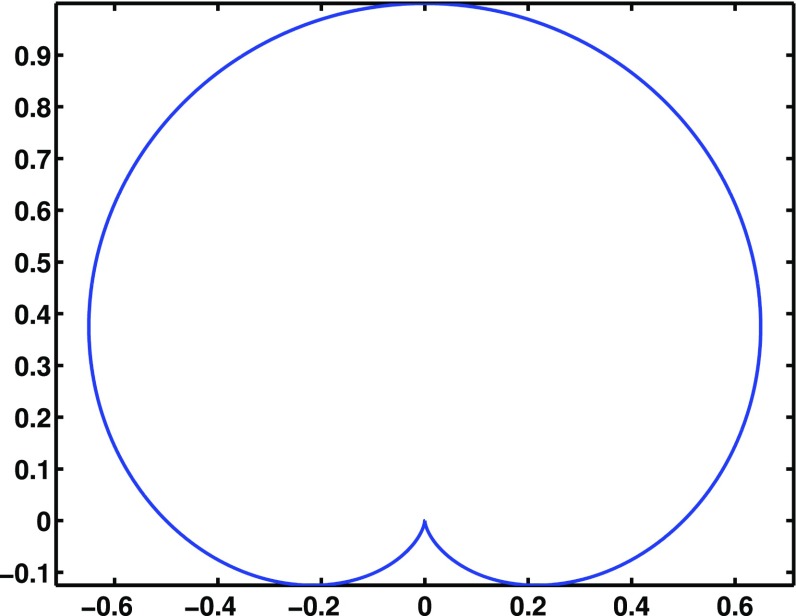
Antenna pattern for unpolarized gravitational waves for a one-way tracking Doppler frequency measurement with $\hat{u} =-\hat{z}$. The gravitational waves propagate toward the origin. The 3-d antenna pattern is axially symmetric around $\hat{u}$

In Fig. 26 we plot the antenna beam pattern (5.13) for unpolarized gravitational waves for a one-way tracking Doppler frequency measurement (e.g., pulsar timing) with $\hat{u}=-\hat{z}$. For this calculation, we chose $\vec {r}_2=0$ and ignored the exponential (i.e., ‘pulsar’) term in the timing transfer function, which yields

5.21 $$\begin{aligned} R^A_\mathrm{doppler}(f,\hat{n}) = \frac{1}{2} \frac{u^a u^b}{1+\hat{u}\cdot \hat{n}} e_{ab}^A(\hat{n}) \quad (\mathrm{Earth\ term\ only}), \end{aligned}$$

for the $A=+,\times$ polarization modes. Setting $\hat{u}=-\hat{z}$ and taking the gravitational waves to propagate inward (toward the origin), we find

5.22 $$\begin{aligned} \mathcal{R}_\mathrm{doppler}(\hat{n}) = \frac{1}{2}(1+\cos \theta ), \end{aligned}$$

which is axially symmetric around $\hat{u}$. The response is maximum when the photon and the gravitational wave both propagate in the same direction.

**Fig. 27:**
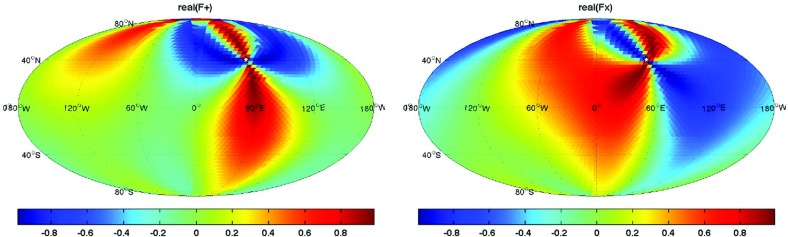
Mollweide projections of the response functions $R_\mathrm{doppler}^+(\hat{n})$, $R_\mathrm{doppler}^\times (\hat{n})$, for one-way tracking Doppler frequency measurements corresponding to a pulsar located in the direction of the white star $(\theta ,\phi )=(50^\circ , 60^\circ )$. The imaginary parts of both response functions are identically zero, so are not shown above

Figure 27 shows plots of the real parts of the individual polarization basis response functions (5.21), represented as color bar plots on a Mollweide projection of the sky. For this plot we chose the pulsar to be located in the direction $(\theta ,\phi ) = (50^\circ ,60^\circ )$. (The direction $\hat{p}$ to the pulsar is given by $\hat{p} = -\hat{u}$). The imaginary parts of both response functions are identically zero, so are not shown in the figure.

Making the same approximations as above, we can also calculate the corresponding Doppler-frequency response functions for the gradient and curl tensor spherical harmonic components $\{a^G_{lm}(f),a^C_{lm}(f)\}$ by performing the integration in (5.12). As shown in Gair et al. (2014), this leads to^11^

5.23 $$\begin{aligned} R^G_{(lm)}(f) = 2\pi \,{}^{(2)}\!N_l Y_{lm}(\hat{p}), \quad R^C_{(lm)}(f) = 0, \end{aligned}$$

where ${}^{(2)}\!N_l$ is given by (2.8) and $\hat{p} = -\hat{u}$ is the direction on the sky to a pulsar. Note, somewhat surprisingly, that the curl response is *identically zero*. We will discuss the consequences of this result in more detail in Sect. 7.5.6, in the context of phase-coherent mapping of anisotropic gravitational-wave backgrounds.

#### Two-way tracking

To calculate $\Delta T(t)$ for *two-way* spacecraft Doppler tracking, we need to generalize the calculation of the previous subsection to include a return trip of the photon from $\vec {r}_2$ back to $\vec {r}_1$. This can be done by simply summing the expressions for the one-way timing residuals:

5.24 $$\begin{aligned} \Delta T(t) = \Delta T_{12}(t-L/c) + \Delta T_{21}(t) \end{aligned}$$

where the subscripts on the $\Delta T$’s on the right-hand side of the above equation indicate the direction of one-way photon propagation (e.g., 12 indicates photon propagation from test mass 1 to test mass 2), and the arguments of $\Delta T_{12}$ and $\Delta T_{21}$ indicate when the photon arrived at test mass 2 and test mass 1, respectively. Doing this calculation leads to the following expression for the timing residual:

5.25 $$\begin{aligned} {\Delta T(t)}&= \int _{-\infty }^\infty df \frac{1}{2} u^a u^b h_{ab}(f,\hat{n})\, \frac{1}{i 2\pi f}\, \bigg [ \frac{1}{1-\hat{n}\cdot \hat{u}}\, e^{i 2\pi f(t+\hat{n}\cdot \vec {r}_1/c)} \nonumber \\&\quad -\frac{2\hat{n}\cdot \hat{u}}{1-(\hat{n}\cdot \hat{u})^2}\, e^{i 2\pi f(t-L/c+\hat{n}\cdot \vec {r}_2/c)} -\frac{1}{1+\hat{n}\cdot \hat{u}}\, e^{i 2\pi f(t-2L/c+\hat{n}\cdot \vec {r}_1/c)} \bigg ],\nonumber \\ \end{aligned}$$

which has *three* terms corresponding to the final reception of the photon at $\vec {r}_1$ at time *t*, the reflection of the photon at $\vec {r}_2$ at time $t-L/c$, and the emission of the photon at $\vec {r}_1$ at time $t-2L/c$. The timing response function is given by

5.26 $$\begin{aligned} R^{ab}_\mathrm{timing}(f,\hat{n}) =\frac{1}{2}u^a u^b\, \mathcal{T}_{{{\vec {u}}},\mathrm{rt}}(f,\hat{n}\cdot \hat{u}) e^{i2\pi f\hat{n}\cdot \mathbf {r}_1/c}, \end{aligned}$$

where

5.27 $$\begin{aligned} \mathcal{T}_{{{\vec {u}}},\mathrm{rt}}(f,\hat{n}\cdot \hat{u})\equiv & {} \frac{L}{c} e^{-\frac{i 2\pi f L}{c}}\, \bigg [e^{-\frac{i\pi f L}{c}(1-{\hat{n}}\cdot {\hat{u}})}\, \mathrm{sinc}\left( \frac{\pi f L}{c}[1+{\hat{n}}\cdot {\hat{u}}]\right) \nonumber \\&+\,e^{\frac{i\pi f L}{c}(1+{\hat{n}}\cdot {\hat{u}})}\, \mathrm{sinc}\left( \frac{\pi f L}{c}[1-{\hat{n}}\cdot {\hat{u}}]\right) \bigg ] \end{aligned}$$

is the timing transfer function for *two-way* (or roundtrip) photon propagation along ${\vec {u}}$ and back. For normal incidence, the magnitude of the timing transfer function is given by $|\mathcal{T}_{{{\vec {u}}},\mathrm{rt}}(f,0)|= (2L/c)|\mathrm{sinc}(2\pi fL/c)|$, which is identical to the expression for one-way tracking with *L* / *c* replaced by 2*L* / *c*. We also note that if we choose the origin of coordinates to be at $\vec {r}_1$ (which we can always do for a single detector), and if the frequency *f* is such that $fL/c\ll 1$, then the timing response simplifies to

5.28 $$\begin{aligned} R^{ab}_\mathrm{timing}(f,\hat{n}) = u^a u^b\,\frac{L}{c} \quad (\mathrm{for}\ fL/c\ll 1). \end{aligned}$$

We will use the terminology *small-antenna limit* (instead of *long-wavelength limit*) for this type of limit, since it avoids an ambiguity that might arise if we want to compare three or more length scales. For example, if we have two detectors that are physically separated and the wavelength of a gravitational wave is *large* compared to the size of each detector but *small* compared to the separation of the detectors, we would be in the long-wavelength limit with respect to detector size but in the short-wavelength limit with respect to detector separation. (This is actually the case for the current network of ground-based interferometers). The terminology *small-antenna, large-separation limit* is more appropriate for this case.

**Fig. 28:**
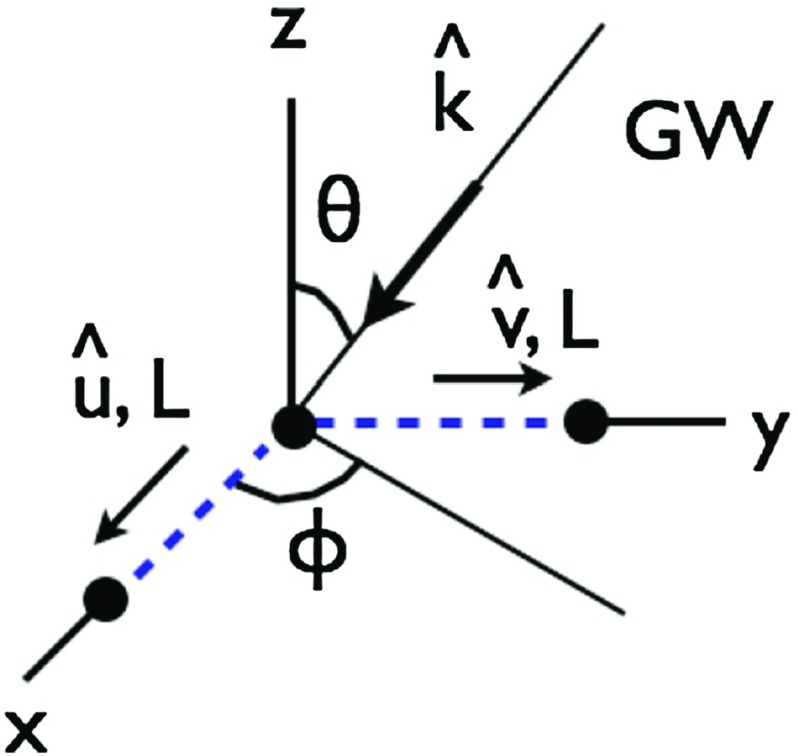
Geometry for calculating the difference in the round-trip light-travel times in the two arms of a Michelson interferometer: $\hat{k}=-\hat{n}$ is the direction of propagation for a plane gravitational wave; $\hat{u}$ and $\hat{v}$ are unit vectors that point from the vertex of the interferometer (e.g., the beam splitter) to the two end masses; and *L* denotes the lengths of each of the arms in the absence of a gravitational wave

#### Michelson interferometer

For an equal-arm Michelson interferometer, the timing residual that we calculate is the difference in the round-trip light-travel times down and back each of the arms. (See Fig. 28). If we let ${\vec {u}}$ and ${\vec {v}}$ denote the vectors pointing from e.g., the beam splitter to the two end mirrors for LIGO, or from one spacecraft to the other two spacecraft for LISA, then^12^

5.29 $$\begin{aligned} \Delta T(t)\equiv T_{{{\vec {u}}},\mathrm{rt}}(t)- T_{{{\vec {v}}},\mathrm{rt}}(t) = \Delta T_{{{\vec {u}}},\mathrm{rt}}(t)- \Delta T_{{{\vec {v}}},\mathrm{rt}}(t), \end{aligned}$$

where the last equality is valid for an equal-arm interferometer. But we just calculated these single-arm round-trip $\Delta T$’s in the previous section. Thus, the timing response of an equal-arm Michelson is simply

5.30 $$\begin{aligned} R^{ab}_\mathrm{timing}(f,\hat{n})&=\frac{1}{2} \left[ u^a u^b\,\mathcal{T}_{{{\vec {u}}},\mathrm{rt}}(f,\hat{n}\cdot \hat{u})- v^a v^b\,\mathcal{T}_{{{\vec {v}}},\mathrm{rt}}(f,\hat{n}\cdot \hat{v}) \right] , \end{aligned}$$

where we have chosen the origin of coordinates to be at the vertex of the interferometer. The phase and strain responses of a Michelson are related to the timing response by constant multiplicative factors, cf. (5.3) and (5.4), so that

5.31 $$\begin{aligned} \begin{aligned} R^{ab}_\mathrm{phase}(f,\hat{n})&= 2\pi \nu _0 R^{ab}_\mathrm{timing}(f,\hat{n}), \\ R^{ab}_\mathrm{strain}(f,\hat{n})&= R^{ab}_\mathrm{timing}(f,\hat{n})/(2L/c), \end{aligned} \end{aligned}$$

where $\nu _0$ is the frequency of the laser. Note that in the small-antenna limit, which is valid for the LIGO detectors below a few kHz, the strain response is given by

5.32 $$\begin{aligned} R^{ab}_\mathrm{strain}(f,\hat{n}) = \frac{1}{2}(u^a u^b-v^a v^b)\, \quad (\mathrm{for}\ fL/c\ll 1). \end{aligned}$$

**Fig. 29:**
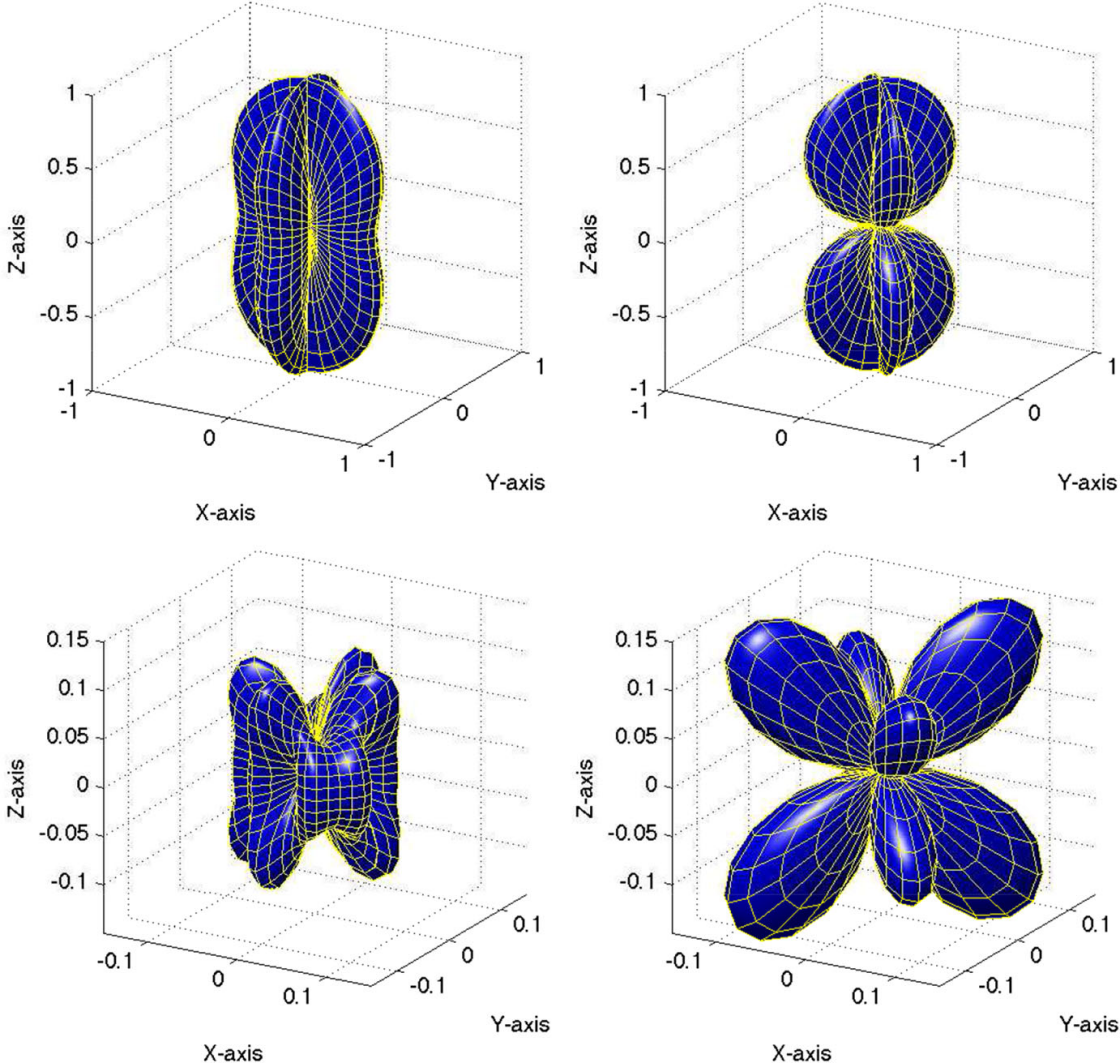
Antenna patterns for Michelson interferometer strain response $|R^+_\mathrm{strain}|$ and $|R^\times _\mathrm{strain}|$ evaluated in the small-antenna limit, $f=0$ (*top two plots*) and at the free-spectral range frequency, $f=c/(2L)$ (*bottom two plots*). The interferometer arms point in the $\hat{x}$ and $\hat{y}$ directions. Note the change in the scale of the axes between the top and bottom two plots

**Fig. 30:**
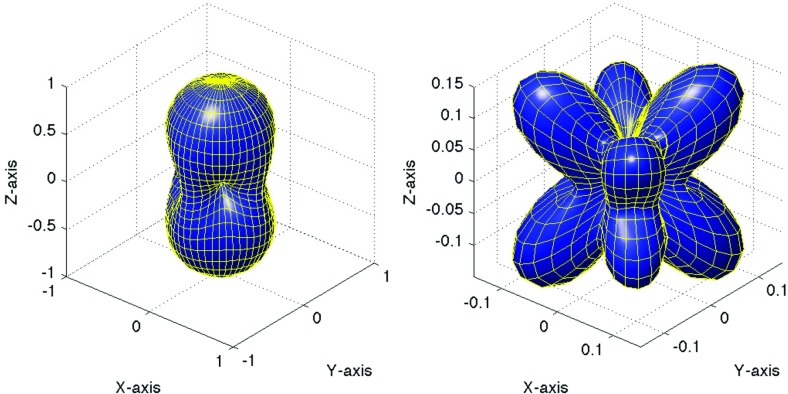
Antenna pattern for Michelson interferometer strain response to unpolarized gravitational waves evaluated in the small-antenna limit, $f=0$ (*left plot*) and at the free-spectral range frequency, $f=c/(2L)$ (*right plot*). The interferometer arms point in the $\hat{x}$ and $\hat{y}$ directions. Note the change in the scale of the axes between the two plots

**Fig. 31:**
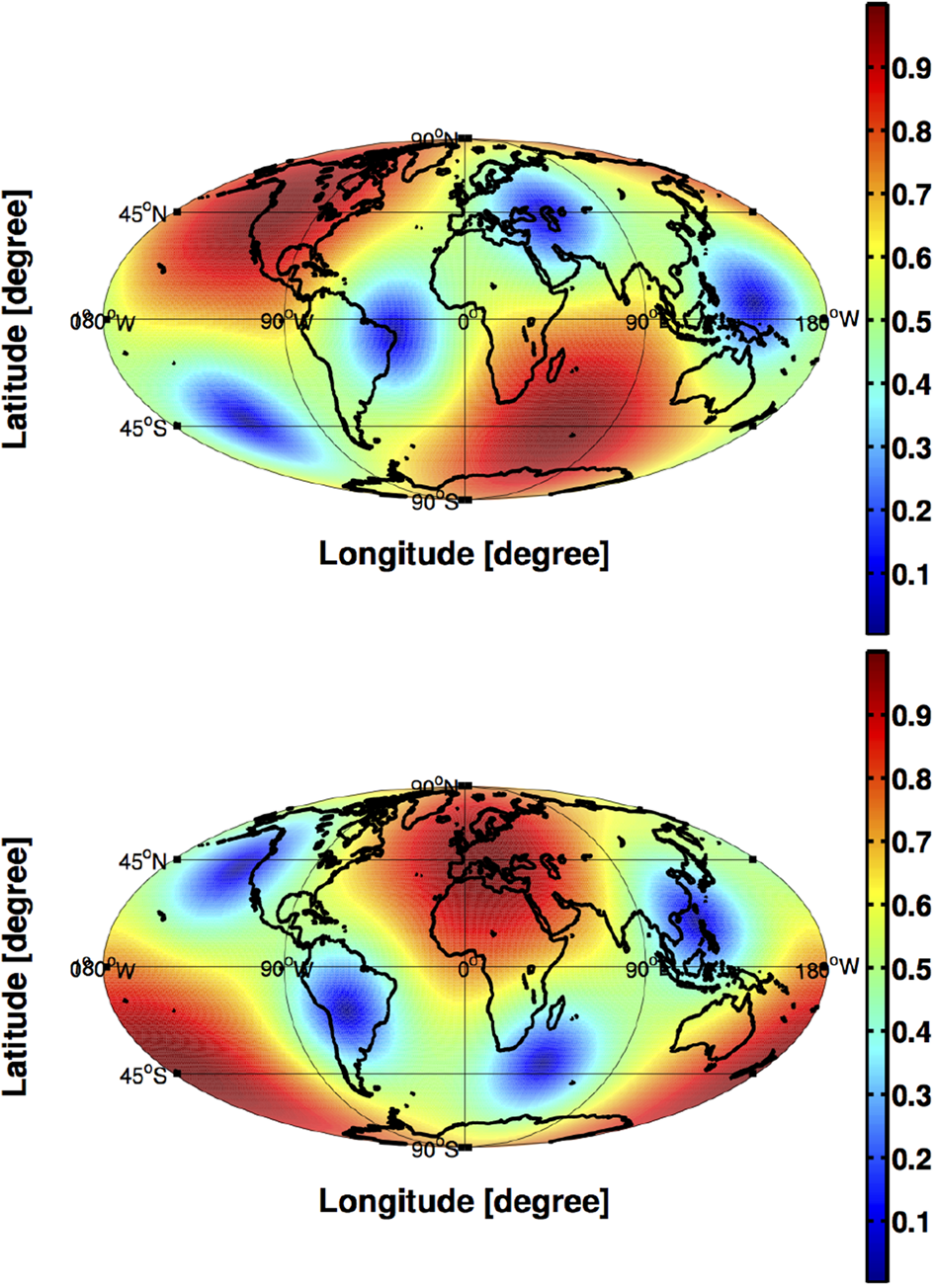
Antenna patterns for the strain response to unpolarized gravitational waves for the LIGO Hanford (*top panel*) and Virgo (*bottom panel*) interferometers evaluated in the small-antenna limit. The antenna patterns are represented as *colorbar* plots on a Mollweide projection of the Earth. Note that the maxima of the antenna patterns (the centers of the *red regions*) are directly *above* (and *below*) the location of the two interferometers—in Hanford, WA and Cascina, Italy, respectively. The *blue regions* correspond to the minima of the antenna patterns—i.e., the ‘dimples’ in the *left panel* plot of Fig. 30

Plots of the antenna patterns for the strain response to $A=+,\times$ polarized gravitational waves are given in Fig. 29, for both the small-antenna limit (where we simply set $f=0$) and at the *free-spectral range* of the interferometer, $f = f_\mathrm{fsr}\equiv c/(2L)$. Similar plots of the antenna patterns for unpolarized gravitational waves are given in Fig. 30. In Fig. 31 we show colorbar plots of the antenna patterns for the strain response to unpolarized gravitational waves for the LIGO Hanford and Virgo interferometers (located in Hanford, WA and Cascina, Italy, respectively), again evaluated in the small-antenna limit.

We can also calculate the strain response of an interferometer to the gradient and curl tensor spherical harmonic components $\{a^G_{(lm)}(f), a^C_{(lm)}(f)\}$ by performing the integration in (5.12). As shown in Appendix E of Gair et al. (2014), this leads to

5.33 $$\begin{aligned} R^G_{(lm)}(f) = \delta _{l2}\frac{4\pi }{5}\sqrt{\frac{1}{3}} \left[ Y_{2m}(\hat{u}) - Y_{2m}(\hat{v})\right] , \quad R^C_{(lm)}(f) = 0, \end{aligned}$$

for an interferometer in the small-antenna limit, where the vertex is at the origin of coordinates, and $\hat{u}$, $\hat{v}$ are unit vectors pointing in the direction of the interferometer arms. Similar to (5.23) for pulsar timing, the curl response is again identically zero. We will discuss the consequences of this result in more detail in Sect. 7.5.7, in the context of phase-coherent mapping of anisotropic gravitational-wave backgrounds.

### Overlap functions

As mentioned in Sect. 4, a stochastic gravitational-wave background manifests itself as a non-vanishing correlation between the data taken by two or more detectors. This correlation differs, in general, from that due to instrumental noise, allowing us to distinguish between a stochastic gravitational-wave signal and other noise sources. In this section, we calculate the expected correlation due to a gravitational-wave background, allowing for non-trivial detector response functions and non-trivial detector geometry. Interested readers can find more details in Hellings and Downs (1983), Christensen (1990, 1992), Flanagan (1993), and Finn et al. (2009).

#### Definition

Let $d_I$ and $d_J$ denote the data taken by two detectors labeled by *I* and *J*. In the presence of a gravitational wave, these data will have the form

5.34 $$\begin{aligned} \begin{aligned} d_I&= h_I + n_I, \\ d_J&= h_J + n_J, \end{aligned} \end{aligned}$$

where $h_{I,J}$ denote the response of detectors *I*, *J* to the gravitational wave, and $n_{I,J}$ denote the contribution from instrumental noise. If the instrumental noise in the two detectors are uncorrelated with one another, it follows that the expected correlation of the data is just the expected correlation of the detector responses, $\langle d_I d_J\rangle = \langle h_I h_J\rangle$. If we also assume that the gravitational wave is due to a stationary, Gaussian, isotropic, and unpolarized stochastic background, then

5.35 $$\begin{aligned} \langle h_I(t) h_J(t')\rangle =\frac{1}{2}\int _{-\infty }^\infty df\> e^{i 2\pi f(t-t')}\Gamma _{IJ}(f)S_h(f), \end{aligned}$$

where $S_h(f)$ is the one-sided strain power spectral density of the gravitational-wave background, computed from the expectation values of the Fourier components of the metric perturbations (2.14), and

5.36 $$\begin{aligned} \Gamma _{IJ}(f) \equiv \frac{1}{8\pi } \int d^2\Omega _{\hat{n}} \sum _A R_I^A(f,\hat{n})R_J^{A}{}^*(f,\hat{n}) \end{aligned}$$

is the so-called *overlap function* for the two detectors *I*, *J* written in terms of the polarization-basis response function $R^A_{I,J}(f,\hat{n})$,^13^ where $A=\{+,\times \}$. In terms of the tensor spherical harmonic-basis response functions $R^P_{I,J(lm)}(f)$, we would have

5.37 $$\begin{aligned} \Gamma _{IJ}(f) = \frac{1}{8\pi } \sum _{(lm)} \sum _P R_{I(lm)}^P(f)R_{J(lm)}^{P*}(f), \end{aligned}$$

where $P=\{G,C\}$ for the gradient and curl tensor spherical harmonic components.

#### Interpretation

The overlap function $\Gamma _{IJ}(f)$ quantifies the reduction in sensitivity of the cross-correlation to a stochastic gravitational-wave background due to the non-trivial response of the detectors and their separation and orientation relative to one another. This meaning of the overlap function is most easily seen in the frequency domain, where (5.35) becomes

5.38 $$\begin{aligned} \langle \tilde{h}_I(f) \tilde{h}_J^*(f')\rangle = \frac{1}{2}\delta (f-f')\, \Gamma _{IJ}(f)S_h(f). \end{aligned}$$

This implies

5.39 $$\begin{aligned} \tilde{C}_{h_I h_J}(f) = \Gamma _{IJ}(f) S_h(f), \end{aligned}$$

where $\tilde{C}_{h_I h_J}(f)$ is the (one-sided) cross-spectrum of the response in the two detectors. Thus, $\Gamma _{IJ}(f)$ can be interpreted as the transfer function between gravitational-wave strain power $S_h(f)$ and detector response cross-power $\tilde{C}_{h_I h_J}(f)$.

Expression (5.36) for the overlap function involves four length scales: the lengths of the two detectors, $L_I$ and $L_J$, which appear in the response functions $R^A_{I,J}(f,\hat{n})$; the separation of the detectors, $s\equiv |{\vec {x}}_I-{\vec {x}}_J|$, which appears in the exponential factor; and the wavelength of the gravitational waves, $\lambda = c/f$. In general, one has to evaluate the integral in (5.36) *numerically*, due to the non-trivial frequency dependence of the response functions. However, as we shall see in Sect. 5.4, in certain limiting cases of the ratio of these length scales, we can do the integral *analytically* and obtain relatively simple expressions for the overlap function in terms of spherical Bessel or trigonometric functions. This is the case for ground-based interferometers, which operate in the *small-antenna limit*—i.e., $fL/c\ll 1$ for both detectors, even though the separation can be large compared to the wavelength, $fs/c \gtrsim 1$. It is also the case for pulsar timing arrays, which operate in the *large-antenna, small-separation limit*, since $fL/c\gg 1$ for each pulsar and $fs/c\ll 1$ for different radio receivers on Earth. (The Earth effectively resides at the solar system barycenter relative to the wavelength of the gravitational waves relevant for pulsar timing).

#### Normalization

It is often convenient to define a *normalized* overlap function $\gamma _{IJ}(f)\propto \Gamma _{IJ}(f)$ by requiring that $\gamma _{IJ}(0)=1$ for two detectors that are co-located and co-aligned. For the strain response of two identical equal-arm Michelson interferometers, this leads to the relation

5.40 $$\begin{aligned} \gamma _{IJ}(f) = \frac{5}{\sin ^2\beta }\,\Gamma _{IJ}(f) \end{aligned}$$

where $\beta$ is the opening angle between the two arms ($\pi /2$ for LIGO and $\pi /3$ for LISA).

#### Auto-correlated response

To obtain the *auto-correlated* response of a *single* detector, we can simply set $I=J$ in the previous expressions. This means that the gravitational-wave strain power $S_h(f)$ and the detector response power $P_{h_I}(f)$ in detector *I* are related by

5.41 $$\begin{aligned} P_{h_I}(f) = \Gamma _{II}(f) S_h(f), \end{aligned}$$

where

5.42 $$\begin{aligned} \Gamma _{II}(f) =\frac{1}{8\pi } \int d^2\Omega _{\hat{n}} \sum _A |R_I^A(f,\hat{n})|^2. \end{aligned}$$

**Fig. 32:**
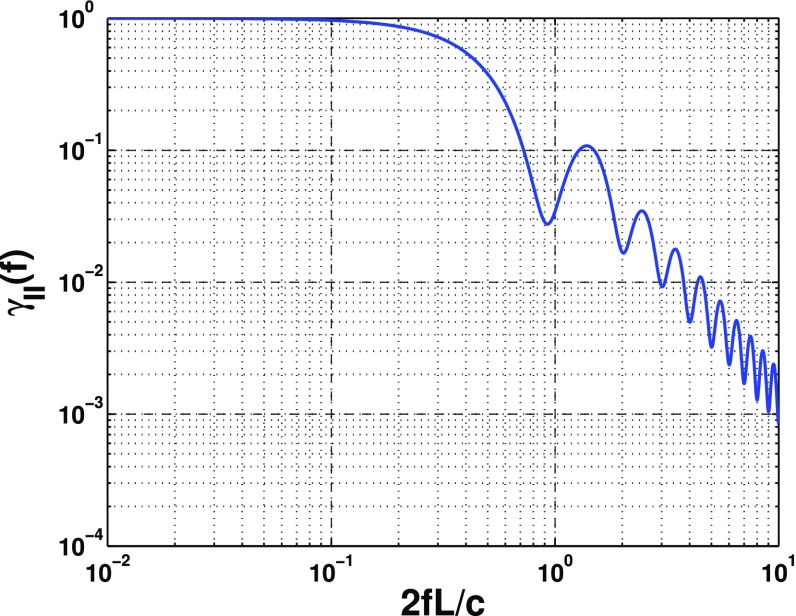
A plot of the normalized transfer function $\gamma _{II}(f)$ for the strain response of an equal-arm Michelson interferometer. The dips in the transfer function occur around integer multiples of *c* / (2*L*)

**Fig. 33:**
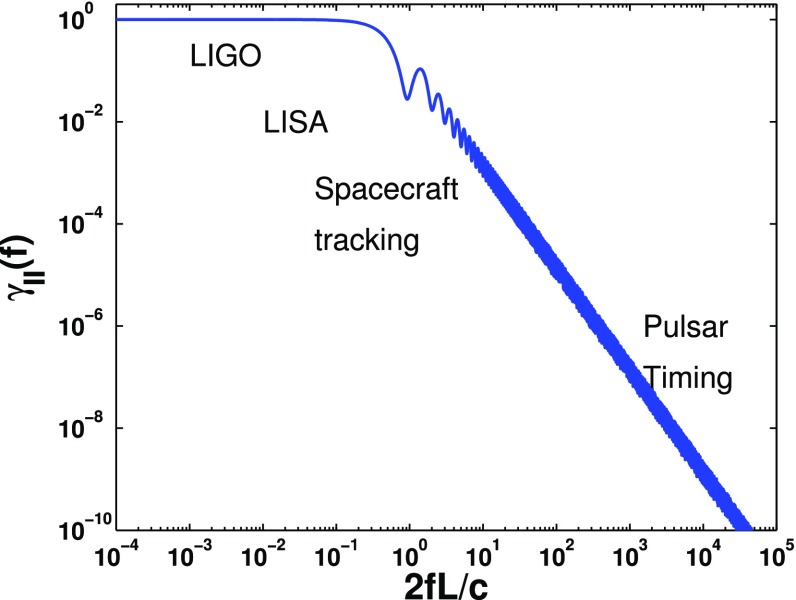
An extension of Fig. 32 to lower and higher frequencies, and plotted on a log–log scale. The position of the labels show the relative location of the frequency bands for gravitational-wave searches using ground-based interferometers like LIGO, space-based interferometers like LISA, spacecraft Doppler tracking, and pulsar timing arrays, expressed in units of *c* / (2*L*). See also Table 5 for more details

Note that $\Gamma _{II}(f)$ is just the square of the antenna pattern for the response to unpolarized gravitational waves *integrated over the whole sky*. A plot of the normalized transfer function $\gamma _{II}(f)$ for the strain response of an equal-arm Michelson interferometer is shown in Fig. 32. Compared to Fig. 25 for the timing transfer function $|\mathcal{T}_{{\vec {u}}}(f,0)|$ for one-way photon propagation evaluated at normal incidence of the gravitational wave, we see that the relevant frequency scale for an equal-arm Michelson is *c* / (2*L*) (as opposed to *c* / *L*) due to the round-trip motion of the photons. Also, the hard nulls in Fig. 25 have been softened into *dips* due to averaging of the waves over the whole sky. The high-frequency ‘bumps’ for $\gamma _{II}(f)$ are lower than those for $|\mathcal{T}_{{\vec {u}}}(f,0)|$ due to the squaring of $|R^A_I(f,\hat{n})|$ which enters into the definition of $\Gamma _{II}(f)$ (and $\gamma _{II}(f)$). Figure 33 is an extended version of Fig. 32, with the appropriate frequency ranges for ground-based interferometers (like LIGO), space-based interferometers (like LISA), spacecraft Doppler tracking, and pulsar timing searches indicated on the plot. See also Table 5 for more details.

### Examples of overlap functions

#### LHO-LLO overlap function

As mentioned above, Earth-based interferometers like LIGO operate in the small-antenna limit where $fL/c\ll 1$. This implies that the associated response functions are well-approximated by the expression in (5.32). If we denote the unit vectors along the two arms of one Earth-based interferometer by $\hat{u}_1$ and $\hat{v}_1$, and the corresponding unit vectors of a second Earth-based interferometer by $\hat{u}_2$ and $\hat{v}_2$, then the strain responses in the two interferometers are simply

5.43 $$\begin{aligned} \begin{aligned} R^A_{1,\mathrm{strain}}(f,\hat{n})&\simeq D_1^{ab}\,e^A_{ab}(\hat{n}) e^{i2\pi f\hat{n}\cdot {\vec {x}}_1/c}, \\ R^A_{2,\mathrm{strain}}(f,\hat{n})&\simeq D_2^{ab}\,e^A_{ab}(\hat{n}) e^{i2\pi f\hat{n}\cdot {\vec {x}}_2/c}, \end{aligned} \end{aligned}$$

where

5.44 $$\begin{aligned} D_1^{ab} \equiv \frac{1}{2}\left( u_1^a u_1^b-v_1^a v_1^b\right) , \quad D_2^{ab} \equiv \frac{1}{2}\left( u_2^a u_2^b-v_2^a v_2^b\right) , \end{aligned}$$

and ${\vec {x}}_1$ and ${\vec {x}}_2$ denote the vertices of the two interferometers. The tensors $D_1^{ab}$, $D_2^{ab}$ defined above are called *detector tensors*; they are symmetric and trace-free with respect to their *ab* indices. In terms of the detector tensors, the overlap function becomes

5.45 $$\begin{aligned} \Gamma _{12}(f)=D_1^{ab} D_2^{cd} \Gamma _{abcd}(\Delta {\vec {x}}), \end{aligned}$$

where

5.46 $$\begin{aligned} \Gamma _{abcd}(\Delta {\vec {x}}) \equiv \int d^2\Omega _{\hat{n}}\sum _A e_{ab}^A(\hat{n})e_{cd}^A(\hat{n})\, e^{-i2\pi f\hat{n}\cdot \Delta {\vec {x}}/c} \end{aligned}$$

and $\Delta {\vec {x}}\equiv {\vec {x}}_2-{\vec {x}}_1$ is the separation vector connecting the two vertices. We will also define:

5.47 $$\begin{aligned} \alpha \equiv 2\pi fs/c, \quad s\equiv |\Delta {\vec {x}}|, \quad \hat{s}\equiv \Delta {\vec {x}}/s. \end{aligned}$$

Thus, in the small-antenna limit, the orientation-dependence of the overlap function $\Gamma _{12}(f)$ is encoded in the detector tensors $D_1^{ab}$, $D_2^{ab}$, while the separation-dependence is encoded in $\Gamma _{abcd}(\Delta {\vec {x}})$.

Note that $\Gamma _{abcd}$ is a tensor which is symmetric under the interchanges $a\leftrightarrow b$, $c\leftrightarrow d$, and $ab\leftrightarrow cd$; it is also trace-free with respect to the *ab* and *cd* index pairs. The most general expression that we construct for $\Gamma _{abcd}(\Delta {\vec {x}})$ given $\delta _{ab}$, $s_a$, and its symmetry properties is:

5.48 $$\begin{aligned} \Gamma _{abcd}(\Delta {\vec {x}})= & {} A(\alpha )\delta _{ab}\delta _{cd} + B(\alpha )(\delta _{ac}\delta _{bd} + \delta _{bc}\delta _{ad}) + C(\alpha )(\delta _{ab}s_c s_d + \delta _{cd}s_a s_b) \nonumber \\&+\,D(\alpha )(\delta _{ac}s_b s_d + \delta _{ad} s_b s_c + \delta _{bc}s_a s_d + \delta _{bd}s_a s_c) + E(\alpha ) s_a s_b s_c s_d. \nonumber \\ \end{aligned}$$

By contracting the above expression with tensors of the form $\delta ^{ab}\delta ^{cd}$, $(\delta ^{ac}\delta ^{bd} + \delta ^{bc}\delta ^{ad})$, $\ldots$, $s^a s^b s^c s^d$, we obtain a linear system of equations for $A, B,\ldots , E$, which we can solve in terms of scalar integrals involving contractions of the products of the polarization tensors, $e^A_{ab}(\hat{n})e^A_{cd}(\hat{n})$, with various combinations of $\delta ^{ab}$ and $s^a$. As shown in Flanagan (1993) and Allen and Romano (1999), these integrals can be done *analytically*, leading to

5.49 $$\begin{aligned} \left[ \begin{array}{c} A(\alpha )\\ B(\alpha )\\ C(\alpha )\\ D(\alpha )\\ E(\alpha )\\ \end{array} \right] = \frac{1}{2\alpha ^2} \left[ \begin{array}{rrr} -5\alpha ^2 &{} 10\alpha &{} 5 \\ 5\alpha ^2 &{} -10\alpha &{} 5 \\ 5\alpha ^2 &{} -10\alpha &{} -25 \\ -5\alpha ^2 &{} 20\alpha &{} -25 \\ 5\alpha ^2 &{} -50\alpha &{} 175 \\ \end{array} \right] \left[ \begin{array}{c} j_0(\alpha )\\ j_1(\alpha )\\ j_2(\alpha )\\ \end{array} \right] , \end{aligned}$$

where $j_0(\alpha )$, $j_1(\alpha )$, and $j_2(\alpha )$ are the standard spherical Bessel functions (Abramowitz and Stegun 1972). With these explicit expressions for $A, B,\ldots , E$ in hand, all that is left to do is to contract the right-hand side of (5.48) with $D_1^{ab} D_2^{cd}$ to obtain $\Gamma _{12}(f)$. If we only assume that the detector tensors are symmetric,^14^ then all terms contribute (Coughlin and Harms 2014):

5.50 $$\begin{aligned} \begin{aligned} \Gamma _{12}(f)&=A(\alpha )\mathrm {Tr}\,(D_1)\mathrm {Tr}\,(D_2) +2B(\alpha )D_1^{ab}D_{2ab} \\&\quad +C(\alpha )\left[ \mathrm {Tr}\,(D_1)D_2^{ab} + \mathrm {Tr}\,(D_2)D_1^{ab}\right] s_a s_b \\&\quad +4D(\alpha ) D_1^{ab} D_{2a}{}^c s_b s_c +E(\alpha )D_1^{ab} D_2^{cd} s_a s_b s_c s_d. \end{aligned} \end{aligned}$$

For symmetric, *trace-free* detector tensors, as is the case for ground-based interferometers, there is no contribution from the *A* and *C* terms. Thus, in the small-antenna limit, the overlap function for the strain response of two equal-arm Michelson interferometers can be written as a sum of the first three spherical Bessel functions with coefficients that depend on the product of the frequency and separation of the two detectors. (The analytic expression for the overlap function can also be derived using (5.37), which involves the tensor spherical harmonic response functions. A detailed derivation using these response functions is given in Romano et al., 2015).

**Fig. 34:**
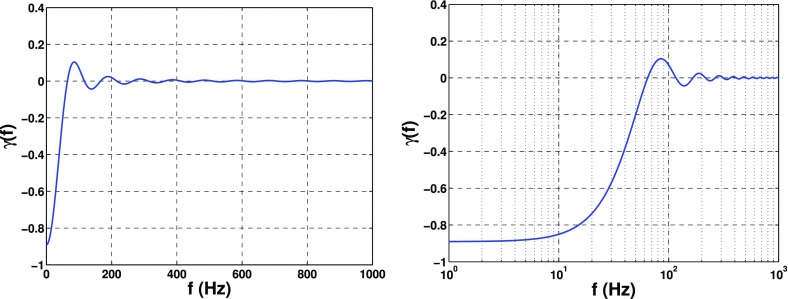
Overlap function for the LIGO Hanford-LIGO Livinston cross-correlation in the small-antenna limit. *Left panel* linear frequency scale. *Right panel* logarithmic frequency scale

Figure 34 is a plot of the normalized overlap function for the strain response of the 4-km LIGO interferometers in Hanford, WA and Livingston, LA. There are several things to note about the plot: (i) The overlap function is negative as $f\rightarrow 0$. This is because the arms of the Hanford and Livingston interferometers are rotated by $90^\circ$ with respect to one another. (ii) The magnitude of the overlap function at $f=0$ is less than unity—i.e., $|\gamma _{HL}(0)|=0.89$, even though the overlap function was normalized. This is because the planes of the Hanford and Livingston interferometers are not identical; these two detectors are separated by $27.2^\circ$ as seen from the center of the Earth. (iii) The first zero of the overlap function occurs just above 60 Hz. This is roughly equal to $c/(2s)=50~\mathrm{Hz}$, where $s= 3000~\mathrm{km}$ is the separation between the two interferometers. Note that $f=c/(2s)$ is the frequency of a gravitational wave that has a wavelength equal to twice the separation of the two sites. For lower frequencies, the two interferometers will be driven (on average) by the same positive (or negative) part of the incident gravitational wave. For slightly higher frequencies, one interferometer will be driven by the positive (or negative) part of the incident wave, while the other interferometer will be driven by the negative (or positive) part. The zeros of the overlap function correspond to the transitions between the in-phase and out-of-phase excitations of the two interferometers.

#### Big-bang observer overlap function

**Fig. 35:**
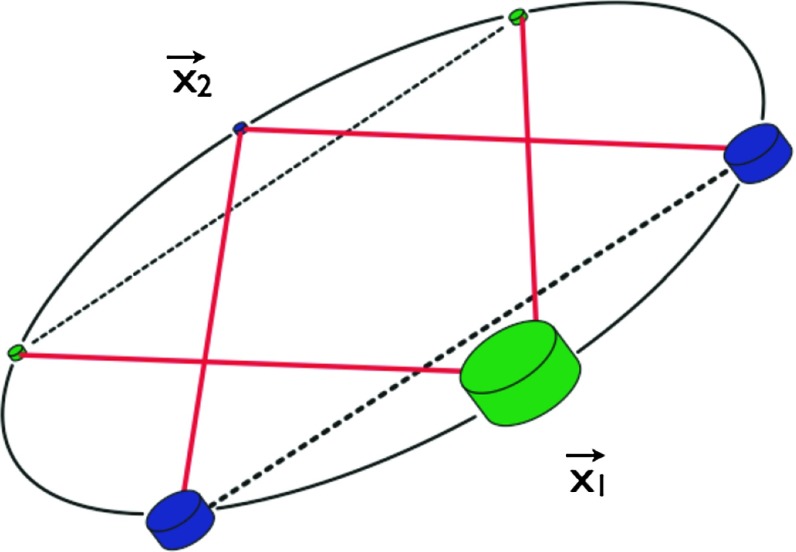
Hexagram configuration for the cross-correlation of two LISA-like detectors, relevant for the proposed Big-Bang Observer space mission. Spacecraft, which house lasers and freely-falling test masses, are located at each vertex of the hexagram. The vectors ${\vec {x}}_1$ and ${\vec {x}}_2$ denote the vertices of two equal-arm Michelson interferometers, with opening angle $\beta =60^\circ$. Image reproduced with permission from Cornish and Larson (2001), copyright by IOP

**Fig. 36:**
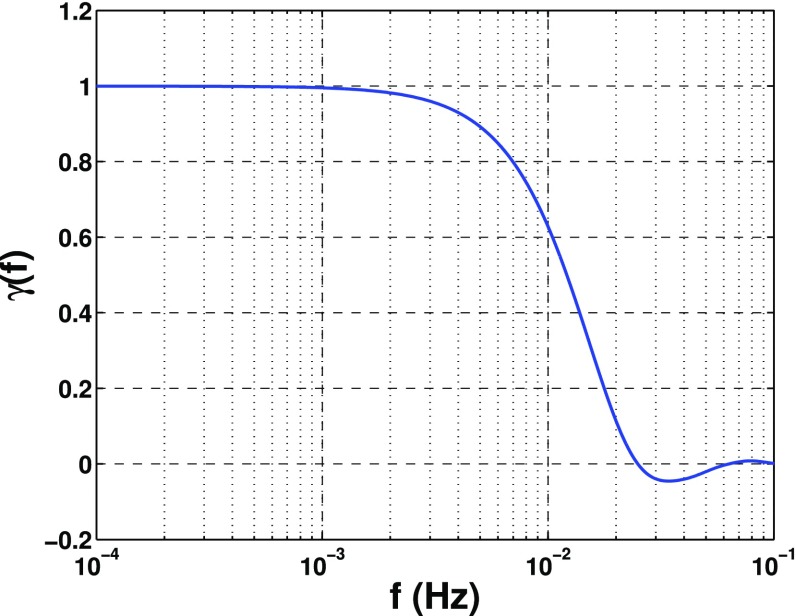
Plot of the normalized overlap function for strain response for the hexagram configuration shown in Fig. 35

As a second example, we consider the overlap function between two LISA-like constellations oriented in a hexagram (i.e., ‘six-pointed star’) configuration as shown in Fig. 35. This is one of the configurations being considered for the Big-Bang Observer (BBO), which is a proposed space mission designed to detect or put stringent limits on a cosmologically-generated gravitational-wave background (Phinney et al. 2004). The arm lengths of the two interferometers, with vertices ${\vec {x}}_1$ and ${\vec {x}}_2$, are taken to be $L=5\times 10^6~\mathrm{km}$. The opening angle for the two interferometers is $\beta = 60^\circ$. For this example, we calculate the normalized overlap function for strain response numerically, since the small-antenna limit is not valid for the high-frequency end of the sensitivity band. A plot of the normalized overlap function is given in Fig. 36.

#### Pulsar timing overlap function (Hellings and Downs curve)

As our final example, we consider the overlap function for timing residual measurements from an array of *N* pulsars, labeled by index $I=1,2,\ldots , N$. Each pulsar defines a one-way tracking beam detector with the position of pulsar *I* at $\vec {p}_{I}$ and the position of detector *I* (i.e., a radio receiver on Earth) by ${\vec {x}}_{I}$. For convenience, we will take the origin of coordinates to lie at the solar system barycenter. Since the diameter of the Earth (${\sim } 10^4~\mathrm{km}$) and its distance from the Sun (${\sim } 10^8~\mathrm{km}$) are both small compared to the wavelength of gravitational waves relevant for pulsar timing ($\lambda = c/f\sim 10^{13}~\mathrm{km}$), we can effectively set ${\vec {x}}_{I}\approx {\vec {x}}_J\approx {\vec {0}}$ in the argument of the exponential term that enters expression (5.36) for the overlap function. Thus,5.51where the unit vectors $\hat{u}_I$, $\hat{u}_J$ are defined by ${\vec {x}}_I= \vec {p}_I + L_I\hat{u}_I$, where $L_I$ is the distance to pulsar *I*. But since ${\vec {x}}_I\approx {\vec {0}}$, it follows that $\hat{u}_I$ and $\hat{u}_J$ are just unit vectors pointing from the location of pulsars *I* and *J* *toward* the solar system barycenter. For distinct pulsars ($I\ne J$), we can ignore the exponential terms in the square brackets, since $fL/c\gg 1$ for $L\sim 1~\mathrm{kpc}\ ({=}3\times 10^{16}~\mathrm{km})$ implies that $e^{-i 2\pi fL_I(1+\hat{n}\cdot \hat{u}_I)/c}$ and its product with the corresponding term for pulsar *J* are rapidly varying functions of $\hat{n}$ and do not contribute significantly when integrated over the whole sky (Hellings and Downs 1983; Anholm et al. 2009). (For a single pulsar ($I=J$), the product of the two exponential terms equals 1 and hence cannot be ignored). With these simplifications, the integral can be done analytically (Hellings and Downs 1983; Anholm et al. 2009; Jenet and Romano 2015). The result is

5.52 $$\begin{aligned} \Gamma _{IJ}(f) = \frac{1}{(2\pi f)^2}\, \frac{1}{3}\,\chi (\zeta _{IJ}), \end{aligned}$$

where

**Fig. 37:**
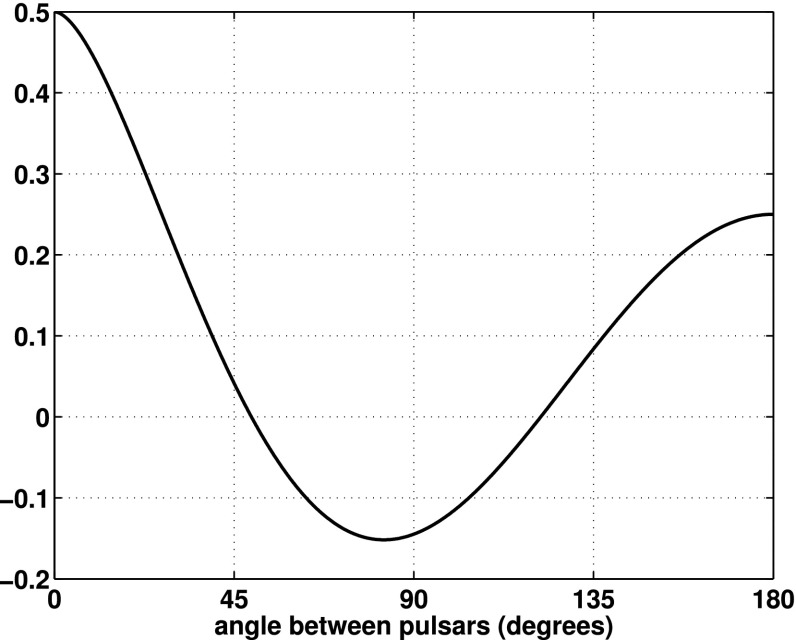
Plot of the Hellings and Downs curve as a function of the angular separation between two distinct pulsars

5.53 $$\begin{aligned} \chi (\zeta _{IJ})\equiv \frac{3}{2}\left( \frac{1-\cos \zeta _{IJ}}{2}\right) \ln \left( \frac{1-\cos \zeta _{IJ}}{2}\right) -\frac{1}{4} \left( \frac{1-\cos \zeta _{IJ}}{2}\right) +\frac{1}{2} +\frac{1}{2}\delta _{IJ}, \end{aligned}$$

and $\zeta _{IJ}$ is the angle between the two pulsars *I* and *J* relative to the solar system barycenter. (For Doppler frequency measurements, the overlap function is *independent* of frequency, $\Gamma _{IJ}=\chi (\zeta _{IJ})/3$). $\chi (\zeta )$ is the *Hellings and Downs* function (Hellings and Downs 1983); it depends only on the angular separation of a pair of pulsars. The normalization was chosen so that for a single pulsar, $\chi (0)=1$ (for two *distinct* pulsars occupying the same angular position on the sky, $\chi (0)=0.5$). A plot of the Hellings and Downs curve is given in Fig. 37.

**Fig. 38:**
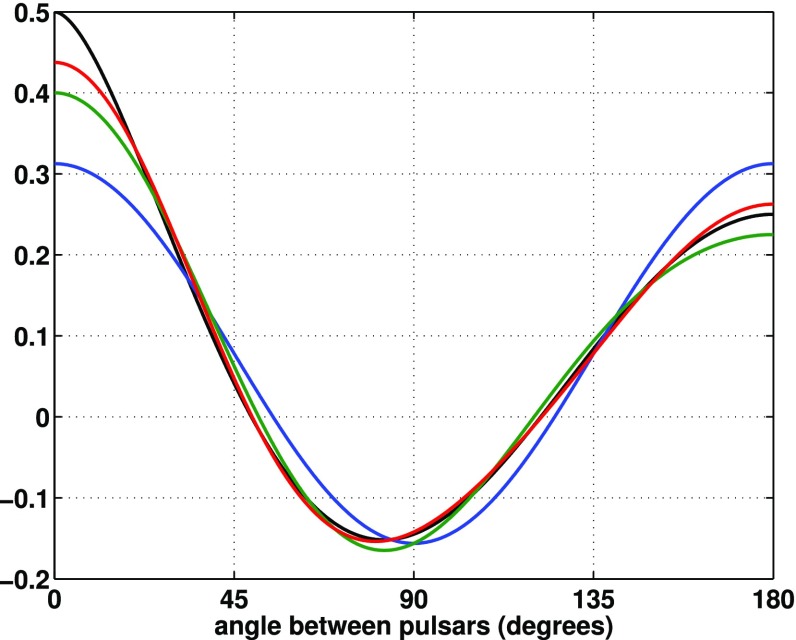
Comparison of the exact expression of the Hellings and Downs curve (*black*) with Legendre series approximations for different values of $l_\mathrm{max}$. The *blue*, *green*, and *red curves* correspond to $l_\mathrm{max} = 2$, 3, and 4, respectively

A couple of remarks are in order: (i) The Hellings and Downs curve is *independent* of frequency; it is a function of the *angle* $\zeta$ between different pulsar pairs. This contrasts with the overlap functions for the two LIGO interferometers and for BBO given in Figs. 34 and 36. These overlap functions were calculated for a fixed pair of detectors; they are functions instead of the *frequency* of the gravitational wave. (ii) The value of the Hellings and Downs function $\chi (\zeta _{IJ})$ for a pair of pulsars *I*, *J* can be written as a Legendre series in the cosine of the angle between the two pulsars. This follows immediately if one uses (5.37) for the overlap function and (5.23) for the pulsar timing response functions in the tensor spherical harmonic basis. As shown in Gair et al. (2014):

5.54 $$\begin{aligned} \chi (\zeta _{IJ}) = \frac{3}{4}\sum _{l=2}^\infty ({}^{(2)}\!N_l)^2 (2l+1)P_l(\hat{p}_I\cdot \hat{p}_j), \end{aligned}$$

where $\hat{p}_I$ and $\hat{p}_J$ are unit vectors that point in the directions to the two pulsars. A Legendre series expansion out to $l_\mathrm{max}=4$ (i.e., only three terms) gives very good agreement with the exact expression for the Hellings and Downs function, except for very small angular separations. This is illustrated in Fig. 38.

### Moving detectors

**Fig. 39:**
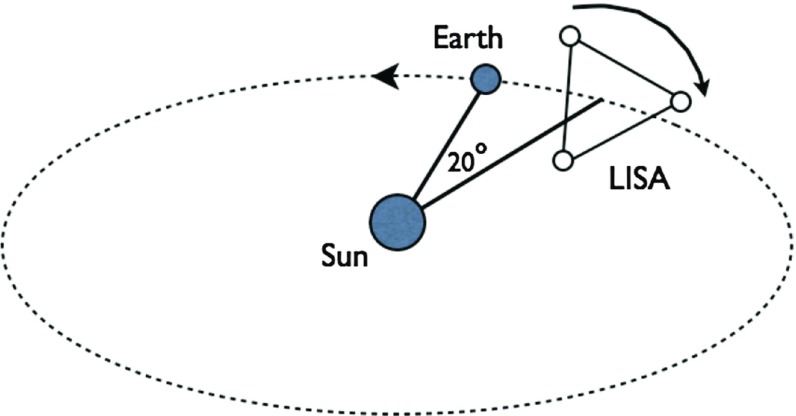
Original LISA configuration: the center-of-mass of the equilateral-triangle configuration of spacecrafts orbits the Sun in a circle of radius 1 AU, $20^\circ$ behind Earth, while the configuration ‘cartwheels’ in retrograde motion about the center-of-mass, also with a period of 1 year. [Figure adapted from Cornish and Larson (2001)]

So far, we have ignored any time-dependence in the detector response introduced by the motion of the detectors relative to the gravitational-wave source. In general, this relative motion produces a *modulation* in both the *amplitude* and the *phase* of the response of a detector to a monochromatic, plane-fronted gravitational wave (Cutler 1998). For Earth-based interferometers like LIGO, the modulation is due to both the Earth’s daily rotation and yearly orbital motion around the Sun. For space-based interferometers like LISA, the modulation is due to the motion of the individual spacecraft as they orbit the Sun with a period of 1 year. For example, for the original LISA design, three spacecraft fly in an equilateral-triangle configuration around the Sun. The center-of-mass (or guiding center) of the configuration moves in a circular orbit of radius 1 AU, at an angle of $20^\circ$ behind Earth, while the configuration ‘cartwheels’ in retrograde motion about the guiding center, also with a period of 1 year (see Fig. 39).

#### Monochromatic plane waves

The phase modulation of a monochromatic plane wave will have contributions from both the time-varying *orientation* of the detector as well as the detector’s *translational* motion relative the source. The time-varying orientation leads to changes in the response of the detector to the $+$ and $\times$ polarization components of the wave, $|R^+ h_+|$ and $|R^\times h_\times |$. The translational motion leads to a Doppler shift in the observed frequency of the wave, which is proportional to *v* / *c* times the nominal frequency, where *v* is velocity of the detector relative to the source:

5.55 $$\begin{aligned} \Delta _{\mathrm{D}} f = \frac{1}{2\pi } \frac{d\varphi _{\mathrm{D}}(t)}{dt} =-f\hat{n}\cdot {\vec {v}}(t)/c. \end{aligned}$$

For example, for a monochromatic source with $f=100~\mathrm{Hz}$ observed by ground-based detectors like LIGO, the Earth’s daily rotational motion ($v\approx 500~\mathrm{m/s}$) produces a Doppler shift of order ${\sim } 10^{-4}~\mathrm{Hz}$, while the Earth’s yearly orbital motion ($v\approx 3\times 10^4~\mathrm{m/s}$), produces a shift of order ${\sim } 10^{-2}~\mathrm{Hz}$. A matched-filter search for a sinusoid must take this latter modulation into account, as the frequency shift is larger than the width of a frequency bin for a typical search for such a signal.

#### Stochastic backgrounds

For stochastic gravitational-wave backgrounds, things are slightly more complicated as the signal is an incoherent sum of sinusoidal plane waves having different amplitudes, frequencies, and phases, and coming from different directions on the sky (2.1). But since the signal is *broad-band*, the Doppler shift associated with the phase modulation of the individual component plane waves is not important, as the gravitational-wave signal power is (at worst) shuffled into nearby bins.^15^ On the other hand, the amplitude modulation of the signal, due to the time-varying orientation of a detector, *can* be significant if the background is *anisotropic*—i.e., stronger coming from certain directions on the sky than from others. (We will discuss searches for anisotropic backgrounds in detail in Sect. 7). As the lobes of the antenna pattern sweep through the “hot” and “cold” spots of the anisotropic background, the amplitude of the signal is modulated in time.

**Fig. 40:**
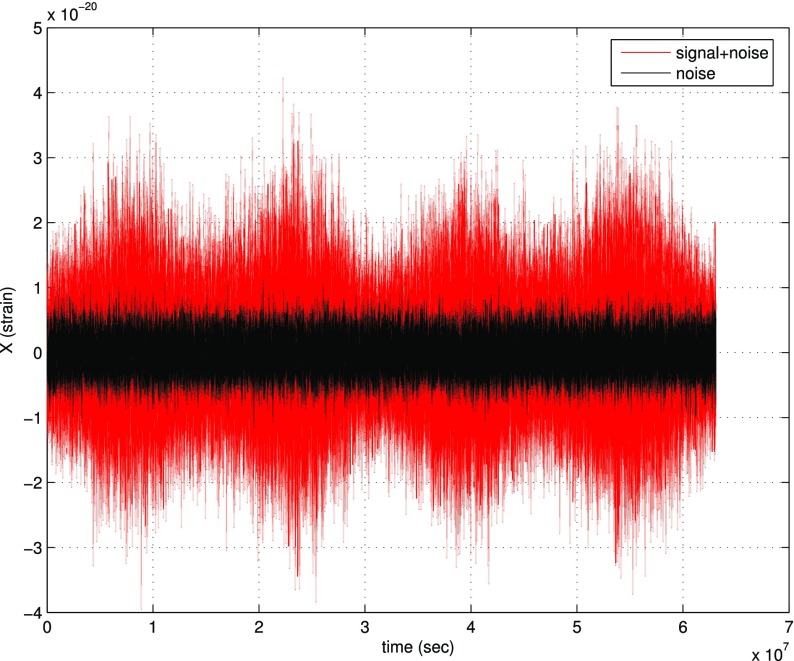
The time-domain output of a particular Michelson combination, *X*(*t*), of the LISA data over a 2-year period. The contribution from the detector noise is shown in *black*. The combined output, consisting of both detector noise and the confusion noise from the Galactic population of compact white-dwarf binaries, is shown in *red*. The modulation in the amplitude is due to the time-varying orientation of the LISA constellation as it performs a ‘cart-wheel’ in its 1-year orbit around the Sun (Fig. 39). The amplitude of the output is largest when the main lobes of LISA’s antenna pattern points in the general direction of the galactic center. (Data provided by Matt Benacquista)

Figure 40 shows the expected time-domain output of a particular Michelson combination, *X*(*t*), of the LISA data over a 5-year period. The combined signal (red) consists of both detector noise (black) and the confusion-limited gravitational-wave signal from the galactic population of compact white-dwarf binaries. At frequencies ${\sim } 10^{-4} - 10^{-3}~\mathrm{Hz}$, which corresponds to the lower end of LISA’s sensitivity band, the contribution from these binaries dominates the detector noise. The modulation of the detector output is clearly visible in the figure. The peaks in amplitude are more than 50% larger than the minimima; they repeat on a 6 month time scale, as expected from LISA’s yearly orbital motion around the Sun (Fig. 39).

**Fig. 41:**
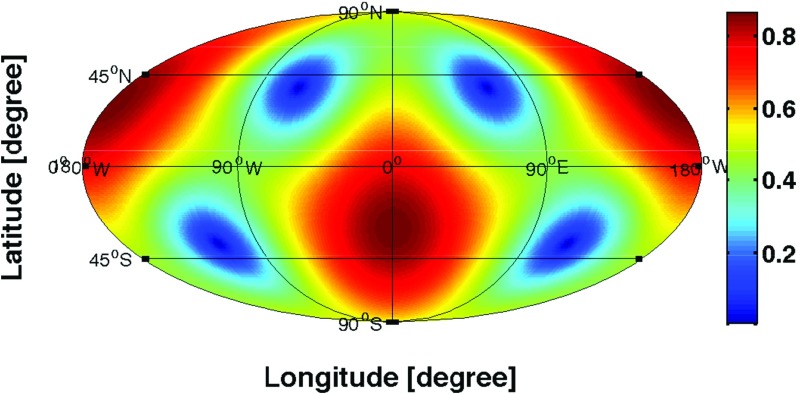
A single frame of an animation showing the time evolution of the LISA antenna pattern, represented as a colorbar plot on a Mollweide projection of the sky in ecliptic coordinates. Maxima (minima) of the antenna pattern are shown by the *red* (*blue*) *regions*. The full animation corresponds to a period of 1 year. To view the animation, please go to the online version of this review article at http://dx.doi.org/10.1007/s41114-017-0004-1

Figure 41 is a single frame of an animation showing the time evolution of the LISA antenna pattern, represented as a colorbar plot on a Mollweide projection of the sky in ecliptic coordinates. The peaks in the detector output that we saw earlier in Fig. 40 correspond to those times when the maxima of the antenna pattern point in the general direction of the galactic center, $(\mathrm{lon},\mathrm{lat})=(-93.3^\circ , -5.6^\circ )$ in ecliptic coordinates.^16^ The motion of the LISA constellation was taken from Cutler (1998), and the antenna pattern was calculated for the *X*-Michelson combination of the LISA data, assuming the small-antenna approximation for the interferometer response functions. The full animation corresponds to LISA’s orbital period of 1 year. Go to http://dx.doi.org/10.1007/s41114-017-0004-1 to view the animation.

#### Rotational and orbital motion of Earth-based detectors

As mentioned above, given the broad-band nature of a stochastic signal, the Doppler shift associated with the motion of a detector does not play an important role for stochastic background searches. This means that we can effectively ignore the velocity of a detector, and treat its motion as *quasi-static*. So, for example, the motion of a single Earth-based detector like LIGO can be thought of as synthesizing a *set* of *static* virtual detectors located along an approximately circular ring 1 AU from the solar system barycenter (Romano et al. 2015). Each virtual detector in this set observes the gravitational-wave background from a different spatial location and with a different orientation.

As described in Romano et al. (2015), the relevant time-scale for a set of virtual detectors is the time over which measurements made by the different virtual detectors are *correlated* with one another. Basically, we want two neighboring virtual detectors to be spaced far enough apart that they provide *independent* information about the background. For a gravitational wave of frequency *f*, the minimal separation corresponds to $|\Delta {\vec {x}}|\approx \lambda /2$, where $\lambda = c/f$ is the wavelength of the gravitational wave. For smaller separations, the two detectors will be driven in coincidence (on average), as discussed in item (iii) at the very end of Sect. 5.4.1. Writing $|\Delta {\vec {x}}|= v\Delta t$ and solving for $\Delta t$ yields

5.56 $$\begin{aligned} \Delta t\approx \frac{\lambda }{2v} = \frac{c}{2vf}\equiv t_\mathrm{corr}, \end{aligned}$$

where $t_\mathrm{corr}$ is the correlation time-scale. For $\Delta t \lesssim t_\mathrm{corr}$, the measurements taken by the two virtual detectors will be correlated with one another; for $\Delta t\gtrsim t_\mathrm{corr}$ the measurements will be uncorrelated with one another.

As a concrete example, let us consider a gravitational wave having frequency $f=100~\mathrm{Hz}$, and calculate the correlation time scale for the Earth’s rotational and orbital motion, treated independently. Since $v\approx 500~\mathrm{m/s}$ for daily rotation and $v\approx 3\times 10^4~\mathrm{m/s}$ for orbital motion, we get

5.57 $$\begin{aligned} \begin{array}{l@{\quad }l} t_\mathrm{corr}\approx 3000 ~\mathrm{s} &{} (\mathrm{rotational\ motion}), \\ t_\mathrm{corr}\approx 50 ~\mathrm{s} &{} (\mathrm{orbital\ motion}). \end{array} \end{aligned}$$

Thus, the orbital motion of the Earth around the Sun will more rapidly synthesize a large network of independent detectors from the motion of a single detector, compared to just rotational motion.

**Fig. 42:**
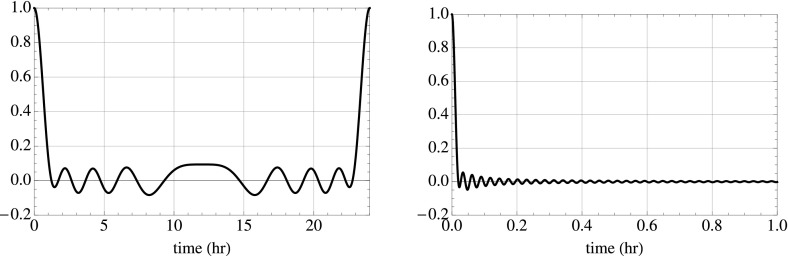
Overlap function at $f=100~\mathrm{Hz}$ for two virtual interferometers as a function of time. The *left-hand plot* is for a set of virtual interferometers located on Earth’s equator, associated with Earth’s daily rotational motion. The *right-hand plot* is for a set of virtual interferometers at $1~\mathrm{AU}$ from the SSB, associated with Earth’s yearly orbital motion. The first zero-crossing times in these two plots are consistent with the correlation times given in (5.57). Image reproduced with permission from Romano et al. (2015), copyright by APS

We can confirm these approximate results by plotting the overlap function at $f=100~\mathrm{Hz}$ for two virtual interferometers synthesized by the Earth’s rotational and orbital motion as function of time. This is done in Fig. 42, assuming an isotropic and unpolarized stochastic background, and using the small-antenna approximation to calculate the detector response functions. The left-hand plot is for a set of virtual interferometers synthesized by the daily rotation of a detector located on the Earth’s equator, with no orbital motion. The center of the Earth is fixed at the solar system barycenter, and the virtual interferometers have one arm pointing North and the other pointing East. One sees from the plot that the virtual interferometers decorrelate on a timescale of roughly an hour, consistent with (5.57), and recorrelate after 24 h when the original detector returns to its starting position. The right-hand plot is for a set of virtual interferometers at $1~\mathrm{AU}$ from the solar system barycenter, associated with Earth’s yearly orbital motion. There is no rotational motion for this case, as the interferometers are located at the center of the Earth in its orbit around the Sun, with the orientation of the interferometer arms unchanged by the orbital motion. Here we see that the virtual interferometers decorrelate on a timescale of roughly 1 min, again consistent with (5.57). They will recorrelate only after 1 year (not shown on the plot). Since the orbital velocity of the Earth is much larger than the velocity of a detector on the surface of the Earth due to the Earth’s daily rotational motion, the virtual interferometers associated with orbital motion build up a larger separation and decorrelate on a much shorter time scale.

We will return to this idea of using the motion of a detector to synthesize a set of static virtual detectors when we discuss a *phase-coherent* approach for mapping anisotropic gravitational-wave backgrounds in Sect. 7.5.

## Optimal filtering

Filters are for cigarettes and coffee. *Cassandra Clare*

Optimal filtering, in its most simple form, is a method of combining data so as to extremize some quantity of interest. The optimality criterion depends on the particular application, but for signal processing, one typically wants to: (i) maximize the detection probability for a fixed rate of false alarms, (ii) maximize the signal-to-noise ratio of some test statistic, or (iii) find the minimal variance, unbiased estimator of some quantity. Finding such optimal combinations plays a key role in both Bayesian and frequentist approaches to statistical inference (Sect. 3), and it is an important tool for every data analyst. For a Bayesian, the optimal combinations are often implicitly contained in the likelihood function, while for a frequentist, optimal filtering is usually more explicit, as there is much more freedom in the construction of a statistic.

In this section, we give several simple examples of optimal (or matched) filtering for deterministic signals, and we then show how the standard optimally-filtered cross-correlation statistic (Allen 1997; Allen and Romano 1999) for an Gaussian-stationary, unpolarized, isotropic gravitational-wave background can be derived as a matched-filter statistic for the expected cross-correlation. This derivation of the optimally-filtered cross-correlation statistic differs from the standard derivation given, e.g., in Allen (1997), but it illustrates a connection between searches for deterministic and stochastic signals, which is one of the goals of this review article.

### Optimal combination of independent measurements

As a simple explicit example, suppose we have *N* *independent* measurements

6.1 $$\begin{aligned} d_i = a+n_i, \quad i=1,2,\ldots ,N, \end{aligned}$$

where *a* is some astrophysical parameter that we want to estimate and $n_i$ are (independent) noise terms. Assuming the noise has zero mean and known variance $\sigma _i^2$ (which can be different from measurement to measurement), it follows that

6.2 $$\begin{aligned} \langle d_i\rangle = a, \quad \mathrm {Var}(d_i) \equiv \langle d_i^2\rangle - \langle d_i\rangle ^2 =\sigma _i^2. \end{aligned}$$

The goal is to find a linear combination of the data

6.3 $$\begin{aligned} \hat{a}\equiv \sum _i \lambda _i d_i \end{aligned}$$

that is optimal in the sense of being an *unbiased, minimal variance* estimator of *a*. Unbiased (i.e., $\langle \hat{a}\rangle =a$) implies

6.4 $$\begin{aligned} \sum _i \lambda _i=1, \end{aligned}$$

while minimum variance implies

6.5 $$\begin{aligned} \mathrm {Var}(\hat{a}) \equiv \sigma _{\hat{a}}^2 =\sum _i\lambda _i^2 \sigma _i^2 =\mathrm {minimum}. \end{aligned}$$

Since (6.4) is a constraint that must hold when we minimize the variance, we can use Lagrange’s method of undetermined multipliers (Boas 2006) and minimize instead

6.6 $$\begin{aligned} f(\lambda _i,\Lambda ) \equiv \sum _i\lambda _i^2 \sigma _i^2 +\Lambda \left( 1-\sum _i\lambda _i\right) \end{aligned}$$

with respect to both $\lambda _i$ and $\Lambda$. The final result is:

6.7 $$\begin{aligned} \lambda _i = \left( \sum _j \frac{1}{\sigma _j^2}\right) ^{-1} \frac{1}{\sigma _i^2} \end{aligned}$$

so that

6.8 $$\begin{aligned} \hat{a} = \left( \sum _j \frac{1}{\sigma _j^2}\right) ^{-1} \sum _i \frac{d_i}{\sigma _i^2}. \end{aligned}$$

Thus, the linear combination is a *weighted average* that gives less weight to the noiser measurements (i.e., those with large variance $\sigma _i^2$). The variance of the optimal combination is

6.9 $$\begin{aligned} \sigma _{\hat{a}}^2 =\left( \sum _j \frac{1}{\sigma _j^2}\right) ^{-1}. \end{aligned}$$

If the individual variances happen to be equal (i.e., $\sigma _i^2\equiv \sigma ^2$), then the above expressions reduce to $\hat{a} = N^{-1}\sum _i d_i$ and $\sigma _{\hat{a}}^2 = \sigma ^2/N$, which are the standard formulas for the sample mean and the reduction in the variance for *N* independent and identically-distributed measurements as we saw in Sect. 3.5.

The above results can also be derived by maximizing the likelihood function

6.10 $$\begin{aligned} p(d|a,\sigma _1^2, \sigma _2^2,\ldots ,\sigma _N^2)= \frac{1}{(2\pi )^{N/2} \sqrt{\sigma _1^2\sigma _2^2\ldots \sigma _N^2}} \exp \left[ -\frac{1}{2}\sum _{i=1}^N\frac{(d_i-a)^2}{\sigma _i^2}\right] \end{aligned}$$

with respect to the signal parameter *a*, assuming that the noise terms $n_i$ are Gaussian-distributed and independent of one another. In fact, similar to what we showed in Sect. 3.5, one can rewrite the argument of the exponential so that

6.11 $$\begin{aligned} p(d|a,\sigma _1^2, \sigma _2^2,\ldots ,\sigma _N^2) \propto \exp \left[ -\frac{1}{2}\frac{(a-\hat{a})^2}{\sigma _{\hat{a}}^2}\right] , \end{aligned}$$

where $\hat{a}$ and $\sigma _{\hat{a}}^2$ are given by (6.8) and (6.9), respectively. From this expression, it immediately follows that $\hat{a}$ maximizes the likelihood, and also the posterior distribution of *a*, if the prior for *a* is flat.

### Correlated measurements

Suppose the *N* measurements $d_i$ are *correlated*, so that the covariance matrix *C* has non-zero elements

6.12 $$\begin{aligned} C_{ij} \equiv \langle d_i d_j\rangle -\langle d_i\rangle \langle d_j\rangle \end{aligned}$$

when $i\ne j$. Again, we want to find a linear combination (6.3) that is unbiased and has minimum variance

6.13 $$\begin{aligned} \sigma _{\hat{a}}^2 =\sum _i\sum _j\lambda _i\lambda _j C_{ij}. \end{aligned}$$

By following the same Lagrange multiplier procedure described in the previous subsection, one can show that the optimal estimator is

6.14 $$\begin{aligned} \hat{a} = \left( \sum _k\sum _l \left( C^{-1}\right) _{kl}\right) ^{-1} \sum _i \sum _j \left( C^{-1}\right) _{ij} d_i. \end{aligned}$$

Thus, the weighting factors $1/\sigma _i^2$ of the previous subsection are replaced by $\sum _j (C^{-1})_{ij}$. Note that for uncorrelated measurements, $C_{ij}=\delta _{ij}\sigma _i^2$, so the above expression for $\hat{a}$ reduces to that found previously in (6.8).

Note that although (6.14) shows how to optimally combine data that are correlated with one another, it turns out that for most practical purposes one can get by using expressions like (6.8) and (6.18) below, which are valid for *uncorrelated* data. This is because the values of the Fourier transform of a stationary random process are uncorrelated for different frequency bins. Basically, the Fourier transform is a rotation in data space to a basis in which the covariance matrix is diagonal; this is called a *Karhunen–Loeve transformation*. (See also Appendix D.6). This is one of the reasons why much of signal processing is done in the frequency domain.

### Matched filter

Suppose that the astrophysical signal is not constant but also has a ‘shape’ $h_i$ so that

6.15 $$\begin{aligned} d_i = a h_i + n_i, \quad i=1,2,\ldots ,N. \end{aligned}$$

We will assume that the $h_i$ are known, so that the only unknown signal parameter is *a*. We will also assume that the different measurements are independent, as will be the case for a stationary random process in the frequency domain. Since $\langle d_i\rangle = a h_i$ is not a constant, the analysis of the previous subsection does not immediately apply. However, if we simply rescale $d_i$ by $h_i$, we obtain a new set of measurements

6.16 $$\begin{aligned} \bar{d}_i \equiv d_i/h_i \end{aligned}$$

for which

6.17 $$\begin{aligned} \langle \bar{d}_i\rangle = a, \quad \mathrm {Var}(\bar{d}_i) \equiv \bar{\sigma }_i^2 =\sigma _i^2/h_i^2, \end{aligned}$$

so that the previous analysis *is* now valid. Thus,

6.18 $$\begin{aligned} \hat{a} =\left( \sum _j \frac{1}{\bar{\sigma }_j^2}\right) ^{-1} \sum _i \frac{\bar{d}_i}{\bar{\sigma }_i^2} =\left( \sum _j \frac{h_j^2}{\sigma _j^2}\right) ^{-1} \sum _i \frac{h_i d_i}{\sigma _i^2} \end{aligned}$$

is the optimal estimator of *a*.

The above expression for $\hat{a}$ is often called a *matched filter* (Wainstein and Zubakov 1971) since the data $d_i$ are projected onto the expected signal shape $h_i$ (as well as weighted by the inverse of the noise variance $\sigma _i^2$). The particular combination

6.19 $$\begin{aligned} Q_i \equiv h_i/\sigma _i^2 \end{aligned}$$

multiplying $d_i$ is the *optimal filter* for this analysis.^17^ When there are many possible candidate signal shapes, one constructs a *template bank*—i.e., a collection of possible shapes against which the data compared. By normalizing each of the templates so that $\sum _i (h_i^2/\sigma _i^2)=1$, the signal-to-noise ratio of the matched filter

6.20 $$\begin{aligned} \hat{\rho }(h)\equiv \sum _i \frac{h_i d_i}{\sigma _i^2}, \end{aligned}$$

or its square, can be used as a frequentist detection statistic. That is, the maximum value of $\hat{\rho }(h)$ over the space of templates $\{h_i\}$ is compared against some threshold $\rho _*$ (chosen so that the false alarm probability is below some acceptable value). If the maximum signal-to-noise ratio exceeds the threshold, then one claims detection of the signal with a certain level of confidence. The shape of the detected signal is that which corresponds to the maximum matched-filter signal-to-noise ratio.

### Optimal filtering for a stochastic background

As noted by Fricke (2006), the above results can be used to derive the optimal cross-correlation statistic for the stochastic background search. (A more standard derivation can be found, e.g., in Allen 1997). To see this, consider a cross-correlation search for a Gaussian-stationary, unpolarized, isotropic gravitational-wave background using two detectors having uncorrelated noise. Let *T* be the total observation time of the measurement. In the frequency domain, the measurements are given by the values of the complex-valued cross-correlation

6.21 $$\begin{aligned} x(f)=\tilde{d}_1(f)\tilde{d}_2^*(f) \end{aligned}$$

where $\tilde{d}_I(f)$, $I=1,2$ are the Fourier transforms of the time-series output of the two detectors:

6.22 $$\begin{aligned} \begin{aligned} d_1(t)&=h_1(t) + n_1(t), \\ d_2(t)&=h_2(t) + n_2(t). \end{aligned} \end{aligned}$$

The *x*(*f*) for different frequencies correspond to the measurements $d_i$ of the previous subsections. Since we are assuming uncorrelated detector noise,

6.23 $$\begin{aligned} \langle x(f)\rangle =\langle \tilde{h}_1(f)\tilde{h}_2^*(f)\rangle =\frac{T}{2}\Gamma _{12}(f)S_h(f), \end{aligned}$$

where $S_h(f)$ is the power spectral density of the stochastic background signal, and $\Gamma _{12}(f)$ is the overlap function for the two detectors.^18^ In the weak-signal limit, the covariance matrix is dominated by the diagonal terms:

6.24 $$\begin{aligned} \begin{aligned} C_{ff'}&\equiv \langle x(f)x^*(f')\rangle - \langle x(f)\rangle \langle x^*(f')\rangle \\&\approx \langle \tilde{n}_1(f)\tilde{n}^*_1(f')\rangle \langle \tilde{n}^*_2(f)\tilde{n}_2(f')\rangle \\&=\frac{T}{4} P_{n_1}(f)P_{n_2}(f)\,\delta (f-f'), \end{aligned} \end{aligned}$$

where $P_{n_I}(f)$ are the 1-sided power spectral densities of the noise in the two detectors:

6.25 $$\begin{aligned} \langle \tilde{n}_I(f)\tilde{n}^*_I(f')\rangle = \frac{1}{2}P_{n_I}(f)\,\delta (f-f'). \end{aligned}$$

Thus, in this approximation

6.26 $$\begin{aligned} \int _{-\infty }^\infty df'\> (C^{-1})_{ff'} \approx \frac{4}{T}\frac{1}{P_{n_1}(f) P_{n_2}(f)}. \end{aligned}$$

Now, suppose we are searching for a stochastic background with a power-law spectrum

6.27 $$\begin{aligned} \Omega _\mathrm {gw}(f)=\Omega _\beta \left( \frac{f}{f_{\mathrm {ref}}}\right) ^\beta , \end{aligned}$$

whose amplitude $\Omega _\beta$ we would like to estimate. Then, according to (2.18),

6.28 $$\begin{aligned} S_h(f) = \frac{3 H_0^2}{2\pi ^2} \frac{\Omega _\beta }{f_\mathrm{ref}^3} \left( \frac{f}{f_{\mathrm {ref}}}\right) ^{\beta -3} = \Omega _\beta H_\beta (f), \end{aligned}$$

where

6.29 $$\begin{aligned} H_\beta (f)\equiv \frac{3 H_0^2}{2\pi ^2} \frac{1}{f_\mathrm{ref}^3} \left( \frac{f}{f_{\mathrm {ref}}}\right) ^{\beta -3}. \end{aligned}$$

Using the above form of $S_h(f)$ and (6.23), we see that

6.30 $$\begin{aligned} \frac{T}{2}\Gamma _{12}(f)H_\beta (f) \quad \longleftrightarrow \quad h_i \end{aligned}$$

is the expected signal ‘shape’ $h_i$ in the notation of the previous subsection. Given (6.26) and (6.30), it is now a simple matter to show that

6.31 $$\begin{aligned} \hat{\Omega }_\beta =\mathcal{N}\int _{-\infty }^\infty df\> \frac{\Gamma _{12}(f)H_\beta (f)}{P_{n_1}(f) P_{n_2}(f)} \tilde{d}_1(f)\tilde{d}_2^*(f), \end{aligned}$$

where

6.32 $$\begin{aligned} \mathcal{N} \equiv \left[ \frac{T}{2} \int _{-\infty }^\infty df\> \frac{\Gamma ^2_{12}(f)H_\beta ^2(f)}{P_{n_1}(f) P_{n_2}(f)}\right] ^{-1}. \end{aligned}$$

The variance and expected signal-to-noise ratio of the estimator $\hat{\Omega }_\beta$ are:

6.33 $$\begin{aligned} \sigma _{\hat{\Omega }_\beta }^2 =\left[ T \int _{-\infty }^\infty df\> \frac{\Gamma ^2_{12}(f)H_\beta ^2(f)}{P_{n_1}(f) P_{n_2}(f)}\right] ^{-1}, \end{aligned}$$

and

6.34 $$\begin{aligned} \rho = \sqrt{T} \left[ \int _{-\infty }^\infty df\> \frac{\Gamma ^2_{12}(f) S_h^2(f)}{P_{n_1}(f) P_{n_2}(f)} \right] ^{1/2}. \end{aligned}$$

The combination

6.35 $$\begin{aligned} \tilde{Q}(f)\equiv \mathcal{N} \frac{\Gamma _{12}(f)H_\beta (f)}{P_{n_1}(f) P_{n_2}(f)} \end{aligned}$$

multiplying $\tilde{d}_1(f)\tilde{d}_2^*(f)$ in (6.31) is the standard optimal filter (see, e.g., Allen 1997; Allen and Romano 1999), which was derived in those references for a flat spectrum, $\beta =0$. The optimally-filtered cross-correlation statistic, denoted *S* in Allen (1997) and Allen and Romano (1999), is given by $S=\hat{\Omega }_0 T$.

#### Optimal estimators for individual frequency bins

As shown in Aasi et al. (2015), we can also construct estimators of the amplitude $\Omega _\beta$ of a power-law spectrum using cross-correlation data for *individual* frequency bins, of width $\Delta f$, centered at *each* (positive) frequency *f*:

6.36 $$\begin{aligned} \hat{\Omega }_\beta (f)\equiv \frac{2}{T} \frac{\mathfrak {R}[\tilde{d}_1(f) \tilde{d}_2^*(f)]}{\Gamma _{12}(f)H_\beta (f)}. \end{aligned}$$

Note that these estimators are just the measured values of the cross-spectrum divided by the expected spectral shape of the cross-correlation due to a gravitational-wave background with spectral index $\beta$. In the above expression, *T* is the duration of the data segments used in calculating the Fourier transforms $\tilde{d}_1(f)$, $\tilde{d}_2(f)$; and $\Gamma _{12}(f)$ is the overlap function for the two detectors.

In the absence of correlated noise, the above estimators are optimal in the sense that they are unbiased estimators of $\Omega _\beta$ and have minimal variance for a single bin:

6.37 $$\begin{aligned} \sigma ^2_{\hat{\Omega }_\beta }(f) \approx \frac{1}{2T\Delta f}\frac{P_{n_1}(f) P_{n_2}(f)}{\Gamma ^2_{12}(f)H^2_\beta (f)}, \end{aligned}$$

where we assumed the weak-signal limit to obtain the approximate equality for the variance. For a frequency band consisting of many bins of width $\Delta f$, we can optimally combine the individual estimators $\hat{\Omega }_\beta (f)$ using the standard $1/\sigma ^2$-weighting discussed earlier:

6.38 $$\begin{aligned} \hat{\Omega }_\beta \equiv \frac{\sum _f \sigma ^{-2}_{\hat{\Omega }_\beta }(f) \hat{\Omega }_\beta (f)}{\sum _{f'} \sigma ^{-2}_{\hat{\Omega }_\beta }(f')}, \quad \sigma ^{2}_{\hat{\Omega }_\beta }\equiv \left[ \sum _{f} \sigma ^{-2}_{\hat{\Omega }_\beta }(f)\right] ^{-1}. \end{aligned}$$

The expressions for $\hat{\Omega }_\beta$ and $\sigma ^2_{\hat{\Omega }_\beta }$ obtained in this way reproduce the standard optimal filter expressions (6.31) and (6.33) in the limit where $\Delta f\rightarrow df$ and the sums are replaced by integrals.

#### More general parameter estimation

The analyses in the previous two subsections take as given the spectral shape of an isotropic stochastic background, and then construct estimators of its overall amplitude. But it is also possible to construct estimators of *both* the amplitude and spectral index of the background. One simply treats these as free parameters in the signal model e.g., when constructing the likelihood function. Interested readers should see Mandic et al. (2012) for details.

## Anisotropic backgrounds

Sameness is the mother of disgust, variety the cure. *Francesco Petrarch*

An anisotropic background of gravitational radiation has *preferred* directions on the sky—the associated signal is stronger coming from certain directions (“hot” spots) than from others (“cold” spots). The anisotropy is produced primarily by sources that follow the local distribution of matter in the universe (e.g., compact white-dwarf binaries in our galaxy), as opposed to sources at *cosmological* distances (e.g., cosmic strings or quantum fluctuations in the gravitational field amplified by inflation Allen, 1997; Maggiore, 2000), which would produce an *isotropic* background. This means that the measured distribution of gravitational-wave power on the sky can be used to discriminate between cosmologically-generated backgrounds, produced in the very early Universe, and astrophysically-generated backgrounds, produced by more recent populations of astrophysical sources. In addition, an anisotropic distribution of power may allow us to detect the gravitational-wave signal in the first place; as the lobes of the antenna pattern of a detector sweep across the “hot” and “cold” spots of the anisotropic distribution, the amplitude of the signal is modulated in time, while the detector noise remains unaffected (Adams and Cornish 2010).

In this section, we describe several different approaches for searching for anisotropic backgrounds of gravitational waves: The first approach (described in Sect. 7.2) looks for modulations in the correlated output of a pair of detectors, at harmonics of the rotational or orbital frequency of the detectors (e.g., daily rotational motion for ground-based detectors like LIGO, Virgo, etc., or yearly orbital motion for space-based detectors like LISA). This approach assumes a known distribution of gravitational-wave power $\mathcal{P}(\hat{n})$, and filters the data so as to maximize the signal-to-noise ratio of the harmonics of the correlated signal. The second approach (Sect. 7.3) constructs maximum-likelihood estimates of the gravitational-wave power on the sky based on cross-correlated data from a network of detectors. This approach produces sky maps of $\mathcal{P}(\hat{n})$, analogous to sky maps of temperature anisotropy in the cosmic microwave background radiation. The third approach (Sect. 7.4) constructs frequentist detection statistics for either an unknown or an assumed distribution of gravitational-wave power on the sky. The fourth and final approach we describe (Sect. 7.5) attempts to measure both the amplitude *and* phase of the gravitational-wave background at each point on the sky, making minimal assumptions about the statistical properties of the signal. This latter approach produces sky maps of the real and imaginary parts of the random fields $h_+(f,\hat{n})$ and $h_\times (f,\hat{n})$, from which the power in the background $\mathcal{P}(\hat{n}) = |h_+|^2 + |h_\times |^2$ is just one of many quantities that can be estimated from the measured data.

Numerous papers have been written over the last ${\approx }20$ years on the problem of detecting anisotropic stochastic backgrounds, starting with the seminal paper by Allen and Ottewill (1997), which laid the foundation for much of the work that followed. Readers interested in more details should see Allen and Ottewill (1997) regarding modulations of the cross-correlation statistic at harmonics of the Earth’s rotational frequency; Ballmer (2006a, b), Mitra et al. (2008), Thrane et al. (2009), Mingarelli et al. (2013) and Taylor and Gair (2013) for maximum-likelihood estimates of gravitational-wave power; Thrane et al. (2009) and Talukder et al. (2011) for maximum-likelihood ratio detection statistics; and Gair et al. (2014), Cornish and van Haasteren (2014) and Romano et al. (2015) regarding phase-coherent mapping. For results of actual analyses of initial LIGO data and pulsar timing data for anisotropic backgrounds, see Abadie et al. (2011) and Taylor et al. (2015) and Sect. 10.2.5.

Note that we will not discuss in any detail methods to detect anisotropic backgrounds using space-based interferometers like LISA or the Big-Bang Observer (BBO). As mentioned in Sect. 5.5.2, the confusion noise from the galactic population of compact white dwarf binaries is a guaranteed source of anisotropy for such detectors. At low frequencies, measurements made using a single LISA will be sensitive to only the $l=0,2,4$ components of the background, while cross-correlating data from two independent LISA-type detectors (as in BBO) will allow for extraction of the full range of multipole moments. The proposed data analysis methods are similar to those that we will discuss in Sects. 7.2 and 7.3, but using the synthesized *A*, *E*, and *T* data channels for a single LISA (see Sect. 9.7). Readers should see Giampieri and Polnarev (1997), Cornish (2001), Ungarelli and Vecchio (2001), Seto (2004), Seto and Cooray (2004), Kudoh and Taruya (2005), Edlund et al. (2005) and Taruya and Kudoh (2005) for details.

### Preliminaries

#### Quadratic expectation values

For simplicity, we will restrict our attention to Gaussian-stationary, unpolarized, anisotropic backgrounds with quadratic expectation values given by (2.16):

7.1 $$\begin{aligned} \langle h_A(f,\hat{n}) h_{A'}^*(f',\hat{n}')\rangle =\frac{1}{4}\mathcal{P}(f,\hat{n}) \delta (f-f') \delta _{AA'} \delta ^2(\hat{n},\hat{n}'), \end{aligned}$$

where

7.2 $$\begin{aligned} S_h(f) = \int d^2\Omega _{\hat{n}}\> \mathcal{P}(f,\hat{n}). \end{aligned}$$

We will also assume that $\mathcal{P}(f,\hat{n})$ factorizes

7.3 $$\begin{aligned} \mathcal{P}(f,\hat{n}) = \bar{H}(f)\mathcal{P}(\hat{n}), \end{aligned}$$

so that the angular distribution of power on the sky is independent of frequency. We will chose our normalization so that $\bar{H}(f_\mathrm{ref})=1$, where $f_\mathrm{ref}$ is a reference frequency, typically taken to equal 100 Hz for ground-based detectors. We will also assume that the spectral shape $\bar{H}(f)$ is known, so that we only need to recover $\mathcal{P}(\hat{n})$. If we expand the power $\mathcal{P}(\hat{n})$ in terms of spherical harmonics,

7.4 $$\begin{aligned} \mathcal{P}(\hat{n}) = \sum _{l=0}^\infty \sum _{m=-l}^l \mathcal{P}_{lm} Y_{lm}(\hat{n}), \end{aligned}$$

then this normalization choice is equivalent to $\mathcal{P}_{00} = S_h(f_\mathrm{ref})/\sqrt{4\pi }$, and has units of $(\mathrm{strain})^2 \, \mathrm{Hz}^{-1}\, \mathrm{sr}^{-1}$, where $\mathrm{sr} \equiv \mathrm{rad}^2$ is one steradian. Thus, $\mathcal{P}_{00}$ is a measure of the *isotropic* component of the background, and sets the overall normalization of the strain power spectral density $S_h(f)$.

#### Short-term Fourier transforms

Since the response of a detector changes as its antenna pattern sweeps across the “hot” and “cold” spots of an anisotropic distribution, we will need to split the data taken by the detectors into chunks of duration $\tau$, where $\tau$ is much greater than the light-travel time between any pair of detectors, but small enough that the detector response functions do not change appreciably over that interval. (For Earth-based interferometers like LIGO, $\tau \sim 100~\mathrm{s}$ to 1000 s is appropriate). Each chunk of data $[t-\tau /2,t+\tau /2]$ will then be Fourier transformed over the duration $\tau$, yielding

7.5 $$\begin{aligned} \tilde{d}_I(t;f) = \int _{t-\tau /2}^{t+\tau /2} dt'\> d_I(t') e^{-i2\pi ft'}. \end{aligned}$$

This operation is often called a *short-term* Fourier transform. Note that, in this notation, *t* labels a *particular* time chunk, and is not a variable that is subsequently Fourier transformed.

#### Cross-correlations

For many of the approaches that map the distribution of gravitational-wave power, it is convenient to work with cross-correlated data from two detectors, evaluated at the same time chunk *t* and frequency *f*:

7.6 $$\begin{aligned} \hat{C}_{IJ}(t; f) = \frac{2}{\tau }\tilde{d}_I(t;f)\tilde{d}_J^*(t;f). \end{aligned}$$

The factor of 2 is a convention consistent with the choice of one-sided power spectra. Assuming uncorrelated detector noise and using expectation values given in (7.1), we find

7.7 $$\begin{aligned} \langle \hat{C}_{IJ}(t; f)\rangle =\bar{H}(f) \int d^2\Omega _{\hat{n}} \> \gamma _{IJ}(t; f, \hat{n})\mathcal{P}(\hat{n}), \end{aligned}$$

where

7.8 $$\begin{aligned} \gamma _{IJ}(t; f, \hat{n}) \equiv \frac{1}{2}\sum _A R^A_I(t; f, \hat{n}) R^{A*}_J(t; f, \hat{n}). \end{aligned}$$

Note that up to a factor of $1/(4\pi )$, the function $\gamma _{IJ}(t;, f,\hat{n})$ is just the integrand of the isotropic overlap function $\Gamma _{IJ}(f)$ given by (5.36). In what follows, we will drop the detector labels *IJ* from both $\hat{C}_{IJ}(t;f)$ and $\gamma _{IJ}(t;f,\hat{n})$ when there is no chance for confusion.

**Fig. 43:**
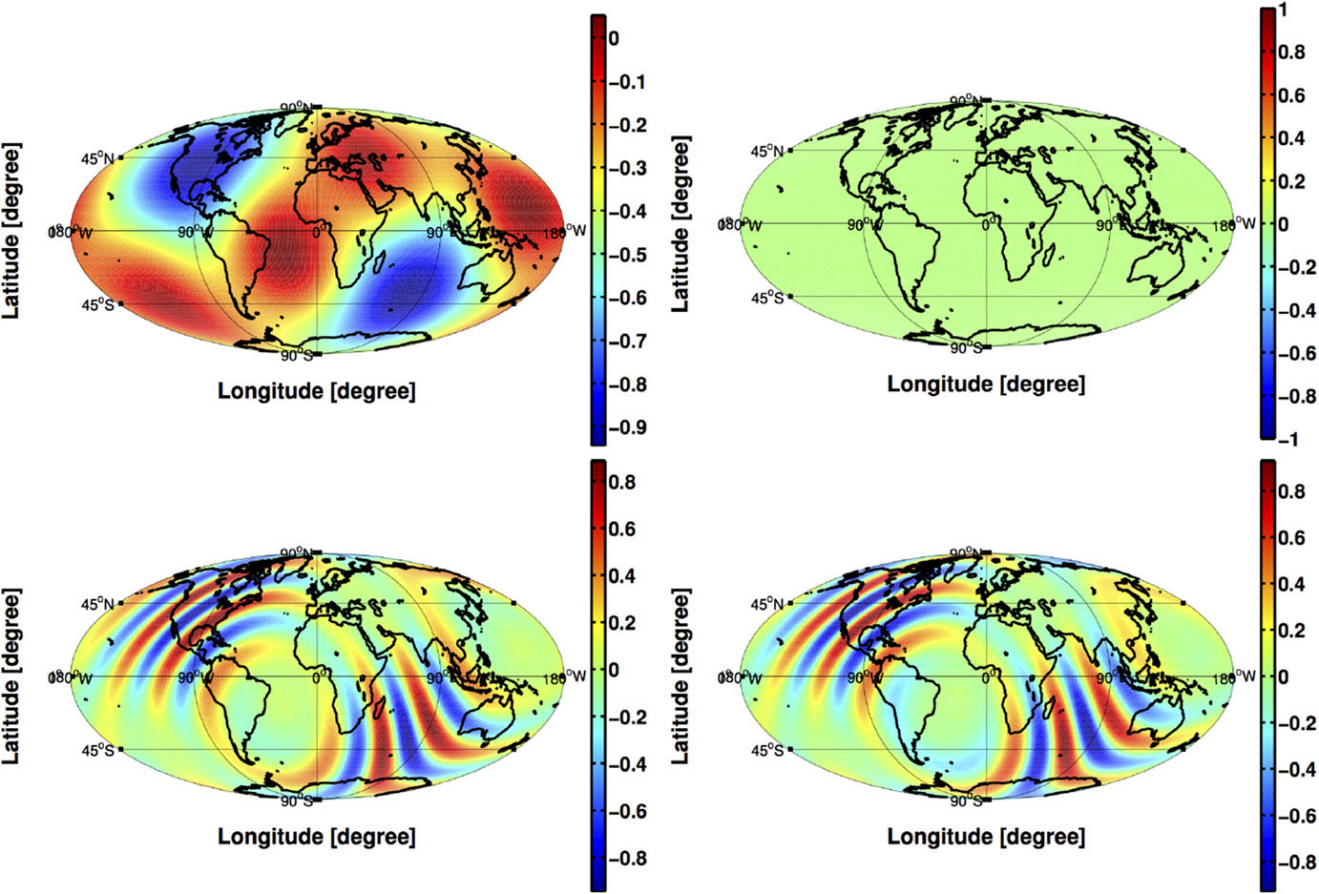
Real and imaginary parts of $\gamma (f,\hat{n})$ (appropriately normalized) for the strain response of the 4-km LIGO Hanford and LIGO Livingston interferometers for $f=0~\mathrm{Hz}$ (*top two plots*) and $f=200~\mathrm{Hz}$ (*bottom two plots*). In the *top left plot*, note the *large blue region* in the vicinity of the two detectors, corresponding to the *anti-alignment* of the Hanford and Livingston interferometers—i.e., the arms of the two interferometers are rotated by $90^\circ$ with respect to one another. As shown in the *top right plot*, there is no imaginary component to the integrand of the overlap function at 0 Hz. The *bottom two plots* show multiple positive and negative oscillations (‘lobes’), which come from the exponential factor $e^{-i 2\pi f\hat{n}\cdot \Delta {\vec {x}}/c}$ of the product of the two response functions (5.43). The location of the positive and negative lobes are shifted relative to one another for the real and imaginary parts. The separation between the lobes depends inversely on the frequency

Figure 43 shows maps of the real and imaginary parts of $\gamma (t; f, \hat{n})$ (appropriately normalized) for the strain response of the 4-km LIGO Hanford and LIGO Livingston interferometers evaluated at $f=0~\mathrm{Hz}$ (top two plots) and $f=200~\mathrm{Hz}$ (bottom two plots). (In the Earth-fixed frame, the detectors don’t move so there is no time dependence to worry about). Note the presence of oscillations or ‘lobes’ for the $f=200~\mathrm{Hz}$ plots, which come from the exponential factor $e^{-i2\pi f\hat{n}\cdot \Delta {\vec {x}}/c}$ of the product of the two response functions (5.43). For $f=0$, this factor is unity.

**Fig. 44:**
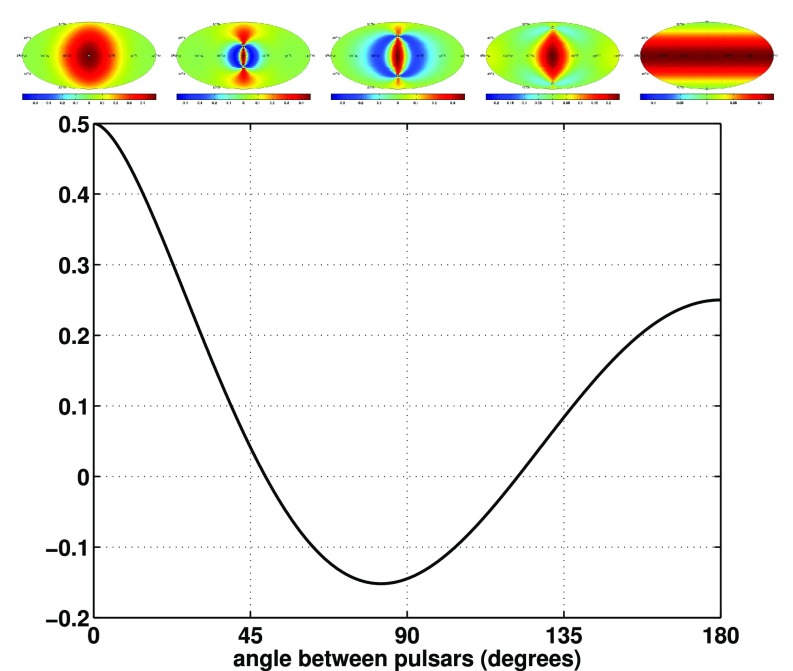
*Top row* Mollweide projections of $\gamma (\hat{n})$ for pairs of pulsars separated on the sky by $\zeta =0^\circ$, $45^\circ$, $90^\circ$, $135^\circ$, $180^\circ$. *Reddish regions* correspond to positive values of $\gamma (\hat{n})$; *blueish regions* correspond to negative values of $\gamma (\hat{n})$. *Bottom* Hellings and Downs curve as a function of the angular separation between two distinct pulsars. The integral of the top plots over the whole sky equal the values of the Hellings and Downs curve for these angular separations. (See also Fig. 37)

Figure 44 is a similar plot, showing Mollweide projections of $\gamma (t; f, \hat{n})$ for the Earth-term-only Doppler frequency response (5.21) of pairs of pulsars separated on the sky by $\zeta =0^\circ$, $45^\circ$, $90^\circ$, $135^\circ$, $180^\circ$. (There is no time dependence nor frequency dependence for these functions). The bottom panel is a plot of the Hellings and Downs curve as a function of the angular separation between a pair of Earth-pulsar baselines. By integrating the top plots over the whole sky (appropriately normalized), one obtains the values of the Hellings and Downs curve for those angular separations.

#### Spherical harmonic components of $\gamma (t; f, \hat{n})$

As first noted in Allen and Ottewill (1997), the functions $\gamma (t; f,\hat{n})$ defined above (7.8) play a very important role in searches for anisotropic backgrounds. For a fixed pair of detectors at a fixed time *t* and for fixed frequency *f*, these functions are scalar fields on the unit 2-sphere and hence can be expanded in terms of the ordinary spherical harmonics $Y_{lm}(\hat{n})$:

7.9 $$\begin{aligned} \gamma (t; f, \hat{n}) \equiv \sum _{l=0}^\infty \sum _{m=-l}^l \gamma _{lm}(t;f) Y_{lm}^*(\hat{n}), \end{aligned}$$

or, equivalently,

7.10 $$\begin{aligned} \gamma _{lm}(t;f) \equiv \int d^2\Omega _{\hat{n}}\> \gamma (t; f, \hat{n})Y_{lm}(\hat{n}). \end{aligned}$$

Note that this definition differs from (7.4) for $\mathcal{P}_{lm}$ by a complex conjugation, but agrees with the convention used in Allen and Ottewill (1997). In terms of the spherical harmonic components, it follows that

7.11 $$\begin{aligned} \int d^2\Omega _{\hat{n}}\>\gamma (t;f,\hat{n})\mathcal{P}(\hat{n}) = \sum _{l=0}^\infty \sum _{m=-l}^l \gamma _{lm}(t;f)\mathcal{P}_{lm}, \end{aligned}$$

as a consequence of the orthogonality of the $Y_{lm}(\hat{n})$. This expression enters (7.7) for the expected cross-correlation of the output in two detectors. As explained in Allen and Ottewill (1997) and Thrane et al. (2009), the time dependence of $\gamma _{lm}(t;f)$ is particularly simple:

7.12 $$\begin{aligned} \gamma _{lm}(t;f) = \gamma _{lm}(0; f)\,e^{im2\pi t/T_\mathrm{mod}}, \end{aligned}$$

where $T_\mathrm{mod}$ is the relevant modulation period associated with the motion of the detectors. For example, for ground-based detectors like LIGO and Virgo, $T_\mathrm{mod}=1$ sidereal day, since the displacement vector $\Delta {\vec {x}}(t) \equiv {\vec {x}}_2(t)-{\vec {x}}_1(t)$ connecting the vertices of the two interferometers (and which enters the expression for the overlap function) traces out a cone on the sky with a period of one sidereal day. If there is no time dependence, as is the case for pulsar timing, $T_\mathrm{mod}$ is infinite.

*Example: Earth-based interferometers*

As was also shown in Allen and Ottewill (1997), one can derive *analytic* expressions for $\gamma _{lm}(t;f)$ for a pair of Earth-based interferometers in the short-antenna limit. If we set $t=0$, then $\gamma _{lm}(0;f)$ can be written as a linear combination^19^ involving spherical Bessel functions, $j_n(x)/x^n$ (for *l* even) and $j_n(x)/x^{n-1}$ (for *l* odd), where *x* depends on the relative separation of the two detectors, $x \equiv 2\pi f|\Delta {\vec {x}}|/c$. The coefficients of the expansions are complex numbers that depend on the relative orientation of the detectors. Explicit expression for the first few spherical harmonic components for the LIGO Hanford–LIGO Livingston pair are given below:

7.13 $$\begin{aligned} \gamma _{00}(0;f)= & {} -0.0766 j_0(x) -2.1528 j_1(x)/x +2.4407 j_2(x)/x^2, \nonumber \\ \gamma _{10}(0;f)= & {} - 0.0608i\,j_1(x) - 2.6982i\,j_2(x)/x + 7.7217i\,j_3(x)/x^2, \nonumber \\ \gamma _{11}(0;f)= & {} -(0.0519 + 0.0652i)j_1(x) -(1.8621 + 1.0517i)j_2(x)/x \nonumber \\&+(4.0108 - 2.4933i)j_3(x)/x^2, \nonumber \\ \gamma _{20}(0;f)= & {} \ 0.0316 j_0(x) -0.9612 j_1(x)/x +10.9038 j_2(x)/x^2 -52.7905 j_3(x)/x^3, \nonumber \\ \gamma _{21}(0;f)= & {} -(0.0669 - 0.0532i)j_0(x) -(1.9647 - 2.6145i) j_1(x) \nonumber \\&+(15.0524 -24.7604i)j_2(x)/x^2 -(36.5620 -50.7179i)j_3/x^3, \nonumber \\ \gamma _{22}(0;f)= & {} -(0.0186 - 0.0807i) j_0(x) +(1.2473 + 1.6858i) j_1(x)/x \nonumber \\&-(12.2048 +12.5814i) j_2(x)/x^2 +(60.7859 +12.7191i) j_3(x)/x^3.\nonumber \\ \end{aligned}$$

**Fig. 45:**
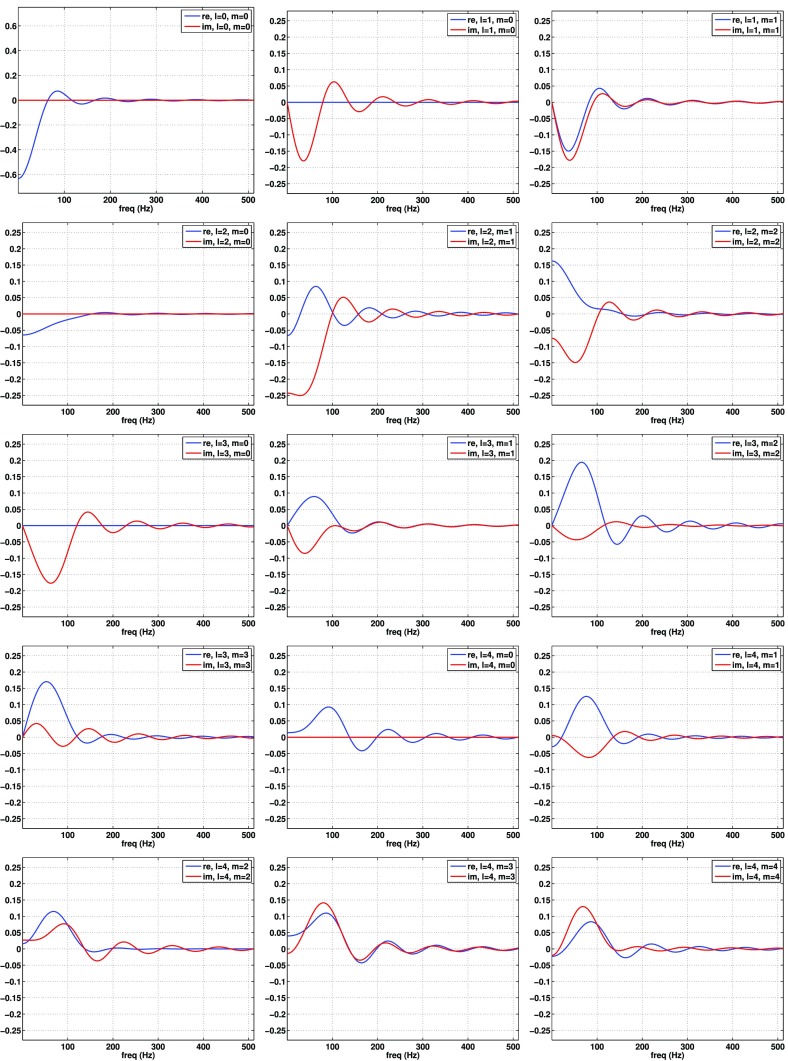
Real and imaginary parts of the spherical harmonic components $\gamma _{lm}(0;f)$ for the LIGO Hanford–LIGO Livingston detector pair. Here we show plots for $l=0$, 1, 2, 3, 4 and $m\ge 0$. For $m<0$, use (7.14)

Note that the above numerical coefficients do not agree with those in Allen and Ottewill (1997) due to an overall normalization factor of $4\pi /5$ and phase $e^{im\phi }$, where $\phi = -38.52^\circ$ is the angle between the separation vector between the vertices of the LIGO-Hanford and LIGO-Livingston interferometers and the Greenwich meridian (Thrane et al. 2009). Plots of the real and imaginary parts of $\gamma _{lm}(0;f)$ for $l=0$, 1, 2, 3, 4 and $m\ge 0$ for the LIGO Hanford-LIGO Livingston detector pair are given in Fig. 45. For $m<0$, one can use the relation

7.14 $$\begin{aligned} \gamma _{lm}(t; f) = (-1)^{l+m} \gamma _{l,-m}(t;f), \end{aligned}$$

which follows from the properties of the spherical harmonics $Y_{lm}(\hat{n})$ (see Appendix E). Note that up to an overall normalization factor of $5/\sqrt{4\pi }$, the real part of $\gamma _{00}(0;f)$ is the Hanford-Livingston overlap function for an unpolarized, isotropic stochastic background, shown in Fig. 34.

**Fig. 46:**
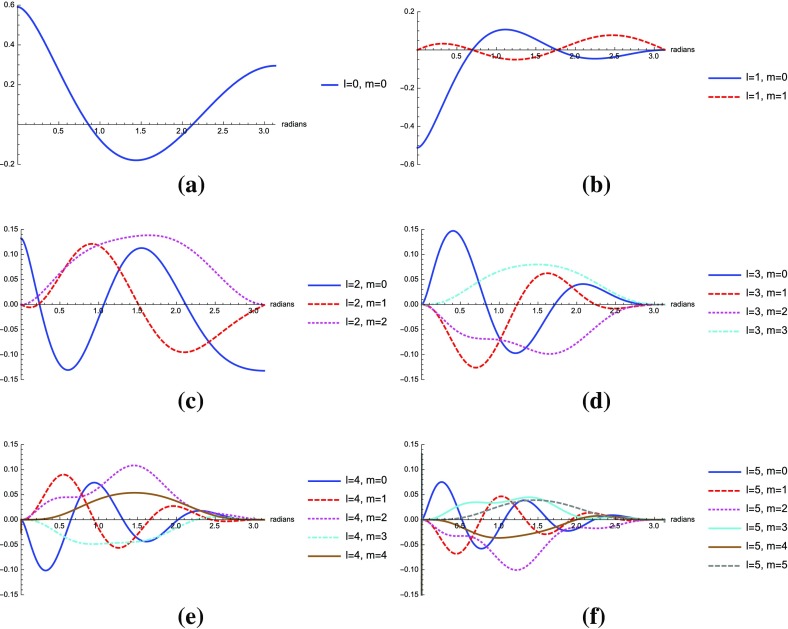
Spherical harmonic component functions $\gamma _{lm}(\zeta )$ for pulsar timing as a function of the angle $\zeta$ between two distinct pulsars. Here we show plots for $l=0,1,\ldots ,5$ and $m\ge 0$. We used the Earth-term-only Doppler-frequency response (5.21) to calculate these functions

*Example: Pulsar timing arrays*

In Fig. 46, we show plots of the spherical harmonic components of $\gamma (t;f, \hat{n})$ calculated using the Earth-term-only Doppler-frequency response functions (5.21) for pulsar timing. Since there is no frequency or time-dependence for these response functions, the spherical harmonic components of $\gamma (\hat{n})$ depend only of the angular separation $\zeta$ between the two pulsars that define the detector pair. As shown in Mingarelli et al. (2013) and Gair et al. (2014), these functions can be calculated analytically for *all* values of *l* and *m*. A detailed derivation with all the relevant formulae can be found in Appendix E of Gair et al. (2014); there the calculation is done in a ‘computational’ frame, where one of the pulsars is located along the *z*-axis and the other is in the *xz*-plane, making an angle $\zeta$ with respect to the first. In this computational frame, all of the components $\gamma _{lm}(\zeta )$ are real. Note that up to an overall normalization factor^20^ of $3/\sqrt{4\pi }$, the function $\gamma _{00}(\zeta )$ is just the Hellings and Downs function for an unpolarized, isotropic stochastic background, shown in Fig. 37.

### Modulations in the correlated output of two detectors

For ground-based detectors like LIGO and Virgo, an anisotropic gravitational-wave background will modulate the correlated output of a pair of detectors at harmonics of the Earth’s rotational frequency. It turns out that for an unpolarized, anisotropic background, the contribution to the *m*th harmonic of the correlation has a frequency dependence proportional to

7.15 $$\begin{aligned} \bar{H}(f) \sum _{l=|m|}^\infty \gamma _{lm}(0;f)\mathcal{P}_{lm}, \end{aligned}$$

where $\mathcal{P}_{lm}$ are the spherical harmonic components of the gravitational-wave power on the sky $\mathcal{P}(\hat{n})$. (We are assuming here that the spherical harmonic decomposition of $\mathcal{P}(\hat{n})$ is with respect to a coordinate system whose *z*-axis points along the Earth’s rotational axis). In this section, we derive the above result following the presentation in Allen and Ottewill (1997) and construct an optimal filter for the cross-correlation that maximizes the signal-to-noise ratio for the *m*th harmonic. This was the first concrete approach that was proposed for detecting an anisotropic stochastic background.

#### Time-dependent cross-correlation

We start by writing down an expression (in the frequency domain) for the correlated output of two ground-based detectors (e.g., LIGO Hanford and LIGO Livingston):

7.16 $$\begin{aligned} \hat{C}(t) = \int _{-\infty }^\infty df\> \tilde{Q}(t;f) \tilde{d}_1(t;f) \tilde{d}_2^*(t;f), \end{aligned}$$

where $\tilde{d}_{1,2}(t;f)$ are (short-term) Fourier transforms (7.5) centered around *t*, and where we have included a filter function $\tilde{Q}(t;f)$, whose specific form we will specify later. Since the cross-correlation is periodic with a period $T_\mathrm{mod} = 1$ sidereal day (due to the motion of the detectors attached to the surface of the Earth), we can expand $\hat{C}(t)$ as a Fourier series:

7.17 $$\begin{aligned} \begin{aligned}&\hat{C}(t) = \sum _{m=-\infty }^\infty \hat{C}_m e^{im 2\pi t/T_\mathrm{mod}}, \\&\hat{C}_m = \frac{1}{T}\int _{0}^T dt\> \hat{C}(t) e^{-im 2\pi t/T_\mathrm{mod}}. \end{aligned} \end{aligned}$$

Here *T* is the total observation time, e.g., 1 sidereal year, which we will assume for simplicity is an integer multiple of $T_\mathrm{mod}$.

Assuming as usual that the detector noise is uncorrelated across detectors, and using the expectation values (7.1) for an unpolarized, anisotropic background, we find

7.18 $$\begin{aligned} \langle \hat{C}(t)\rangle =\frac{\tau }{2}\int _{-\infty }^\infty df\> \tilde{Q}(t;f)\bar{H}(f)\sum _{l=0}^\infty \sum _{m=-l}^l \gamma _{lm}(t;f) \mathcal{P}_{lm}, \end{aligned}$$

where $\gamma _{lm}(t;f)$ are the spherical harmonic components of $\gamma _{12}(t;f,\hat{n})$. (We have dropped the 12 indices to simplify the notation). Similarly, if we assume that the gravitational-wave signal is weak compared to the detector noise, and that the duration $\tau$ is also much larger than the correlation time of the detectors, then

7.19 $$\begin{aligned} \langle \hat{C}(t)\hat{C}^*(t')\rangle -\langle \hat{C}(t)\rangle \langle \hat{C}^*(t')\rangle \approx \frac{\tau }{4} \delta _{tt'}^2 \int _{-\infty }^\infty df\> |\tilde{Q}(t;f)|^2 P_{n_1}(t;f) P_{n_2}(t;f), \end{aligned}$$

where $P_{n_1}(t;f)$ is the one-sided power spectral density for the noise in detector $I=1,2$ centered around *t*. These two results can now be cast in terms of the Fourier components $\hat{C}_m$ using (7.17). Since (7.12) implies

7.20 $$\begin{aligned} \frac{1}{T}\int _0^T dt\> \gamma _{lm'}(t;f) e^{-im2\pi t/T_\mathrm{mod}} = \delta _{mm'}\,\gamma _{lm}(0;f), \end{aligned}$$

we immediately obtain

7.21 $$\begin{aligned} \langle \hat{C}_m\rangle =\frac{\tau }{2}\int _{-\infty }^\infty df\> \tilde{Q}(t;f)\bar{H}(f)\sum _{l=|m|}^\infty \gamma _{lm}(0;f)\mathcal{P}_{lm}, \end{aligned}$$

where we used

7.22 $$\begin{aligned} \sum _{l=0}^\infty \sum _{m=-l}^l = \sum _{m=-\infty }^\infty \sum _{l=|m|}^\infty . \end{aligned}$$

Similarly,

7.23 $$\begin{aligned} \langle \hat{C}_m\hat{C}^*_{m'}\rangle -\langle \hat{C}_m\rangle \langle \hat{C}^*_{m'}\rangle \approx \delta _{mm'}\frac{1}{T}\left( \frac{\tau }{2}\right) ^2 \int _{-\infty }^\infty df\> |\tilde{Q}(t;f)|^2 P_{n_1}(t;f) P_{n_2}(t;f) \end{aligned}$$

for the covariance of the estimators.

#### Calculation of the optimal filter

To determine the optimal form of the filter $\tilde{Q}(t;f)$ for the *m*th harmonic $\hat{C}_m$, we *maximize* the (squared) signal-to-noise:

7.24 $$\begin{aligned} \mathrm{SNR}_m^2 \equiv \frac{|\langle C_m\rangle |^2}{\langle |\hat{C}_m|^2\rangle -|\langle \hat{C}_m\rangle |^2} =\frac{T\left| \int _{-\infty }^\infty df\> \tilde{Q}(t;f)\bar{H}(f)\sum _{l=|m|}^\infty \gamma _{lm}(0;f)\mathcal{P}_{lm} \right| ^2}{\int _{-\infty }^\infty df\> |\tilde{Q}(t;f)|^2 P_{n_1}(t;f) P_{n_2}(t;f)}. \end{aligned}$$

The above expression can be written in a more suggestive form if we introduce an *inner product* on the space of complex-valued functions (Allen 1997):

7.25 $$\begin{aligned} (A,B)\equiv \int _{-\infty }^\infty df\> A(f)B^*(f) P_{n_1}(t;f) P_{n_2}(t;f). \end{aligned}$$

In terms of this inner product,

7.26 $$\begin{aligned}&\mathrm{SNR}_m^2 =\frac{T\left| \left( \tilde{Q}, \frac{\bar{H}}{P_{n_1}P_{n_2}}\sum _{l=|m|}^\infty \gamma _{lm}\mathcal{P}_{lm}\right) \right| ^2}{(\tilde{Q},\tilde{Q})}. \end{aligned}$$

But now the maximization problem is trivial, as it has been cast as a simple problem in vector algebra—namely to find the vector $\tilde{Q}$ that maximizes the ratio $|(\tilde{Q}, A)|^2/(\tilde{Q},\tilde{Q})$ for a *fixed* vector *A*. But since this ratio is proportional to the squared cosine of the angle between $\tilde{Q}$ and *A*, it is maximized by choosing $\tilde{Q}$ *proportional* to *A*. Thus,

7.27 $$\begin{aligned} \tilde{Q}(t;f) \propto \frac{\bar{H}(f)}{P_{n_1}(t;f) P_{n_2}(t;f)} \sum _{l=|m|}^\infty \gamma _{lm}(0;f)\mathcal{P}_{lm} \end{aligned}$$

is the form of the filter function that maximizes the SNR for the *m*th harmonic.

Note that this expression reduces to the standard form of the optimal filter (6.35) for an isotropic background, $\mathcal{P}_{lm} = \delta _{l0}\delta _{m0}\mathcal{P}_{00}$. Note also that the optimal filter assumes knowledge of both the spectral shape $\bar{H}(f)$ *and* the angular distribution of gravitational-wave power on the sky, $\mathcal{P}_{lm}$. So if one has some model for the expected anisotropy (e.g., a dipole in the same direction as the cosmic microwave background), then one can filter the cross-correlated data to be optimally sensitive to the harmonics $\hat{C}_m$ induced by that anisotropy.

#### Inverse problem

In Allen and Ottewill (1997), there was no attempt to solve the *inverse problem*—that is, given the *measured values* of the correlation harmonics, how can one *infer* (or *estimate*) the components $\mathcal{P}_{lm}$? The first attempt to solve the inverse problem was given in Cornish (2001), in the context of correlation measurements for both ground-based and space-based interferometers. Further developments in solving the inverse problem were given in subsequent papers, e.g., Ballmer (2006a, b), Mitra et al. (2008) and Thrane et al. (2009), which we explain in more detail in the following subsections. Basically, these latter methods constructed frequentist maximum-likelihood estimators for the $\mathcal{P}_{lm}$, using singular-value decomposition to ‘invert’ the Fisher matrix (or point spread function), which maps the true gravitational-wave power distribution to the measured distribution on the sky.

### Maximum-likelihood estimates of gravitational-wave power

In this section, we describe an approach for constructing maximum-likehood estimates of the gravitational-wave power distribution $\mathcal{P}(\hat{n})$. It is a solution to the inverse problem discussed at the end of the previous subsection. But since a network of gravitational-wave detectors typically does not have perfect coverage of the sky, the inversion requires some form of regularization, which we describe below. The gravitational-wave radiometer and spherical harmonic decomposition methods (Sect. 7.3.6) are the two main implementations of this approach, and have been used to analyze LIGO science data (Abadie et al. 2011; Abbott et al. 2016a).

#### Likelihood function and maximum-likelihood estimators

As shown in Sect. 7.1.3 the cross-correlated data from two detectors

7.28 $$\begin{aligned} \hat{C}_{IJ}(t; f) = \frac{2}{\tau }\tilde{d}_I(t;f)\tilde{d}_J^*(t;f) \end{aligned}$$

has expectation values

7.29 $$\begin{aligned} \langle \hat{C}_{IJ}(t; f)\rangle =\bar{H}(f) \int d^2\Omega _{\hat{n}} \> \gamma _{IJ}(t; f, \hat{n})\mathcal{P}(\hat{n}). \end{aligned}$$

We can write this relation abstractly as a matrix equation

7.30 $$\begin{aligned} \langle \hat{C}_{IJ}\rangle = M_{IJ}\,\mathcal{P}, \end{aligned}$$

where $M_{IJ}\equiv \bar{H}(f)\gamma _{IJ}(t;f,\hat{n})$ and the matrix product is summation over directions $\hat{n}$ on the sky. The covariance matrix for the cross-correlated data is given by

7.31 $$\begin{aligned} \begin{aligned} N_{tf,t'f'}&\equiv \langle \hat{C}_{IJ}(t;f) \hat{C}^*_{IJ}(t';f')\rangle -\langle \hat{C}_{IJ}(t;f)\rangle \langle \hat{C}^*_{IJ}(t';f')\rangle \\&\approx \delta _{tt'}\delta _{ff'} P_{n_I}(t; f) P_{n_J}(t; f), \end{aligned} \end{aligned}$$

where we have assumed as before that there is no cross-correlated detector noise, and that the gravitational-wave signal is weak compared to the detector noise.

If we treat the detector noise and the gravitational-wave spectral shape $\bar{H}(f)$ as known quantities (or if we estimate the detector noise from the auto-correlated output of each detector), then we can write down a likelihood function for the cross-correlated data given the signal model (7.30). Assuming a Gaussian-stationary distribution for the noise, we have

7.32 $$\begin{aligned} p(\hat{C}|\mathcal{P}) \propto \exp \left[ -\frac{1}{2} (\hat{C}- M\mathcal{P})^\dagger N^{-1} (\hat{C}- M\mathcal{P})\right] , \end{aligned}$$

where we have temporarily dropped the *IJ* indices for notational convenience.^21^ Since the gravitational-wave power distribution $\mathcal{P}$ enters quadratically in the exponential of the likelihood, we can immediately write down the maximum-likelihood estimators of $\mathcal{P}$:

7.33 $$\begin{aligned} \hat{\mathcal{P}} =F^{-1} X, \end{aligned}$$

where

7.34 $$\begin{aligned} F\equiv M^\dagger N^{-1} M, \quad X\equiv M^\dagger N^{-1} \hat{C}. \end{aligned}$$

The (square) matrix *F* is called the *Fisher information matrix*. It is typically a singular matrix, since the response matrix $M=\bar{H}\gamma$ usually has *null* directions (i.e., anisotropic distributions of gravitational-wave power that are mapped to zero by the detector response). Inverting *F* therefore requires some sort of regularization, such as singular-value decomposition (Press et al. 1992) (Sect. 7.3.5). The vector *X* is the so-called *dirty map*, as it represents the gravitational-wave sky as ‘seen’ by a pair of detectors. If the spectral shape $\bar{H}(f)$ that we used for our signal model exactly matches that of the observed background, then

7.35 $$\begin{aligned} \langle X\rangle = M^\dagger N^{-1} M \,\mathcal{P} = F\,\mathcal{P}. \end{aligned}$$

Thus, even in the absence of noise, a point source $\mathcal{P}(k) = \delta ^2(\hat{n},\hat{n}_0)$ does not map to a point source by the response of the detectors, but it maps instead to $F_{\hat{n}\hat{n}_0}$. This ‘blurring’ or ‘spreading’ of point sources is represented by a *point spread function*, which is a characteristic feature of any imaging system. We give plots of point spread functions for both pulsar timing arrays and ground-based interferometers in Sect. 7.3.4.

#### Extension to a network of detectors

The above results generalize to a *network* of detectors. One simply replaces *X* and *F* in (7.33) by their network expressions, which are simply sums of the dirty maps and Fisher matrices for each distinct detector pair:

7.36 $$\begin{aligned} X= \sum _I\sum _{J>I} X_{IJ}, \quad F= \sum _I\sum _{J>I} F_{IJ}. \end{aligned}$$

Explicit expressions for the dirty map and Fisher matrix for a network of detectors are:

7.37 $$\begin{aligned} X \equiv X_{\hat{n}} = \sum _I\sum _{J>I}\sum _t\sum _f \gamma _{IJ}^*(t;f,\hat{n}) \frac{\bar{H}(f)}{P_{n_I}(t;f)P_{n_J}(t,f)} \hat{C}_{IJ}(t; f), \end{aligned}$$

and

7.38 $$\begin{aligned} F \equiv F_{\hat{n}\hat{n}'} = \sum _I\sum _{J>I}\sum _t\sum _f \gamma _{IJ}^*(t;f,\hat{n}) \frac{\bar{H}^2(f)}{P_{n_I}(t;f)P_{n_J}(t,f)} \gamma _{IJ}(t; f,\hat{n}'). \end{aligned}$$

Note that including more detectors in the network is itself a form of regularization, as adding more detectors typically means better coverage of the sky. This tends to ‘soften’ the singularities that may exist when trying to deconvolve (i.e., invert) the detector response.

#### Error estimates

Using (7.35) it follows that $\hat{\mathcal{P}}$ is an unbiased estimator of $\mathcal{P}$:

7.39 $$\begin{aligned} \langle \hat{\mathcal{P}}\rangle = \mathcal{P}. \end{aligned}$$

Similarly, in the weak-signal approximation,

7.40 $$\begin{aligned} \begin{aligned}&\langle XX^\dagger \rangle - \langle X\rangle \langle X^\dagger \rangle \approx F, \\&\langle \hat{\mathcal{P}}\hat{\mathcal{P}}^\dagger \rangle - \langle \hat{\mathcal{P}}\rangle \langle \hat{\mathcal{P}}^\dagger \rangle \approx F^{-1}. \end{aligned} \end{aligned}$$

Thus, *F* is the covariance matrix for the dirty map *X*, while $F^{-1}$ is the covariance matrix of the clean map $\hat{\mathcal{P}}$. We will see below (Sect. 7.3.5) that regularization necessarily changes these results as one cannot recover modes of $\mathcal{P}$ to which the detector network is insensitive. This introduces a bias in $\hat{\mathcal{P}}$, and changes the corresponding elements of the covariance matrix for $\hat{\mathcal{P}}$.

#### Point spread functions

As discussed in the previous section, the point spread function for mapping gravitational-wave power is given by the components of the Fisher information matrix:

7.41 $$\begin{aligned} \mathrm{PSF}_{\hat{n}_0}(\hat{n}) \equiv \mathrm{PSF}(\hat{n}, \hat{n}_0) =F_{\hat{n} \hat{n}_0}. \end{aligned}$$

**Fig. 47:**
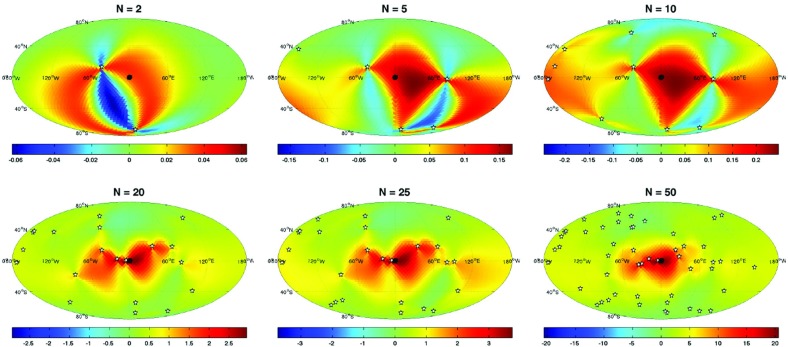
Point spread functions for gravitational-wave power for pulsar timing arrays consisting of $N=2$, 5, 10, 20, 25, 50 pulsars. The point source is located at the center of the maps, $(\theta ,\phi )=(90^\circ , 0^\circ )$, indicated by a *black dot*. The pulsar locations (indicated by *white stars*) are randomly placed on the sky. The point spread function becomes tighter as the number of pulsars in the array increases

**Fig. 48:**
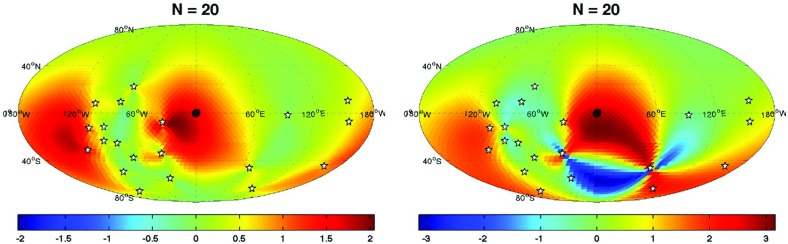
Point spread functions for the array of $N=20$ pulsars listed in Table 6 for both *equal-noise* weighting (*left panel*) and *actual-noise* weighting (*right panel*), using the timing noise values in the second column of the Table. The timing noise values were rescaled by an overall factor so that the maps for the two different weighting schemes could be meaningfully compared with one another. The point source is located at the center of the maps, indicated by a *black dot*

**Fig. 49:**
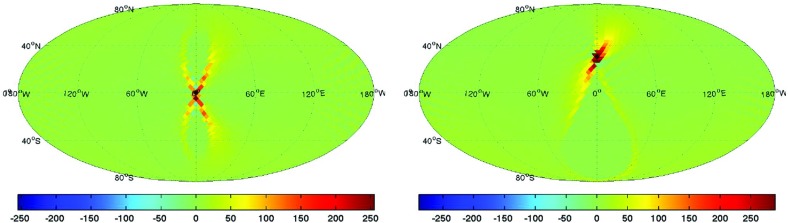
Point spread functions for gravitational-wave power for the LIGO Hanford–LIGO Livingston detector pair. *Left panel* point source at the center of the map, $(\theta ,\phi )=(90^\circ , 0^\circ )$. *Right panel* point source at $(\theta ,\phi )=(60^\circ ,0^\circ )$

Here $\hat{n}_0$ is the direction to the point source and $\hat{n}$ is an arbitrary point on the sky. In the following three figures (Figs. 47, 48, 49) we shows plots of point spread functions for both pulsar timing arrays and the LIGO Hanford–LIGO Livingston detector pair.

*Example: Pulsar timing arrays*

Figure 47 shows plots of point spread functions for pulsar timing arrays consisting of $N=2$, 5, 10, 20, 25, 50 pulsars. The point source is located at the center of the maps, indicated by a black dot. The pulsar locations (indicated by white stars) were randomly-distributed on the sky, and we used equal-noise weighting for calculating the point spread function. One can see that the point spread function becomes tighter as the number of pulsars in the array increases. Figure 48 are similar plots for an actual array of $N=20$ pulsars given in Table 6. Note that the pulsar locations are concentrated in the direction of the galactic center, $(\mathrm{ra},\mathrm{dec}) = (-6^\mathrm{h}15^\mathrm{m}, -29^\circ )$ in equatorial coordinates. The point source is again located at the center of the maps, indicated by a black dot. The left panel shows the point spread function calculated using equal-noise weighting, while the right panel shows the point spread function calculated using *actual-noise* weighting, based on the timing noise values given in the second column of Table 6. Note that this latter plot is similar to the small-*N* plots in Fig. 47, being dominated by pulsars with low timing noise—in this particular case, J0437−4715 and J2124−3358, which have the lowest and third-lowest timing noise.

**Table 6:** Actual pulsar locations and timing noise

Pulsar name	Timing noise ($\upmu$s)	Pulsar name	Timing noise ($\upmu$s)
J0437−4715	0.14	J1730−2304	0.51
J0613−0200	2.19	J1732−5049	1.81
J0711−6830	1.04	J1744−1134	0.17
J1022$+$1001	0.60	J1824−2452	3.62
J1024−0719	0.35	J1909−3744	0.56
J1045−4509	3.24	J1939$+$2134	3.58
J1600−3053	2.67	J2124−3358	0.25
J1603−7202	1.64	J2129−5721	2.55
J1643−1224	4.86	J2145−0750	0.50
J1713$+$0747	0.89	B1855$+$0900	0.70

The pulsar name specifies its location: the first four digits is right ascension (ra) in hours and minutes (hhmm); the last four digits is declination (dec) in degrees and minutes (ddmm), with the preceding $+$ or − sign. The rms timing noise is in microsec

*Example: Earth-based interferometers*

In Fig. 49 we plot point spread functions for gravitational-wave power for the LIGO Hanford-LIGO Livingston pair of detectors. The left-hand plot is for a point source located at the center of the map, $(\theta ,\phi )=(90^\circ , 0^\circ )$, while the right-hand plot is for a point source located at $(\theta ,\phi )=(60^\circ , 0^\circ )$ (indicated by black dots). We assumed equal white-noise power spectra for the two detectors, and we combined the contributions from 100 discrete frequencies between 0 and 100 Hz, and 100 discrete time chunks over the course of one sidereal day. The point spread functions for the two different point source locations are shaped, respectively, like a *figure-eight* with a bright region at the center of the figure-eight pattern, and a *tear drop* with a bright region near the top of the drop. These results are in agreement with Mitra et al. (2008) (see e.g., Fig. 1 in that paper). Provided one combines data over a full sidereal day, the point spread function is independent of the right ascension (i.e., azimuthal) angle of the source. Readers should see Mitra et al. (2008) for more details, including a stationary phase approximation for calculating the point spread function.

*Angular resolution estimates*

There are “rules of thumb” that can be used to estimate the angular resolution $\Delta \theta$ (or size of a point spread function) for an anisotropic stochastic background search. For cross-correlations using ground-based interferometers like LIGO, Virgo, etc., the angular resolution of the detector network can be estimated from the diffraction limit (Monnier 2003):

7.42 $$\begin{aligned} \Delta \theta \simeq \frac{\lambda }{2D} = \frac{c}{2fD}, \end{aligned}$$

where *f* is gravitational-wave frequency and *D* is separation between a pair of detectors. Thus, the larger the separation between detectors and the higher frequencies searched for, the better the angular resolution. For a pulsar timing array consisting of *N* pulsars, the corresponding estimate is given by

7.43 $$\begin{aligned} \Delta \theta \simeq 180^\circ /l_\mathrm{max} \simeq 180^\circ /\sqrt{N}, \end{aligned}$$

where $l_\mathrm{max}$ is the maximum value of *l* for a spherical harmonic decomposition of the background having angular features of size $\Delta \theta$. The last approximate equality follows from the fact that, at each frequency, one can extract at most *N* (complex) pieces of information about the gravitational-wave background using an *N*-pulsar array (Boyle and Pen 2012; Cornish and van Haasteren 2014; Gair et al. 2014); and those *N* pieces of information correspond to the number of spherical harmonic components (*lm*) out to $l_\mathrm{max}$, so $N\sim l_\mathrm{max}^2$. (We will discuss this again in Sect. 7.5.4, in the context of *basis skies* for a phase-coherent search for anisotropic backgrounds). Note that if we knew the distances to the pulsars in the array and used information from the pulsar-term contribution to the timing residuals (5.17), then $\Delta \theta$ for a pulsar timing array would have the same form as (7.42), but with *D* now representing the Earth-pulsar distance. See Boyle and Pen (2012) for details.

#### Singular-value decomposition

Expression (7.33) for the maximum-likelihood estimator $\hat{\mathcal{P}}$ involves the inverse of the Fisher matrix *F*. But this is just a *formal* expression, as *F* is typically a singular matrix, requiring some sort of regularization to invert. Here we describe how *singular-value decomposition* (Press et al. 1992) can be used to ‘invert’ *F*. Since this a general procedure, we will frame our discussion in terms of an arbitrary matrix *S*.

Singular value decomposition factorizes an $n\times m$ matrix *S* into the product of three matrices:

7.44 $$\begin{aligned} S = U\Sigma V^\dagger , \end{aligned}$$

where *U* and *V* are $n\times n$ and $m\times m$ unitary matrices, and $\Sigma$ is an $n\times m$ rectangular matrix with (real, non-negative) singular values $\sigma _k$ along its diagonal, and with zeros everywhere else. We will assume, without loss of generality, that the singular values are arranged from largest to smallest along the diagonal. We define the *pseudo-inverse* $S^+$ of *S* as

7.45 $$\begin{aligned} S^+ \equiv V\Sigma ^+ U^\dagger , \end{aligned}$$

where $\Sigma ^+$ is obtained by taking the reciprocal of each nonzero singular value of $\Sigma$, leaving all the zeros in place, and then transposing the resulting matrix. Note that when *S* is a square matrix with non-zero determinant, then the pseudo-inverse $S^+$ is identical to the ordinary matrix inverse $S^{-1}$. Thus, the pseudo-inverse of a matrix generalizes the notion of ordinary inverse to non-square or singular matrices.

As a practical matter, it is important to note that if the nonzero singular values of $\Sigma$ vary over several orders of magnitude, it is usually necessary to first set to zero (by hand) all nonzero singular values $\le$ some minimum threshold value $\sigma _\mathrm{min}$ (e.g., $10^{-5}$ times that of the largest singular value). Alternatively, we can set those very small singular values equal to the threshold value $\sigma _\mathrm{min}$. This procedure helps to reduce the noise in the maximum-likelihood estimates, which is dominated by the modes to which we are least sensitive.

Returning to the gravitational-wave case, the above discussion means that all of the previous expressions for the inverse of the Fisher matrix, $F^{-1}$, should actually be written in terms of the pseudo-inverse $F^+$. Thus,

7.46 $$\begin{aligned} \hat{\mathcal{P}} = F^+ X, \end{aligned}$$

which then implies

7.47 $$\begin{aligned} \begin{aligned}&\langle \hat{\mathcal{P}}\rangle = F^+ F\,\mathcal{P}, \\&\langle \hat{\mathcal{P}}\hat{\mathcal{P}}^\dagger \rangle - \langle \hat{\mathcal{P}}\rangle \langle \hat{\mathcal{P}}^\dagger \rangle \approx F^+. \end{aligned} \end{aligned}$$

So $\hat{\mathcal{P}}$ is actually a *biased* estimator of $\mathcal{P}$ if $F^+\ne F^{-1}$, as was discussed in Thrane et al. (2009).

**Fig. 50:**
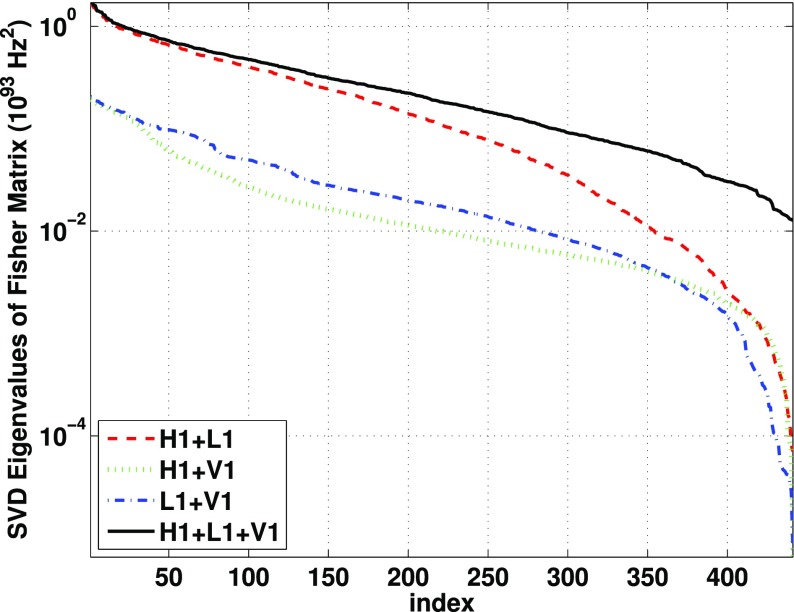
Singular values of typical Fisher matrices *F* for different ground-based interferometer detector pairs and a multibaseline detector network. For this analysis there were 441 total modes. For each individual detector pair, some of the singular values are (almost) null. The multibaseline network has fewer null modes, thus acting as a natural regularizer. Image reproduced with permission from Thrane et al. (2009), copyright by APS

Figure 50 is a plot of the singular values of typical Fisher matrices for different ground-based interferometer detector pairs (Hanford–Livingston, Hanford–Virgo, Livingston–Virgo) and a multibaseline detector network (Hanford–Livingston–Virgo). For these examples, we chose to expand the gravitational-wave power on the sky $\mathcal{P}(\hat{n})$ and the integrand of the overlap functions $\gamma _{IJ}(t;f,\hat{n})$ in terms of spherical harmonics out to $l_\mathrm{max}=20$. (See Sect. 7.3.6 for more details about the spherical harmonic decomposition method). This yields $(l_\mathrm{max}+1)^2 = 441$ modes of gravitational-wave sky that we would like to recover. Note how the inclusion of more detectors to the network reduces the dynamic range of the singular values of *F*, hence making the matrix less singular without any external form of regularization.

#### Radiometer and spherical harmonic decomposition methods

The gravitational-wave radiometer (Ballmer 2006a, b; Mitra et al. 2008) and spherical harmonic decomposition methods (Thrane et al. 2009; Abadie et al. 2011) are two different ways of implementing the maximum-likelihood approach for mapping gravitational-wave power $\mathcal{P}(\hat{n})$. They differ primarily in their choice of signal model, and their approach for deconvolving the detector response from the underlying (true) distribution of power on the sky.

*Gravitational-wave radiometer*

The radiometer method takes as its signal model a point source characterized by a direction $\hat{n}_0$ and amplitude $\mathcal{P}_{\hat{n}_0}$:

7.48 $$\begin{aligned} \mathcal{P}(\hat{n}) = \mathcal{P}_{\hat{n}_0}\,\delta ^2(\hat{n},\hat{n}_0). \end{aligned}$$

It is applicable to an anisotropic gravitational-wave background dominated by a limited number of widely-separated point sources. As the number of point sources increases or if two point sources are sufficiently close to one another, the point spread function for the detector network will cause the separate signals to interfere with one another. Thus, the radiometer method is not appropriate for diffuse backgrounds. Moreover, by assuming that the signal is point-like, the radiometer method ignores correlations between neighboring pixels on the sky, effectively side-stepping the deconvolution problem. Explicitly, the inverse of the Fisher matrix that appears in the maximum-likelihood estimator $\hat{\mathcal{P}}=F^{-1} X$ is replaced by the inverse of the *diagonal element* $F_{\hat{n}\hat{n}}$ to obtain an estimate of the point-source amplitude at $\hat{n}$:

7.49 $$\begin{aligned} \hat{\mathcal{P}}_{\hat{n}} = (F_{\hat{n}\hat{n}})^{-1} X_{\hat{n}}, \end{aligned}$$

where *X* is the dirty map (7.34). Thus, the radiometer method estimates the strength of point sources at different points on the sky, *ignoring* any correlations between neighboring pixels.

Note that for a single pair of detectors *IJ* the above estimator (7.49) is equivalent to an appropriately normalized cross-correlation statistic:

7.50 $$\begin{aligned} \hat{C}_{IJ}(t; \hat{n}) \equiv \int _{-\infty }^\infty df\> Q_{IJ}(t; f,\hat{n}) \tilde{d}_I(t;f) \tilde{d}_J^*(t;f), \end{aligned}$$

with filter function

7.51 $$\begin{aligned} Q_{IJ}(t;f,\hat{n}) \propto \frac{\gamma _{IJ}(t;f,\hat{n})\, \bar{H}(f)}{P_{n_I}(t;f) P_{n_J}(t;f)}, \end{aligned}$$

where $\gamma _{IJ}$ is given by (7.8). For a network of detectors, one recovers the estimator $\hat{\mathcal{P}}_{\hat{n}}$ by summing the individual-baseline statistics (7.50) over both time and distinct detector pairs, weighted by the inverse variances of the individual-baseline statistics. See e.g., Ballmer (2006a, b) and Mitra et al. (2008) for more details.

*Spherical harmonic decomposition*

The spherical harmonic decomposition method is appropriate for extended anisotropic distributions on the sky, assuming a signal model for gravitational-wave power that includes spherical harmonic components up to some specified value of $l_\mathrm{max}$:

7.52 $$\begin{aligned} \mathcal{P}(\hat{n}) = \sum _{l=0}^{l_\mathrm{max}}\sum _{m=-l}^l \mathcal{P}_{lm} Y_{lm}(\hat{n}). \end{aligned}$$

The cutoff in the expansion at $l_\mathrm{max}$ corresponds to an angular scale $\Delta \theta \simeq 180^\circ /l_\mathrm{max}$. The diffraction limit (Monnier 2003):

7.53 $$\begin{aligned} \Delta \theta \simeq \frac{\lambda }{2D} = \frac{c}{2f D}, \end{aligned}$$

where *f* is the maximum gravitational-wave frequency and *D* is the separation between a pair of detectors, sets an upper limit on the size of $l_\mathrm{max}$, since the detector network is not able to resolve features having smaller angular scales. For example, for the LIGO Hanford–LIGO Livingston detector pair ($D= 3000\ \mathrm{km}$) and a stochastic background having contributions out to $f\sim 500~\mathrm{Hz}$, we find $l_\mathrm{max} \lesssim 30$. Alternatively, one can use Bayesian model selection to determine the value of $l_\mathrm{max}$ that is most consistent with the data.

Since the spherical harmonic method targets extended distributions of gravitational-wave power on the sky, correlations between neighboring pixels or, equivalently, between different spherical harmonic components must be taken into account. This is addressed by using singular-value decomposition as described in Sect. 7.3.5 to ‘invert’ the Fisher matrix. By effectively ignoring those modes to which the detector network is insensitive, we can construct the pseudo-inverse $F^+$ to perform the deconvolution. In terms of $F^+$, we have

7.54 $$\begin{aligned} \hat{\mathcal{P}}_{lm} = \sum _{l'=0}^{l_\mathrm{max}} \sum _{m'=-l'}^{l'} F^+_{lm,l'm'} X_{l'm'} \end{aligned}$$

for the spherical harmonic components of the maximum-likelihood estimators $\hat{\mathcal{P}}$. The sky map constructed from the $\hat{\mathcal{P}}_{lm}$ is called a ‘clean’ map, since the inversion removes the detector response from the ‘dirty’ map *X*.

**Fig. 51:**
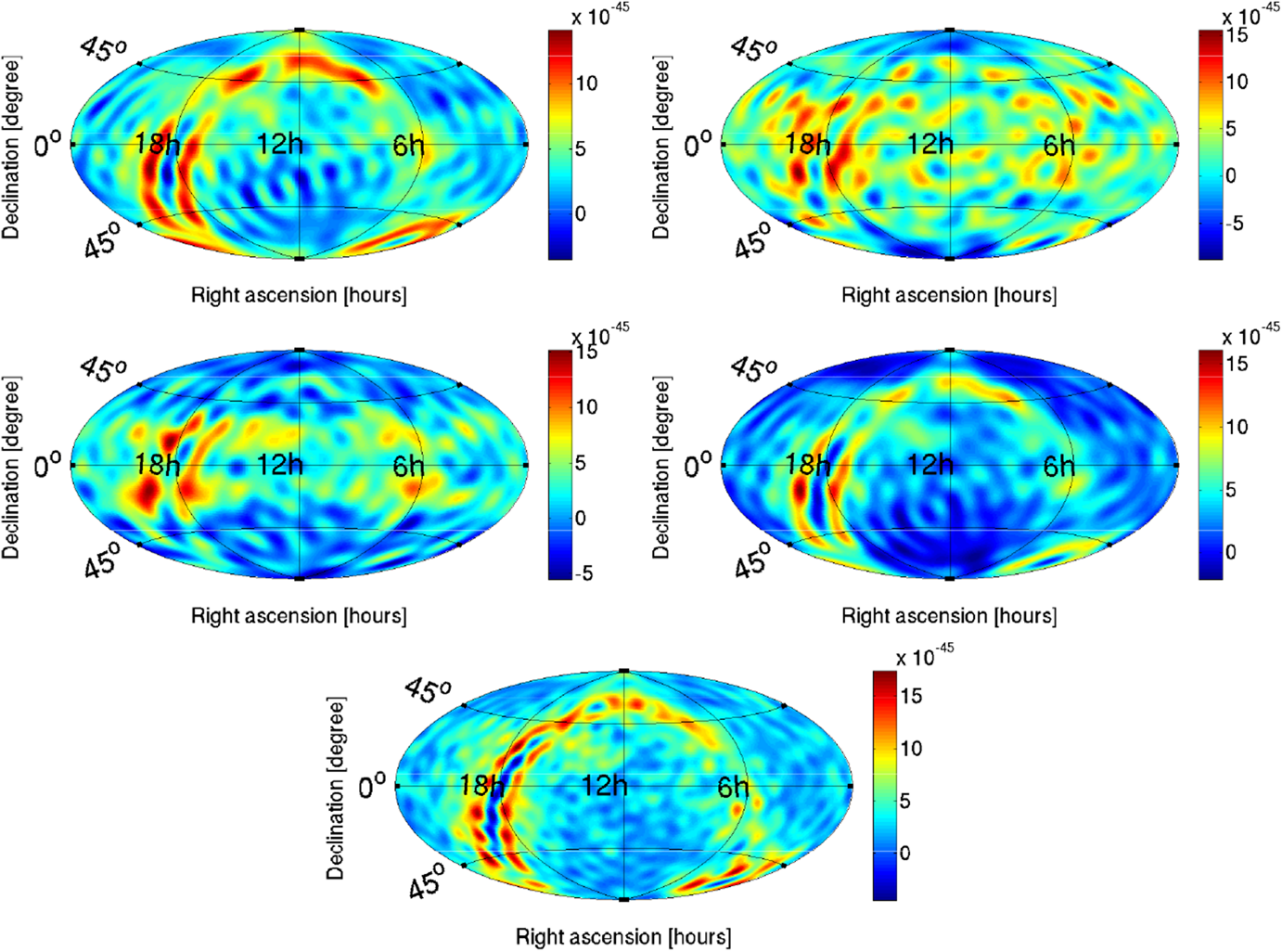
Results of spherical harmonic decomposition analyses performed using different detector pairs and a multibaseline detector network. The simulated anisotropic power distribution is shown in the bottom plot. *Top row* clean maps for the Hanford–Livingston and Hanford–Virgo detector pairs. *Second row* same as the *top row*, but for the Livingston–Virgo detector pair and for the Hanford–Livingston–Virgo multibaseline detector network. For all maps $l_\mathrm{max}=20$. Image reproduced with permission from Thrane et al. (2009), copyright by APS

**Fig. 52:**
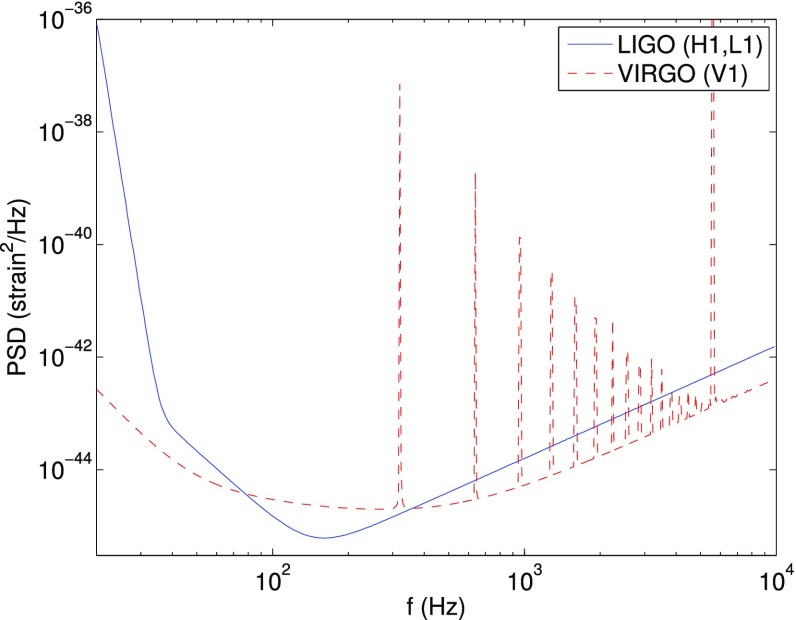
The power spectral densities used for the simulated detector noise for the injections described in Fig. 51. Image reproduced with permission from Thrane et al. (2009), copyright by APS

Figure 51 shows clean maps produced by the spherical harmonic decomposition method for a simulated anisotropic background distributed along the galactic plane (Thrane et al. 2009). The injected map is the bottom plot in the figure. (All sky maps are in equatorial coordinates). The four maps shown in the top two rows of the figure correspond to analyses with different interferometer detector pairs (Hanford–Livingston, Hanford–Virgo, and Livingston–Virgo) and a multibaseline detector network (Hanford–Livingston–Virgo). Consistent with our findings in Fig. 50, we see that the recovered map is best for the multibaseline network, whose Fisher matrix has singular values with the smallest dynamic range. For the reconstructed maps, $F^+$ was calculated by keeping 2/3 of all the eigenmodes (those with the largest singular values), setting the remaining singular values equal to the minimum value $\sigma _\mathrm{min}$ of the modes that were kept. For all cases, $l_\mathrm{max}=20$. The anisotropic background was injected into simulated LIGO and Virgo detector noise (initial design sensitivity) whose power spectra are shown in Fig. 52. The overall amplitude of the signal was chosen to be large enough that it was easily detectable in 1 sidereal day’s worth of simulated data. For additional details see Thrane et al. (2009).

### Frequentist detection statistics

As discussed in Sects. 3.4 and 4.4, one can construct a frequentist detection statistic $\Lambda _\mathrm{ML}(d)$ by taking the ratio of the maxima of the likelihood functions for the signal-plus-noise model to the noise-only model. The logarithm,

7.55 $$\begin{aligned} \Lambda (d) \equiv 2\, \ln [\Lambda _\mathrm{ML}(d)], \end{aligned}$$

is the squared signal-to-noise ratio of the data. If we calculate this quantity for an anisotropic background $\mathcal{P}(\hat{n})$ using (7.32) for the signal-plus-noise model, we find

7.56 $$\begin{aligned} \Lambda (d) = \hat{\mathcal{P}}^\dagger F \hat{\mathcal{P}}, \end{aligned}$$

where $\hat{\mathcal{P}}$ are the maximum-likelihood estimators of $\mathcal{P}$. As described in Sect. 3.2.1, one can use this statistic to do frequentist hypothesis testing, comparing its observed value $\Lambda _\mathrm{obs}$ to a threshold $\Lambda _*$ to decide whether or not to claim detection of a signal.

The above detection statistic can be written in several alternative forms:

7.57 $$\begin{aligned} \Lambda (d) = \hat{\mathcal{P}}^\dagger F \hat{\mathcal{P}} = X^\dagger F^{-1} X = \frac{1}{2}\left( \hat{\mathcal{P}}^\dagger X + X^\dagger \hat{\mathcal{P}}\right) , \end{aligned}$$

where *X* is the ‘dirty’ map, which is related to $\hat{\mathcal{P}}$ via $\hat{\mathcal{P}} = F^{-1} X$. The last form suggests a standard matched-filter statistic:

7.58 $$\begin{aligned} \lambda (d) \equiv \frac{1}{2}\left( \bar{\mathcal{P}}^\dagger _\mathrm{model} X + X^\dagger \bar{\mathcal{P}}_\mathrm{model} \right) , \end{aligned}$$

where $\bar{\mathcal{P}}_\mathrm{model}$ is an *assumed* distribution of gravitational-wave power on the sky, normalized such that

7.59 $$\begin{aligned} \bar{\mathcal{P}}^\dagger _\mathrm{model} F \bar{\mathcal{P}}_\mathrm{model}=1. \end{aligned}$$

The above normalization is chosen so that if the true gravitational-wave background has the same spectral shape $\bar{H}(f)$ and the same angular distribution $\bar{\mathcal{P}}_\mathrm{model}$, then $\lambda (d)$ is an estimator of the overall amplitude of the background. In the absence of a signal, $\lambda (d)$ has zero mean and unit variance.

Such a matched-filter statistic was proposed in Appendix C of Thrane et al. (2009) and studied in detail in Talukder et al. (2011). One nice property of this statistic is that it does not require inverting the Fisher matrix. Hence it avoids the inherent bias (7.47) and introduction of other uncertainties associated with the deconvolution process. Indeed, if we are *given* a model of the expected anisotropy, $\lambda (d)$ is the *optimal* statistic for detecting its presence. Thus, $\lambda (d)$ is especially good at detecting weak anisotropic signals. See Talukder et al. (2011) for more details.

### Phase-coherent mapping

Phase-coherent mapping is an approach that constructs estimates of both the amplitude and phase of the gravitational-wave background at each point of the sky (Cornish and van Haasteren 2014; Gair et al. 2014; Romano et al. 2015). In some sense, it can be thought of as the “square root” of the approaches described in the previous subsections, which attempt to measure the distribution of gravitational-wave *power* $\mathcal{P}(\hat{n}) = |h_+|^2 + |h_\times ^2|$. The gravitational-wave signal can be characterized in terms of either the standard polarization basis components $\{h_+(f,\hat{n}), h_\times (f,\hat{n})\}$ or the tensor spherical harmonic components $\{a^G_{(lm)}(f), a^C_{(lm)}(f)\}$. In what follows we will restrict our attention the polarization basis components, although a similar analysis can be carried out in terms of the spherical harmonic components (Gair et al. 2014).

#### Maximum-likelihood estimators and Fisher matrix

Unlike the previous approaches, which target gravitational-wave power and hence use cross-correlations (7.6) as their fundamental data product, phase-coherent mapping works directly with the data from the individual detectors. In terms of the short-term Fourier transforms defined in Sect. 7.1.2, we can write

7.60 $$\begin{aligned} \tilde{d}_{I}(t;f) =\int d^2\Omega _{\hat{n}} \sum _A R^A_{I}(t; f,\hat{n}) h_A(f,\hat{n}) + \tilde{n}_{I}(t;f), \end{aligned}$$

where *I* labels the different detectors, and $\tilde{n}_I(t;f)$ denotes the corresponding detector noise. Given our assumption (7.3) that the spectral and angular dependence of the background factorize with known spectral function $\bar{H}(f)$, we can rewrite the above equation as

7.61 $$\begin{aligned} \tilde{d}_{I}(t; f) =\int d^2\Omega _{\hat{n}}\> \bar{H}^{1/2}(f) \sum _A R^A_{I}(t; f, \hat{n}) h_A(\hat{n}) + \tilde{n}_{I}(t;f), \end{aligned}$$

so that the only unknowns are $\{h_+(\hat{n}), h_\times (\hat{n})\}$ at different locations on the sky. We will write this equation abstractly as a matrix equation

7.62 $$\begin{aligned} d = M a + n, \end{aligned}$$

where

7.63 $$\begin{aligned} M \equiv \{\bar{H}^{1/2}(f) R_I^A(t;f,\hat{n})\}, \quad a \equiv \{h_A(\hat{n})\}. \end{aligned}$$

The matrix multiplication corresponds to a sum over polarizations *A* and directions $\hat{n}$ on the sky.

Assuming that the noise is uncorrelated across detectors, the noise covariance matrix is given by:

7.64 $$\begin{aligned} \begin{aligned} N_{Itf,I't'f'}&\equiv \langle \tilde{n}_{I}(t;f)\tilde{n}^*_{I'}(t';f')\rangle -\langle \tilde{n}_{I}(t;f)\rangle \langle \tilde{n}^*_{I'}(t';f')\rangle \\&=\frac{\tau }{2}\delta _{II'}\delta _{tt'}\delta _{ff'} P_{n_{I}}(t;f), \end{aligned} \end{aligned}$$

where $P_{n_{I}}(t;f)$ is the one-sided power spectral density of the noise in detector *I* at time *t*. Thus, we can write down a likelihood function for the data $d\equiv \{\tilde{d}_I(t;f)\}$ given *a*:

7.65 $$\begin{aligned} p(d|a) \propto \exp \left[ -\frac{1}{2}(d - Ma)^\dagger N^{-1} (d- Ma)\right] \, \end{aligned}$$

where the multiplications inside the exponential are matrix multiplications, involving summations over detectors *I*, times *t*, and frequencies *f*, or summations over polarizations *A* and directions $\hat{n}$ on the sky. Note that (7.65) has exactly the same form as (7.32), so the same general remarks made in Sect. 7.3.1 apply here as well. Namely, the maximum-likelihood estimators of *a* are

7.66 $$\begin{aligned} \hat{a} =F^{-1} X, \end{aligned}$$

where

7.67 $$\begin{aligned} F\equiv M^\dagger N^{-1} M, \quad X\equiv M^\dagger N^{-1} d, \end{aligned}$$

are the Fisher matrices and ‘dirty’ maps for this analysis. (The definitions of *M*, *N* here are different, of course, from those in Sect. 7.3.1). Explicit expression for *X* and *F* are given below:

7.68 $$\begin{aligned} X\equiv X_{A\hat{n}} =\frac{2}{\tau }\sum _{I}\sum _t\sum _f R^{A*}_{I}(t;f,\hat{n}) \frac{\bar{H}^{1/2}(f)}{P_{n_{I}}(f)} \tilde{d}_{I}(t;f), \end{aligned}$$

and

7.69 $$\begin{aligned} F\equiv F_{A\hat{n},A'\hat{n}'} =\frac{2}{\tau }\sum _{I}\sum _t\sum _f R^{A*}_{I}(t;f,\hat{n}) \frac{\bar{H}(f)}{P_{n_{I}}(f)} R^{A'}_{I}(t;f,\hat{n}'). \end{aligned}$$

Note that these expressions have an extra polarization index *A*, compared to the corresponding expressions, (7.37) and (7.38), for gravitational-wave power.

#### Point spread functions

The point spread function for the above analysis can now be obtained by fixing values for both $A'$ and $\hat{n}'$, and letting *A* and $\hat{n}$ vary. Since there are two polarization modes ($+$ and $\times$), there are actually *four* different point spread functions for each direction $\hat{n}'$ on the sky:

7.70 $$\begin{aligned} \mathrm{PSF}_{AA'}(\hat{n}, \hat{n}') = F_{A\hat{n},A'\hat{n}'}. \end{aligned}$$

These correspond to the $A=+,\times$ responses to the $A'=+,\times$-polarized point sources located in direction $\hat{n}'$.

**Fig. 53:**
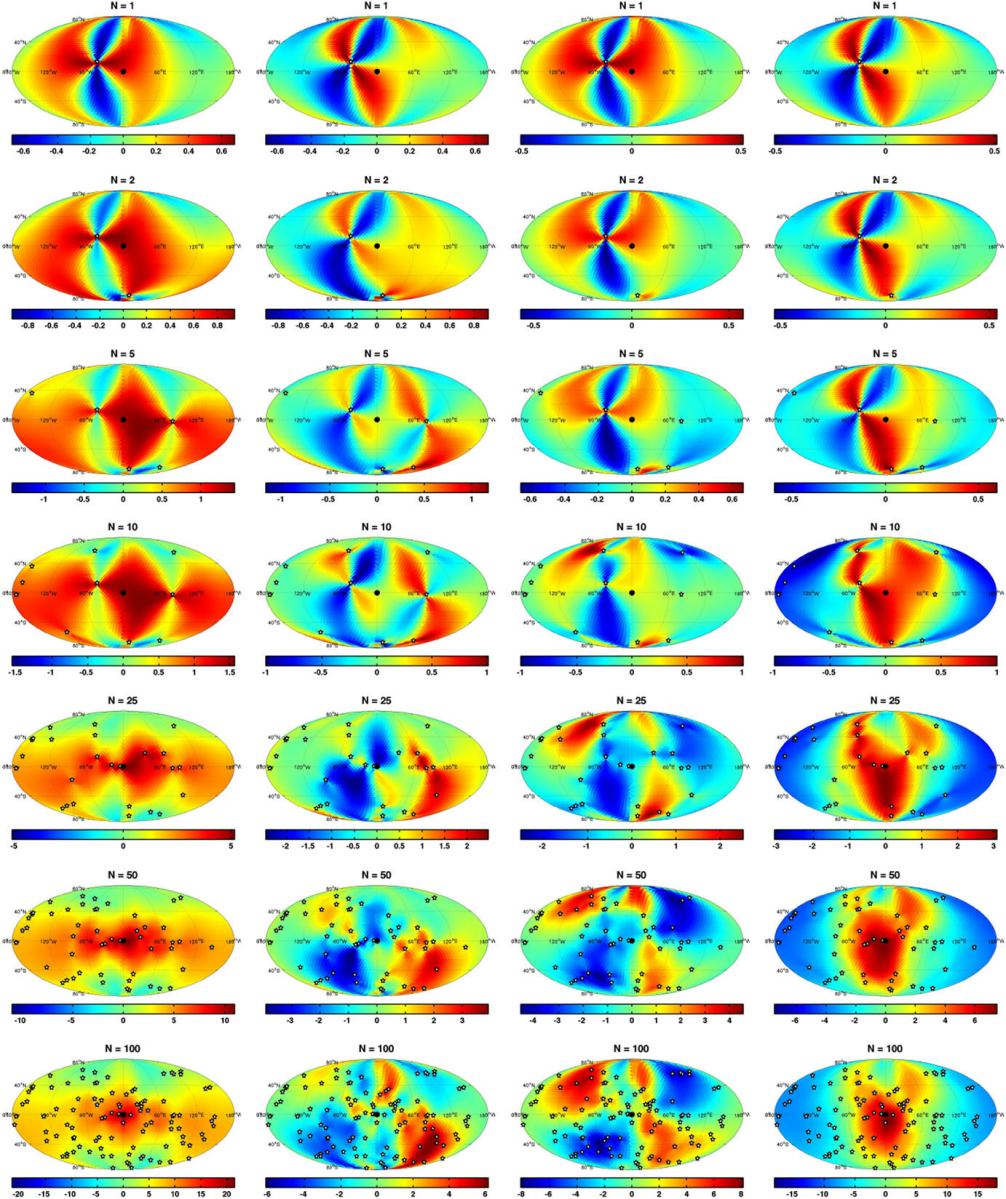
Point spread functions for phase-coherent mapping, for pulsar timing arrays consisting of $N=1$, 2, 5, 10, 25, 50, 100 pulsars. The point source is located at the center of the maps, $(\theta ,\phi )=(90^\circ , 0^\circ )$, indicated by a *black dot*. The pulsar locations (indicated by *white stars*) are randomly placed on the sky. Different rows correspond to different numbers of pulsars in the array. Columns 1 and 2 correspond to the $+$ and $\times$ response of the pulsar timing array to a $+$-polarized point source; columns 3 and 4 correspond to the $+$ and $\times$ response of the pulsar timing array to a $\times$-polarized point source

To illustrate the above procedure, we calculate point spread functions for phase-coherent mapping, for pulsar timing arrays consisting of $N=1$, 2, 5, 10, 25, 50, 100 pulsars. Figure 53 show plots of these point spread functions. The pulsars are randomly distributed over the sky (indicated by white stars), and the point source is located at the center of the maps (indicated by a black dot). For simplicity, we assumed a single frequency bin, and used equal-noise weighting for calculating the point spread functions. (In addition, there is no time dependence as the directions to the pulsars are fixed on the sky). Different rows in the figure correspond to different numbers of pulsars in the array. Different columns correspond to different choices for *A* and $A'$: columns 1, 2 correspond to the $A=+,\times$ response of the pulsar timing array to an $A'=+$-polarized point source; columns 3, 4 correspond to the $A=+,\times$ response of the pulsar timing array to an $A'=\times$-polarized point source. Note that for $N=1$, the point spread functions are proportional to either $R_I^+(\hat{n})$ or $R_I^\times (\hat{n})$ for that pulsar, producing maps similar to those shown in Fig. 27. As *N* increases the $++$ and $\times \times$ point spread functions (columns 1 and 4) become tighter around the location of the point source, which is at the center of the maps. But since the $+$ and $\times$ polarizations are orthogonal, the $\times +$ and $+\times$ point spread functions (columns 2 and 3) have values close to zero around the location of the point source.

#### Singular value decomposition

Just as we had to deconvolve the detector response in order to obtain the estimators $\hat{\mathcal{P}}$ for gravitational-wave power, we need to do the same for the estimators $\hat{a}$ for the phase-coherent mapping approach. Although we could use singular-value decomposition for the Fisher matrix *F* given by (7.69), we will first *whiten* the data, which leads us directly to pseudo-inverse of the whitened response matrix *M*, (7.63). This is the approach followed in Cornish and van Haasteren (2014) and Romano et al. (2015), and it leads to some interesting results regarding *sky-map basis vectors*, which we will describe in more detail in Sect. 7.5.4. An alternative approach involving the pseudo-inverse of the unwhitened response matrix is given in Gair et al. (2014) and Appendix B of Romano et al. (2015).

To whiten the data, we start by finding the Cholesky decomposition of the inverse noise covariance matrix $N^{-1} = LL^\dagger$, where *L* is a lower triangular matrix. The whitened data are then given by $\bar{d} = L^\dagger d$ (since this has unit covariance matrix), and the whitened response matrix is given by $\bar{M} = L^\dagger M$. In terms of these whitened quantities,

7.71 $$\begin{aligned} F=\bar{M}^\dagger \bar{M}, \quad X= \bar{M}^\dagger \bar{d}, \end{aligned}$$

implying

7.72 $$\begin{aligned} \hat{a} = F^{-1} X = (\bar{M}^\dagger \bar{M})^{-1} M^\dagger \bar{d} \equiv \bar{M}^+ \bar{d}. \end{aligned}$$

The last equality is a formal expression for the pseudo-inverse $\bar{M}^+$ since $\bar{M}^\dagger \bar{M}$ is not necessarily invertible. But as shown in Sect. 7.3.5 it is *always* possible to define the pseudo-inverse of a matrix in terms of its singular-value decomposition. Thus, given the singular-value decomposition:

7.73 $$\begin{aligned} \bar{M} = U \Sigma V^\dagger , \end{aligned}$$

we have

7.74 $$\begin{aligned} \bar{M}^+ = V\Sigma ^+ U^\dagger , \end{aligned}$$

where $\Sigma ^+$ is defined by the procedure described in Sect. 7.3.5. Thus,

7.75 $$\begin{aligned} \hat{a} = \bar{M}^+ \bar{d} = V\Sigma ^+ U^\dagger \bar{d}. \end{aligned}$$

This is the expression we need to compute to calculate the maximum-likelihood estimators $\hat{a}$ for the phase-coherent mapping approach.

**Fig. 54:**
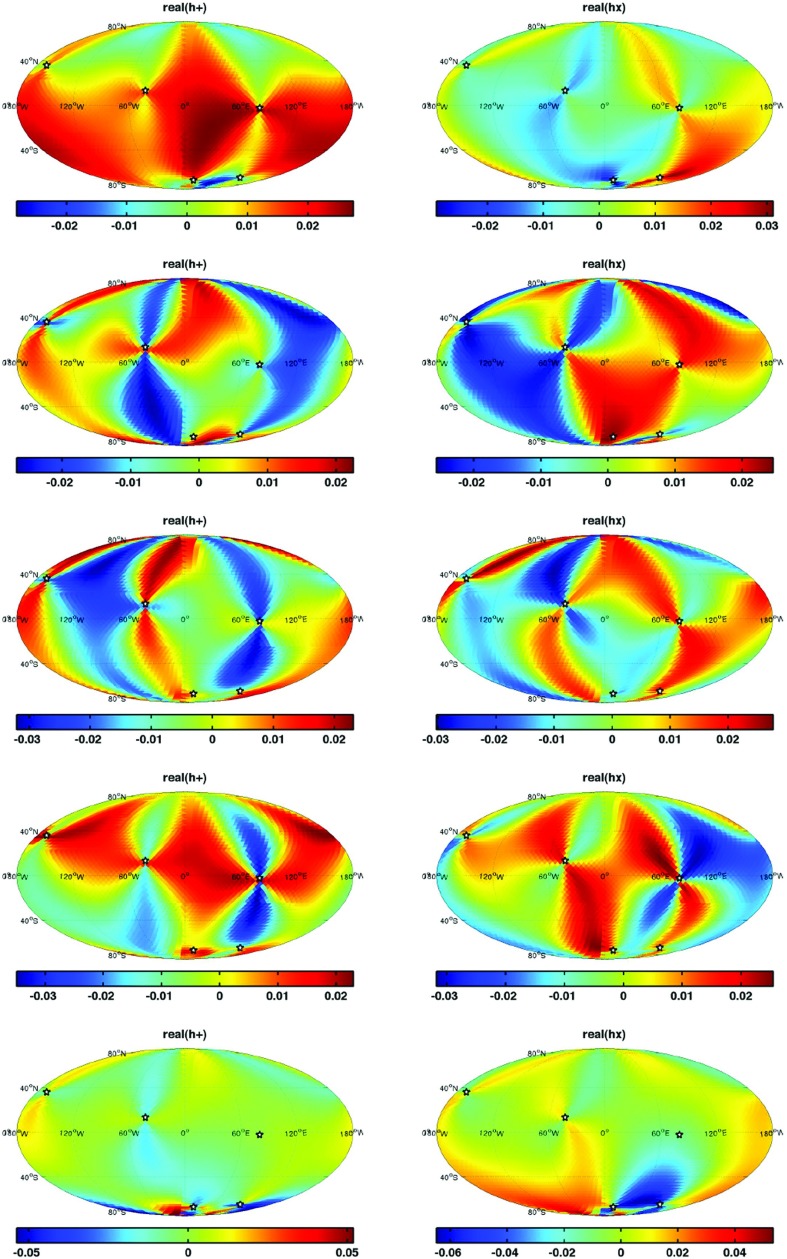
The real parts of the $+$ and $\times$-polarization basis skies for pulsar timing array consisting of $N=5$ pulsars randomly distributed on the sky. The imaginary components of the basis skies are identically zero. The basis skies are shown in decreasing size of their singular values, from the *top* of the figure to the *bottom*

#### Basis skies

The singular-value decomposition of $\bar{M}$ also has several nice geometrical properties. For example, from (7.75), we see that the columns of *V* corresponding to the non-zero singular values of $\Sigma$ are *basis vectors* (which we will call *basis skies*) in terms of which $\hat{a}$ can be written as a linear combination. Similarly, if write the whitened response to the gravitational-wave background as

7.76 $$\begin{aligned} \bar{M} a = U\Sigma V^\dagger a, \end{aligned}$$

then we see that the columns of *U* corresponding to the non-zero singular values of $\Sigma$ can be interpreted as *range vectors* for the response. To be more explicit, let $u_{(k)}$ and $v_{(k)}$ denote the *k*th columns of *U* and *V*, and let *r* be the number of non-zero singular values of $\Sigma$. Then

7.77 $$\begin{aligned} \begin{aligned} \hat{a}&= \sum _{k=1}^r \sigma _k^{-1} (u_{(k)}\cdot \bar{d})\, v_{(k)}, \\ \bar{M} a&= \sum _{k=1}^r \sigma _k (v_{(k)}\cdot a)\, u_{(k)}, \end{aligned} \end{aligned}$$

where the dot product of two vectors *a* and *b* is defined as $a\cdot b = a^\dagger b$. If we further expand $\bar{d} = \bar{M} a + \bar{n}$ in the first of these equations, then

7.78 $$\begin{aligned} \hat{a} = \sum _{k=1}^r (v_{(k)}\cdot a) v_{(k)} +\bar{M}^+ \bar{n}. \end{aligned}$$

This last expression involves the projection of the true gravitational-wave sky *a* onto the basis skies $v_{(k)}$ for only the non-zero singular values of $\Sigma$.

In Fig. 54, we show plots of the real parts of the $+$ and $\times$-polarization basis skies for a pulsar timing array consisting of $N=5$ pulsars randomly distributed on the sky. The imaginary components of the basis skies are identically zero, and hence are not shown in the figure. The basis skies are shown in decreasing size of their singular values, from top to bottom. In general, if *N* is the number of pulsars in the array, then the number of basis skies is 2*N* (the factor of 2 corresponding to the two polarizations, $+$ and $\times$). This means that one can extract at most 2*N* real pieces of information about the gravitational-wave background with an *N*-pulsar array. This is typically fewer than the number of modes of the background that we would like to recover.

#### Underdetermined reconstructions

More generally, let’s consider the case where the total number of measured data points *n* is less than the number of modes *m* that we are trying to recover (so $n<m$), or where there are certain modes of the gravitational-wave background (e.g., *null skies*) that our detector network is simply insensitive to. Then, for both of these cases, the linear system of equations that we are trying to solve, $\bar{d} = \bar{M} a$, is *underdetermined*—i.e., there exist multiple solutions for *a*, which differ from (7.75) by terms of the form

7.79 $$\begin{aligned} a_\mathrm{null} = (\mathbbm {1}_{m\times m} - \bar{M}^+\bar{M}) a_\mathrm{arb}, \end{aligned}$$

where $a_\mathrm{arb}$ is an *arbitrary* gravitational-wave background. (Note that $a_\mathrm{null}$ is an element of the *null space* of $\bar{M}$ as it maps to zero under the action of $\bar{M}$). Our solution for $\hat{a}$ given in (7.75) sets to zero those modes that we are insensitive to. Our solution also sets to zero the variance of these modes.

In a Bayesian formulation of the problem, one needs to specify prior probability distributions for the signal parameters, in addition to specifying the likelihood function (7.65). For a mode of the background to which our detector network is insensitive, the marginalized posterior for that mode will be the same as the prior, since the data are uniformative about this mode. This is what one would expect for a mode that is unconstrained by the data, in contrast to setting the variance equal to zero as we do with our maximum-likelihood reconstruction. Basically, our maximum-likelihood reconstruction does not attempt to say anything about the modes of the background for which we have no information.

**Fig. 55:**
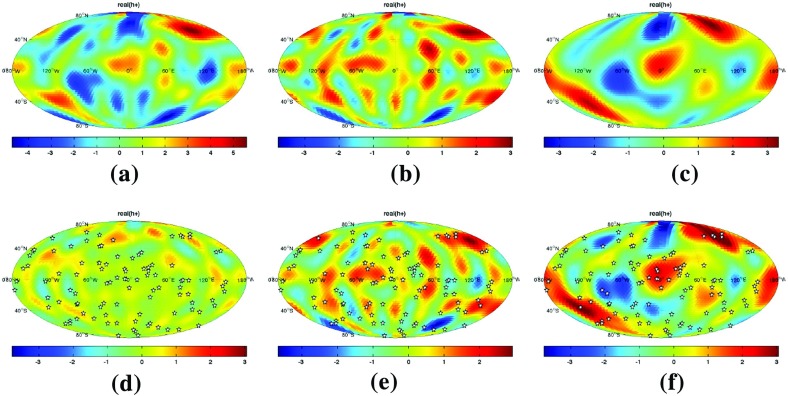
Mollweide projections of the real parts of $h_+(\hat{n})$ for the different components of the simulated background (panels **a**–**c**), the maximum-likelihood recovered map for a pulsar timing array consisting of $N=100$ pulsars (panel **e**), and the corresponding residual maps for the grad-component (panel **d**) and the total simulated background (panel **f**). Sky maps of the imaginary part of $h_+(\hat{n})$ and the real and imaginary parts of $h_\times (\hat{n})$ are similar, and hence are not shown in this figure. Note that the maximum-likelihood recovered map most-closely resembles the gradient component of the simulated background, since a pulsar timing array is insensitive to the curl modes of a gravitational-wave background. Image reproduced with permission from Gair et al. (2014), copyright by APS. **a** Total map (grad+curl). **b** Gradient component. **c** Curl component. **d** Gradient residual map. **e** Max-likelihood recovered map. **f** Total residual map

#### Pulsar timing arrays

The phase-coherent mapping approach was first developed in the context of pulsar timing arrays (Cornish and van Haasteren 2014; Gair et al. 2014). In Cornish and van Haasteren (2014), the analysis was done in terms of the standard polarization components $a\equiv \{h_+(f,\hat{n}),h_\times (f,\hat{n})\}$, similar to what we described above. In Gair et al. (2014), the analysis was done in terms of the tensor spherical harmonic components $a\equiv \{a_{(lm)}^G(f), a_{(lm)}^C(f)\}$. Now recall from (5.23) that the Earth-term-only, Doppler-frequency response functions are given by

7.80 $$\begin{aligned} R^G_{(lm)}(f) = 2\pi {}^{(2)}\!N_l Y_{lm}(\hat{p}), \quad R^C_{(lm)}(f) = 0, \end{aligned}$$

where $\hat{p}$ is the direction to an arbitrary pulsar. Thus, the pulsar response to curl modes is identically zero. This means that a pulsar timing array is blind to *half* of all possible modes of a gravitational-wave background, regardless of how many pulsars there are in the array. Note that this statement is not restricted to the tensor spherical harmonic analysis; it is also true in terms of the standard $(+,\times )$ polarization components, since $a^G_{(lm)}(f)$ and $a^C_{(lm)}(f)$ are linear combinations of $h_+(f,\hat{n})$ and $h_\times (f,\hat{n})$, see (2.11). It is just that the insensivity of a pulsar timing array to half of the gravitational-wave modes is *manifest* in the gradient and curl spherical harmonic basis for which (7.80) is valid.

To explicitly demonstrate that a pulsar timing array is insensitive to the curl-component of a gravitational-wave background, Gair et al. (2014) constructed maximum-likelihood estimates of a simulated background containing both gradient and curl modes. The total simulated background and its gradient and curl components are shown in the top row (panels a–c) of Fig. 55. (Note that this is for a noiseless simulation so as not to confuse the lack of reconstructing the curl component with the presence of detector noise). Panel e shows the maximum-likelihood recovered map for a pulsar timing array consisting of $N=100$ pulsars randomly distributed on the sky. Panels d and f are residual maps obtained by subtracting the maximum-likelihood recovered map from the gradient component and the total simulated background, respectively. Note that the maximum-likelihood recovered map resembles the gradient component of the background, consistent with the fact that a pulsar timing array is insenstive to the curl component of a gravitational-wave background. The residual map for the gradient component (panel d) is much cleaner than the residual map for the total simulated background (panel f), which has angular structure that closely resembles the curl component of the background.

#### Ground-based interferometers

The phase-coherent mapping approach can also be applied to data taken by a network of ground-based interferometers (Romano et al. 2015). Again the analysis can be performed in terms of either the standard $+$, $\times$ polarization components or the gradient and curl spherical harmonic components. Recall from (5.33) that

7.81 $$\begin{aligned} R^G_{(lm)}(f) = \delta _{l2}\frac{4\pi }{5}\sqrt{\frac{1}{3}} \left[ Y_{2m}(\hat{u}) - Y_{2m}(\hat{v})\right] , \quad R^C_{(lm)}(f) = 0, \end{aligned}$$

for a ground-based interferometer in the small-antenna limit, with its vertex at the origin, and with unit vectors $\hat{u}$, $\hat{v}$ pointing in the direction of the interferometer arms. At first, one might think that these expressions imply that a network of ground-based interferometers is also blind to the curl component of a gravitational-wave background. But (7.81) are valid only for interferometers with their vertices *at the origin* of coordinates. Since a translation mixes gradient and curl components, the response functions for an interferometer displaced from the origin by $\hat{x}_0$ are given by Romano et al. (2015):

7.82 $$\begin{aligned} \begin{aligned} R^G_{(lm)}(f) =&\sum _{m'=-2}^2 \sum _{L=l-2}^{l+2} \sum _{M=-L}^L F_{m'}(\hat{u},\hat{v})\\&\times 4\pi (-i)^L j_L(\alpha )Y^*_{LM}(\hat{x}_0) \frac{(-1)^{m'}}{2} \left[ (-1)^l+(-1)^L\right] \\&\times \sqrt{\frac{(2\cdot 2+1)(2l+1)(2L+1)}{4\pi }} \left( \begin{array}{ccc} 2 &{} l &{} L\\ -m' &{} m &{} M \end{array}\right) \left( \begin{array}{ccc} 2 &{} l &{} L\\ 2 &{} -2 &{} 0 \end{array}\right) \\ R^C_{(lm)}(f) =&\sum _{m'=-2}^2 \sum _{L=l-2}^{l+2} \sum _{M=-L}^L F_{m'}(\hat{u},\hat{v}) \\&\times 4\pi (-i)^L j_L(\alpha )Y^*_{LM}(\hat{x}_0) \frac{(-1)^{m'}}{2i} \left[ (-1)^l-(-1)^L\right] \\&\times \sqrt{\frac{(2\cdot 2+1)(2l+1)(2L+1)}{4\pi }} \left( \begin{array}{ccc} 2 &{} l &{} L\\ -m' &{} m &{} M \end{array}\right) \left( \begin{array}{ccc} 2 &{} l &{} L\\ 2 &{} -2 &{} 0 \end{array}\right) , \end{aligned} \end{aligned}$$

where $\alpha \equiv 2\pi f|{\vec {x}}_0|/c$ and $j_L(\alpha )$ are spherical Bessel functions of order *L*. Here

7.83 $$\begin{aligned} F_m(\hat{u}, \hat{v}) \equiv \frac{4\pi }{5}\sqrt{\frac{1}{3}} \left[ Y_{2m}(\hat{u}) - Y_{2m}(\hat{v})\right] , \end{aligned}$$

is shorthand for the particular combination of spherical harmonics that enter the expression for $R^G_{(lm)}(f)$ in (7.81). The two expressions in parentheses $(\ )$ for each response function are Wigner 3-*j* symbols (see, e.g., Wigner 1959; Messiah 1962). Note that the curl response is now non-zero, and both response functions depend on frequency via the quantity $\alpha$, which equals $2\pi$ times the number of radiation wavelengths between the origin and the vertex of the interferometer. These expressions are valid in an arbitrary translated and rotated coordinate system, provided the angles for $\hat{u}$, $\hat{v}$, and $\hat{x}_0$ are calculated in the rotated frame.

**Fig. 56:**
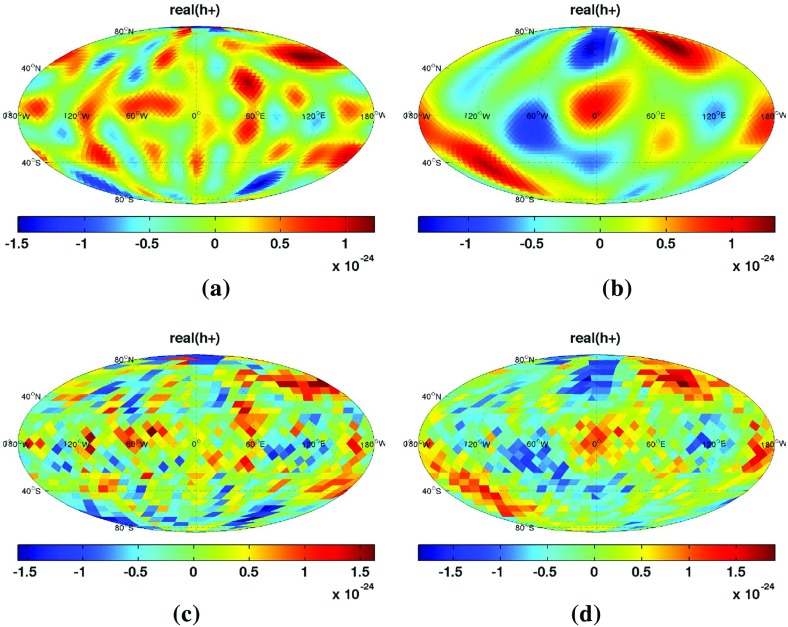
Mollweide projections of the real parts of $h_+(\hat{n})$ for grad-only and curl-only anisotropic backgrounds injected into noise and analysed using a 3-detector network of ground-based laser interferometers (Hanford–Livingston–Virgo). The injected maps are shown in the *top row*; the maximum-likelihood recovered maps are shown in the *second row*. Sky maps of the imaginary part of $h_+(\hat{n})$ and the real and imaginary parts of $h_\times (\hat{n})$ are similar for both the injections and the recovered maps, and hence are not shown in the figure. Note that a network of ground-based interferometers is capable of recovering both the gradient and curl components of a gravitational-wave background, in contrast to a pulsar timing array (compare with Fig. 55). Image reproduced with permission from Romano et al. (2015), copyright by APS. **a** Injected grad-only map. **b** Injected curl-only map. **c** Max-likelihood recovered map. **d** Max-likelihood recovered map

Thus, the spatial separation of a network of ground-based interferometers, or of a single interferometer at different times during its daily rotational and yearly orbital motion around the Sun (Sect. 5.5.3), allows for recovery of both the gradient *and* curl components of a gravitational-wave background. This is in contrast to a pulsar timing array, which is insensitive to the curl component, because one vertex of all the pulsar baselines are ‘pinned’ to the solar system barycenter. To illustrate this difference, we show in Fig. 56, maximum-likelihood recovered sky maps for simulated grad-only and curl-only anistropic backgrounds injected into noise for a 3-detector network of ground-based interferometers (Hanford–Livingston–Virgo). The grad-only and curl-only backgrounds are the same as those used for the simulated maps in Fig. 55. In contrast to the recovered maps shown in that figure for the pulsar timing array, the maximum-likelihood maps (bottom row) for the network of ground-based interferometers reproduce the general angular structure of both the grad-only *and* curl-only injected maps (shown in the top row). (The noise for these injections degrades the recovery compared to the noiseless injections in Fig. 55). See Romano et al. (2015) for more details and related simulations.

## Searches for other types of backgrounds/signals

No idea is so outlandish that it should not be considered with a searching but at the same time a steady eye. *Winston Churchill*

Since stochastic gravitational-wave backgrounds come in many different “flavors”, one needs additional search methods that go beyond the standard “vanilla” cross-correlation search for a Gaussian-stationary, unpolarized, isotropic signal (Sects. 4, 5) to extract the relevant information from the more exotic backgrounds. In Sect. 7, we discussed how to search for *anisotropic* signals, which are stronger coming from certain directions on the sky than from others. In this section, we discuss search methods for non-Gaussian signals (Sect. 8.1), circularly polarized backgrounds (Sect. 8.2), and additional polarization modes predicted by alternative (non-general-relativity) metric theories of gravity (Sects. 8.3, 8.4, 8.5). In Sect. 8.6, we also briefly mention searches for other types of gravitational-wave signals, which are not really stochastic backgrounds, but nonetheless can be searched for using the basic idea of cross-correlation, which we developed in Sect. 4. The majority of the search methods that we will describe here have been implemented “across the band”—i.e., for ground-based interferometers, space-based interferometers, and pulsar timing arrays. For these methods, we will highlight any significant differences in the implementations for the different detectors, if there are any.

**Fig. 57:**
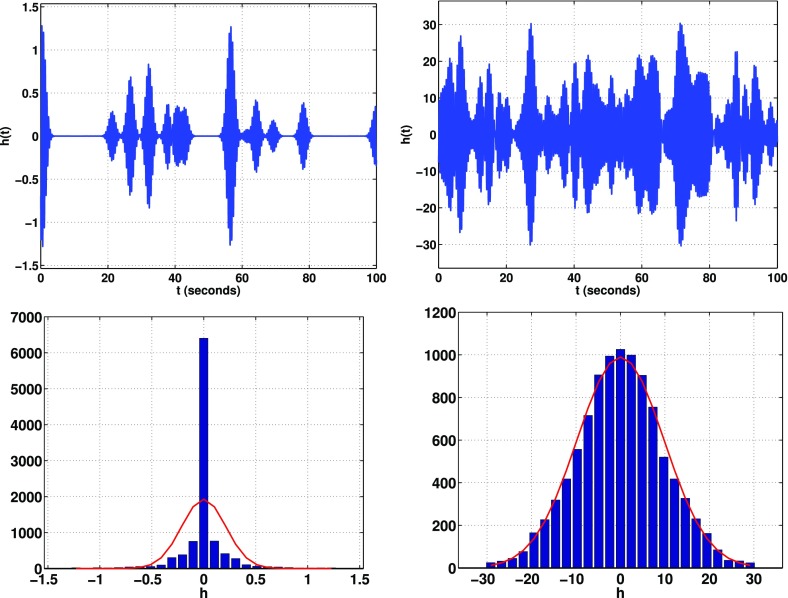
Simulated toy-model signals and histograms for different duty cycles. The *left two panels* correspond to 1 burst every 10 s (on average); the *right two panels* correspond to 100 bursts every second (on average). The *red curves* in the *bottom two panels* show the best-fit Gaussian distributions to the data. Similar to Fig. 1 from Thrane (2013)

Of course, we do not have enough time or space in this section to do justice for all of these methods. As such, readers are strongly encouraged to read the original papers for more details. For non-Gaussian backgrounds, see Drasco and Flanagan (2003), Seto (2009), Thrane (2013), Martellini and Regimbau (2014) and Cornish and Romano (2015); for circular polarization, see Seto and Taruya (2007, 2008) and Kato and Soda (2016); for polarization modes in alternative theories of gravity, see Lee et al. (2008), Nishizawa et al. (2009), Chamberlin and Siemens (2012) and Gair et al. (2015); and for the other types of signals, see Thrane et al. (2011) and Messenger et al. (2015).

### Non-Gaussian backgrounds

In Sect. 2.1, we asked the question “when is a gravitational-wave signal stochastic” to highlight the practical distinction between searches for deterministic and stochastic signals. From an operational perspective, a signal is stochastic if it is best searched for using a stochastic signal model (i.e., one defined in terms of probability distributions), even if the signal is *intrinsically* deterministic, e.g., a superposition of sinusoids. This turns out to be the case if the signals are: (i) *sufficiently weak* that they are individually unresolvable in a single detector, and hence can only be detected by integrating their correlated contribution across multiple detectors over an extended period of time, or (ii) they are *sufficiently numerous* that they overlap in time-frequency space, again making them individually unresolvable, but producing a *confusion noise* that can be detected by cross-correlation methods. If the rate of signals is large enough, the confusion noise will be Gaussian thanks to the central limit theorem. But if the rate or duty-cycle is small, then the resulting stochastic signal will be non-Gaussian and “popcorn-like”, as we discussed in Sect. 1.1. This is the type of signal that we expect from the population of binary black holes that produced GW150914 and GW151226; and it is the type of signal that we will focus on in the following few subsections.

Figure 57 illustrates the above statements in the context of a simple toy-model signal consisting of simulated sine-Gaussian bursts (each with a width $\sigma _t= 1~\mathrm{s}$) having different rates or duty cycles. The left two panels correspond to the case where there is 1 burst every 10 seconds (on average). The probability distribution of the signal samples *h* (estimated by the histogram in the lower-left-hand panel) is far from Gaussian for this case. The right two panels correspond to 100 bursts every second (on average), for which the probability distribution is approximately Gaussian-distributed, as expected from the central limit theorem.

#### Non-Gaussian search methods: overview

There are basically two different approaches that one can take to search for non-Gaussian stochastic signals: (i) The first is to incorporate the non-Gaussianity of the signal into the likelihood function by marginalizing over the appropriate signal model (Sect. 8.1.2). Then given the likelihood, one can construct frequentist detection statistics and estimators from the maximum-likelihood ratio (3.24), or do Bayesian model selection in the usual way (Sect. 3). (ii) The second approach is to construct specific frequentist statistics that targets the higher-order moments of the non-Gaussian distribution, and then use these statistics to do standard frequentist hypothesis testing and parameter estimation. This approach is most simply cast in terms of the *skewness* and (excess) *kurtosis* of the distribution, which are the third and fourth-order *cumulants*, defined as follows: If *X* is a random variable with probability distribution $p_X(x)$, then the *moments* are defined by (Appendix B):

8.1 $$\begin{aligned} \mu _n \equiv \langle X^n\rangle = \int dx\> x^n p_X(x), \end{aligned}$$

and the *cumulants* by

8.2 $$\begin{aligned} \begin{aligned} c_1&= \mu _1, \\ c_2&= \mu _2-\mu _1^2, \\ c_3&= \mu _3-3\mu _2\mu _1 + 2\mu _1^3, \\ c_4&= \mu _4-4\mu _3\mu _1 - 3\mu _2^2 + 12\mu _2\mu _1^2-6\mu _1^4, \\&\vdots \end{aligned} \end{aligned}$$

Note that $c_1$ and $c_2$ are just the *mean* $\mu$ and *variance* $\sigma ^2$ of the distribution. For a Gaussian distribution, $c_3=0, c_4=0, \ldots$. For a distribution with zero mean, the above formulas simplify to $c_1=0$, $c_2=\mu _2$, $c_3=\mu _3$, and $c_4 = \mu _4 - 3\mu _2^2$. The higher-order-moment approach requires 3rd or 4th-order correlation measurements (Sect. 8.1.5).

#### Likelihood function approach for non-Gaussian backgrounds

Fundamentally, searching for non-Gaussian stochastic signals is no different than searching for a Gaussian stochastic signal. In both cases one must: (i) specify a signal model, (ii) incorporate that signal model into a likelihood function or frequentist detection statistic/estimator, and (iii) then analyze the data to determine how likely it is that a signal is present. It is the choice of signal model, of course, that determines what type of signal is being searched for.

The signal model is incorporated into the likelihood via marginalization over the signal samples as discussed in Sect. 3.6.2. Assuming Gaussian-stationary noise^22^ with covariance matrix $C_n$, the probability of observing data *d* in a network of detectors given signal model $\bar{h}$ is (3.53):

8.3 $$\begin{aligned} p(d|\bar{h}, C_n) = \frac{1}{\sqrt{\det (2\pi C_n)}} e^{-\frac{1}{2} \sum _{Ii,Jj}r_{Ii} \left( C_n^{-1}\right) _{Ii,Jj} r_{Jj}}, \end{aligned}$$

where

8.4 $$\begin{aligned} r_{Ii}\equiv d_{Ii} - \bar{h}_{Ii} \end{aligned}$$

are the residuals in detector *I*. (The subscript *i* labels either a time or frequency sample for the analysis, whichever is being used). Since one is often not interested in the particular values of $\bar{h}$, but rather the values of the parameters $\mathbf {\theta }_h$ that describe the signal, one marginalizes over $\bar{h}$:

8.5 $$\begin{aligned} p(d|\mathbf {\theta }_h,\mathbf {\theta }_n) = \int \! d\bar{h}\> p(d|\bar{h}, C_n) p(\bar{h}|\mathbf {\theta }_h). \end{aligned}$$

This yields a likelihood function that depends on the signal and noise parameters $\mathbf {\theta }_h$, $\mathbf {\theta }_n\equiv C_n$. It is this likelihood function that we then use for our statistical analysis.

Several different signal priors, which have been proposed in the literature, are given below. For simplicity, we will consider the case where the detectors are colocated and coaligned, and have isotropic antenna patterns, so that the contribution from the signal is the same in each detector, and is independent of direction on the sky. For real analyses, these simplifications will need to be dropped, as is done e.g., in Thrane (2013).

*Gaussian signal prior*

8.6 $$\begin{aligned} p(\bar{h}| S_h)= \frac{1}{(2\pi S_h)^{N/2}}\, e^{-\frac{1}{2 S_h}\sum _{i=1}^N \bar{h}_i^2}. \end{aligned}$$

This is the standard prior that one uses for describing a Gaussian-stochastic signal, and leads to the usual Gaussian-stochastic cross-correlation detection statistic (Sect. 4.4).

Drasco and Flanagan (2003) *non-Gaussian signal prior*

8.7 $$\begin{aligned} p(\bar{h}| \xi , \alpha )= \prod _{i=1}^N\left[ \xi \,\frac{1}{\sqrt{2\pi \alpha ^2}}\, e^{-\bar{h}_i^2/2\alpha ^2} +(1-\xi )\,\delta (\bar{h}_i) \right] . \end{aligned}$$

This prior corresponds to Gaussian bursts occuring with probability $0\le \xi \le 1$ and with root-mean-square (rms) amplitude $\alpha$.

*Mixture-Gaussian signal prior*

8.8 $$\begin{aligned} p(\bar{h}| \xi , \alpha , \beta )= \prod _{i=1}^N\left[ \xi \,\frac{1}{\sqrt{2\pi \alpha ^2}}\, e^{-\bar{h}_i^2/2\alpha ^2} +(1-\xi )\,\frac{1}{\sqrt{2\pi \beta ^2}}\, e^{-\bar{h}_i^2/2\beta ^2} \right] . \end{aligned}$$

The mixture-Gaussian signal prior is a non-Gaussian distribution, which reduces to the Gaussian signal prior in the limit $\xi \rightarrow 1$. It reduces to the Drasco and Flanagan signal prior in the limit $\beta \rightarrow 0$.

Martellini and Regimbau (2014) *non-Gaussian signal prior*

8.9 $$\begin{aligned} p(\bar{h}| \xi , \alpha )= \prod _{i=1}^N\left[ \xi \,p_\mathrm{NG}(\bar{h}_i) +(1-\xi )\,\delta (\bar{h}_i) \right] , \end{aligned}$$

where

8.10 $$\begin{aligned}&p_\mathrm{NG}(\bar{h}_i) \nonumber \\&\quad = \frac{1}{\sqrt{2\pi \alpha ^2}}\, e^{-\bar{h}_i^2/2\alpha ^2} \left[ 1 +\frac{c_3}{6\alpha ^3}H_3\left( \frac{\bar{h}_i}{\alpha }\right) +\frac{c_4}{24\alpha ^4}H_4\left( \frac{\bar{h}_i}{\alpha }\right) +\frac{c_3^2}{72\alpha ^6}H_6\left( \frac{\bar{h}_i}{\alpha }\right) \right] \nonumber \\ \end{aligned}$$

is the 4th-order Edgeworth expansion (Martellini and Regimbau 2014) of a non-Gaussian distribution with third and fourth-order cumulants $c_3$ and $c_4$. ($H_n(x)$ denotes a Hermite polynomial of order *n*). The Edgeworth expansion is referenced off a Gaussian probability distribution, and is thus said to be a *semi-parametric* representation of a non-Gaussian distribution. This prior reduces to the Drasco and Flanagan signal prior when $c_3=0$, $c_4=0$.

*Multi-sinusoid signal prior*

8.11 $$\begin{aligned} \begin{aligned}&p(\bar{h}|\mathbf {\theta }_h) = \delta \left( \bar{h} - \bar{h}(\mathbf {\theta }_h)\right) , \\&\bar{h}_i(\mathbf {\theta }_h) = \sum _{I=1}^M A_I \cos (2\pi f_I t_i - \varphi _I). \end{aligned} \end{aligned}$$

This is a *deterministic* signal prior, corresponding to the superposition of *M* sinusoids with unknown amplitudes, frequencies, and phases, $\mathbf {\theta }_h = \{A_I, f_I, \varphi _I\vert I=1,2,\ldots , M\}$. This was one of the signal models used in Cornish and Romano (2015) to investigate the question of when is a signal stochastic.

*Superposition of finite-duration deterministic signals*

8.12 $$\begin{aligned} \begin{aligned}&p(\bar{h}|\mathbf {\theta }_h) = \delta \left( \bar{h} - \bar{h}(\mathbf {\theta }_h)\right) , \\&\bar{h}_i(\mathbf {\theta }_h) = \sum _{I=1}^M A_I \mathcal{T}(t_i - t_I|\mathbf {\theta }_\mathcal{T}). \end{aligned} \end{aligned}$$

Here, $\mathcal{T}(t|\mathbf {\theta }_\mathcal{T})$ is a normalized waveform (template) for some deterministic signal (e.g., a chirp from an inspiralling binary, a sine-Gaussian burst, a ringdown signal, $\ldots$) described by parameters $\mathbf {\theta }_\mathcal{T}$ (e.g., chirp mass, correlation time, frequency, $\ldots$). $A_I$ is the amplitude of the *I*th signal and $t_I$ is its arrival time. Note that these signal waveforms can be *extended* in time, having a characteristic duration $\tau$. Thus, this signal model is intermediate between the single-sample burst and multi-sinusoid signal models.

*Generic likelihood for unresolvable signals*

In Thrane (2013), Thrane writes down a generic likelihood function for a non-Gaussian background formed from the superposition of signals which are individually unresolvable in a single detector. The likelihood function:

8.13 $$\begin{aligned} p(\hat{\rho }|\xi ,\mathbf {\theta }_h, \mathbf {\theta }_n) =\prod _i\left[ \xi \,S(\hat{\rho }_i|\mathbf {\theta }_h) +(1-\xi )\,B(\hat{\rho }_i|\mathbf {\theta }_n) \right] \end{aligned}$$

is defined for a pair of detectors *I*, *J*, and takes as its fundamental data vector estimates of the signal-to-noise ratio of the cross-correlated power in the two detectors:

8.14 $$\begin{aligned} \hat{\rho }_i \equiv \hat{\rho }(t;f) = \sqrt{\tau \delta f} \frac{\hat{C}_{IJ}(t;f)}{\sqrt{P_{n_I}(t;f) P_{n_J}(t;f)}}, \end{aligned}$$

where

8.15 $$\begin{aligned} \hat{C}_{IJ}(t;f) \equiv \frac{2}{\tau }\tilde{d}_I(t;f) \tilde{d}_J^*(t;f). \end{aligned}$$

Here $\tau$ is the duration of the short-term Fourier transforms and $\delta f$ is the frequency resolution. (Note that $\delta f$ can be greater than $1/\tau$ if one averages together neighboring frequency bins). The product over *i* is over time-frequency pixels *tf*. The functions *S* and *B* are probability distributions for $\hat{\rho }_i$ for the signal and noise models, respectively. These distributions are generic in the sense that they are to be estimated using Monte Carlo simulations with injected signals for the signal model *S*, and via time-slides on real data for the noise model *B*. They need not be Gaussian for either the signal or the detector noise. The vectors $\mathbf {\theta }_h$ and $\mathbf {\theta }_n$ denote parameters specific to the signal and noise models. Although the above likelihood function was not obtained by explicitly marginalizing over $\bar{h}$, mathematically there is some signal prior and noise model which yields this likelihood upon marginalization.

#### Frequentist detection statistic for non-Gaussian backgrounds

As discussed in Sect. 3.4, given likelihood functions for the signal-plus-noise and noise-only models, we can construct a frequentist detection statistic from either the maximum-likelihood ratio $\Lambda _\mathrm{ML}(d)$ given by (3.24), or twice its logarithm, $\Lambda (d) \equiv 2\ln (\Lambda _\mathrm{ML}(d))$, which has the interpretation of being the squared signal-to-noise ratio of the relevant data. For a white Gaussian stochastic signal in white Gaussian detector noise (assuming a pair of colocated and coaligned detectors), we showed in Sect. 4.4:

8.16 $$\begin{aligned} \Lambda ^\mathrm{G}_\mathrm{ML}(d) =\left[ 1-\frac{\hat{S}_h^2}{\hat{S}_1\hat{S}_2}\right] ^{-N/2}, \quad \Lambda ^\mathrm{G}(d) \approx \frac{\hat{S}_h^2}{\hat{S}_{n_1}\hat{S}_{n_2}/N}, \end{aligned}$$

where *N* is the number of samples, and where the last approximate equality assumes that the gravitational-wave signal is weak compared to the detector noise. We have added the superscript G to indicate that this is for a Gaussian-stochastic signal model.

We can perform exactly the same calculations, making the same assumptions, for the likelihood functions constructed from *any* of the non-Gaussian signal priors given above (in Sect. 8.1.2). These calculations have already been done for the Drasco–Flanagan and Martellini–Regimbau signal priors (Drasco and Flanagan 2003; Martellini and Regimbau 2014). The expressions that they find for the maximum-likelihood ratios $\Lambda _\mathrm{ML}^\mathrm{NG}(d)$ for their non-Gaussian signal models are rather long and not particularly informative, so we do not bother to write them down here (interested readers should see (1.8) in Drasco and Flanagan 2003, and the last equation in Martellini and Regimbau 2014). The values of the parameters that maximize the likelihood ratio are estimators of $\xi$, $\alpha$, $S_{n_1}$, $S_{n_2}$ for the Drasco and Flanagan signal model, and estimators of $\xi$, $\alpha$, $c_3$, $c_4$, $S_{n_1}$, $S_{n_2}$ for the Martellini and Regimbau signal model.

**Fig. 58:**
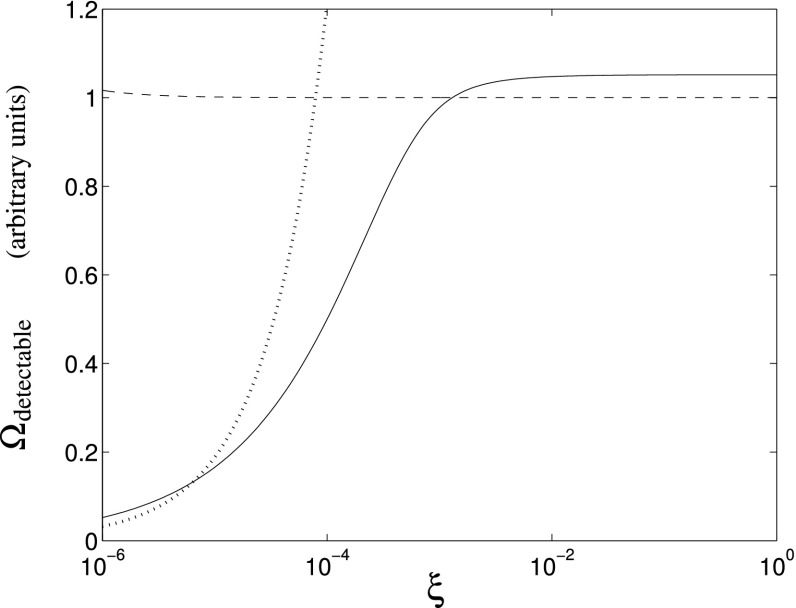
The minimum detectable value of $\Omega _\mathrm{gw}$ as a function of the duty cycle $\xi$. The *solid line* is the theoretical prediction for the Drasco and Flanagan non-Gaussian maximum-likelihood statistic; the *dashed line* is for the standard Gaussian-stochastic cross-correlation statistic; and the *dotted line* is for a single-detector burst statistic. The number of data points used was $N=10^9$, and the false alarm and false dismissal probabilities were both chosen to equal 0.01. Image reproduced with permission from Drasco and Flanagan (2003), copyright by APS

To illustrate the performance of a non-Gaussian detection statistic, we plot in Fig. 58 the minimum value of $\Omega _\mathrm{gw}$ ($S_h$ in the notation above) necessary for detection as a function of the duty cycle $\xi$. (The signal becomes Gaussian as $\xi \rightarrow 1$). The solid line is the theoretical prediction for the Drasco and Flanagan non-Gaussian maximum-likelihood statistic, while the dashed line is the theoretical prediction for the standard Gaussian-stochastic cross-correlation statistic. The dotted line is the theoretical prediction for a single-detector *burst* statistic, which is just the maximum of the absolute value of the data samples in e.g., detector 1: $\Lambda ^\mathrm{B}(d) = \max _{i} |d_{1i}|$. The false alarm and false dismissal probabilities were both chosen to equal 0.01 for this calculation. From the figure one sees that for $\xi \,{\gtrsim }10^{-3}$, the Gaussian-stochastic cross-correlation statistic performs best. For smaller values of $\xi$, the non-Gaussian statistic is better. In particular, for $\xi \sim 10^{-4}$. there is a factor of ${\sim }2$ improvement in the minimum detectable signal amplitude if one uses the non-Gaussian maximum-likelihood detection statistic.

**Fig. 59:**
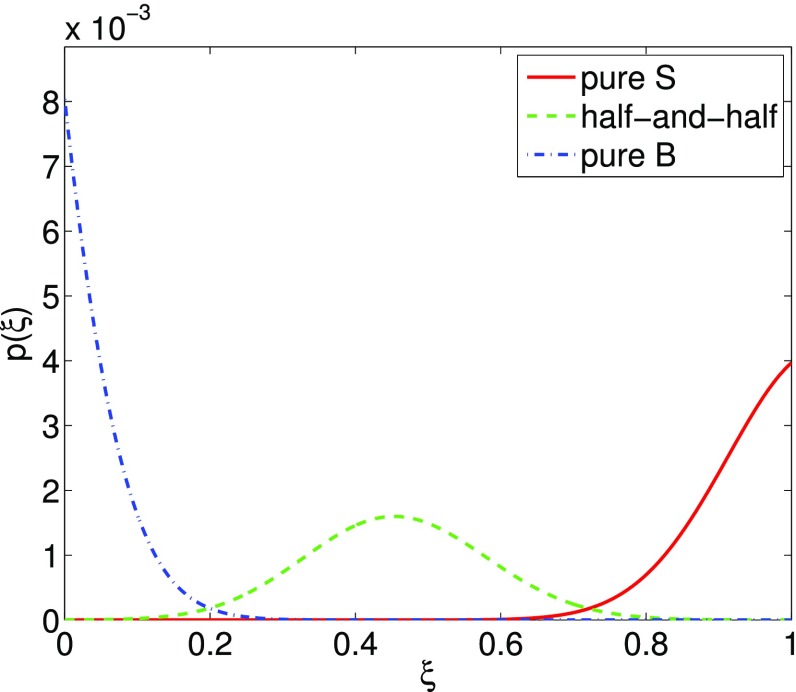
Posterior distributions for the duty cycle $\xi$ calculated for Monte Carlo simulations having $\xi =0$ (*dash-dot blue*), $\xi =1$ (*solid red*), and $\xi =0.5$ (*dashed green*). Image reproduced with permission from Thrane (2013), copyright by APS

Figure 59 is taken from Thrane (2013) and shows posterior distributions for the duty cycle $\xi$ calculated for Monte Carlo simulations corresponding to pure background $\xi =0$ (dash-dot blue), pure signal $\xi =1$ (solid red), and an even mixture $\xi =0.5$ (dashed green). These curves illustrate that the formalism in Thrane (2013) can provide estimates of the duty cycle $\xi$ of the non-Gaussian background. See Thrane (2013) for more details.

#### Bayesian model selection

As an alternative to using frequentist detection statistics and estimators to search for potentially non-Gaussian signals, one can use Bayesian model selection to compare the noise-only model $\mathcal{M}_0$ to different signal-plus-noise models $\mathcal{M}_1, \mathcal{M}_2, \ldots$. This is a general procedure for Bayesian inference, which was discussed in Sect. 3.3.3. As shown there, the posterior odds ratio between two different models $\mathcal{M}_\alpha$ and $\mathcal{M}_\beta$ can be written as

8.17 $$\begin{aligned} \mathcal{O}_{\alpha \beta }(d) = \frac{p(\mathcal{M}_\alpha \vert d )}{p(\mathcal{M}_\beta \vert d )} = \frac{p(\mathcal{M}_\alpha )}{p(\mathcal{M}_\beta )}\, \frac{ p(d \vert \mathcal{M}_\alpha )}{p(d \vert \mathcal{M}_\beta )}, \end{aligned}$$

where the first ratio on the right-hand side is the *prior* odds for the two models, while the second term is the *Bayes factor*:

8.18 $$\begin{aligned} \mathcal{B}_{\alpha \beta }(d) = \frac{p(d \vert \mathcal{M}_\alpha )}{p(d \vert \mathcal{M}_\beta )}, \end{aligned}$$

which is a ratio of model evidences:

8.19 $$\begin{aligned} p(d \vert \mathcal{M}_\alpha ) = \int p( d\vert \mathbf {\theta }_\alpha , \mathcal{M}_\alpha ) p(\mathbf {\theta }_\alpha \vert \mathcal{M}_\alpha ) \, d\mathbf {\theta }_\alpha , \end{aligned}$$

and similarly for $p(d|\mathcal{M}_\beta )$. If one assumes equal prior odds, then the posterior odds ratio is just the Bayes factor, and we can use its value to rule in favor of one model or another (see Table 3).

The idea of using Bayesian model selection in the context of searches for non-Gaussian stochastic backgrounds was proposed by us in Cornish and Romano (2015). We considered a simple toy-problem consisting of simulated data in two colocated and coaligned detectors, having uncorrelated white Gaussian detector noise plus a gravitational-wave signal formed from the superposition of sinusoids having amplitudes drawn from an astrophysical population of sources. Such a signal is effectively the *frequency-domain version* of the short-duration time-domain bursts discussed in the previous subsections. Five different models were considered:

- $\mathcal{M}_0$: noise-only model, consisting of uncorrelated white Gaussian noise in two detectors with unknown variances $\sigma _1^2$, $\sigma _2^2$.
- $\mathcal{M}_1$: noise plus the Gaussian-stochastic signal model defined by (8.6).
- $\mathcal{M}_2$: noise plus the mixture-Gaussian stochastic signal model defined by (8.8).
- $\mathcal{M}_3$: noise plus the deterministic multisinusoid model defined by (8.11).
- $\mathcal{M}_4$: noise plus the deterministic multisinusoid signal model plus the Gaussian-stochastic signal model. This is a *hybrid* signal model that allows for both stochastic and deterministic components for the signal.

Simulated data were generated by coadding sinusoidal signals with amplitudes drawn from an astrophysical model (Sesana 2013), and phases and frequencies drawn uniformly across the range spanned by the data. Gaussian-distributed white noise for the two detectors were then added to the signal data. The amplitude of the signals were scaled so as to produce a specified matched filter signal-to-noise ratio per frequency bin. Markov Chain Monte Carlo analyses were run to compare the noise-only model $\mathcal{M}_0$ to each of the four signal-plus-noise models $\mathcal{M}_1,\ldots , \mathcal{M}_4$. Quantile intervals for the Bayes factors were estimated from 256 independent realizations of the simulated data for each set of parameter values. These intervals capture the fluctuation in the Bayes factors that come from *different* realizations of the data; they are not uncertainties in the Bayes factors associated with different Monte Carlo simulations for a *single* realization, which were ${\lesssim }10\%$.

**Fig. 60:**
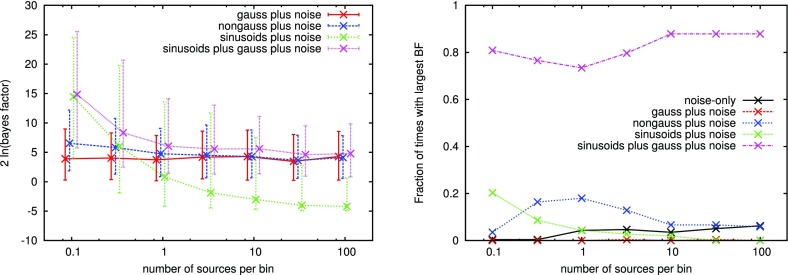
*Left panel* Bayes factor 80% quantile intervals for the four different signal-plus-noise models relative to the noise-only model as a function of the number of sources per bin. *Right panel* Fraction of time that the different models had the largest Bayes factor for the different simulations. Image reproduced with permission from Cornish and Romano (2015), copyright by APS

Figure 60 is a representative plot taken from Cornish and Romano (2015), comparing the different models. The left panel shows the Bayes factors for the four different signal-plus-noise models relative to the noise-only model plotted as a function of the average number of sources per bin. The right panel shows the fraction of time that the different models had the largest Bayes factor for the different simulations. The total number of bins was set to 32 for these simulations and the SNR per bin was fixed at 2. From these and other similar plots in Cornish and Romano (2015), one can draw the general conclusion that deterministic models are generally favored for small source densities, a non-Gaussian stochastic model is preferred for intermediate source densities, and a Gaussian-stochastic model is preferred for large source densities. Given the large fluctuations in the Bayes factors associated with different signal realizations, the boundaries between these three regimes is rather fuzzy. The hybrid model, which has a deterministic component for the bright signals and a Gaussian-stochastic component for the remaining confusion background, is the best model for the majority of cases.

#### Fourth-order correlation approach for non-Gaussian backgrounds

In this section, we briefly describe a fourth-order correlation approach for detecting non-Gaussian stochastic signals, originally proposed in Seto (2009). The key idea is that by forming a particular combination of data from 4 detectors (the *excess kurtosis*), one can separate the non-Gaussian contribution to the background from any Gaussian-distributed component. This approach requires that the noise in the four detectors be uncorrelated with one another, but it does not require that the noise be Gaussian. Here we sketch out the calculation for 4 colocated and coaligned detectors, which we will assume have isotropic antenna patterns, so that the contribution from the gravitational-wave signal is the same in each detector, and is independent of direction on the sky. These simplifying assumptions are not essential for this approach; the calculation for separated and misalinged detectors with non-isotropic response functions can also be done (Seto 2009).

Let’s begin then by denoting the output of the four detectors $I=1,2,3,4$ in the Fourier domain by

8.20 $$\begin{aligned} \tilde{d}_I = \tilde{n}_I + \tilde{h}, \quad \tilde{h} = \tilde{g} + \sum _{i=1}^n \tilde{b}_i, \end{aligned}$$

where $\tilde{n}_I$ denotes the noise in detector *I* and $\tilde{h}$ denotes the total gravitational-wave contribution, which has a Gaussian-stochastic component $\tilde{g}$, and a non-Gaussian component formed from the superposition of short-duration burst signals $\tilde{b}_i$, $i=1,2,\ldots , n$. We assume that the noise in the detectors are uncorrelated with one another and with the gravitational-wave signals, and that the individual gravitational-wave signals are also uncorrelated amongst themselves. The (random) number of bursts present in a particular segment of data is determined by a Poisson distribution

8.21 $$\begin{aligned} P(n) = \frac{\lambda ^n e^{-\lambda }}{n!}, \end{aligned}$$

where

8.22 $$\begin{aligned} \lambda = \langle n\rangle = \sum _{n=0}^\infty nP(n), \end{aligned}$$

is the expected number of bursts in segment duration $T_\mathrm{seg}$. The 4th-order combination of data that we consider is

8.23 $$\begin{aligned} \mathcal{K} \equiv \langle \tilde{d}_1 \tilde{d}_2 \tilde{d}_3^* \tilde{d}_4^*\rangle -\langle \tilde{d}_1 \tilde{d}_2\rangle \langle \tilde{d}_3^* \tilde{d}_4^*\rangle -\langle \tilde{d}_1 \tilde{d}_3^*\rangle \langle \tilde{d}_2 \tilde{d}_4^*\rangle -\langle \tilde{d}_1 \tilde{d}_4^*\rangle \langle \tilde{d}_2 \tilde{d}_3^*\rangle , \end{aligned}$$

where angle brackets $\langle \ \rangle$ can be thought of as either expectation value (i.e., ensemble average) or as an average over the Fourier components of the data, i.e., as an *estimator* of the expected correlations. Since the noise in the detectors are uncorrelated with everything, the only contributions to $\mathcal{K}$ will come from expectation values of products of $\tilde{h} = \tilde{g} +\sum _i \tilde{b}_i$ with itself. Calculating the quadratic terms that enter (8.23), we find:

8.24 $$\begin{aligned} \begin{aligned} \langle \tilde{d}_I\tilde{d}_J\rangle&= \langle \tilde{g}\tilde{g}\rangle + \lambda \langle \tilde{b}\tilde{b}\rangle , \\ \langle \tilde{d}_I\tilde{d}_J^*\rangle&= \langle \tilde{g}\tilde{g}^*\rangle + \lambda \langle \tilde{b}\tilde{b}^*\rangle , \end{aligned} \end{aligned}$$

where we used

8.25 $$\begin{aligned} \left\langle \sum _i\sum _j \tilde{b}_i \tilde{b}_j\right\rangle =\left\langle \sum _i \tilde{b}_i \tilde{b}_i\right\rangle = \lambda \langle \tilde{b}\tilde{b}\rangle , \end{aligned}$$

which assumes that all the bursts have the same mean-square value, $\langle \tilde{b}_i\tilde{b}_i\rangle \equiv \langle \tilde{b}\tilde{b}\rangle$. For the 4th-order term, we find:

8.26 $$\begin{aligned} \langle \tilde{d}_1\tilde{d}_2\tilde{d}_3^*\tilde{d}_4^*\rangle= & {} \langle \tilde{g}\tilde{g}\tilde{g}^*\tilde{g}^*\rangle +\,\lambda ^2\left[ |\langle \tilde{b}\tilde{b}\rangle |^2 +2\langle \tilde{b}\tilde{b}^*\rangle ^2 \right] \nonumber \\&+\,\lambda \left[ \langle \tilde{b}\tilde{b}\tilde{b}^*\tilde{b}^*\rangle +\langle \tilde{g}\tilde{g}\rangle \langle \tilde{b}\tilde{b}\rangle ^* +\langle \tilde{g}\tilde{g}\rangle ^*\langle \tilde{b}\tilde{b}\rangle +4\langle \tilde{g}\tilde{g}^*\rangle \langle \tilde{b}\tilde{b}^*\rangle \right] \!.\nonumber \\ \end{aligned}$$

Substituting these results back into expression (8.23) yields:

8.27 $$\begin{aligned} \mathcal{K} = \lambda \langle \tilde{b} \tilde{b}\tilde{b}^*\tilde{b}^*\rangle , \end{aligned}$$

where we used

8.28 $$\begin{aligned} \langle \tilde{g}\tilde{g}\tilde{g}^*\tilde{g}^*\rangle -|\langle \tilde{g}\tilde{g}\rangle |^2 -2\langle \tilde{g}\tilde{g}^*\rangle ^2 =0, \end{aligned}$$

for the Gaussian-stochastic signal component $\tilde{g}$. Thus, both the detector noise and the Gaussian-stochastic component of the signal have dropped out of the expression for $\mathcal{K}$, leaving only the contribution from the non-Gaussian component of the background.

As mentioned already, the above calculation can be extended to the case of separated and misaligned detectors (Seto 2009). In so doing, one obtains expressions for *generalized* (4th-order) overlap functions, which are sky-averages of the product of the response functions for four different detectors. The expected value of the 4th-order detection statistic for this more general analysis involves generalized overlap functions for both the (squared) overall intensity and circular polarization components of the non-Gaussian background. We will discuss circular polarization in the following section, but in the simpler context of Gaussian-stationary isotropic backgrounds. Readers should see Seto (2009) for more details regarding circular polarization in the context of non-Gaussian stochastic signals discussed above.

### Circular polarization

Up until now, we have only considered *unpolarized* stochastic backgrounds. That is, we have assumed that the gravitational-wave power in the $+$ and $\times$ polarization modes are equal (on average) and are statistically independent of one another (i.e., there are no correlations between the $+$ and $\times$ polarization modes). It is possible, however, for some processes in the early Universe to give rise to *parity violations* (Alexander et al. 2006), which would manifest themselves as an asymmetry in the amount of right and left *circularly* polarized gravitational waves. Following Seto and Taruya (2007, 2008), we now describe how to generalize our cross-correlation methods to look for evidence of circular polarization in a stochastic background.

#### Polarization correlation matrix

Let us start by writing down the quadratic expectation values for the Fourier components $h_{ab}(f,\hat{n})$ of the metric perturbations $h_{ab}(t,{\vec {x}})$ for a *polarized anisotropic* Gaussian-stationary background. (We will restrict attention to isotropic backgrounds later on). It turns out that these expectation values can be written in terms of the *Stokes’ parameters* *I*, *Q*, *U*, and *V*, which are defined for a monochromatic plane gravitational wave in Appendix A. If we expand $h_{ab}(f,\hat{n})$ in terms of the *linear* polarization basis tensors $e^A_{ab}(\hat{n})$, where $A=\{+,\times \}$, we have

8.29 $$\begin{aligned} \langle h_A(f,\hat{n}) h_{A'}^*(f',\hat{n}')\rangle =\frac{1}{2} S_h^{AA'}(f,\hat{n}) \delta (f-f') \delta ^2(\hat{n},\hat{n}'), \end{aligned}$$

where

8.30 $$\begin{aligned} S_h^{AA'}(f,\hat{n}) =\frac{1}{2}\left[ \begin{array}{cc} I(f,\hat{n}) + Q(f,\hat{n}) &{} \quad U(f,\hat{h})-iV(f,\hat{n}) \\ U(f,\hat{n})+iV(f,\hat{n}) &{} \quad I(f,\hat{n}) - Q(f,\hat{n}) \\ \end{array} \right] . \end{aligned}$$

If instead we expand $h_{ab}(f,\hat{n})$ in terms of the *circular* polarization basis tensors $e^C_{ab}(\hat{n})$, where $C=\{R,L\}$, then

8.31 $$\begin{aligned} \langle h_C(f,\hat{n}) h_{C'}^*(f',\hat{n}')\rangle =\frac{1}{2} S_h^{CC'}(f,\hat{n}) \delta (f-f') \delta ^2(\hat{n},\hat{n}'), \end{aligned}$$

where

8.32 $$\begin{aligned} S_h^{CC'}(f, \hat{n}) =\frac{1}{2}\left[ \begin{array}{cc} I(f,\hat{n}) + V(f,\hat{n}) &{} \quad Q(f,\hat{h})-iU(f,\hat{n}) \\ Q(f,\hat{n})+iU(f,\hat{n}) &{} \quad I(f,\hat{n}) - V(f,\hat{n}) \\ \end{array} \right] . \end{aligned}$$

This second representation of the polarization correlation matrix is sometimes more convenient when one is searching for evidence of circular polarization in the background, as *V* is a measure of a possible asymmetry between the right and left circular polarization components:

8.33 $$\begin{aligned} \langle h_R(f,\hat{n}) h_{R'}^*(f',\hat{n}')\rangle -\langle h_L(f,\hat{n}) h_{L'}^*(f',\hat{n}')\rangle =\frac{1}{2} V(f,\hat{n}) \delta (f-f') \delta ^2(\hat{n},\hat{n}'). \end{aligned}$$

The factor of 1/2 on the right-hand side of the above equation, as compared to (A.16), is for one-sided power spectra.

As discussed in Appendix A, the Stokes’ parameters *I* and *V* are ordinary scalar (spin 0) fields on the sphere, while *Q* and *U* transform like spin 4 fields under a rotation of the unit vectors $\{\hat{l},\hat{m}\}$ tangent to the sphere. Thus, *I* and *V* can be written as linear combinations of the ordinary spherical harmonics $Y_{lm}(\hat{n})$:

8.34 $$\begin{aligned} \begin{aligned} I(f,\hat{n})&= \sum _{l=0}^\infty \sum _{m=-l}^l I_{lm}(f) Y_{lm}(\hat{n}), \\ V(f,\hat{n})&= \sum _{l=0}^\infty \sum _{m=-l}^l V_{lm}(f) Y_{lm}(\hat{n}), \\ \end{aligned} \end{aligned}$$

while $Q\pm iU$ can be written as linear combination of the spin-weighted $\pm 4$ spherical harmonics ${}_{\pm 4}Y_{lm}(\hat{n})$:

8.35 $$\begin{aligned} Q(f,\hat{n}) \pm i U(f,\hat{n}) = \sum _{l=4}^\infty \sum _{m=-l}^l C^{\pm }_{lm}(f)\, {}_{\pm 4}Y_{lm}(\hat{n}). \end{aligned}$$

Note that the expansions for $Q\pm i U$ start at $l=4$, which means that the *Q*, *U* components of the polarization correlation matrix vanish if the background is isotropic (i.e., has only a contribution from the monopole $l=0$, $m=0$). So for simplicity, we will restrict our attention to polarized *isotropic backgrounds*, for which the circular polarization correlation matrix becomes diagonal and the quadratic exprectation values reduce to:

8.36 $$\begin{aligned} \langle h_C(f,\hat{n}) h_{C'}^*(f',\hat{n}')\rangle =\frac{1}{8\pi } S_h^{C}(f) \delta _{CC'} \delta (f-f') \delta ^2(\hat{n},\hat{n}'), \end{aligned}$$

where

8.37 $$\begin{aligned} \begin{aligned} S_h^{R}(f)&\equiv \frac{1}{2}(I(f) + V(f)), \\ S_h^{L}(f)&\equiv \frac{1}{2}(I(f) - V(f)). \end{aligned} \end{aligned}$$

Note that

8.38 $$\begin{aligned} S_h^R(f) + S_h^L(f) = I(f) \equiv S_h(f), \end{aligned}$$

which is just the total strain power spectral density for the gravitational-wave background.

#### Overlap functions

Given (8.36), we are now in a position to calculate the expected value of the product of the Fourier transforms of the response of two detectors *I* and *J* to such a background. Similar to (5.9), we can write the response of detector *I* as

8.39 $$\begin{aligned} \tilde{h}_I(f) = \int d^2\Omega _{\hat{n}}\> \left( R^R(f,\hat{n}) h_R(f,\hat{n})+R^L(f,\hat{n}) h_L(f,\hat{n})\right) , \end{aligned}$$

where *R*, *L* label the right and left circular polarization states for both the Fourier components and the detector response functions. Writing down a similar expression for the response of detector *J*, and using (8.36) to evaluate the expected value of the product of the responses, we find

8.40 $$\begin{aligned} \langle \tilde{h}_I(f)\tilde{h}_J^*(f')\rangle = \frac{1}{2}\delta (f-f') \left[ \Gamma ^{(I)}_{IJ}(f)I(f) + \Gamma ^{(V)}_{IJ}(f)V(f)\right] , \end{aligned}$$

where

8.41 $$\begin{aligned} \begin{aligned} \Gamma ^{(I)}_{IJ}(f)&\equiv \frac{1}{8\pi }\int d^2\Omega _{\hat{n}}\> \left[ R^R_I(f,\hat{n}) R^{R*}_J(f,\hat{n}) + R^L_I(f,\hat{n}) R^{L*}_J(f,\hat{n}) \right] , \\ \Gamma ^{(V)}_{IJ}(f)&\equiv \frac{1}{8\pi }\int d^2\Omega _{\hat{n}}\> \left[ R^R_I(f,\hat{n}) R^{R*}_J(f,\hat{n}) - R^L_I(f,\hat{n}) R^{L*}_J(f,\hat{n}) \right] , \end{aligned} \end{aligned}$$

are the overlap functions for the *I* and *V* Stokes parameters for a polarized isotropic stochastic background. Using

8.42 $$\begin{aligned} \begin{aligned} R^R&= \frac{1}{\sqrt{2}}\left( R^++iR^\times \right) , \\ R^L&= \frac{1}{\sqrt{2}}\left( R^+-iR^\times \right) , \end{aligned} \end{aligned}$$

we can also write the above overlap functions as

8.43 $$\begin{aligned} \begin{aligned} \Gamma ^{(I)}_{IJ}(f)&\equiv \frac{1}{8\pi }\int d^2\Omega _{\hat{n}}\> \left[ R^+_I(f,\hat{n}) R^{+*}_J(f,\hat{n}) + R^\times _I(f,\hat{n}) R^{\times *}_J(f,\hat{n}) \right] , \\ \Gamma ^{(V)}_{IJ}(f)&\equiv \frac{i}{8\pi }\int d^2\Omega _{\hat{n}}\> \left[ R^+_I(f,\hat{n}) R^{+*}_J(f,\hat{n}) - R^\times _I(f,\hat{n}) R^{\times *}_J(f,\hat{n}) \right] . \end{aligned} \end{aligned}$$

Note that $\Gamma ^{(I)}_{IJ}(f)$ is identical to the ordinary overlap function $\Gamma _{IJ}(f)$ for an isotropic background (5.36).

**Fig. 61:**
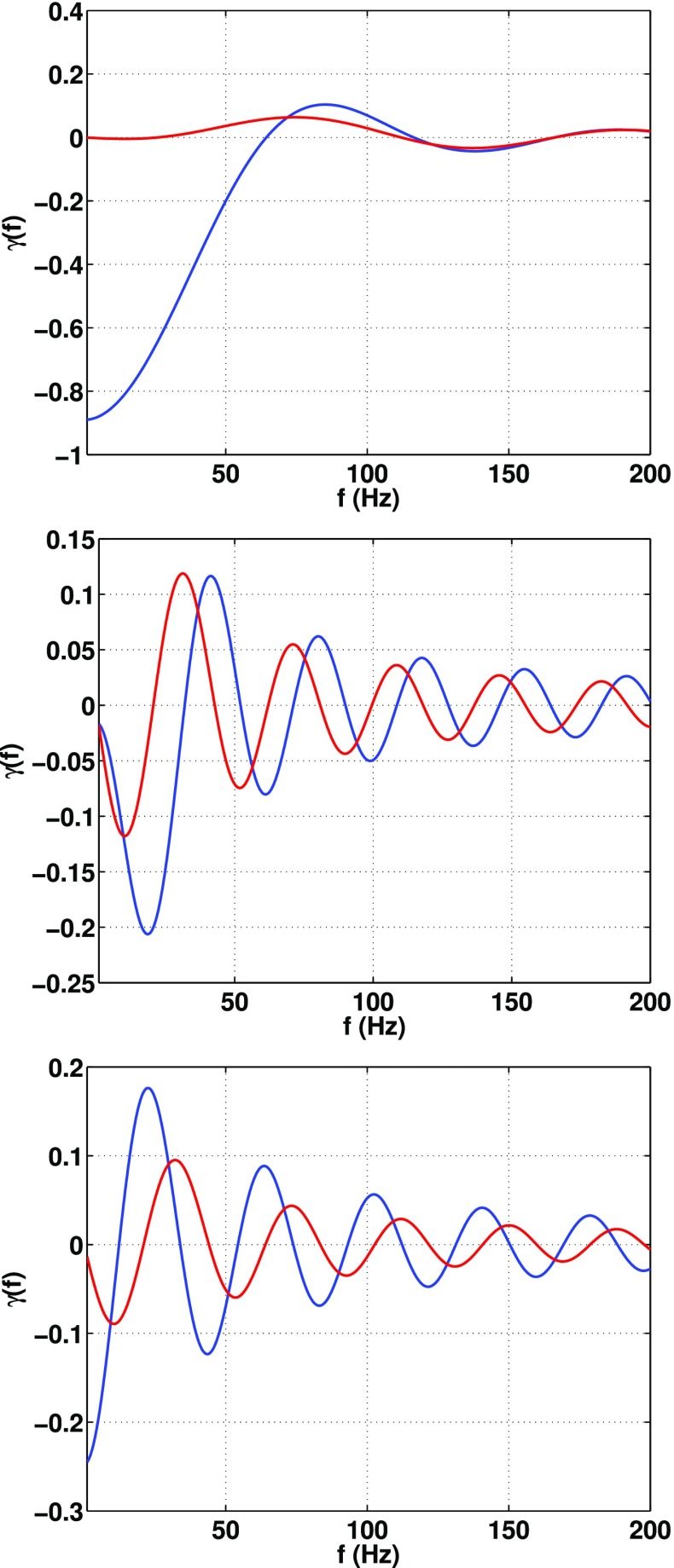
Normalized overlap functions for the *I* and *V* Stokes’ parameters for the LIGO Hanford-LIGO Livingston detector pair (*top panel*); for the LIGO Hanford-Virgo detector pair (*middle panel*); for the LIGO Livingston-Virgo detector pair (*bottom panel*). The *I* overlap functions are shown in *blue*; the *V* overlap functions are shown in *red*. Note the change in scale of the vertical axes

Figure 61 show plots of the *I* and *V* overlap functions for the LIGO-Virgo detector pairs, using the small-antenna limit for the strain response functions. The overlap functions have been normalized (5.40) so that $\gamma ^{(I)}_{IJ}(f)=1$ for colocated and coaligned detectors. Similar plots can be made for other interferometer pairs, by simply using the appropriate response functions for those detectors.

Note that for pulsar timing, one can show that $\Gamma ^{(V)}_{IJ}(f) = 0$ for any pair of pulsars. This means that one cannot detect the presence of a circularly polarized stochastic background using a pulsar timing array if one restricts attention to just the isotropic component of the background. One must include higher-order multipoles in the analysis—i.e., do an *anisotropic* search as discussed in Sect. 7. Such an analysis for anisotropic polarized backgrounds using pulsar timing arrays is given in Kato and Soda (2016). In that paper, they extend the analysis of Mingarelli et al. (2013) to include circular polarization. See Kato and Soda (2016) for additional details.

#### Component separation: ML estimates of *I* and *V*

As shown in Seto and Taruya (2007, 2008), in order to separate the *I*(*f*) and *V*(*f*) contributions to a polarized isotropic background at each frequency *f*, we will need to analyze data from at least two independent baselines (so three or more detectors). In what follows, we will use the notation $\alpha =1,2,\ldots , N_b$ to denote the individual baselines (detector pairs) and $\alpha _1$, $\alpha _2$ to denote the two detectors that constitute that baseline. The formalism we adopt here is similar to that for constructing maximum-likelihood estimators of gravitational-wave power for unpolarized anisotropic backgrounds (Sect. 7.3). For a general discussion of component separation for isotropic backgrounds, see Parida et al. (2016).

As usual, we begin by cross-correlating the data from pairs of detectors for the independent baselines:

8.44 $$\begin{aligned} \hat{C}_\alpha (f) \equiv \frac{2}{T}\, \tilde{d}_{\alpha _1}(f) \tilde{d}^*_{\alpha _2}(f), \end{aligned}$$

where

8.45 $$\begin{aligned} \tilde{d}_{\alpha _I}(f) = \tilde{h}_{\alpha _I}(\tilde{f}) + \tilde{n}_{\alpha _I}(f), \quad I=1,2, \end{aligned}$$

are the Fourier transforms of the time-domain data $d_{\alpha _I}(t)$, and *T* is the duration of the data. Assuming that the noise in the individual detectors are uncorrelated with one another, we can easily calculate the expected value of $\hat{C}_\alpha (f)$ using our previous result (8.40). The result is

8.46 $$\begin{aligned} \langle \hat{C}_\alpha (f) \rangle =\Gamma ^{(I)}_\alpha (f)I(f)+ \Gamma ^{(V)}_\alpha (f)V(f). \end{aligned}$$

We will write this equation abstractly as a matrix equation

8.47 $$\begin{aligned} \langle \hat{C}\rangle = M \mathcal{S}, \end{aligned}$$

where

8.48 $$\begin{aligned} \hat{C} = \left[ \begin{array}{c} \hat{C}_1 \\ \hat{C}_2 \\ \vdots \\ \hat{C}_{N_b} \\ \end{array} \right] , \quad M \equiv \left[ \begin{array}{cc} \Gamma _1^{(I)} &{} \Gamma _1^{(V)} \\ \Gamma _2^{(I)} &{} \Gamma _2^{(V)} \\ \vdots &{} \vdots \\ \Gamma _{N_b}^{(I)} &{} \Gamma _{N_b}^{(V)} \end{array} \right] , \quad \mathcal{S}\equiv \left[ \begin{array}{c} I \\ V \\ \end{array} \right] . \end{aligned}$$

In this notation, $\hat{C}$ is an $N_f N_b\times 1$ data vector, *M* is an $N_f N_b\times 2 N_f$ detector network response matrix, and $\mathcal{S}$ is an $2N_f\times 1$ vector containing the unknown Stokes’ parameters, which we want to estimate from the data.^23^

We also need an expression for the noise covariance matrix $\mathcal{N}$ for the cross-correlated data $\hat{C}$. In the weak-signal limit, the covariance matrix is approximately diagonal with matrix elements

8.49 $$\begin{aligned} \begin{aligned} \mathcal{N}_{\alpha \alpha '}(f,f')&\equiv \langle \hat{C}_\alpha (f) \hat{C}^*_{\alpha '}(f')\rangle -\langle \hat{C}_\alpha (f)\rangle \langle \hat{C}^*_{\alpha '}(f')\rangle \\&\approx \delta _{\alpha \alpha '} \delta _{ff'} P_{n_{\alpha _1}}(f) P_{n_{\alpha _2}}(f), \end{aligned} \end{aligned}$$

where $P_{n_{\alpha _I}}(f)$ are the one-sided power spectral densities of the noise in the detectors. If we treat the noise power spectra as known quantities (or if we estimate the noise power spectra from the auto-correlated output of each detector), we can write down a likelihood function for the cross-correlated data given the signal model (8.47). Assuming a Gaussian-stationary distribution for the noise, we have

8.50 $$\begin{aligned} p(\hat{C}|\mathcal{S}) \propto \exp \left[ -\frac{1}{2}(\hat{C} - M\mathcal{S})^\dagger \mathcal{N}^{-1} (\hat{C} - M\mathcal{S})\right] . \end{aligned}$$

This likelihood has exactly the same form as that in (7.32), so the maximum-likehood estimators for the Stokes’ parameters $\mathcal{S} = [I, V]^T$ also have the same form:

8.51 $$\begin{aligned} \hat{\mathcal{S}} =F^{-1} X, \end{aligned}$$

where

8.52 $$\begin{aligned} F\equiv M^\dagger \mathcal{N}^{-1} M, \quad X\equiv M^\dagger \mathcal{N}^{-1} \hat{C}, \end{aligned}$$

with *M* and $\mathcal{N}$ given above. As before, inverting *F* may require some sort of regularization, e.g., using singular-value decomposition (Sect. 7.3.5). If that’s the case then $F^{-1}$ should be replaced in the above formula by its pseudo-inverse $F^+$. The uncertainty in the maximum likelihood recovered values is given by the covariance matrix

8.53 $$\begin{aligned} \langle \hat{\mathcal{S}}\hat{\mathcal{S}}^\dagger \rangle - \langle \hat{\mathcal{S}}\rangle \langle \hat{\mathcal{S}}^\dagger \rangle \approx F^{-1}, \end{aligned}$$

where we are again assuming the weak-signal limit.

#### Example: component separation for two baselines

As an explicit example, we now write down the maximum-likelihood estimators for the Stokes’ parameters $\mathcal{S} = [I, V]^T$ for a detector network consisting of two baselines $\alpha$ and $\beta$. For this case, the detector network response matrix *M* is a square $2 N_f\times 2 N_f$ matrix, which we assume has non-zero determinant. Then it follows simply from the definitions (8.52) of *F* and *X* that

8.54 $$\begin{aligned} \hat{\mathcal{S}} = F^{-1} X = M^{-1}\hat{C}, \end{aligned}$$

for which

8.55 $$\begin{aligned} \begin{aligned} \hat{I}(f)&= \left( \Gamma ^{(I)}_\alpha \Gamma ^{(V)}_\beta - \Gamma ^{(I)}_\beta \Gamma ^{(V)}_\alpha \right) ^{-1} \left[ \Gamma ^{(V)}_\beta \hat{C}_\alpha - \Gamma ^{(V)}_\alpha \hat{C}_\beta \right] , \\ \hat{V}(f)&= \left( \Gamma ^{(I)}_\alpha \Gamma ^{(V)}_\beta - \Gamma ^{(I)}_\beta \Gamma ^{(V)}_\alpha \right) ^{-1} \left[ -\Gamma ^{(I)}_\beta \hat{C}_\alpha + \Gamma ^{(I)}_\alpha \hat{C}_\beta \right] . \end{aligned} \end{aligned}$$

The marginalized uncertainties in these estimates are obtained by taking the diagonal elements of the inverse of the Fisher matrix:

8.56 $$\begin{aligned} \begin{aligned} \sigma _{\hat{I}}^2&= (F^{-1})_{II} = \frac{N_\alpha \,(\Gamma ^{(V)}_\beta )^2+ N_\beta \,(\Gamma ^{(V)}_\alpha )^2}{\left( \Gamma ^{(I)}_\alpha \Gamma ^{(V)}_\beta - \Gamma ^{(I)}_\beta \Gamma ^{(V)}_\alpha \right) ^2}, \\ \sigma _{\hat{V}}^2&= (F^{-1})_{VV} = \frac{N_\alpha \,(\Gamma ^{(I)}_\beta )^2+ N_\beta \,(\Gamma ^{(I)}_\alpha )^2}{\left( \Gamma ^{(I)}_\alpha \Gamma ^{(V)}_\beta - \Gamma ^{(I)}_\beta \Gamma ^{(V)}_\alpha \right) ^2}, \end{aligned} \end{aligned}$$

where $N_\alpha$, $N_\beta$, defined by $N_\alpha (f)\equiv P_{n_{\alpha _1}}(f) P_{n_{\alpha _2}}(f)$ (and similarly for $N_\beta$), is a diagonal element of the noise covariance matrix $\mathcal{N}$ (8.49).

#### Effective overlap functions for *I* and *V* for multiple baselines

The above expressions for the uncertainties in the estimates of *I* and *V* can easily be extended to the case of an arbitrary number of baselines $\alpha = 1,2,\ldots , N_b$. For multiple baselines with noise spectra $N_\alpha (f)\equiv P_{n_{\alpha _1}}(f) P_{n_{\alpha _2}}(f)$, one can show that

8.57 $$\begin{aligned} F = \left[ \begin{array}{cc} \sum _\alpha N_\alpha ^{-1}(\Gamma ^{(I)}_\alpha )^2 &{}\quad \sum _\alpha N_\alpha ^{-1}\Gamma ^{(I)}_\alpha \Gamma ^{(V)}_\alpha \\ \sum _\alpha N_\alpha ^{-1}\Gamma ^{(V)}_\alpha \Gamma ^{(I)}_\alpha &{}\quad \sum _\alpha N_\alpha ^{-1}(\Gamma ^{(V)}_\alpha )^2 \\ \end{array} \right] . \end{aligned}$$

Let us assume that the determinant of the $2\times 2$ matrices for each frequency (which we will denote by $\bar{F}$) are not equal to zero. Then

8.58 $$\begin{aligned} \begin{aligned} \sigma _{\hat{I}}^2&= (\bar{F}^{-1})_{II} = \frac{1}{\det (\bar{F})}\, \sum _\alpha N_\alpha ^{-1}(\Gamma ^{(V)}_\alpha )^2, \\ \sigma _{\hat{V}}^2&= (\bar{F}^{-1})_{VV} = \frac{1}{\det (\bar{F})}\, \sum _\alpha N_\alpha ^{-1}(\Gamma ^{(I)}_\alpha )^2. \end{aligned} \end{aligned}$$

Following Seto and Taruya (2008), we can now define *effective* overlap functions for *I* and *V* associated with a multibaseline detector network by basically inverting the above uncertainties. For simplicity, we will assume that the noise power spectra for the detectors are equal to one another so that $N_\alpha \equiv N$ can be factored out of the above expressions. We then define

8.59 $$\begin{aligned} \begin{aligned} \Gamma _\mathrm{eff}^{(I)}(f) \equiv \sqrt{N}\sigma _{\hat{I}}^{-1}&=\left( \frac{N^2\det (\bar{F})}{\sum _\alpha (\Gamma ^{(V)}_\alpha )^2}\right) ^{1/2}, \\ \Gamma _\mathrm{eff}^{(V)}(f) \equiv \sqrt{N}\sigma _{\hat{V}}^{-1}&=\left( \frac{N^2\det (\bar{F})}{\sum _\alpha (\Gamma ^{(I)}_\alpha )^2}\right) ^{1/2}. \end{aligned} \end{aligned}$$

**Fig. 62:**
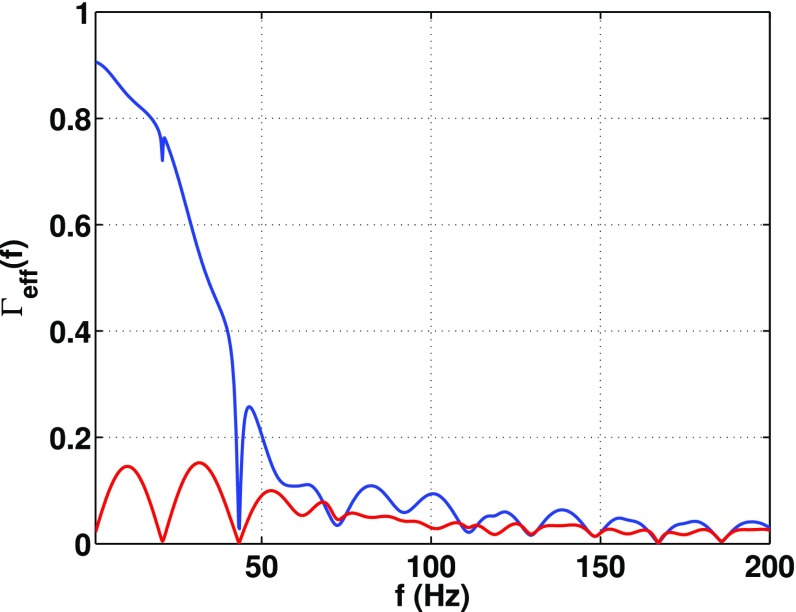
Effective overlap functions for *I* and *V* for the multibaseline network formed from the LIGO Hanford, LIGO Livingston, and Virgo detectors. $\Gamma _\mathrm{eff}^{(I)}(f)$ is shown in *blue*; $\Gamma _\mathrm{eff}^{(V)}(f)$ is shown in *red*

These quantities give us an indication of how sensitive the multibaseline network is to extracting the *I* and *V* components of the background. Plots of $\Gamma _\mathrm{eff}^{(I)}(f)$ and $\Gamma _\mathrm{eff}^{(V)}(f)$ are shown in Fig. 62 for the multibaseline network formed from the LIGO Hanford, LIGO Livingston, and Virgo detectors. Recall that the overlap functions for the individual detectors pairs are shown in Fig. 61. Dips in sensitivity correspond to frequencies where the determinant of $\bar{F}$ is zero (or close to zero).

### Non-GR polarization modes: preliminaries

**Fig. 63:**
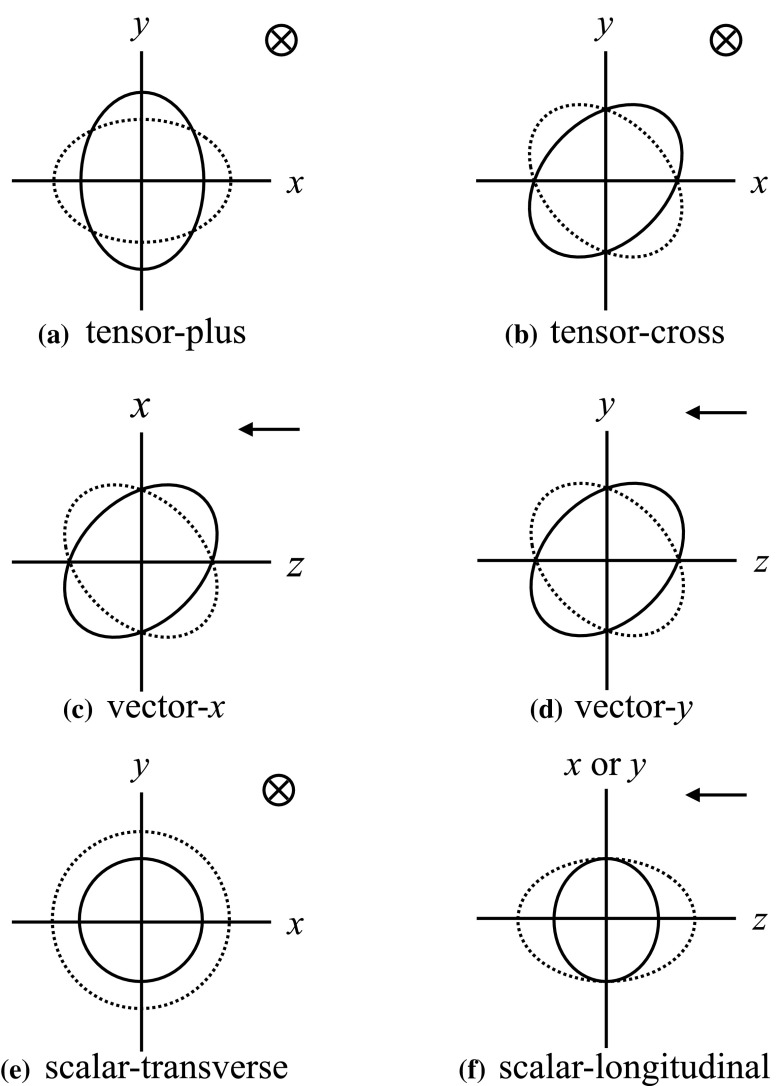
Graphical representation of the six different polarization modes. The *circle with a cross or arrow* represents the direction of propagation of the gravitational wave. The *solid and dotted circles and ellipses* denote deformations to a ring of particles $180^\circ$ out of phase with one another. Adapted from Fig. 1 in Nishizawa et al. (2009).

In a general metric theory of gravity, there are six possible polarization modes: The standard $+$ and $\times$ *tensor* modes predicted by general relativity (GR); two *vector* (or “shear”) modes, which we will denote by *X* and *Y*; and two *scalar* modes: a “breathing” mode *B* and a pure longitudinal mode *L* (see, e.g., Nishizawa et al. 2009). The tensor and breathing modes are *transverse* to the direction of propagation, while the two vector modes and the scalar longitudinal mode have *longitudinal* components (parallel to the direction of propagation). See Fig. 63.

In terms of the orthonormal vectors $\{\hat{n},\hat{l},\hat{m}\}$ defined by (2.4), the polarization basis tensors for the six different polarization modes are:

8.60 $$\begin{aligned} \begin{array}{ll} e_{ab}^{+}(\hat{n}) =\hat{l}_a\hat{l}_b-\hat{m}_a\hat{m}_b, &{}\quad e_{ab}^{\times }(\hat{n}) =\hat{l}_a\hat{m}_b + \hat{m}_a\hat{l}_b. \\ e_{ab}^{X}(\hat{n}) =\hat{l}_a\hat{n}_b+\hat{n}_a\hat{l}_b, &{}\quad e_{ab}^{Y}(\hat{n}) =\hat{m}_a\hat{n}_b + \hat{n}_a\hat{m}_b, \\ e_{ab}^{B}(\hat{n}) =\hat{l}_a\hat{l}_b+\hat{m}_a\hat{m}_b, &{}\quad e_{ab}^{L}(\hat{n}) =\sqrt{2}\,\hat{n}_a\hat{n}_b. \end{array} \end{aligned}$$

We will denote these collectively as $e^A_{ab}(\hat{n})$, where $A=\{+,\times ,X,Y,B,L\}$. In a coordinate system where $\hat{n}$ points along the *z*-axis, and $\hat{l}$ and $\hat{m}$ point along the *x* and *y* axes, the polarization tensors can be represented by the following $3\times 3$ matrices:

8.61 $$\begin{aligned}&e_{ab}^+= \left[ \begin{array}{c@{\quad }c@{\quad }c} 1 &{} 0 &{} 0\\ 0 &{} -1 &{} 0\\ 0 &{} 0 &{} 0\\ \end{array} \right] , \quad e_{ab}^\times = \left[ \begin{array}{c@{\quad }c@{\quad }c} 0 &{} 1 &{} 0\\ 1 &{} 0 &{} 0\\ 0 &{} 0 &{} 0\\ \end{array} \right] , \nonumber \\&e_{ab}^X= \left[ \begin{array}{c@{\quad }c@{\quad }c} 0 &{} 0 &{} 1\\ 0 &{} 0 &{} 0\\ 1 &{} 0 &{} 0\\ \end{array} \right] , \quad e_{ab}^Y= \left[ \begin{array}{ccc} 0 &{} 0 &{} 0\\ 0 &{} 0 &{} 1\\ 0 &{} 1 &{} 0\\ \end{array} \right] , \nonumber \\&e_{ab}^B= \left[ \begin{array}{c@{\quad }c@{\quad }c} 1 &{} 0 &{} 0\\ 0 &{} 1 &{} 0\\ 0 &{} 0 &{} 0\\ \end{array} \right] , \quad e_{ab}^L= \left[ \begin{array}{ccc} 0 &{} 0 &{} 0\\ 0 &{} 0 &{} 0\\ 0 &{} 0 &{} \sqrt{2}\\ \end{array} \right] . \end{aligned}$$

#### Transformation of the polarization tensors under a rotation about $\hat{n}$

We have already seen (Appendix A) that under a rotation of the unit vectors $\{\hat{l}, \hat{m}\}$ by an angle $\psi$ around $\hat{n}$, the polarization tensors $e^+_{ab}(\hat{n})$, $e^\times _{ab}(\hat{n})$ transform to:

8.62 $$\begin{aligned} \begin{aligned} \epsilon ^+_{ab}(\hat{n},\psi )&=\cos 2\psi \, e^+_{ab}(\hat{n})+\sin 2\psi \, e^\times _{ab}(\hat{n}), \\ \epsilon ^\times _{ab}(\hat{n},\psi )&=-\sin 2\psi \, e^+_{ab}(\hat{n})+\cos 2\psi \, e^\times _{ab}(\hat{n}). \end{aligned} \end{aligned}$$

This reflects the spin 2 nature of the tensor modes $+$, $\times$ in general relativity. Similarly, under the same rotation, the polarization tensors $e^X_{ab}(\hat{n})$, $e^Y_{ab}(\hat{n})$ transform to:

8.63 $$\begin{aligned} \begin{aligned} \epsilon ^X_{ab}(\hat{n},\psi )&=\cos \psi \, e^X_{ab}(\hat{n})+\sin \psi \, e^Y_{ab}(\hat{n}), \\ \epsilon ^Y_{ab}(\hat{n},\psi )&=-\sin \psi \, e^X_{ab}(\hat{n})+\cos \psi \, e^Y_{ab}(\hat{n}), \end{aligned} \end{aligned}$$

while $e^B_{ab}(\hat{n})$, $e^L_{ab}(\hat{n})$ are left unchanged:

8.64 $$\begin{aligned} \begin{aligned} \epsilon ^B_{ab}(\hat{n},\psi )&= e^B_{ab}(\hat{n}), \\ \epsilon ^L_{ab}(\hat{n},\psi )&= e^L_{ab}(\hat{n}). \end{aligned} \end{aligned}$$

These last two transformations correspond to the spin 1 nature of the vector modes *X*, *Y*, and the spin 0 nature of the scalar modes *B*, *L*.

#### Polarization and spherical harmonic basis expansions

For the tensor modes $+$, $\times$, we found (Sect. 2.2.2) that it was convenient to expand the Fourier components $h_{ab}(f,\hat{k})$ of the metric perturbations $h_{ab}(t,{\vec {x}})$ in terms of either the polarization basis tensors:

8.65 $$\begin{aligned} h_{ab}(f,\hat{n}) = h_+(f,\hat{n}) e^+_{ab}(\hat{n}) + h_\times (f,\hat{n}) e^\times _{ab}(\hat{n}), \end{aligned}$$

or the gradient and curl tensor spherical harmonics:

8.66 $$\begin{aligned} h_{ab}(f,\hat{n}) =\sum _{l=2}^\infty \sum _{m=-l}^l \left[ a^G_{(lm)}(f)Y^G_{(lm)ab}(\hat{n}) +a^C_{(lm)}(f)Y^C_{(lm)ab}(\hat{n})\right] . \end{aligned}$$

Recall that $Y^G$ and $Y^C$ are related to spin-weight $\pm 2$ spherical harmonics as described in Appendices G and E. For the vector and scalar modes we can perform similar expansions, provided we use appropriately defined tensor spherical harmonics, which transform properly under rotations. For the vector modes *X*, *Y*, we need to use the *vector-gradient* and *vector-curl* spherical harmonics $Y^{V_G}$, $Y^{V_C}$, which are defined in terms of spin-weight $\pm 1$ spherical harmonics (Appendices F and E). For the scalar modes, we can use

8.67 $$\begin{aligned} \begin{aligned} Y^B_{(lm)ab}(\hat{n})\equiv \frac{1}{\sqrt{2}} Y_{lm}(\hat{n}) e^B_{ab}(\hat{n}), \quad Y^L_{(lm)ab}(\hat{n})\equiv \frac{1}{\sqrt{2}} Y_{lm}(\hat{n}) e^L_{ab}(\hat{n}), \end{aligned} \end{aligned}$$

which are defined in terms of ordinary (scalar) spherical harmonics. In terms of these definitions, we can write the expansions in compact form

8.68 $$\begin{aligned} h_{ab}(f,\hat{n}) = \sum _A h_A(f,\hat{n}) e^A_{ab}(\hat{n}), \end{aligned}$$

or

8.69 $$\begin{aligned} h_{ab}(f,\hat{n}) = \sum _P \sum _{(lm)} a^P_{(lm)}(f)Y^P_{(lm)ab}(\hat{n}), \end{aligned}$$

where $A=\{+,\times , X, Y, B, L\}$ and $P=\{G,C, V_G, V_C, B, L\}$ or some subsets thereof. Note that $\sum _{(lm)}$ is shorthand for

8.70 $$\begin{aligned} \sum _{l=2}^\infty \sum _{m=-l}^l, \quad \sum _{l=1}^\infty \sum _{m=-l}^l, \quad \sum _{l=0}^\infty \sum _{m=-l}^l, \end{aligned}$$

for the tensor, vector, and scalar modes, respectively.

#### Detector response

The detector response functions corresponding to the above two expansions (8.68) and (8.69) are:

8.71 $$\begin{aligned} R^A(f,\hat{n}) = R^{ab}(f,\hat{n}) e^A_{ab}(\hat{n}), \end{aligned}$$

and

8.72 $$\begin{aligned} R^P_{(lm)}(f) =\int d^2\Omega _{\hat{n}}\> R^{ab}(f, \hat{n}) Y^P_{(lm)ab}(\hat{n}). \end{aligned}$$

In terms of these response functions, the detector response (in the frequency domain) to a gravitational-wave background (2.1) is:

8.73 $$\begin{aligned} \tilde{h}(f) = \int d^2\Omega _{\hat{n}}\> \sum _A R^A(f,\hat{n}) h_A(f,\hat{n}), \end{aligned}$$

or

8.74 $$\begin{aligned} \tilde{h}(f) = \sum _P \sum _{(lm)} R^P_{(lm)}(f)a^P_{(lm)}(f). \end{aligned}$$

#### Searches for non-GR polarizations using different detectors

Evidence for non-GR polarization modes can show up in searches for *either* deterministic or stochastic gravitational-wave signals. Whether these alternative polarization modes are first discovered from the observation of gravitational waves from a resolvable source (like a binary black hole merger) or from a stochastic background depends in part on the type and number of detectors making the observations. For example, individual binary black holes mergers (GW150914 and GW151226) have already been observed by advanced LIGO. But it was not possible to extract information about the polarization of the waves, since the two LIGO interferometers are effectively co-aligned (and hence see the *same* polarization). Adding Virgo, KAGRA, and LIGO-India to the global network will eventually allow for the extraction of this polarization information. Pulsar timing arrays, on the other hand, are expected to first detect a stochastic background from the inspirals of SMBHBs in the centers of distant galaxies (Rosado et al. 2015). So if evidence of alternative polarization modes are discovered by pulsar timing, it will most-likely first come from stochastic background observations.

In the following sections, we describe stochastic background search methods for non-GR polarization modes using both ground-based interferometers (Sect. 8.4) and pulsar timing arrays (Sect. 8.5). We will calculate antenna patterns, overlap functions, and discuss component separation for the tensor, vector, and scalar polarization modes. For ground-based interferometers, our discussion will be based on Nishizawa et al. (2009). For pulsar timing arrays, see Lee et al. (2008), Chamberlin and Siemens (2012) and Gair et al. (2015).

### Searches for non-GR polarizations using ground-based detectors

We now describe cross-correlation searches for non-GR polarization modes using a network of ground-based laser interferometers. For additional details, see Nishizawa et al. (2009).

#### Response functions

For ground-based interferometers in the small antenna limit, the strain response functions $R^A(f,\hat{n})$ for the different polarization modes $A=\{+,\times , X,Y, B, L\}$ are given by

8.75 $$\begin{aligned} R^A(f,\hat{n}) \simeq \frac{1}{2}(u^a u^b - v^a v^b)e^A_{ab}(\hat{n}), \end{aligned}$$

where $\hat{u}$, $\hat{v}$ are unit vectors pointing in the direction of the arms of the interferometer, and where we have chosen the origin of coordinates to be at the vertex of the interferometer. Note that there is no frequency dependence of the response function in the small-antenna limit. Assuming a $90^\circ$ opening angle between the interferometer arms, and choosing a coordinate system such that $\hat{u}$ and $\hat{v}$ point in the $\hat{x}$ and $\hat{y}$ direction, we find

8.76 $$\begin{aligned} \begin{aligned} R^+(\hat{n})&= \frac{1}{2}(1+\cos ^2\theta )\cos 2\phi , \quad&R^\times (\hat{n}) = -\cos \theta \sin 2\phi , \\ R^X(\hat{n})&= \sin \theta \cos \theta \cos 2\phi , \quad&R^Y(\hat{n}) = -\sin \theta \sin 2\phi , \\ R^B(\hat{n})&= -\frac{1}{2}\sin ^2\theta \cos 2\phi , \quad&R^L(\hat{n}) = \frac{1}{\sqrt{2}}\sin ^2\theta \cos 2\phi , \end{aligned} \end{aligned}$$

where we used (2.4) for our definition of $\{\hat{n},\hat{l},\hat{m}\}$.

**Fig. 64:**
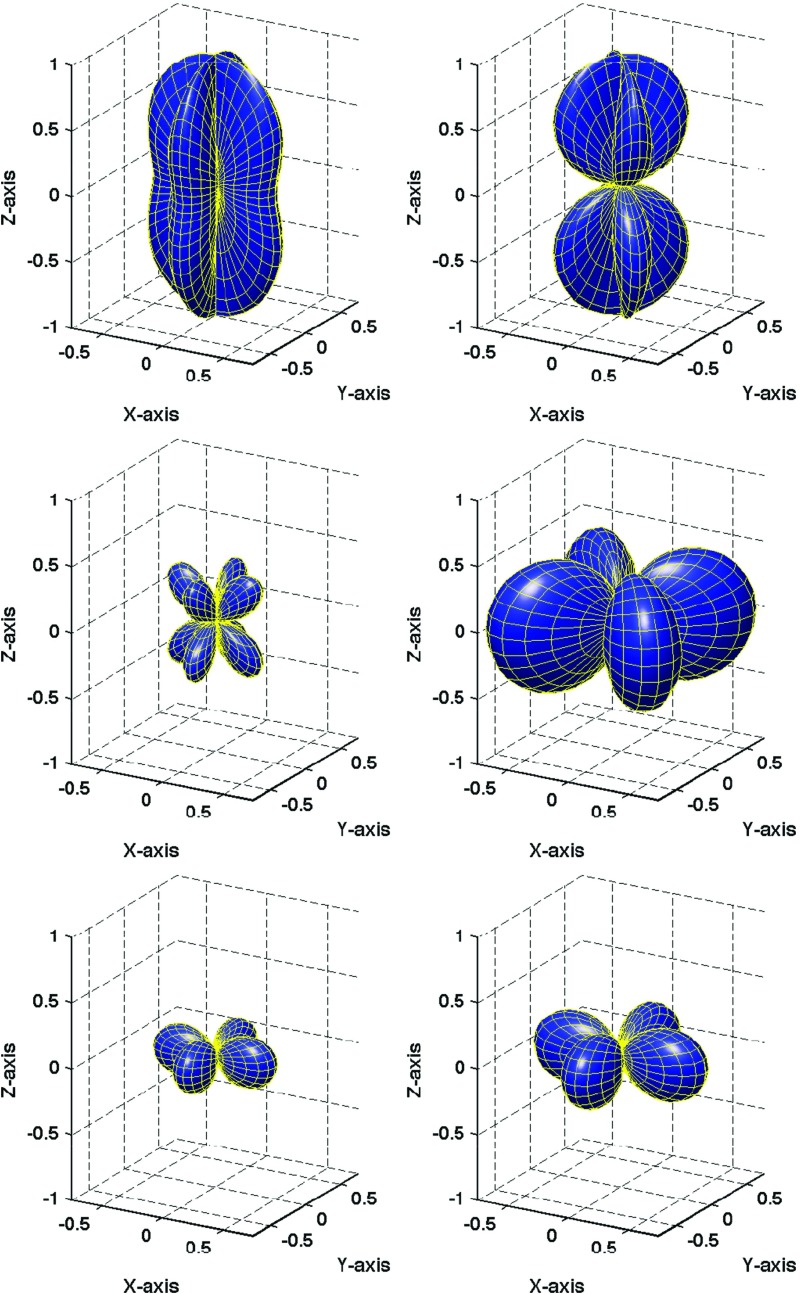
Antenna patterns for Michelson interferometer strain response $|R^A(\hat{n})|$ evaluated in the small-antenna limit, $f=0$. The *top two plots* correspond to the two tensor modes, $A=+, \times$. The *middle two plots* correspond to the two vector modes, $A=X, Y$. The *bottom two plots* correspond to the two scalar modes, $A=B, L$. The interferometer arms point in the $\hat{x}$ and $\hat{y}$ directions

**Fig. 65:**
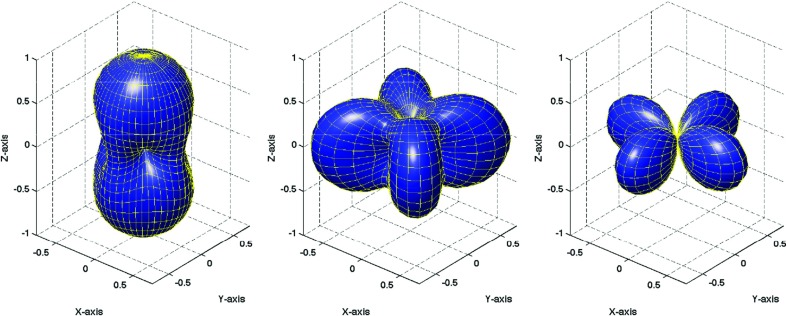
Antenna patterns for Michelson interferometer strain response to unpolarized gravitational waves for tensor (*left plot*), vector (*middle plot*), ans scalar modes (*right plot*), evaluated in the small antenna limit, $f=0$. The interferometer arms point in the $\hat{x}$ and $\hat{y}$ directions

From these expressions, we see that the response functions for the breathing and longitudinal modes differ only by a constant multiplicative factor of $-\sqrt{2}$. This degeneracy means that we will not be able to distinguish these two polarization modes using ground-based interferometers. Plots of the antenna patterns $|R^A(\hat{n})|$ for the six different polarization modes are shown in Fig. 64. Note that the overall magnitude of the response gets smaller as one moves from tensor, to vector, to scalar polarization modes. In Fig. 65, we plot the “peanut” antenna patterns for the response to unpolarized gravitational waves for the tensor, vector, and scalar modes, respectively. By unpolarized we simply mean that the incident gravitational waves have equal power in the $+$ and $\times$ polarizations for the tensor modes; equal power in the *X* and *Y* polarizations for the vector modes, and equal power in the *B* and *L* polarizations for the scalar modes.

#### Overlap functions

Similar to what we did in Sect. 8.2.2, let us assume that the stochastic background is *independently polarized*, but is otherwise Gaussian-stationary and isotropic. This means that the quadratic expectation values of the Fourier components of the metric perturbations can be written as

8.77 $$\begin{aligned} \langle h_A(f,\hat{n}) h_{A'}^*(f',\hat{n}')\rangle =\frac{1}{8\pi } S_h^{A}(f) \delta _{AA'} \delta (f-f') \delta ^2(\hat{n},\hat{n}'), \end{aligned}$$

where $A=\{+,\times ,X,Y,B,L\}$. The functions $S_h^A(f)$ are such that

8.78 $$\begin{aligned} \begin{aligned} S^{(T)}_h(f)&= S_h^+(f) + S_h^\times (f), \\ S^{(V)}_h(f)&= S_h^X(f) + S_h^Y(f), \\ S^{(S)}_h(f)&= S_h^B(f) + S_h^L(f), \end{aligned} \end{aligned}$$

are the one-sided strain spectral densities for the tensor, vector, and scalar modes individually. For simplicity, we will also assume that the tensor, vector, and scalar modes are individually unpolarized so that $S_h^+(f)=S_h^\times (f)$, $S_h^X(f)=S_h^Y(f)$, etc. All of these assumptions together define the stochastic signal model for this example.

The above expectation values (8.77) can now be used to calculate the expected value of the correlated response of two detectors to such a background. Writing the response of detector *I* as

8.79 $$\begin{aligned} \tilde{h}_I(f) = \int d^2\Omega _{\hat{n}}\> \sum _A R_I^A(f,\hat{n}) h_A(f,\hat{n}), \end{aligned}$$

it follows (as we have done many times before) that

8.80 $$\begin{aligned} \langle \tilde{h}_I(f)\tilde{h}_J^*(f')\rangle = \frac{1}{2}\delta (f-f') \left[ \Gamma ^{(T)}_{IJ}(f)S_h^{(T)}(f) + \Gamma ^{(V)}_{IJ}(f)S_h^{(V)}(f) + \Gamma ^{(S)}_{IJ}(f)S_h^{(S)}(f)\right] , \end{aligned}$$

where

8.81 $$\begin{aligned} \begin{aligned} \Gamma ^{(T)}_{IJ}(f)&\equiv \frac{1}{8\pi }\int d^2\Omega _{\hat{n}}\> \left[ R^+_I(f,\hat{n}) R^{+*}_J(f,\hat{n}) + R^\times _I(f,\hat{n}) R^{\times *}_J(f,\hat{n}) \right] , \\ \Gamma ^{(V)}_{IJ}(f)&\equiv \frac{1}{8\pi }\int d^2\Omega _{\hat{n}}\> \left[ R^X_I(f,\hat{n}) R^{X*}_J(f,\hat{n}) + R^Y_I(f,\hat{n}) R^{Y*}_J(f,\hat{n}) \right] , \\ \Gamma ^{(S)}_{IJ}(f)&\equiv \frac{1}{8\pi }\int d^2\Omega _{\hat{n}}\> \left[ R^B_I(f,\hat{n}) R^{B*}_J(f,\hat{n}) + R^L_I(f,\hat{n}) R^{L*}_J(f,\hat{n}) \right] , \end{aligned} \end{aligned}$$

are the corresponding overlap functions for the tensor, vector, and scalar modes $\{T,V,S\}$. Note that $\Gamma ^{(T)}_{IJ}(f)$ is identical to the ordinary overlap function $\Gamma _{IJ}(f)$ for an isotropic background (5.36).

**Fig. 66:**
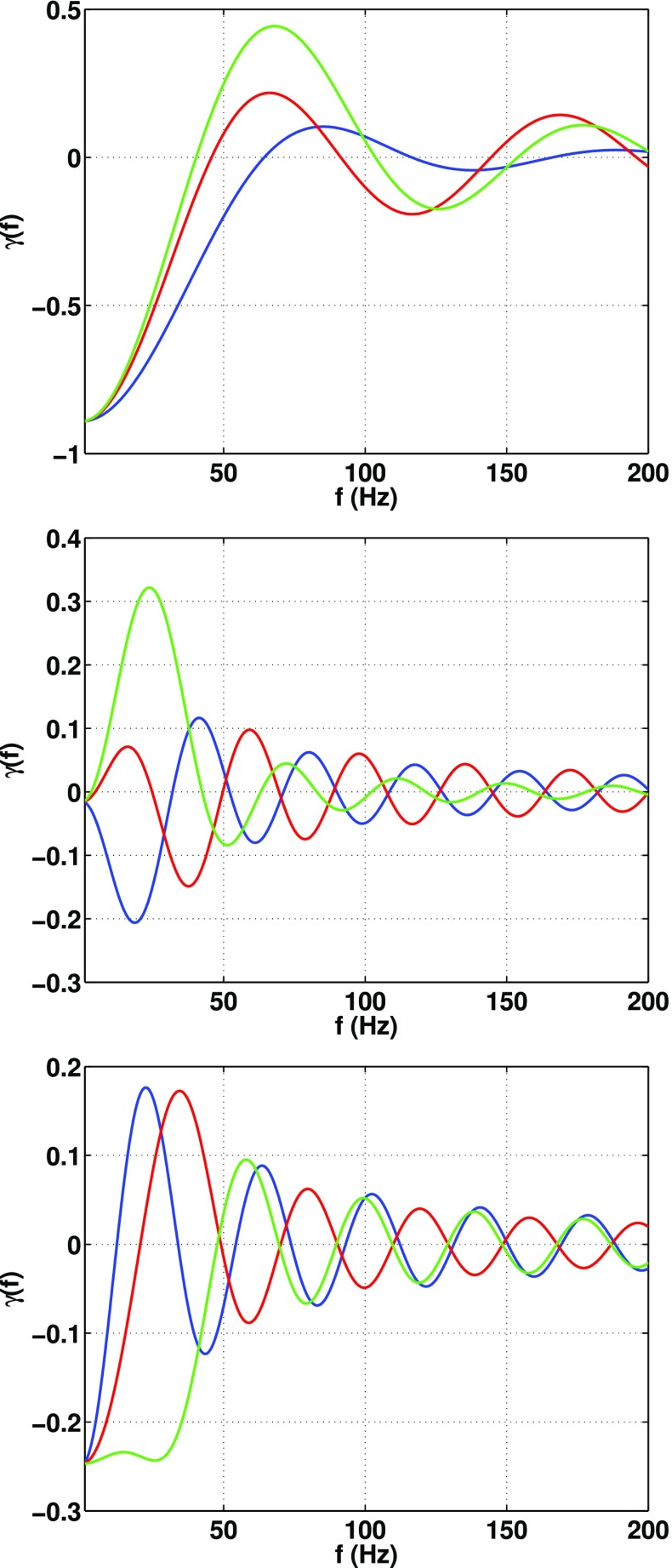
Normalized overlap functions for unpolarized tensor, vector, and scalar modes for the LIGO Hanford-LIGO Livingston detector pair (*top panel*); for the LIGO Hanford-Virgo detector pair (*middle panel*); and for the LIGO Livingston-Virgo detector pair (*bottom panel*). The tensor overlap functions are shown in *blue*; the vector overlap functions are shown in *red*; the scalar overlap functions are shown in *green*. These overlap functions were calculated in the small-antenna limit

Figure 66 show plots of the tensor, vector, and scalar overlap functions for the three different LIGO-Virgo detector pairs. The overlap functions have been normalized so that they equal 1 for colocated and coaligned detectors. This requires multiplying $\Gamma _{IJ}(f)$ by a factor of 5 for the tensor and vector overlap functions (5.40), but by a factor of 10 for the scalar overlap functions.

#### Component separation: ML estimates of $S_h^{(T)}$, $S_h^{(V)}$, and $S_h^{(S)}$

Proceeding along the same lines as in Sect. 8.2.3, we now describe a method for separating the tensor, vector, and scalar contributions to the total strain spectral density. As shown in Nishizawa et al. (2009), we will need to analyze data from at least three independent baselines (so at least three detectors) to separate the tensor, vector, and scalar contributions at each frequency *f*. As before, we will adopt the notation $\alpha =1,2,\ldots , N_b$ to denote the individual baselines (detector pairs) and $\alpha _1$, $\alpha _2$ to denote the two detectors that constitute that baseline.

Our starting point is again the cross-correlated data from pairs of detectors in the network:

8.82 $$\begin{aligned} \hat{C}_\alpha (f) \equiv \frac{2}{T}\,\tilde{d}_{\alpha _1}(f) \tilde{d}^*_{\alpha _2}(f), \end{aligned}$$

where

8.83 $$\begin{aligned} \tilde{d}_{\alpha _I}(f) = \tilde{h}_{\alpha _I}(\tilde{f}) + \tilde{n}_{\alpha _I}(f), \quad I=1,2. \end{aligned}$$

Assuming that the noise in the individual detectors are uncorrelated with one another, it follows that

8.84 $$\begin{aligned} \langle \hat{C}_\alpha (f) \rangle = \Gamma ^{(T)}_\alpha (f)S_h^{(T)}(f)+ \Gamma ^{(V)}_\alpha (f)S_h^{(V)}(f)+ \Gamma ^{(S)}_\alpha (f)S_h^{(S)}(f). \end{aligned}$$

In addition,

8.85 $$\begin{aligned} \begin{aligned} \mathcal{N}_{\alpha \alpha '}(f,f')&\equiv \langle \hat{C}_\alpha (f) \hat{C}^*_{\alpha '}(f')\rangle -\langle \hat{C}_\alpha (f)\rangle \langle \hat{C}^*_{\alpha '}(f')\rangle \\&\approx \delta _{\alpha \alpha '} \delta _{ff'} P_{n_{\alpha _1}}(f) P_{n_{\alpha _2}}(f), \end{aligned} \end{aligned}$$

where $P_{n_{\alpha _I}}(f)$ are the one-sided power spectral densities of the noise in the detectors, and where we have assumed again that the gravitational-wave signal is weak compared to the detector noise. As we did in Sect. 8.2.3 we can write down a likelihood function for the cross-correlated data given the signal model (8.84):

8.86 $$\begin{aligned} p(\hat{C}|\mathcal{A}) \propto \exp \left[ -\frac{1}{2}(\hat{C} - M\mathcal{A})^\dagger \mathcal{N}^{-1} (\hat{C} - M\mathcal{A})\right] . \end{aligned}$$

Here we have adopted the matrix notation:

8.87 $$\begin{aligned} M \equiv \left[ \begin{array}{ccc} \Gamma _1^{(T)} &{} \Gamma _1^{(V)} &{} \Gamma _1^{(S)} \\ \Gamma _2^{(T)} &{} \Gamma _2^{(V)} &{} \Gamma _2^{(S)} \\ \vdots &{} \vdots &{} \vdots \\ \Gamma _{N_b}^{(T)} &{} \Gamma _{N_b}^{(V)} &{} \Gamma _{N_b}^{(S)} \end{array} \right] , \quad \mathcal{A}\equiv \left[ \begin{array}{c} S_h^{(T)} \\ S_h^{(V)} \\ S_h^{(S)} \end{array} \right] . \end{aligned}$$

Since $\mathcal{A}$ enters quadratically in the exponential, we have the usual expression for the maximum-likehood estimators:

8.88 $$\begin{aligned} \hat{\mathcal{A}} =F^{-1} X, \end{aligned}$$

where

8.89 $$\begin{aligned} F\equiv M^\dagger \mathcal{N}^{-1} M, \quad X\equiv M^\dagger \mathcal{N}^{-1} \hat{C}, \end{aligned}$$

with *M* and $\mathcal{N}$ given above, and with the standard proviso about possibly having to use singular-value decomposition to invert *F*. The uncertainty in the maximum-likelihood recovered values is given by the covariance matrix

8.90 $$\begin{aligned} \langle \hat{\mathcal{A}}\hat{\mathcal{A}}^\dagger \rangle - \langle \hat{\mathcal{A}}\rangle \langle \hat{\mathcal{A}}^\dagger \rangle \approx F^{-1}, \end{aligned}$$

which we will use below to define *effective* overlap functions for the tensor, vector, and scalar modes for a multibaseline network of detectors.

#### Effective overlap functions for multiple baselines

For a multibaseline network of detectors, one has

8.91 $$\begin{aligned} F=\left[ \begin{array}{c@{\quad }c@{\quad }c} \sum _\alpha N_\alpha ^{-1}(\Gamma ^{(T)}_\alpha )^2 &{} \sum _\alpha N_\alpha ^{-1}\Gamma ^{(T)}_\alpha \Gamma ^{(V)}_\alpha &{} \sum _\alpha N_\alpha ^{-1}\Gamma ^{(T)}_\alpha \Gamma ^{(S)}_\alpha \\ \sum _\alpha N_\alpha ^{-1}\Gamma ^{(V)}_\alpha \Gamma ^{(T)}_\alpha &{} \sum _\alpha N_\alpha ^{-1}(\Gamma ^{(V)}_\alpha )^2 &{} \sum _\alpha N_\alpha ^{-1}\Gamma ^{(V)}_\alpha \Gamma ^{(S)}_\alpha \\ \sum _\alpha N_\alpha ^{-1}\Gamma ^{(S)}_\alpha \Gamma ^{(T)}_\alpha &{} \sum _\alpha N_\alpha ^{-1}\Gamma ^{(S)}_\alpha \Gamma ^{(V)}_\alpha &{} \sum _\alpha N_\alpha ^{-1}(\Gamma ^{(S)}_\alpha )^2 \\ \end{array} \right] , \end{aligned}$$

where $N_\alpha (f)\equiv P_{n_{\alpha _1}}(f) P_{n_{\alpha _2}}(f)$. Let us assume that the determinant of the $3\times 3$ matrices for each frequency (which we will denote by $\bar{F}$) are not equal to zero. Then the uncertainties in the estimators of $S_h^{(T)}$, $S_h^{(V)}$, and $S_h^{(S)}$ can be written as

8.92 $$\begin{aligned} \sigma _{\hat{T}}^2&= (\bar{F}^{-1})_{TT}\nonumber \\&= \frac{1}{\det (\bar{F})} \left( {\sum _\alpha N_\alpha ^{-1}(\Gamma ^{(V)}_\alpha )^2} \sum _{\alpha '} N_{\alpha '}^{-1}(\Gamma ^{(S)}_{\alpha '})^2- \left( \sum _\alpha N_\alpha ^{-1}\Gamma ^{(S)}_\alpha \Gamma ^{(V)}_\alpha \right) ^2 \right) ,\nonumber \\ \sigma _{\hat{V}}^2&= (\bar{F}^{-1})_{VV} \nonumber \\&= \frac{1}{\det (\bar{F})} \left( {\sum _\alpha N_\alpha ^{-1}(\Gamma ^{(T)}_\alpha )^2} \sum _{\alpha '} N_{\alpha '}^{-1}(\Gamma ^{(S)}_{\alpha '})^2- \left( \sum _\alpha N_\alpha ^{-1}\Gamma ^{(S)}_\alpha \Gamma ^{(T)}_\alpha \right) ^2 \right) ,\nonumber \\ \sigma _{\hat{S}}^2&= (\bar{F}^{-1})_{SS} \nonumber \\&= \frac{1}{\det (\bar{F})} \left( {\sum _\alpha N_\alpha ^{-1}(\Gamma ^{(T)}_\alpha )^2} \sum _{\alpha '} N_{\alpha '}^{-1}(\Gamma ^{(V)}_{\alpha '})^2- \left( \sum _\alpha N_\alpha ^{-1}\Gamma ^{(V)}_\alpha \Gamma ^{(T)}_\alpha \right) ^2 \right) .\nonumber \\ \end{aligned}$$

Following Nishizawa et al. (2009), we can now define the *effective* overlap functions for the tensor, vector, and scalar modes, associated with a multibaseline detector network. As we did in Sect. 8.2.5, we will assume for simplicity that the noise power spectra for the detectors are equal to one another so that $N_\alpha \equiv N$ can be factored out of the above expressions. We then define

8.93 $$\begin{aligned} \begin{aligned} \Gamma _\mathrm{eff}^{(T)}(f) \equiv \sigma _{\hat{T}}^{-1}\sqrt{N}&= \left( \frac{N^3 \det (\bar{F})}{\sum _\alpha (\Gamma ^{(V)}_\alpha )^2 \sum _{\alpha '}(\Gamma ^{(S)}_{\alpha '})^2- \left( \sum _\alpha \Gamma ^{(S)}_\alpha \Gamma ^{(V)}_\alpha \right) ^2} \right) ^{1/2}, \\ \Gamma _\mathrm{eff}^{(V)}(f) \equiv \sigma _{\hat{V}}^{-1}\sqrt{N}&= \left( \frac{N^3 \det (\bar{F})}{\sum _\alpha (\Gamma ^{(T)}_\alpha )^2 \sum _{\alpha '}(\Gamma ^{(S)}_{\alpha '})^2- \left( \sum _\alpha \Gamma ^{(S)}_\alpha \Gamma ^{(T)}_\alpha \right) ^2} \right) ^{1/2}, \\ \Gamma _\mathrm{eff}^{(S)}(f) \equiv \sigma _{\hat{S}}^{-1}\sqrt{N}&= \left( \frac{N^3 \det (\bar{F})}{\sum _\alpha (\Gamma ^{(T)}_\alpha )^2 \sum _{\alpha '}(\Gamma ^{(V)}_{\alpha '})^2- \left( \sum _\alpha \Gamma ^{(V)}_\alpha \Gamma ^{(T)}_\alpha \right) ^2} \right) ^{1/2}. \end{aligned} \end{aligned}$$

**Fig. 67:**
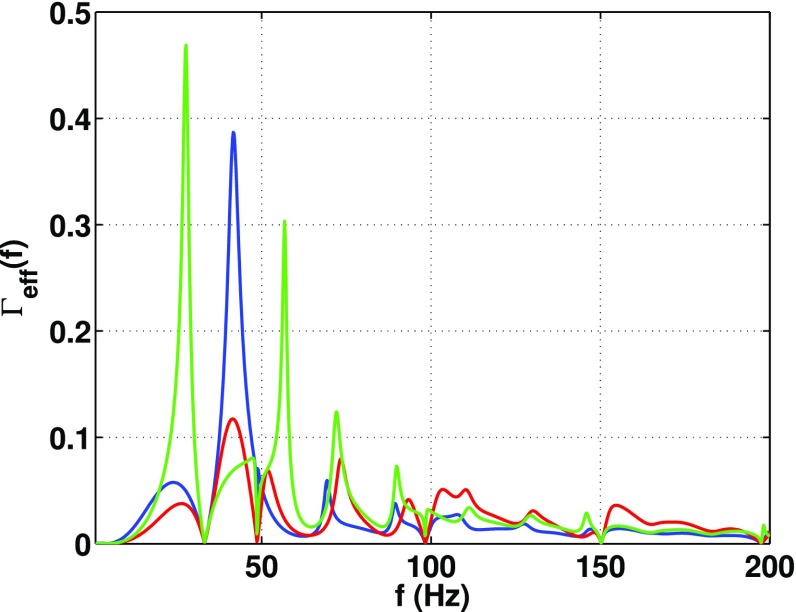
Effective overlap functions for $S_h^{(T)}$, $S_h^{(V)}$, $S_h^{(S)}$, for the multibaseline network formed from the LIGO Hanford, LIGO Livingston, and Virgo detectors. $\Gamma _\mathrm{eff}^{(T)}(f)$ is shown in *blue*; $\Gamma _\mathrm{eff}^{(V)}(f)$ is shown in *red*; $\Gamma _\mathrm{eff}^{(S)}(f)$ is shown in *green*

Plots of $\Gamma _\mathrm{eff}^{(T)}(f)$, $\Gamma _\mathrm{eff}^{(V)}(f)$, and $\Gamma _\mathrm{eff}^{(S)}(f)$ are shown in Fig. 67 for the multibaseline network formed from the LIGO Hanford, LIGO Livingston, and Virgo detectors. Dips in sensitivity correspond to frequencies where the determinant of $\bar{F}$ is zero (or close to zero).

### Searches for non-GR polarizations using pulsar timing arrays

As discussed in Sect. 8.3.4 it is also possible to search for non-GR polarizations using a pulsar timing array. Although the general concepts are the same as those for ground-based interferometers, there are some important differences, as the vector and scalar longitudinal polarization modes require keeping the pulsar term in the response functions to avoid possible singularities. We shall see below that the sensitivity to the vector and scalar longitudinal modes increases dramatically when cross-correlating data from pairs of pulsars with small angular separations. For additional details, see Lee et al. (2008), Chamberlin and Siemens (2012) and Gair et al. (2015).

#### Polarization basis response functions

For pulsar timing, the response functions for Doppler frequency measurements for the different polarization modes $A=\{+,\times , X,Y,B,L\}$ are given by

8.94 $$\begin{aligned} R^A(f,\hat{n}) = \frac{1}{2} \frac{\hat{p}^a\hat{p}^b}{1-\hat{n}\cdot \hat{p}} e^A_{ab}(\hat{n}) \left[ 1-e^{-\frac{i2\pi fL}{c}(1-\hat{n}\cdot \hat{p})} \right] , \end{aligned}$$

where $\hat{p}$ points in the direction to the pulsar and *L* is its distance from Earth (see Sect. 5.2.1 with $\hat{p}=-\hat{u}$). Without loss of generality, we have assumed that the location of the measurement is at the origin of coordinates. Note that we have kept the *pulsar term* (the second term in the square brackets) since, as we shall see below, it is needed to get finite expressions for the response and overlap functions for the vector and scalar longitudinal modes.

Choosing our coordinate system so that $\hat{z}$ points along $\hat{p}$, we find:

8.95 $$\begin{aligned} R^+(f,\hat{n})= & {} \frac{1}{2}(1+\cos \theta ) \left[ 1-e^{-\frac{i2\pi fL}{c}(1-\cos \theta )}\right] , \nonumber \\ R^\times (f,\hat{n})= & {} 0, \nonumber \\ R^X(f,\hat{n})= & {} -\frac{\sin \theta \cos \theta }{1-\cos \theta } \left[ 1-e^{-\frac{i2\pi fL}{c}(1-\cos \theta )}\right] , \nonumber \\ R^Y(f,\hat{n})= & {} 0, \nonumber \\ R^B(f,\hat{n})= & {} \frac{1}{2}(1+\cos \theta ) \left[ 1-e^{-\frac{i2\pi fL}{c}(1-\cos \theta )}\right] , \nonumber \\ R^L(f,\hat{n})= & {} \frac{1}{\sqrt{2}}\frac{\cos ^2\theta }{1-\cos \theta } \left[ 1-e^{-\frac{i2\pi fL}{c}(1-\cos \theta )}\right] , \end{aligned}$$

where we used (2.4) for our definitions of $\{\hat{n},\hat{l},\hat{m}\}$. Note that the response functions for the breathing mode *B* and the tensor $+$ mode have the same form for our particular choice of $\{\hat{l},\hat{m}\}$. This is not a problem, however, as we can still distinguish these modes due to their different behavior under rotations. The difference between the breathing and tensor modes becomes more apparent in terms of the spherical harmonic basis response functions $R^B_{(lm)}(f)$ and $R^G_{(lm)}(f)$, which are given in (8.98).

**Fig. 68:**
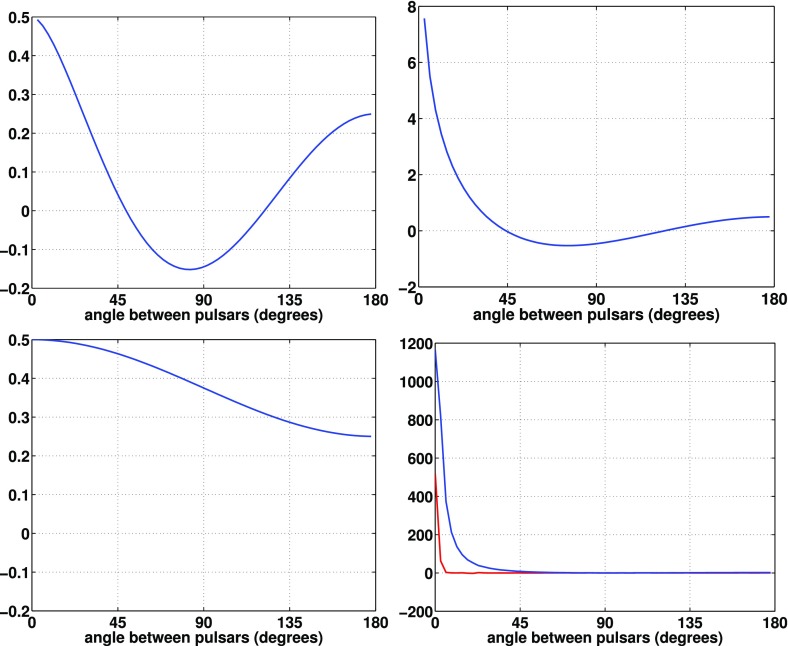
Normalized overlap functions for the tensor (*upper left*), vector-longitudinal (*upper right*), breathing (*lower left*), and scalar-longitudinal (*lower right*) polarization modes, plotted as functions of the angular separation between pairs of pulsars. The *blue and red curves* in the *lower right-hand plot* correspond to the real and imaginary parts of the scalar-longitudinal overlap function. Note the different vertical scales for the vector-longitudinal and scalar-longitudinal overlap functions, compared to those for the tensor and breathing modes

If we did not include the pulsar terms in the above expressions, then the response functions for both the vector and scalar longitudinal modes would become singular at $\theta = 0$ (i.e., $\cos \theta =1$).^24^ The factor of $\sin \theta$ in the numerator for $R^X(f,\hat{n})$ “softens” the $(1-\cos \theta )^{-1}$ singularity to $(1-\cos \theta )^{-1/2}$, so that it becomes integrable when calculating the vector longitudinal overlap functions (Lee et al. 2008; Chamberlin and Siemens 2012; Gair et al. 2015). (We will discuss this in more detail in Sect. 8.5.3). By keeping the pulsar term we remove these singularities as can be seen by expanding the full expressions in (8.95) for $\theta \ll 1$:

8.96 $$\begin{aligned} \begin{aligned} R^+(f,\hat{n})&\approx i y \theta ^2/2, \\ R^\times (f,\hat{n})&=0, \\ R^X(f,\hat{n})&\approx -i y \theta , \\ R^Y(f,\hat{n})&=0, \\ R^B(f,\hat{n})&\approx i y \theta ^2/2, \\ R^L(f,\hat{n})&\approx i y/\sqrt{2}, \end{aligned} \end{aligned}$$

where $y\equiv 2\pi fL/c$, and we have assumed that $y\theta ^2$ is also sufficiently small that we could Taylor expand the exponential. Since the typical distance to a pulsar is a few kiloparsecs and $f= 3 \times 10^{-9}~\mathrm{Hz}$ for 10 yr of observation, we have $y~\sim 10^4$, which means $\theta \lesssim 10^{-2}$ for the above expansions to be valid. Thus, for small angular separations between the direction to the pulsar and the direction to the gravitational wave, the response to the scalar-longitudinal modes will be more than an order-of-magnitude larger than that for the vector modes, and several orders-of-magnitude larger than that for both the tensor and breathing modes. This increased sensitivity of the scalar longitudinal and vector longitudinal modes will also become apparent when we calculate the overlap functions for a pair of pulsars (see Sect. 8.5.3; Fig. 68).

#### Spherical harmonic basis response functions

It is also interesting to calculate the Doppler-frequency response functions for the tensor spherical harmonic components $P=\{G, C, V_G, V_C, B, L\}$. The general expression is given by:

8.97 $$\begin{aligned} R^P_{(lm)}(f) = \int d^2\Omega _{\hat{n}}\> \frac{1}{2} \frac{\hat{p}^a\hat{p}^b}{1-\hat{n}\cdot \hat{p}} Y^P_{(lm)ab}(\hat{n}) \left[ 1-e^{-\frac{i2\pi fL}{c}(1-\hat{n}\cdot \hat{p})} \right] . \end{aligned}$$

As shown in Gair et al. (2015), the above integral can be evaluated and then simplified by taking the limit $y \gg 1$, which as we mentioned above is valid for typical pulsars. The final results (taken from that paper) are:

8.98 $$\begin{aligned} \begin{aligned} R^G_{(lm)}(f)&\approx 2\pi \,{}^{(2)}\!N_l Y_{lm}(\hat{p}),&\quad l=2,3,\ldots , \\ R^C_{(lm)}(f)&\approx 0,&\quad l=2,3,\ldots , \\ R^{V_G}_{(lm)}(f)&\approx 2\pi \,{}^{(1)}\!N_l \left[ 1-\frac{2}{3}\delta _{l1}\right] Y_{lm}(\hat{p}),&\quad l=1,2,\ldots , \\ R^{V_C}_{(lm)}(f)&\approx 0,&\quad l=1,2,\ldots , \\ R^B_{(lm)}(f)&\approx 2\pi \, \frac{1}{\sqrt{2}} \left[ \delta _{l0} + \frac{1}{3}\delta _{l1}\right] Y_{lm}(\hat{p}),&\quad l=0,1,\ldots , \\ R^L_{(lm)}(f)&\approx 2\pi \left[ -\delta _{l0}-\frac{1}{3} \delta _{l1} + \frac{1}{2}\bar{H}_l(y)\right] Y_{lm}(\hat{p}),&\quad l=0,1,\ldots , \end{aligned} \end{aligned}$$

where ${}^{(1)}\!N_l$ and ${}^{(2)}\!N_l$ are constants defined by (F.3) and (G.2), and

8.99 $$\begin{aligned} \bar{H}_l(y) \equiv \int _{-1}^1 dx\> \frac{1}{(1-x)} P_l(x) \left[ 1-e^{-iy(1-x)}\right] . \end{aligned}$$

There are several important features to highlight about these expressions: (i) All of the response functions depend in the same way on the angular position of the pulsar, which is simply $Y_{lm}(\hat{p})$. (ii) Just as we saw earlier (5.23) that the response to the tensor curl mode is zero, so too is the response to the vector curl mode. *Thus, pulsar timing arrays are also insensitive to the curl component of the vector-longitudinal modes.* (iii) In the limit $y\gg 1$, only the response to the scalar-longitudinal mode has frequency dependence (via *y*). (iv) The response to the breathing mode has non-zero contributions only from $l=0$ and $l=1$. In terms of power (which is effectively the square of the response), this means that pulsar timing observations will be insensitive to anisotropies in power in the breathing mode beyond quadrupole (i.e., $l=2$).

#### Overlap functions

To calculate the overlap functions for non-GR polarization modes for pulsar timing arrays, we will proceed as we did in Sect. 8.4.2, assuming that the stochastic background is independently polarized, but is otherwise Gaussian-stationary and isotropic. (Extensions to *anisotropic* backgrounds will be briefly mentioned in Sect. 8.5.4. Details can be found in Gair et al. 2015). Making these assumptions, the quadratic expectation values of the Fourier coefficients $h_A(f,\hat{n})$ take the form

8.100 $$\begin{aligned} \langle h_A(f,\hat{n}) h_{A'}^*(f',\hat{n}')\rangle =\frac{1}{8\pi } S_h^{A}(f) \delta _{AA'} \delta (f-f') \delta ^2(\hat{n},\hat{n}'), \end{aligned}$$

where $S^A_h(f)$ are the one-sided strain spectral densities for the individual polarization modes. The overlap functions can then be calculated in the usual way, leading to

8.101 $$\begin{aligned} \langle \tilde{h}_I(f)\tilde{h}_J^*(f')\rangle =\frac{1}{2}\delta (f-f')\sum _A \Gamma ^A_{IJ}(f) S_h^A(f), \end{aligned}$$

where

8.102 $$\begin{aligned} \Gamma ^{A}_{IJ}(f) \equiv \frac{1}{4\pi } \int d^2\Omega _n R^A_I(f,\hat{n})R_J^{A*}(f,\hat{n}). \end{aligned}$$

Note the factor of $1/4\pi$ as compared to $1/8\pi$ in (8.81), and that there is no summation over *A* on the right-hand side of this expression.

For simplicity we will also assume as before that the tensor modes $\{+,\times \}$ and the vector-longitudinal modes $\{X, Y\}$ are unpolarized, so that

8.103 $$\begin{aligned} \begin{aligned} S_h^+(f)=S_h^\times (f)\equiv \frac{1}{2} S_h^{(T)}(f), \\ S_h^X(f)=S_h^Y(f)\equiv \frac{1}{2} S_h^{(V)}(f). \end{aligned} \end{aligned}$$

Then we can define:

8.104 $$\begin{aligned} \begin{aligned} \Gamma ^{(T)}_{IJ}(f)&\equiv \frac{1}{8\pi }\int \!d^2\Omega _{\hat{n}}\> \left[ R^+_I(f,\hat{n}) R^{+*}_J(f,\hat{n}) + R^\times _I(f,\hat{n}) R^{\times *}_J(f,\hat{n}) \right] , \\ \Gamma ^{(V)}_{IJ}(f)&\equiv \frac{1}{8\pi }\int \! d^2\Omega _{\hat{n}}\> \left[ R^X_I(f,\hat{n}) R^{X*}_J(f,\hat{n}) + R^Y_I(f,\hat{n}) R^{Y*}_J(f,\hat{n}) \right] , \end{aligned} \end{aligned}$$

for the unpolarized tensor and vector-longitudinal components. But we will keep the breathing and scalar-longitudinal overlap functions separate:

8.105 $$\begin{aligned} \begin{aligned} \Gamma ^{B}_{IJ}(f)&\equiv \frac{1}{4\pi }\int d^2\Omega _{\hat{n}}\> R^B_I(f,\hat{n}) R^{B*}_J(f,\hat{n}), \\ \Gamma ^{L}_{IJ}(f)&\equiv \frac{1}{4\pi }\int d^2\Omega _{\hat{n}}\> R^L_I(f,\hat{n}) R^{L*}_J(f,\hat{n}), \end{aligned} \end{aligned}$$

given the complications that arise when trying to explicitly calculate $\Gamma ^L_{IJ}(f)$.

As noted in Sect. 5.4.3, the overlap function for the tensor modes can be calculated analytically (Hellings and Downs 1983), without needing to include the pulsar term in the response functions:

8.106 $$\begin{aligned} \Gamma ^{(T)}_{IJ} = \frac{1}{3} \left[ \frac{3}{2}\left( \frac{1-\cos \zeta _{IJ}}{2}\right) \ln \left( \frac{1-\cos \zeta _{IJ}}{2}\right) -\frac{1}{4}\left( \frac{1-\cos \zeta _{IJ}}{2}\right) +\frac{1}{2}\right] ,\nonumber \\ \end{aligned}$$

where $\zeta _{IJ}$ is the angle between two Earth-pulsar baselines, i.e., $\cos \zeta _{IJ} = \hat{p}_I\cdot \hat{p}_J$. The above expression differs from (5.53) by an overall normalization. The overlap functions for the breathing mode and for the vector longitudinal modes can be also be calculated analytically, again without needing to include the pulsar term in the response. For the breathing mode we have

8.107 $$\begin{aligned} \Gamma ^B_{IJ} = \frac{2}{3} \left[ \frac{3}{8} + \frac{1}{8}\cos \zeta _{IJ}\right] . \end{aligned}$$

For the vector-longitudinal modes we have (Lee et al. 2008; Gair et al. 2015)

8.108 $$\begin{aligned} \Gamma ^{(V)}_{IJ} = \frac{1}{3} \left[ \frac{3}{2}\ln \left( \frac{2}{1-\cos \zeta _{IJ}}\right) - 2\cos \zeta _{IJ} - \frac{3}{2} \right] , \end{aligned}$$

where we have assumed here that the angular separation $\zeta _{IJ}$ is not too small. In the limit $\zeta _{IJ}\rightarrow 0$, the above expression for $\Gamma ^{(V)}_{IJ}$ diverges, which means that we need to include the pulsar terms in the response functions to handle that case. Doing so results in an expression that is finite, but depends on the frequency *f* via the distances to the pulsars, $2\pi fL_I/c$ and $2\pi fL_J/c$. (See Appendix J of Gair et al. (2015) for an analytic expression for $\Gamma ^{(V)}_{IJ}(f)$ in the limit $\zeta _{IJ}\rightarrow 0$.)

Finally, for the scalar longitudinal overlap function $\Gamma ^L_{IJ}(f)$, there is no known analytic expression for the integral in (8.105), except in the limit of codirectional ($\zeta _{IJ}=0$) and anti-directional ($\zeta _{IJ}=\pi$) pulsars (Lee et al. 2008; Chamberlin and Siemens 2012; Gair et al. 2015). The pulsar terms need to be included in the scalar-longitudinal response functions for all cases to obtain a finite result, which again depend on the frequency *f* via the distances to the pulsars. A semi-analytic expression for $\Gamma ^L_{IJ}(f)$ is derived in Gair et al. (2015), which is valid in the $2\pi fL/c \gg 1$ limit. The semi-analytic expression effectively replaces the double integral over directions on the sky $\hat{n}=(\theta ,\phi )$ with just a single numerical integration over $\theta$. See Gair et al. (2015) for additional details regarding that calculation.

Plots of the normalized overlap functions for the tensor, vector-longitudinal, breathing and scalar-longitudinal modes are shown in Fig. 68, plotted as functions of the angular separation $\zeta$ between pairs of pulsars. The normalization is the same for each overlap function, chosen so that the tensor overlap function agrees with the normalized Hellings and Downs curve (5.53). The plots for the tensor, vector-longitudinal, and breathing modes are all real and do not depend on frequency; the plot for the scalar-longitudinal modes has both real and imaginary components (imaginary shown in red), and depends on frequency via the distances to the pulsars. For the scalar-longitudinal overlap function, we chose $y_1=1000$ and $y_2=2000$ for all pulsar pairs, where $y\equiv 2\pi fL/c$, and we did the integration numerically over both $\theta$ and $\phi$. Note the different vertical scales for the vector-longitudinal and scalar-longitudinal overlap functions, compared to those for the tensor and breathing modes. For small angular separations, the sensitivity to vector-longitudinal modes is roughly an order of magnitude larger than that for the tensor and breathing modes, while the sensitivity to the scalar-longitudinal mode is several orders-of-magnitude larger. This is consistent with what we found for the response functions, as discussed at the end of Sect. 8.5.1.

#### Component separation and anisotropic backgrounds

As shown in Gair et al. (2015), the above calculations for non-GR polarization modes can be extended to *anisotropic* stochastic backgrounds. The spherical harmonic components of the overlap functions

8.109 $$\begin{aligned} \Gamma ^A_{lm}(f) =\frac{1}{4\pi }\int \! d^2\Omega _{\hat{n}}\> Y_{lm}(\hat{n})R^A_I(f,\hat{n})R^{A*}_J(f,\hat{n}) \end{aligned}$$

can be calculated *analytically* for the tensor, vector, and breathing polarization modes for all values of *l* and *m*, while the components of the scalar longitudinal overlap function admit only semi-analytic expressions. (This is similar to what we described in the previous section in the context of an isotropic background). Plots of the first few spherical harmonics components, as a function of the angular separation $\zeta _{IJ}$ between a pair of pulsars, are given in Figures 1, 5, 2, and 3 of Gair et al. (2015).

The ability to separate the contributions to the background from the different polarization modes depends crucially on the form of the spherical harmonic basis response functions $R^P_{(lm)}(f)$, where $P=\{G,C, V_G, V_C, B,L\}$. These were defined in (8.97) and have the $y\equiv 2\pi fL/c \gg 1$ limiting expressions given in (8.98). Recall that the (*lm*) indices here correspond to an expansion of the Fourier components of the metric perturbations in terms of tensor (spin 2), vector (spin 1), and scalar (spin 0) spherical harmonics:

8.110 $$\begin{aligned} h_{ab}(f,\hat{n}) = \sum _P \sum _{(lm)} a_{(lm)}^P(f) Y^P_{(lm)ab}(\hat{n}), \end{aligned}$$

for which

8.111 $$\begin{aligned} \tilde{h}_I(f) = \sum _P \sum _{(lm)} R^P_{I(lm)}(f)a^P_{(lm)}(f) \end{aligned}$$

is the response of pulsar *I* to the background. The expansion coefficients $a_{(lm)}^P(f)$ give the contributions of the different polarization modes to the background, and $R^P_{I(lm)}(f)$ are the response functions for those particular coefficients. For an angular resolution of order $180^\circ /l_\mathrm{max}$, the total number of modes that are (in principle) accessible to a pulsar timing array with a sufficient number of pulsars is

8.112 $$\begin{aligned} N_m= 3(l_\mathrm{max}+1)^2 - 1. \end{aligned}$$

This expression uses the result that the response to the curl modes for both the tensor and vector components are identically zero, as is the response to the breathing modes for $l\ge 2$. Since a pulsar timing array having $N_p$ pulsars can measure at most $2N_p$ real components of the background (as discussed in Sect. 7.5.4), we see that at least $N_p = N_m$ pulsars are required to measure the $N_m$ (complex) components.

But as noted in Sect. 8.5.2, all of the response functions $R^P_{(lm)}(f)$ depend on the direction $\hat{p}$ to the pulsar in exactly the same way, being proportional to $Y_{lm}(\hat{p})$. This degeneracy complicates the extraction of the different polarization modes. For the tensor and breathing modes, the degeneracy is broken since pulsar timing arrays typically operate in a regime where $y\gg 1$, for which the pulsar term can be ignored in the response functions for these modes. In that limit, a pulsar timing array is only sensitive to breathing modes with $l=0, 1$, while the tensor modes are non-zero only for $l\ge 2$. On the other hand, the scalar-longitudinal and vector-longitudinal modes can only be distinguished from the tensor and breathing modes if there are multiple pulsars along the same line of sight, or if there is a known correlation between the expansion coefficients $a^P_{(lm)}(f)$ at different frequencies, e.g., a power-law spectrum. For either of these two cases, we can exploit the frequency dependence of the pulsar term, which is more significant for the longitudinal modes of the background. Keeping all of the frequency-dependent terms (Gair et al. 2015):

8.113 $$\begin{aligned} R^L_{(lm)}(f)= & {} 2\pi (-1)^l \bigg \{ -\delta _{l0} + \frac{1}{3} \delta _{l1}+(-i)^l \mathrm{e}^{-iy} \left[ \left( 1-i\frac{l}{y}\right) j_l(y) + i j_{l+1}(y)\right] \nonumber \\&+\, \frac{1}{2}H_l(y)\bigg \} Y_{lm}(\hat{p}), \end{aligned}$$

and

8.114 $$\begin{aligned} R^{V_G}_{(lm)}(f)= & {} \pi (-1)^l\,{}^{(1)}\!N_l \bigg \{\frac{4}{3} \delta _{l1} + 2 (-i)^l \mathrm{e}^{-iy} \bigg [\left( 1-\frac{il}{y}\right) (l+1)j_l(y) \nonumber \\&-\, (y-i(2l+3))j_{l+1}(y) - iy j_{l+2}(y) \bigg ] \bigg \} Y_{lm}(\hat{p}), \end{aligned}$$

for the scalar-longitudinal and vector-longitudinal response functions, where $j_l(y)$ denotes a spherical Bessel functions of order *l* and $y\equiv 2\pi fL/c$. If we take the $y\gg 1$ limit of these equations, we recover the approximate expressions given in (8.98). But to separate the various components of the background, we need to use these more complicated expressions to break the angular-direction degeneracy.

A quantitative analysis of the sensitivity of a phase-coherent mapping search (Sect. 7.5) to the different components $a^P_{(lm)}(f)$ of a stochastic background is given in Gair et al. (2015). The results of that analysis are summarized in Table 7, which is taken from that paper. The entries in the table show how the uncertainties in our measurements change as we search for: (i) only the tensor modes, (ii) both tensor and breathing modes, (iii) tensor, breathing, and scalar-longitudinal modes, and (iv) all possible modes. The uncertainties were obtained by taking the square root of the diagonal elements of the inverse of the Fisher matrix, following the general prescription described in Sect. 7.5.1. For this calculation, 30 pulsars were distributed randomly on the sky, with distances chosen at random, uniformly between 1 and 10 kpc. There was only a single frequency component, $f_0=3\times 10^{-9}~\mathrm{Hz}$, and the measurement uncertainty (associated with pulse time of arrivals) was assumed to be the same for all the pulsars in the array. The background was also assumed to contain modes with equal intrinsic amplitudes up to $l_\mathrm{max}=2$, so that the total number of modes $N_m=26$ was less than the number of pulsars in the array. This gave a fully-determined system of equations that needed to be solved.

**Table 7:** Relative uncertainties for the tensor, breathing, scalar-longitudinal, and vector-longitudinal polarization modes searched for separately or in various combinations for $l_\mathrm{max}=2$ and $N_p=30$ pulsars

	(*l*, *m*) mode								
	(0, 0)	$(1,-1)$	(1, 0)	(1, 1)	$(2,-2)$	$(2,-1)$	(2, 0)	(2, 1)	(2, 2)
Tensor	−	−	−	−	0.44	0.38	0.32	0.38	0.44
Tensor	−	−	−	−	0.49	0.39	0.37	0.39	0.49
Breathing	0.16	0.53	0.46	0.53	−	−	−	−	−
Tensor	−	−	−	−	16.2	10.5	11.4	10.5	16.2
Breathing	4.36	16.1	14.1	16.1	−	−	−	−	−
Longitudinal	0.71	0.96	0.84	0.96	1.21	0.78	0.86	0.78	1.21
Tensor	−	−	−	−	1.4e5	5.4e4	8.0e4	5.4e4	1.4e5
Breathing	18.4	9.4e4	6.2e4	9.4e4	−	−	−	−	−
Longitudinal	3.08	11.5	8.68	11.5	20.9	7.51	11.9	7.52	20.9
Vector	−	6.6e4	4.4e4	6.6e4	7.0e4	2.7e4	4.0e4	2.7e4	7.0e4

This table is adapted from Table II in Gair et al. (2015)

The entries in the table reflect our expectations for recovering the different modes of the background. Namely, there is little change in our ability to recover the tensor modes when the breathing modes are also included in the analysis. This is because the tensor modes are non-zero only for $l\ge 2$, while the response to the breathing modes is non-zero only for $l=0,1$. Adding the scalar-longitudinal modes to the analysis worsens the recovery of the tensor and breathing modes by about an order of magnitude, as the scalar-longitudinal modes can also have non-zero values for all values of *l*. (There are simply more parameters to recover). But one is still able to break the degeneracy as the response to the scalar-longitudinal modes depends *strongly* on the distances to the pulsars. The uncertainity in the recovery of the scalar-longitudinal modes is about an order of magnitude less than that for the tensor and breathing modes, since the analysis assumes equal intrinsic amplitudes for all the modes, while the correlated response to the scalar-longitudinal modes is much larger for small angular separations between the pulsars (Sect. 8.5.3; Fig. 68). Finally, adding the vector-longitudinal modes to the analysis weakens the recovery of the scalar-longitudinal modes by about an order of magnitude, again because more parameters need to be recovered. However, it *severely worsens* the recovery of all the other modes, because of the degeneracy in the response on the angular direction to the pulsars. There is some dependence on frequency for the vector-longitudinal response, as indicated in (8.114), but it is much weaker than the frequency dependence of the scalar-longitudinal modes. So the degeneracy is not broken nearly as strongly for these modes. See Gair et al. (2015) for more details.

### Other searches

It is also possible to use the general cross-correlation techniques described in Sect. 4 to search for signals that don’t really constitute a stochastic gravitational-wave background. Using a stochastic-based cross-correlation method to search for such signals is not optimal, but it still gives valid results for detection statistics or estimators of signal parameters, with error bars that properly reflect the uncertainty in these quantities. It is just that these error bars are *larger* than those for an optimal (minimum variance) search, which is better “tuned” for the signal. Below we briefly describe how the general cross-correlation method can be used to search for (i) long-duration unmodelled transients and (ii) persistent (or continuous) gravitational waves from targeted sources.

#### Searches for long-duration unmodelled transients

The Stochastic Transient Analysis Multi-detector Pipeline (Thrane et al. 2011) (STAMP for short) is a cross-correlation search for unmodelled long-duration transient signals (“bursts”) that last on order a few seconds to several hours or longer. The duration of these transients are long compared to the typical merger signal from inspiralling binaries (tens of milliseconds to a few seconds), but short compared to the persistent quasi-monochromatic signals that one expects from e.g., rotating (non-axisymmetric) neutron stars. STAMP was developed in the context of ground-based interferometers, but the general method, which we briefly describe below, is also valid for other types of gravitational-wave detectors.

STAMP is effectively an adapted gravitational-wave radiometer search (Sect. 7.3.6), which cross-correlates data from pairs of detectors (7.6), weighted by the *inverse* of the integrand of the overlap function $\gamma _{IJ}(t;f,\hat{n})$ for a particular direction $\hat{n}$ on the sky:

8.115 $$\begin{aligned} \tilde{s}_{IJ}(t;f,\hat{n})\equiv \frac{2}{\tau } \frac{\tilde{d}_I(t;f)\tilde{d}_J^*(t;f)}{\gamma _{IJ}(t;f,\hat{n})}, \end{aligned}$$

where $\tau$ is the duration of the segments defining the short-term Fourier transforms. The weighting by the inverse of $\gamma _{IJ}(t;f,\hat{n})$ is used so that the expected value of $\tilde{s}_{IJ}(t;f,\hat{n})$ is just the gravitational-wave power in pixel (*t*; *f*) for a point source in direction $\hat{n}$, which follows from (7.7). The data $\tilde{s}_{IJ}(t;f,\hat{n})$ for a single direction $\hat{n}$ define a *time-frequency map*. For a typical analysis using the LIGO Hanford and LIGO Livingston interferometer, a single map has a frequency range from about 50 to ${\sim }1000~\mathrm{Hz}$, and a time duration of a couple hundred seconds (or whatever the expected duration of the transient might be). A strong burst signal shows up as *cluster* or *track* of bright pixels in the time-frequency map, which stands out above the noise. The data analysis problem thus becomes a *pattern recognition* problem.

The procedure for deciding whether or not a signal is present in the data can be broken down into three steps: (i) determine if a statistically significant clump or track of bright pixels is present in a time-frequency map, which requires using some form of pattern-recognition or clustering algorithm (see Thrane et al. 2011 and relevant references cited therein); (ii) calculate the value of the detection statistic $\Lambda$, obtained from a weighted sum of the power in the pixels for each cluster determined by the previous step; (iii) compare the observed value of the detection statistic to a threshold value $\Lambda _*$, which depends on the desired false alarm rate. This threshold is typically calculated by time-shifting the data to empirically determine the sampling distribution of $\Lambda$ in the absence of a signal. If $\Lambda _\mathrm{obs}>\Lambda _*$, then reject the null hypothesis and claim detection as discussed in 3.2.1. (Actually, in practice, this last step is a bit more complicated, as one typically does follow-up investigations using auxiliary instrumental and environmental channels, and data quality indicators. This provides additional confidence that the gravitational-wave candidate is not some spurious instrumental or environmental artefact.)

**Fig. 69:**
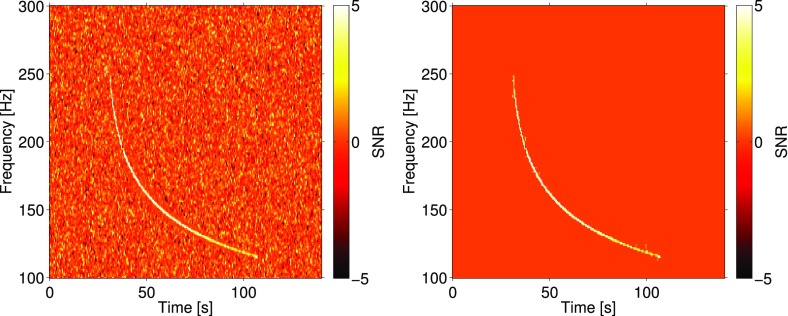
Time-frequency maps for an injected long-duration transient gravitational-wave signal in noise. *Left panel* signal-to-noise ratio map before processing. *Right panel* signal-to-noise ratio map after applying a clustering algorithm. Note that the noise fluctuations have effectively been eliminated in the second plot. Images provided by Tanner Prestegard

Figure 69 is an example of a time-frequency map with a simulated long-duration gravitational-wave signal injected into simulated initial LIGO detector noise. This particular signal is an *accretion disk instability waveform*, based on a model by van Putten (2001); van Putten and Levinson (2003); van Putten (2008). The signal is a (inverse) “chirp” in gravitational radiation having an exponentially decaying frequency. (The magnitude of the signal increases with time as the frequency *decreases*.) The injected signal is strong enough to be seen by eye in the raw time-frequency map (left panel). After applying a clustering algorithm, the fluctuations in the detector noise have been noticeably reduced (right panel).

Readers should see Thrane et al. (2011) for many more details regarding STAMP, and Abbott et al. (2016c) and Aasi et al. (2013) for results from analyses of LIGO data taken during their 5th and 6th science runs—the first paper describes an all-sky search for long-duration gravitational-wave transients; the second, a triggered-search for long-duration gravitational-transients coincident with long duration gamma-ray bursts.

#### Searches for targeted-sources of continuous gravitational waves

The gravitational-wave radiometer method (Sect. 7.3.6) can also be used to look for gravitational waves from persistent (continuous) sources at known locations on the sky, e.g., the galactic center, the location of SN 1987A, or from low-mass X-ray binaries like Sco X-1 (Abadie et al. 2011; Messenger et al. 2015; Abbott et al. 2016a). For example, Sco X-1 is expected to emit gravitational waves from the (suspected) rotating neutron star at its core, having non-axisymmetric distortions produced by the accretion of matter from the low-mass companion. The parameters of this system that determine the phase evolution of the gravitational radiation are not well-constrained: (i) Since the neutron star at the core has not been observed to emit pulsations in the radio or any electromagnetic band, the orbital parameters of the binary are estimated instead from optical observations of the low-mass companion (Steeghs and Casares 2002; Galloway et al. 2014). These observations do not constrain the orbital parameters as tightly as being able to directly monitor the spin frequency of the neutron star. (ii) The intrinsic spin evolution of the neutron star also has large uncertainties due to the high rate of accretion from the low-mass companion star. Both of these features translate into a *large* parameter space volume over which to search, making fully-coherent matched-filter searches for the gravitational-wave signal computationally challenging (Messenger et al. 2015).

Nonetheless, for such sources, one can perform a *narrow-band, targeted* radiometer search, cross-correlating data from a pair of detectors with a filter function proportional to the integrand $\gamma _{IJ}(t;f,\hat{n}_0)$ of the overlap function evaluated at the direction $\hat{n}_0$ to the source on the sky:

8.116 $$\begin{aligned} \hat{C}_{IJ}(f) =\frac{2}{\tau } \sum _t \tilde{d}_I(t;f) \tilde{d}_J^*(t; f) \frac{\mathcal{N}_{IJ}(f)\gamma _{IJ}(t; f,\hat{n}_0)}{P_{n_I}(t;f) P_{n_J}(t; f)}, \end{aligned}$$

where

8.117 $$\begin{aligned} \mathcal{N}_{IJ}(f) \equiv \left[ \sum _t \frac{\gamma ^2_{IJ}(t; f,\hat{n}_0)}{P_{n_I}(t;f) P_{n_J}(t; f)}\right] ^{-1}. \end{aligned}$$

The search is narrow-band in the sense that one doesn’t integrate over the whole frequency band of the detectors, but looks instead for evidence of a gravitational wave in narrow frequency bins that span the sensitive band of the detector. The weighted cross-correlations are summed over time, to build up signal-to-noise ratio, since the source is assumed to be persistent. The frequentist detection statistic is the squared signal-to-noise ratio of the cross-correlated power contained in each narrow frequency band:

8.118 $$\begin{aligned} \Lambda (d) = \frac{|\hat{C}_{IJ}(f)|^2}{\mathrm{Var}[\hat{C}_{IJ}(f)]} \approx \frac{|\hat{C}_{IJ}(f)|^2}{\mathcal{N}_{IJ}(f)}, \end{aligned}$$

where we used the result that the variance of the cross-correlation estimator $\hat{C}_{IJ}(f)$ equals the normalization factor $\mathcal{N}_{IJ}(f)$ in the weak-signal limit. This modified radiometer search is *robust* in the sense that it makes minimal assumptions about the source. The detection efficiency of the search could be improved if one had additional information about the signal (e.g., if one knew that the radiation was circularly polarized), which could then be included in the stochastic signal model.

## Real-world complications

Experience with real-world data, however, soon convinces one that both stationarity and Gaussianity are fairy tales invented for the amusement of undergraduates. *D.J. Thompson* (Thomson 1994)

The analyses described in the previous sections assumed that the instrument noise is stationary, Gaussian distributed, and uncorrelated between detectors. The analyses also implicitly assumed that the data were regularly sampled and devoid of gaps, facilitating an easy transition between the recorded time series and the frequency domain where many of the analyses are performed. In practice, all of these assumptions are violated to varying degrees, and the analyses of real data require additional care. Analyses that assume stationary, Gaussian noise can produced biased results when applied to more complicated real-world data sets.

### Observatory-specific challenges

To begin the discussion, we highlight some of the challenges associated with real-world data, which are specific to the different observational domains—e.g., ground-based detectors, space-based detectors, and pulsar timing. Then, in the following subsections, we discuss the complications in more detail, and suggest ways to deal with or mitigate these problems.

#### Ground-based interferometers

Analysis of data from the first and second generation ground-based interferometers have shown that the data are neither perfectly stationary nor Gaussian. The non-stationarity can be broadly categorized as having two components: slow, adiabatic drifts in the noise spectrum with time; and short-duration noise transients, referred to as *glitches* (Blackburn et al. 2008), which have compact support in time-frequency. These glitches are also the dominant cause of non-Gaussianity in the noise distributions, giving rise to long “tails” (large amplitude events with non-negligible probability), which extend past a core distribution that is well described as Gaussian. The data are evenly sampled by design, though there are often large gaps between data segments due to “loss of lock” (the interferometer being knocked out of data-taking mode due to an environmental disturbance or instrumental malfunction), scheduled maintenance, etc.

An analysis of LIGO-Virgo data that assumes the noise spectrum is constant over days or weeks would produce biased results. In practice, the data is analyzed using ${\sim } 1$ min-long segments. Glitches, on the other hand, do not pose a significant problem for stochastic searches as they are rarely coherent between detectors. Glitches are a more serious problem for searches that target short duration, deterministic signals.

#### Pulsar timing arrays

Pulsar timing data are, in many ways, far more challenging to analyze (Haasteren and Levin 2010). The lack of dedicated telescope facilities, and the practical constraints associated with making the observations, result in data that are irregularly sampled. Moreover, the very long observation timelines (years to decades) and the mixture of facilities yield data sets that have been collected using a variety of receivers, data recorders, and pulse folding schemes. The heterogeneity of the observations causes the data to be non-stationary. In addition, the characteristic period of the gravitational waves searched for is of order the duration of the observations. Thus, Fourier domain methods for pulsar timing analyses have, at best, limited formal utility.

An additional complication for pulsar timing analyses is that a complicated deterministic timing model that predicts the time of arrival of each pulse has to be subtracted from the data to produce the timing residuals used in the gravitational-wave analyses. The timing model includes a pulsar spindown model and a detailed pulse propagation model that accounts for the relative motion of the Earth and pulsar. Many of the pulsars are in binary systems, so the timing model has to include relativistic orbital motion, and propagation effects such as the Shapiro time delay. Since errors in the timing model are strongly correlated with the gravitational-wave signal, subtracting the timing model unfortunately removes part of the signal as well. Subtraction of the timing model also introduces non-stationarity into the data (Haasteren and Levin 2013), again making time-domain analyses the only possibility (van Haasteren et al. 2009).

#### Space-based detectors

For future space detectors we can only guess at the nature of the noise. Results from the LISA Pathfinder mission provide some insight (Armano et al. 2016), but only for a subset of the detector components, and for somewhat different flight hardware. The data will be regularly sampled, but data gaps are expected due to re-pointing of the communication antennae and orbit adjustments. Possible sources of non-stationarity include variations in the solar wind, thermal variations, and tidal perturbations from the Earth and other solar system bodies. The plans for the first space interferometers envision a single array of 3 spacecraft with 6 laser links. From these links three noise-orthogonal signal channels can be synthesized, but these combinations are also signal orthogonal, and so cross-correlation cannot be used to detect a signal.

### Non-stationary noise

Data from existing gravitational-wave detectors, including bars, interferometers, and pulsar timing, exhibit various degrees of non-stationarity. Here we give examples relevant to ground-based interferometers, but the situation is similar for the other detection techniques.

Non-stationary behavior can manifest itself in many forms, and there are no doubt many factors that contribute to the non-stationarity seen in interferometer data. Nonetheless, a simple two-part model does a good job of capturing the bulk of the non-stationary features. The two-part model consists of a slowly-varying noise spectral density $S_n(t{;}f)$, and localized noise transients or “glitches”. The slow drift in the spectrum can be modeled as a locally-stationary noise process (Dahlhaus 2011), which has the nice feature that for small enough time segments, the data in each segment can be treated as stationary. The glitch contribution to the non-stationarity poses more of a challenge, as the non-stationarity persists even for short data segments.

#### Local stationarity

To illustrate the two-component description of non-stationary data, we begin with a toy model of a locally-stationary red noise process. Later we will add a model for the impulsive, glitch component (Sect. 9.2.2). Consider an auto-regressive $\mathrm{AR}(1)$ process of the form:

9.1 $$\begin{aligned} x_t = q(t) x_{t-1} + \epsilon (t) \delta , \end{aligned}$$

where $\delta \sim N(0,1)$ is a unit-variance Gaussian deviate, and *q*(*t*) and $\epsilon (t)$ are slowly-varying functions of time. The local power spectrum *S*(*t*; *f*) for this process has the form

**Fig. 70:**
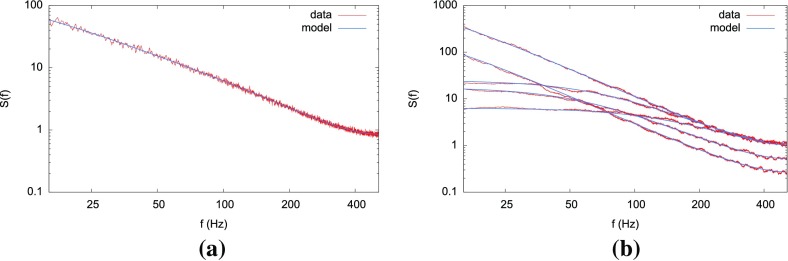
Spectra for the locally-stationary $\mathrm{AR}(1)$ model. Panel **a** shows the smoothed spectrum computed using the full data set compared to the time average of the theoretical spectrum. Panel **b** shows smoothed spectra from the $1\mathrm{st}$, $8\mathrm{th}$, $16\mathrm{th}$, $24\mathrm{th}$ and $32\mathrm{nd}$ time segments compared to the theoretical *S*(*t*; *f*) computed at the central time for each segment

9.2 $$\begin{aligned} S(t;f) = \frac{\epsilon ^2(t)}{1+ q^2(t) - 2 q(t) \cos (\pi f/f_{\mathrm{N}})} , \end{aligned}$$

where $f_{\mathrm{N}}$ is the Nyquist frequency. For a data segment of duration *T* with *N* samples, $f_{\mathrm{N}}=N/(2T)=1/(2 \Delta t)$. Figure 70 shows the average and local spectra for $T=1024$ seconds of data sampled at 1024 Hz with

9.3 $$\begin{aligned} \begin{aligned} q(t) = q_0 \frac{(1+ \alpha \cos (2 \pi t/T))}{(1+\alpha )}, \quad \epsilon (t) = \epsilon _0 (1+t/T), \end{aligned} \end{aligned}$$

and $q_0=0.95$, $\alpha =0.4$, and $\epsilon _0 = 1$. The local spectra are computed using 32-second segments of data that are smoothed and compared to the predicted spectra (9.2). The smoothed average spectrum is computed using the full data set and compared to the theoretical average spectrum

9.4 $$\begin{aligned} S(f) = \frac{1}{T} \int _0^T S(t;f) \, dt . \end{aligned}$$

The high degree of non-stationarity is clearly apparent from the several orders of magnitude variation in the spectra across different segments of data. In LIGO stochastic background analyses, a “delta sigma” cut is used to discard segments of data that exhibit significant non-stationarity. The square-root of the variance (6.33) of the cross-correlation statistic is compared between three consecutive short segments of data (each typically 60 seconds long), and if the levels differ by more than 20%–30% those segments are not used in the analysis (Abbott et al. 2005, 2007).

The degree of non-stationarity can be measured from the auto-correlation of the whitened Fourier coefficients $\bar{x}_f = \tilde{x}_f{/}\sqrt{\hat{S}(f)}$, where $\hat{S}(f)$ is estimated from the smoothed power spectra. The auto-correlation at lag *k* is defined by

9.5 $$\begin{aligned} c(k) \equiv \frac{1}{2N} \sum _{i=1}^N (\bar{x}_i \bar{x}^*_{i+k} + \bar{x}^*_i \bar{x}_{i+k}) . \end{aligned}$$

**Fig. 71:**
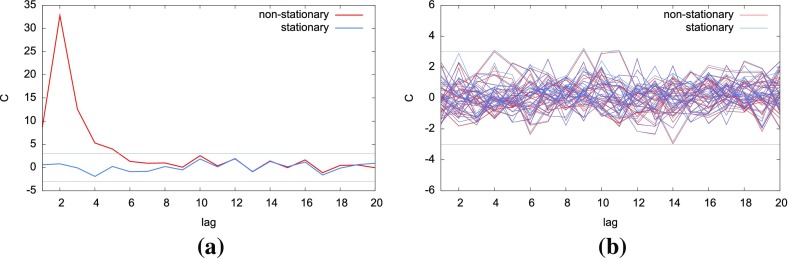
Autocorrelation of the whitened Fourier coefficients as a function of lag for stationary and locally-stationary $\mathrm{AR}(1)$ models. Panel **a** is a comparison for the full data sets, and panel **b** is for each of the 32 sub-segments. The locally-stationary data show clear departures from stationarity in the full data set, but are consistent with stationarity in the shorter sub-segments of data

For stationary, Gaussian noise in the large-*N* limit, *c*(*k*) for $k > 0$ is Gaussian distributed with zero mean and variance $\sigma ^2=1/N$ (Dwivedi and Subba Rao 2009). It is convenient to use the scaled auto-covariance $C(k) \equiv \sqrt{N} c(k)$, which has unit variance for stationary, Gaussian noise. Figure 71 compares *C*(*k*) computed for the locally-stationary $\text {AR}(1)$ model shown in Fig. 70, and a stationary $\text {AR}(1)$ model with $q(t)=q_0$ and $\epsilon (t) = \epsilon _0$. The locally-stationary model shows clear departures from stationarity when the auto-correlation is computed using the full data set (as evidenced by the large autocorrelations for small lags), while the data in each of the 32 sub-segments shows no signs of non-stationarity.

**Fig. 72:**
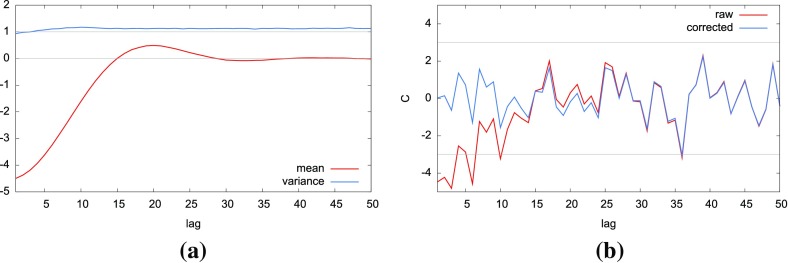
Panel **a** shows the mean and variance of the autocorrelation for stationary, Gaussian noise caused by a Tukey window. Panel **b** shows the raw and corrected autocorrelation for a stationary, Gaussian noise process

One note of caution in using the Fourier autocorrelation *C*(*k*) as an indicator of non-stationarity is that any window that is applied to the time-domain data to lessen spectral leakage in the Fourier transform necessarily makes the data non-stationary. Choosing a window function (Appendix D.2) that is unity across most of the samples, such as a Tukey window (D.14), lessens the taper-induced non-stationarity, but does not eliminate the effect. The solution is to apply a correction to the autocorrelation that accounts for the window. Figure 72 shows the impact that a Tukey window has on the mean and variance of the Fourier autocorrelation *C*(*k*). In this simulation $N= 32768$ samples were used with a Tukey window that is constant across the central 90% of the samples. By subtracting the mean and scaling by the square-root of the variance caused by the Tukey window, the non-stationarity caused by the filter can be corrected for.

#### Glitches

To model the second form of non-stationarity caused by short-duration noise transients, we add Gaussian-enveloped noise bursts to stationary $\text {AR}(1)$ data. The bursts are simulated by generating white noise in the time domain, that is then multiplied by a Gaussian window centered at time $t_0$ with width $\sigma _t$. The data is then Fourier transformed, and the Fourier coefficients are multiplied by a Gaussian window centered at $f_0$ with width $\sigma _f$. In the simulation, the central times were drawn from a Poisson process with a rate of $0.5\, \mathrm{Hz}$, and the central frequencies were drawn from a uniform distribution $U[0,f_{\mathrm{N}}]$. The duration and bandwidth were also drawn from uniform distributions: $\sigma _t \sim U[0.01\,\mathrm{s},0.05\,\mathrm{s}]$, $\sigma _f \sim U[2\,\mathrm{Hz}, 50\,\mathrm{Hz}]$. The signal-to-noise ratio of the bursts was drawn from the distribution

9.6 $$\begin{aligned} p(\mathrm{SNR}) = \frac{ \mathrm{SNR}}{ 2\, \mathrm{SNR}_*^2 \left( 1+ \frac{\mathrm{SNR}}{2\, \mathrm{SNR}_*}\right) ^3} . \end{aligned}$$

**Fig. 73:**
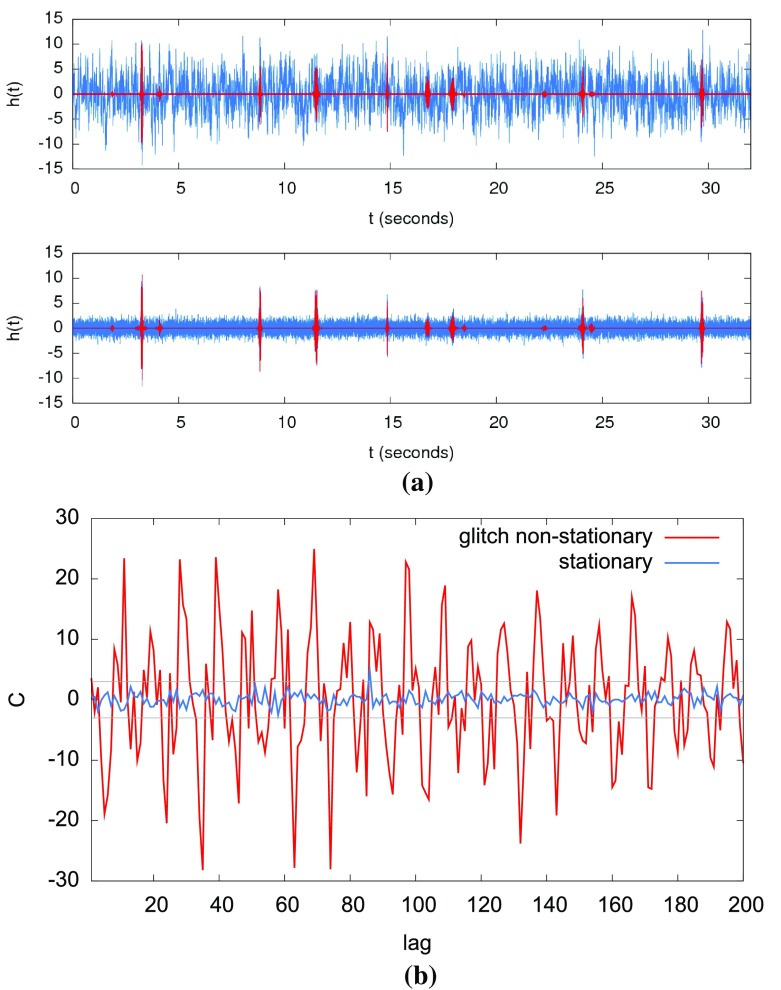
Panel **a** shows simulated stationary $\text {AR}(1)$ data with non-stationary noise transients, or glitches, highlighted in *red*. The *upper panel* is the raw data, while the *lower panel* has been whitened by the estimated amplitude spectral density. Panel **b** shows the autocorrelation for the stationary $\text {AR}(1)$ data without glitches in *blue*, and with glitches in *red*

This form for the $\mathrm{SNR}$ distribution is used by the BayesWave algorithm (Cornish and Littenberg 2015) as a prior on the amplitude of glitches. The truncated power-law form for $p(\mathrm{SNR})$ is motivated by the distribution of glitches seen in real data. Figure 73 shows a 32-second segment of simulated data, and the dramatic effect that the glitches have on the autocorrelation of the Fourier transform. Unlike the locally-stationary noise process, which only introduced correlations for small lags, the glitches produce a much larger deviation from stationarity that extends to large lags.

### Non-Gaussian noise

Gaussian noise processes are ubiquitous in nature, and provide a remarkably good model for the data seen in gravitational-wave detectors. Properly whitened gravitational-wave data typically have a Gaussian core that accounts for the bulk of the samples, along with a small number of outliers in the tails of the distribution. Even these small departures can severely impact analyses that assume perfectly Gaussian distributions.

Gauss developed the *least-squares* (maximum-likelihood) data analysis technique in an effort to determine the orbit of the newly discovered dwarf planet Ceres. Gauss showed that if measurement errors are: (i) more likely small than large, (ii) symmetric, and (iii) have zero mean, then they follow a normal distribution (first described by de Moivre in 1733). Gauss’ proof relied on the law of large numbers: he assumed that under repeated measurements the most-likely value of a quantity is given by the mean of the measured values. The assumptions used in Gauss’ derivation were placed on a firmer footing by Laplace, who derived the central-limit theorem, which states that the arithmetic mean of a sufficiently large number of independent random deviates will be approximately normally distributed, regardless of the underlying distributions the deviates are drawn from, so long as the distributions have finite first and second moments. The central limit theorem is often invoked to explain the ubiquity of Gaussian measurement errors. While the classic central limit theorem applies to noise contributions that are fundamentally stochastic (such as those with a quantum origin), a variant of the central limit theorem also applies to the sum of a large number of *deterministic* effects, so long as the deterministic processes obey certain conditions (Imkeller and von Storch 2001).

**Fig. 74:**
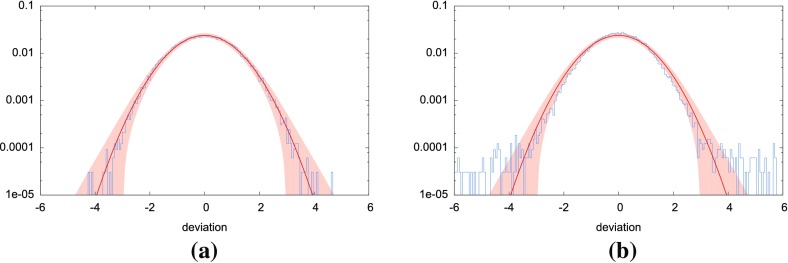
Histograms of the whitened data samples for the simulated data shown in Fig. 73. A reference *N*(0, 1) Gaussian distribution is shown as a *red line*. The *light red band* denotes the 3-sigma confidence interval for the finite number of samples used to produce the histograms. Panel **a** uses the whitened Fourier coefficients, while panel **b** uses the whitened time-domain samples. While the non-Gaussianity is most apparent in the time domain, both distributions fail the Anderson–Darling test for Gaussianity

Since gravitational-wave data typically have highly-colored spectra, one cannot simply compare the distribution of samples in time or frequency to a Gaussian distribution. The data first have to be whitened. This can be done by dividing the Fourier coefficients by the square-root of an estimate of the power spectra, and inverse Fourier transforming the result to arrive at a whitened time series. Figure 74 shows histograms of the whitened Fourier-domain and time-domain samples for the simulated data shown in Fig. 73. By eye, the frequency-domain samples appear fairly Gaussian, while the time-domain samples show clear departures from Gaussianity. Applying the Anderson–Darling test (Anderson and Darling 1954) to both sets of samples indicates that the Gaussian hypothesis is rejected in both cases, with a *p*-value of $p=2.6\times 10^{-5}$ for the Fourier-domain samples and $p<10^{-20}$ for the time-domain samples. Applying the same analysis to the locally-stationary $\text {AR}(1)$ model generated using 32 seconds of data (i.e., setting $T=32$ s in the model for *q*(*t*) and $\epsilon (t)$), we find that the whitened Fourier coefficients generally pass the Anderson-Darling test, while the whitened time-domain samples do not. Overall, glitches cause much larger departures from Gaussianity than adiabatic variation in the noise levels.

To-date, there have been no detailed studies of the effects of non-stationary and non-Gaussian noise on stochastic background analyses beyond the theoretical investigations in Allen et al. (2002), Allen et al. (2003) and Himemoto et al. (2007). However, a variety of checks have been applied to the LIGO-Virgo analyses using time-shifted data and hardware and software signal injections, and the results were found to be consistent with the performance expected for stationary, Gaussian noise (Abbott et al. 2005, 2007). In particular, the distribution of the residuals of the cross-correlation detection statistic, formed by subtracting the mean and scaling by the square root of the variance, have been shown to be Gaussian distributed (Abbott et al. 2007).

### Gaps and irregular sampling

Data gaps and irregular sampling do not significantly impact the analyses of interferometer data, but pose a major challenge to pulsar timing analyses.

#### Interferometer data

Interferometer data are regularly sampled, and gaps in the data pose no great challenge since the non-stationarity already demands that the analysis be performed on short segments of coincident data. The main difficulty working with short segments of data is accounting for the filters that need to be applied to suppress spectral leakage (Abbott et al. 2005; Lazzarini and Romano 2004).

#### Pulsar timing data

The collection of pulsar timing data is constrained by telescope, funding, and personnel availability. A large number of pulsars are now observed fairly regularly, with observations occurring every 2–3 weeks. Older data sets are less regularly sampled, and often have gaps of months or even years (Arzoumanian et al. 2015). Moreover, the sensitivity of the instruments varies significantly over time, making the data highly non-stationary, thus obviating the benefit of performing the analyses in the frequency domain. For these reasons, modern pulsar timing analyses are conducted directly in the time domain (van Haasteren et al. 2009).

Noise modeling for pulsar timing has become increasingly more sophisticated (Lentati et al. 2014; Arzoumanian et al. 2015), but in broad strokes, the two main terms in the noise model are: (i) measurement errors $\sigma _i$ in each time-of-arrival measurement, which are assumed to be uncorrelated between time samples *i* and *j*, and (ii) a stationary red noise component $S_{ij}$ that depends on the lag $\vert i - j\vert$ (van Haasteren et al. 2009). These contribute to the time-domain noise correlation matrix $C_n$, which appears in the Gaussian likelihood (3.51):

9.7 $$\begin{aligned} (C_{n})_{ij} = \sigma _i^2 \delta _{ij} + S_{ij} . \end{aligned}$$

The data gaps and irregular sampling imply that the time lags $\vert i - j\vert$ take on a wide range of values, and do not come in multiples of a fixed sample rate $\Delta t$. Inverting the large noise matrix $C_n$ to compute the likelihood can be very expensive unless clever tricks are used (Haasteren and Vallisneri 2014, 2015).

### Advanced noise modeling

The traditional approach to noise modeling has been to assume a simple model, such as the noise being stationary and Gaussian, and then measure the consequences this has on the analyses using Monte Carlo studies of time-shifted data and simulated signals. An alternative approach is to develop more flexible noise models that can account for various types of non-stationarity and non-Gaussianity.

**Fig. 75:**
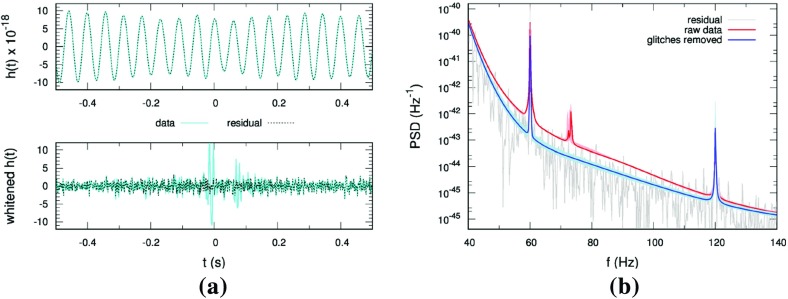
Panel **a** shows a 1-second sample of LIGO S6 data. The *upper plot* shows the raw data and the *lower plot* shows the data whitened by the median *BayesLine* spectra with glitch subtraction by the *BayesWave* algorithm. The *solid aqua line* is the data before glitch removal, and the *dotted black line* is after glitch removal. Panel **b** shows the median and 90% credible bands for the spectral model with (*blue*) and without (*red*) glitch subtraction. The *grey line* shows the power spectra of the data after glitch removal. Images provided by Tyson Littenberg

**Fig. 76:**
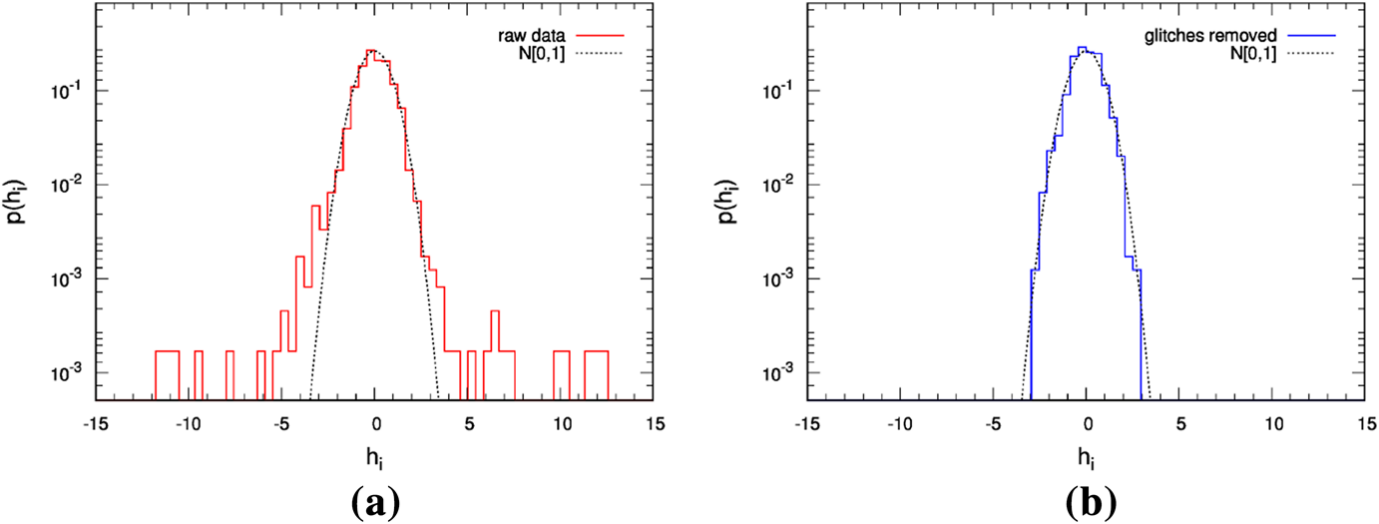
Histograms of the whitened time-domain data shown in Fig. 75. Panel **a** is without glitch subtraction, while panel **b** is with glitch subtraction. Images provided by Tyson Littenberg

One such approach is the *BayesWave/BayesLine* algorithm, which uses a two-part noise model composed of a stationary, Gaussian component *S*(*f*), and short duration glitches, *g*(*t*), modeled as Gaussian-enveloped sinusoids (Cornish and Littenberg 2015; Littenberg and Cornish 2015). The spectral model *S*(*f*) is based on a cubic-spline with a variable number of control points to model the smoothly-varying part of the spectrum, and a collection of truncated Lorentzians to model sharp line features. The optimal number and placement of the control points and Lorentizians is determined from the data using a trans-dimensional Markov Chain Monte Carlo technique. The same technique is used to determine the number of sine-Gaussian glitches and their parameters (central time and frequency, duration, etc.). This approach has been applied to both LIGO data (Cornish and Littenberg 2015; Littenberg and Cornish 2015) and pulsar timing data (Ellis and Cornish 2016). Figure 75 demonstrates the application of the *BayesWave* and *BayesLine* algorithms to data from the LIGO Hanford detector during the S6 science run of the initial LIGO detectors. Removing the glitches has a significant impact on the inferred power spectra. Figure 76 displays histograms of the whitened Fourier coefficients for the data shown in Fig. 75 with and without glitch removal.

Additional models for non-stationary and non-Gaussian noise have been considered by several authors. The detection of deterministic and stochastic signals was considered in Allen et al. (2002), Allen et al. (2003) and Himemoto et al. (2007) for a variety of non-Gaussian noise models, including exponential and two-component Gaussian models. The two-component Gaussian model combined with a non-stationary glitch model was studied in Littenberg and Cornish (2010). Student’s *t*-distribution was considered in Rover (2011). A non-stationary and non-Gaussian noise model was derived in Principe and Pinto (2008) based on a Poisson distribution of sine-Gaussian glitches.

### Correlated noise

The standard cross-correlation statistic for detecting stochastic backgrounds relies on the noise in each detector being uncorrelated. If we return to the simple model for colocated and coaligned detectors, with white Gaussian noise and a white Gaussian signal (Sect. 4.3.1), but now introduce a correlated noise component $S_{n_{12}}$, then the correlation matrix for the signal-plus-noise model becomes

9.8 $$\begin{aligned} C = \left[ \begin{array}{c@{\quad }c} (S_{n_1} +S_h)\,\mathbbm {1}_{N\times N} &{} (S_h + S_{n_{12}})\,\mathbbm {1}_{N\times N} \\ (S_h+S_{n_{12}}) \,\mathbbm {1}_{N\times N} &{} (S_{n_2} +S_h)\,\mathbbm {1}_{N\times N} \\ \end{array} \right] , \end{aligned}$$

yielding the maximum likelihood solution

9.9 $$\begin{aligned} \begin{aligned}&\hat{S}_h \equiv \frac{1}{N}\sum _{i=1}^N d_{1i} d_{2i} - S_{n_{12}} , \\&\hat{S}_{n_1} \equiv \frac{1}{N}\sum _{i=1}^N d_{1i}^2 - \hat{S}_h, \\&\hat{S}_{n_2} \equiv \frac{1}{N}\sum _{i=1}^N d_{2i}^2 - \hat{S}_h. \end{aligned} \end{aligned}$$

We see that there is a degeneracy between the estimate for the signal $\hat{S}_h$ and the correlated noise $S_{n_{12}}$, with no way to separate the two components. Correlated noise with the same spectrum as the signal presents a *fundamental* limit to the detection of stochastic signals.

If the spectral shape of either, or preferably both, the signal and the correlated noise are known, then it is possible to separate the contributions using techniques similar to those that are used to separate the primordial cosmic-microwave-background signal from foreground contamination (Bennett et al. 2003). When the cause of the correlated noise is not fully understood, or when searching for signals with arbitrary spectral shapes, spectrum-based component separation will not be possible.

Several sources of correlated noise have been hypothesized, and in some cases observed, for both interferometer and pulsar timing analyses. Some of the correlations are due to the electronics (Abbott et al. 2005), such as correlations between harmonics of the 60 Hz AC power lines between the LIGO Hanford and LIGO Livingston detectors, and correlations at multiples of 16 Hz from the data sampling referenced to clocks on the Global Positioning System satellites. These narrow-band correlations are easily removed using notch filters. Correlations in the global time standard can also impact pulsar timing observations, as can errors in the ephemeris used in the timing model.

#### Schumann resonances

**Fig. 77:**
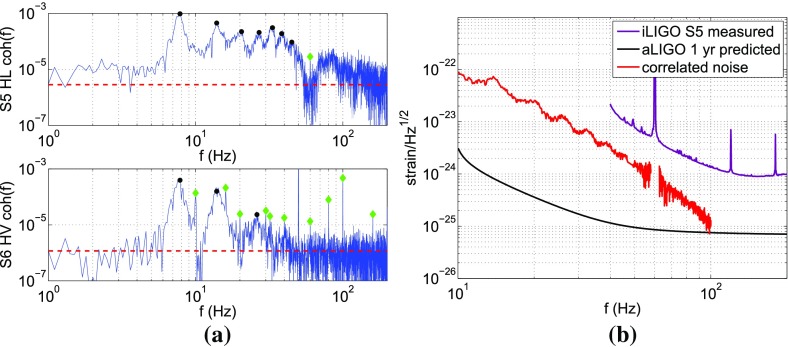
Panel **a** shows the cross-correlation of magnetometer readings between the LIGO-Hanford and LIGO-Livingston sites (HL), and also the LIGO-Hanford and Virgo sites (HV). The peaks indicated by *black dots* are due to Schumann resonances. The *green dots* mark peaks that are due to correlations caused by the electronics. Panel **b** shows the amplitude spectra of the initial and advanced LIGO detectors compared to the estimated level of the correlated noise due to Schumann resonances. The correlated noise level in advanced LIGO should be lower due to differences in the design (notably the lack of magnets attached to the mirrors). Images reproduced with permission from Thrane et al. (2013), copyright by APS

One possible broad-band source of correlated noise for ground-based interferometers that has received considerable attention (Thrane et al. 2013, 2014; Coughlin et al. 2016) are Schumann resonances in the Earth’s magnetic field caused by lightning strikes. These resonances can produce coherent oscillations over thousands of kilometers, and have been observed to produced correlations in magnetometer readings at the LIGO and Virgo sites (Thrane et al. 2013), as shown in panel (a) of Fig. 77. The spectrum of the correlations induced in the detector output depend on both the spectrum of the time-varying magnetic field, and the couplings to the instrument. The estimated spectrum of correlated noise in the initial LIGO detectors from Schumann resonances is shown in panel (b) of Fig. 77. The estimated spectrum lies below the initial LIGO noise curve, but above the design noise curve for the advanced instruments. The situation is not as dire as it looks, however, since the advanced LIGO detectors have a different design that should have weaker coupling to magnetic fields. Nonetheless, Schumann resonances may end up being a limiting factor for advanced LIGO stochastic searches, and efforts are underway to model and subtract their effects (Coughlin et al. 2016). Correlated noise is a much larger problem for colocated detectors, such as the 2 km and 4 km initial LIGO detectors that shared the Hanford site. There it was found that correlated noise prevented the data at frequencies below 460 Hz from being used for stochastic background searches (Aasi et al. 2015).

Perhaps the greatest challenge comes from correlated noise sources of unknown origin. Such noise sources may be well below the auto-correlated noise level in each detector, and thus very hard to detect outside of the cross-correlation analysis. One way of separating these noise sources from a stochastic signal is to build a large number of interferometers at many locations around the world. Each pair of detectors will then have a unique overlap function for gravitational-wave signals that will differ from the spatial correlation pattern of the noise (unless we are incredibly unlucky!). In principle, the difference in the frequency-dependent spatial correlation patterns of the signal and the noise will allow the two components to be separated.

### What can one do with a single detector (e.g., LISA)?

The discovery of the cosmic microwave background was described in a paper with the unassuming title “A Measurement of Excess Antenna Temperature at 4080 Mc/s” (Penzias and Wilson 1965). Penzias and Wilson used a single microwave horn, and announced the result after convincing themselves that no instrumental noise sources, including pigeon droppings, could be responsible for the excess noise seen in the data. In principle, the same approach could be used to detect a stochastic gravitational-wave signal using a single instrument.

Single-detector detection techniques will be put to the test when the first space-based gravitational-wave interferometer is launched, since (unless the funding landscape changes dramatically) the instrument will be a single array of 3 spacecraft. Assuming that pairs of laser links operate between each pair of spacecraft, it will be possible to synthesize multiple interferometry signals from the phase readouts (Estabrook et al. 2000). One particular combination of the phase readouts, called the *T* channel, corresponds to a Sagnac interferometer, and is relatively insensitive to low-frequency gravitational waves, forming an approximate null channel (see Sect. 4.7 for a discussion of null channels). Other combinations, such as the so-called *A* and *E* channels (Prince et al. 2002), are much more sensitive to gravitational-wave signals. Using the Sagnac *T* to measure the instrument noise, the relative power levels in the $\{A,E,T\}$ channels can be used to separate a stochastic signal from instrument noise (Tinto et al. 2001).

LISA-type observatories operate as synthetic interferometers by forming gravitational-wave observables in post-processing using different combinations of the phasemeter readouts from each inter-spacecraft laser link. The combinations synthesize effective equal-path-length interferometers to cancel the otherwise overwhelming laser frequency noise. These combinations have to account for the unequal and time-varying distances between the spacecraft.

**Fig. 78:**
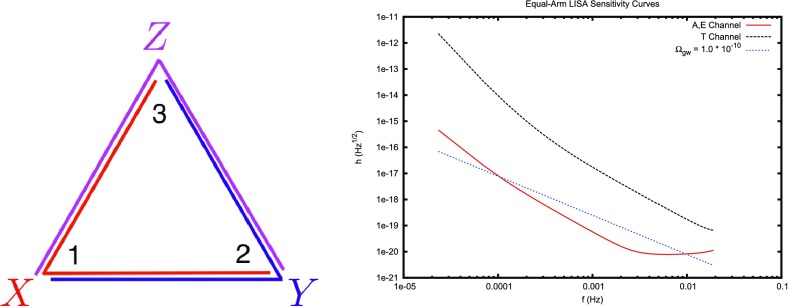
Panel **a** shows the geometry of a LISA-like space interferometer and the laser paths for the synthetic Michelson interferometers *X*, *Y*, *Z*. Panel **b** shows sensitivity curves for the *A*, *E*, *T* interferometry variables compared to a scale-invariant background, $\Omega _\mathrm{gw}(f)=\Omega _0=\mathrm{const}$, with $\Omega _0 = 10^{-10}$. The Sagnac-like *T* channel is far less sensitive than the Michelson-like *A*, *E* channels, and can be used to measure the instrumental noise levels. (Panel **b** is adapted from Adams and Cornish (2010))

In the conceptually simpler equal-arm-length limit, the Michelson-type signal extracted from vertex 1 (see panel (a) of Fig. 78) is given by

9.10 $$\begin{aligned} X(t) = M_1(t) - M_1(t-2L), \end{aligned}$$

where

9.11 $$\begin{aligned} M_1(t) = \Phi _{12}(t-L) + \Phi _{21}(t) - \Phi _{13}(t-L) - \Phi _{31}(t), \end{aligned}$$

and $\Phi _{ij}(t)$ is the readout from the phasemeter on spacecraft *j* that receives light from spacecraft *i*. Permuting the spacecraft labels $\{1,2,3\}$ yields equivalent expressions for the Michelson observables *Y* and *Z*, as shown in panel (a) of Fig. 78. The phasemeter readouts $\Phi _{ij}(t)$ are impacted by acceleration noise $S^a_{ij}$ and position noise $S^p_{ij}$. When the noise levels in each spacecraft are equal, there exist noise-orthogonal combinations (Prince et al. 2002; Adams and Cornish 2010):

9.12 $$\begin{aligned} \begin{aligned} A&\equiv \frac{1}{3} (2X-Y-Z), \\ E&\equiv \frac{1}{\sqrt{3}} (Z-Y), \\ T&\equiv \frac{1}{3} (X+Y+Z). \end{aligned} \end{aligned}$$

Note that these variables are only noise-orthogonal in the symmetric noise limit. For example, the position noise contribution to the cross-spectra $\langle AE \rangle$ is given by

9.13 $$\begin{aligned} \langle AE \rangle = -\frac{4}{3\sqrt{3}}\sin ^2\left( \frac{f}{f_*}\right) \bigg (2\cos \left( \frac{f}{f_*}\right) +1\bigg )\bigg (S^p_{13}-S^p_{12}+S^p_{31}-S^p_{21}\bigg ), \end{aligned}$$

which vanishes when $\{S^p_{13},S^p_{12},S^p_{31},S^p_{21}\}$ are equal, but not otherwise (Adams and Cornish 2010). The synthetic interferometers *A*, *E* are rotated by 45 degrees with respect to each other, and provide instantaneous measurements of the $+$ and $\times$ polarization states. The Sagnac-like *T* channel is relatively insensitive to gravitational waves for frequencies below the transfer frequency $f_*\equiv c/(2\pi L)$. The *T* channel can be used to infer the instrument noise level, so that any excess in the *A*, *E* channels can then be confidently attributed to gravitational waves (Tinto et al. 2001). For frequencies $f \ll f_*$ the $\{A,E,T\}$ channels have uncorrelated responses to unpolarized, isotropic stochastic gravitational-wave signals.

There are some subtleties associated with using the *T* channel as a noise reference as the noise combinations in *T* differ from those in *A*, *E*. For example, the acceleration noise appears in *T* as (Adams and Cornish 2010):

9.14 $$\begin{aligned} \langle TT \rangle = \frac{16}{9}\sin ^2\left( \frac{f}{f_*}\right) \bigg (1-\cos \left( \frac{f}{f_*}\right) \bigg )^2 \bigg (S^a_{12}\,+\,S^a_{13}\,+\,S^a_{31}\,+\,S^a_{32}\,+\,S^a_{23}\,+\,S^a_{21}\bigg ), \end{aligned}$$

while the acceleration noise appears in *A* and *E* as

9.15 $$\begin{aligned} \langle AA \rangle= & {} \frac{16}{9}\sin ^2\left( \frac{f}{f_*}\right) \Bigg \{\cos \left( \frac{f}{f_*}\right) \bigg [4\big (S^a_{12}+S^a_{13}+S^a_{31}+S^a_{21}\big )-2\big (S^a_{23}+S^a_{32}\big )\bigg ] \nonumber \\&+\cos \left( \frac{2f}{f_*}\right) \bigg [\frac{1}{2}(S^a_{12}+S^a_{13}+S^a_{23}+S^a_{32})+2(S^a_{31}+S^a_{21})\bigg ] \nonumber \\&+\frac{9}{2}(S^a_{12}+S^a_{13})+3(S^a_{31}+S^a_{21})+\frac{3}{2}(S^a_{23}+S^a_{32})\Bigg \}, \end{aligned}$$

and

9.16 $$\begin{aligned} \langle EE \rangle= & {} \frac{16}{3}\sin ^2\left( \frac{f}{f_*}\right) \bigg \{ S^a_{23}+S^a_{32}+S^a_{21}+S^a_{31} +2\cos \left( \frac{f}{f_*}\right) \bigg (S^a_{23}+S^a_{32}\bigg ) \nonumber \\&+\cos ^2\left( \frac{f}{f_*}\right) \bigg (S^a_{23}+S^a_{32}+S^a_{12}+S^a_{13}\bigg )\bigg \}. \end{aligned}$$

In the ideal case where the noise levels are the same in each link, *T* provides a measurement of the average noise, which can then be used as an estimator for the noise in *A*, *E*. An analysis that assumes common noise levels will overstate the sensitivity to a signal. A more conservative approach is allow for unequal noise levels and to infer the individual contributions from the data. For example, if one link is particularly noisy, it will dominate the noise in *T*, and enter unequally in *A* and *E*, making it possible to identify the bad link and account for it in the analysis.

Bayesian inference is ideally suited to the task of jointly inferring the signal and noise levels using models that fold in prior knowledge of the signals and instrument components (Adams and Cornish 2010). The separation is aided by the difference in the transfer functions for the signal and the noise. Analytic expressions for the signal transfer or auto-correlated response functions (which are proportional to $\Gamma _{II}$ from Sect. 5.3.4) can be derived in the low-frequency limit $f \ll f_*$:

9.17 $$\begin{aligned} \mathcal{R}_{TT} = 4\sin ^2\left( \frac{f}{f_*}\right) \bigg [\frac{1}{12096} \left( \frac{f}{f_*}\right) ^6 -\frac{61}{4354560}\left( \frac{f}{f_*}\right) ^8 + \cdots \bigg ], \end{aligned}$$

and

9.18 $$\begin{aligned} \mathcal{R}_{AA}= & {} \mathcal{R}_{EE} = 4\sin ^2\left( \frac{f}{f_*}\right) \bigg [\frac{3}{10}-\frac{169}{1680}\left( \frac{f}{f_*}\right) ^2 +\frac{85}{6048}\left( \frac{f}{f_*}\right) ^4 \nonumber \\&- \frac{178273}{159667200}\left( \frac{f}{f_*}\right) ^6 + \frac{19121}{24766560000}\left( \frac{f}{f_*}\right) ^8 + \cdots \bigg ] . \end{aligned}$$

Note that these signal transfer functions are very different from the acceleration noise transfer functions given in (9.14), (9.15), (9.16). The difference in the transfer functions, combined with priors on the functional form of the power spectral density of the noise and signal, allows for the detection of signals that are significantly below the noise level, even when there are not enough links to form the *T* channel (Adams and Cornish 2010). The sensitivity decreases for less informative priors. In the limit that the priors allow for arbitrarily complicated functional forms for the noise and signal spectra—forms so *contrived* that they can compensate for the differences in the transfer functions—it becomes impossible to separate signal from noise. In practice, a combination of pre-flight and on-board testing, combined with physical modeling, will hopefully constrain the noise model sufficiently to inform the analysis and allow for component separation.

An additional complication for space interferometers operating in the mHz frequency range are the millions of astrophysical signals that can drown-out a cosmologically-generated stochastic background. While the brightest signals from massive black hole mergers, stellar captures, and galactic binaries can be identified and subtracted, a large number of weaker overlapping signals will remain, creating a residual *confusion noise*. The largest source of confusion noise is expected to come from millions of compact white-dwarf binaries in our galaxy. The annual modulation of the white-dwarf confusion noise due to the motion of the LISA spacecraft (see Fig. 40) will allow for this component to be separated from an isotropic stochastic background, though at the cost of reduced sensitivity to the background (Adams and Cornish 2014).

## Prospects for detection

It’s tough to make predictions, especially about the future. *Yogi Berra*

The detection of the binary black hole merger signals GW150914 and GW151226 give us confidence that stochastic gravitational waves will be detected in the not-to-distant future. Not only do they show that our basic measurement principles are sound, they also point to the existence of a much larger population of weaker signals from more distant sources that will combine to form a stochastic background that may be detected by 2020 (Abbott et al. 2016h). Indeed, a confusion background from the superposition of weaker signals eventually becomes the limiting noise source for detecting individual systems (Barack and Cutler 2004). As a general rule of thumb, individual bright systems will be detected before the background for transient signals (those that are in-band for a fraction of the observation time), while the reverse is true for long-lived signals, such as the slowly evolving supermassive black-hole binaries targeted by pulsar timing arrays (Rosado et al. 2015). The prospects for detecting more exotic stochastic signals, such as those from phase transitions in the early Universe or inflation, are much less certain, but are worth pursing for their high scientific value. In this section we begin with a brief review of detection sensitivities curves across the gravitational-wave spectrum, followed by a review of the current limits and prospects for detection in each observational window.

### Detection sensitivity curves

Detector sensitivity curves provide a useful visual indicator of the sensitivity of an instrument to potential gravitational-wave sources. A good pedagogical description of the various types of sensitivity curve in common use can be found in Moore et al. (2015a). Here we provide a more condensed summary.

The simplest type of sensitivity curve is a plot of the power spectral density of the detector noise $P_n(f)$, or its amplitude spectral density $\sqrt{P_n(f)}$. (Recall that the mean-squared noise in the band $[f_1,f_2]$ is just the integral of $P_n(f)$ over that band). But plots of $P_n(f)$ or $\sqrt{P_n(f)}$ can be misleading since they do not take into account the frequency-dependent response to gravitational waves seen in Fig. 33. A better quantity to plot is the sky and polarization-averaged amplitude spectral density

10.1 $$\begin{aligned} h_\mathrm{eff}(f) \equiv \sqrt{S_n(f)} = \sqrt{P_n(f)/\mathcal{R}(f)}, \end{aligned}$$

which has units of $\mathrm{strain}/\sqrt{\mathrm{Hz}}$, or the corresponding (dimensionless) characteristic strain noise

**Fig. 79:**
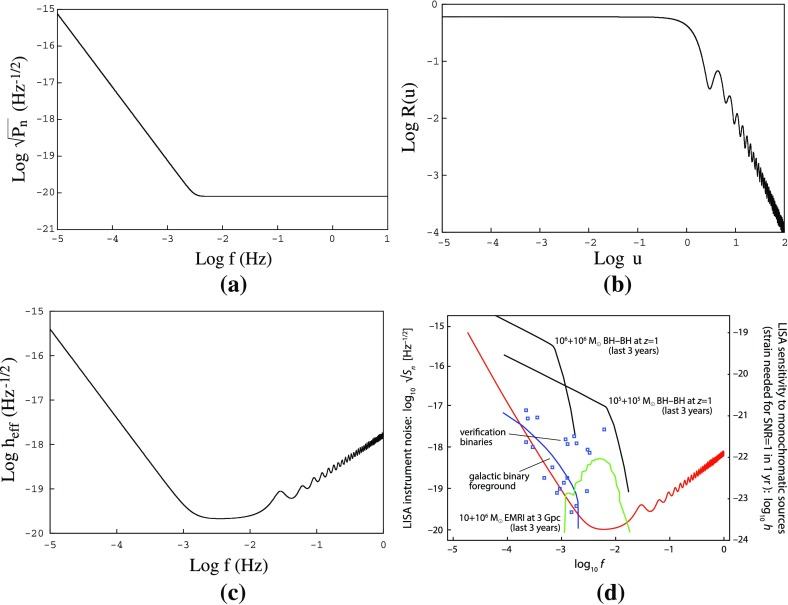
Constructing a sensitivity curve for the LISA detector. Panel **a** shows the amplitude spectral density of the noise. Panel **b** shows the sky and polarization-averaged response function. Panel **c** shows the sensitivity curve found by dividing the noise spectral density by the response function. Panel **d** compares the filtered effective signal strength $\sqrt{2fTS_h(f)}$ for various signals to the LISA sensitivity curve $\sqrt{S_n(f)}$. Panels **a**–**c** Image reproduced with permission from Larson et al. (2000), copyright by APS. Panel **d** Image provided by M. Vallisneri

10.2 $$\begin{aligned} h_n(f) \equiv \sqrt{f S_n(f)}, \end{aligned}$$

where $\mathcal{R}(f) \equiv \Gamma _{II}(f)$ is the transfer function defined in (5.41) and (5.42). Figure 79 shows the construction of a LISA sensitivity curve from $P_n(f)$ and $\mathcal{R}(u)$, where $u=f/f_*$ and $f_*= c/(2 \pi L)$. Note that for LIGO the factor $\mathcal{R}(f)$ is usually not included in sensitivity plots since $f_* \simeq 12 \, \mathrm{kHz}$, and $\mathcal{R}(f)$ is effectively constant across the LIGO band.

The amplitude spectral density sensitivity curve $h_\mathrm{eff}(f)$ has to be interpreted with some care, as simply comparing this curve to the amplitude spectral density of a signal does not immediately convey how detectable the signal is, as the likelihood function and detection statistics derived from the likelihood function involve integrals over frequency. The problem is compounded by the necessity to plot the sensitivity curves on a log–log scale, where “integration-by-eye” misses the increase in the number of frequency bins per logarithmic frequency interval. Rather than plot the raw signals, it is more informative to show quantities that account for the detection techniques being used. For example, the amplitude signal-to-noise ratio $\rho$ for a deterministic signal $\tilde{h}(f)$ is given by

10.3 $$\begin{aligned} \rho ^2 = \int _{f=0}^\infty \frac{4 \vert \tilde{h}(f) \vert ^2}{P_n(f)} \, df = \int _{f=0}^\infty \frac{4 f \vert \tilde{h}(f) \vert ^2}{P_n(f)} \, d(\ln f) . \end{aligned}$$

Averaging over sky location and polarization we have

10.4 $$\begin{aligned} \overline{\rho ^2} = \int _{f=0}^\infty \frac{4 f \tilde{h}_\mathrm{rss}^2(f) \mathcal{R}(f)}{P_n(f)} \,d(\ln f) = \int _{f=0}^\infty \frac{ (2 f T) S_h(f)}{S_n(f)} \, d(\ln f) , \end{aligned}$$

where $\tilde{h}_\mathrm{rss}^2(f) \equiv \vert \tilde{h}_+(f) \vert ^2 + \vert \tilde{h}_\times (f) \vert ^2$, and $S_h(f)$ is the power spectral density of the gravitational-wave signal,

10.5 $$\begin{aligned} S_h(f) \equiv \frac{2 \tilde{h}_\mathrm{rss}^2(f)}{T} . \end{aligned}$$

**Fig. 80:**
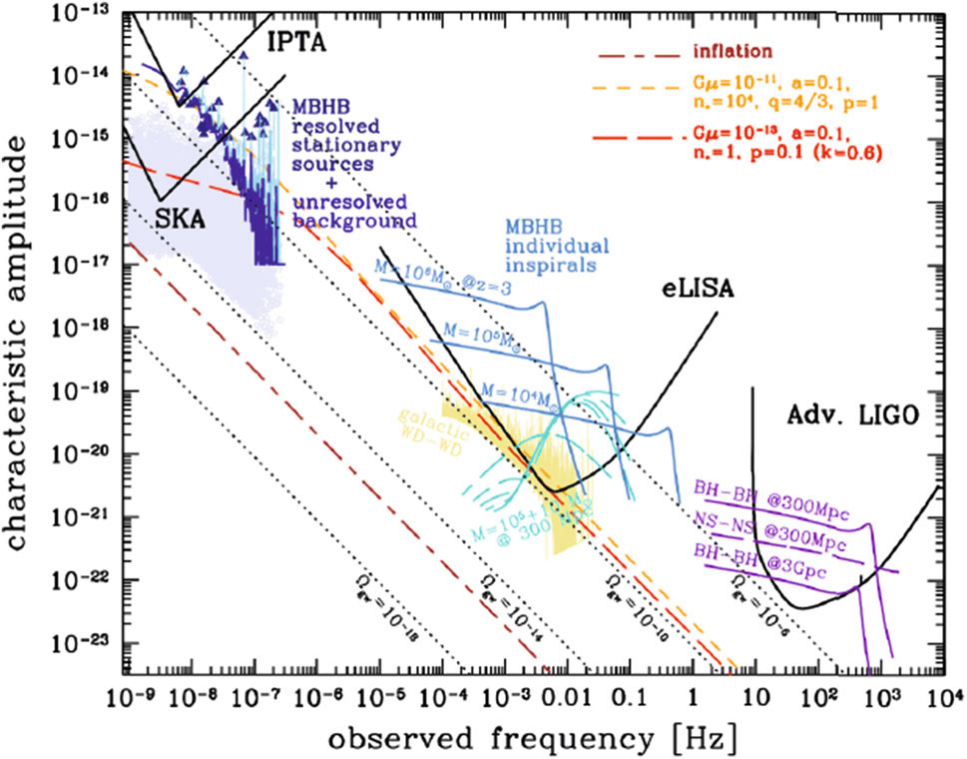
Examples of detector sensitivity curves compared to potential gravitational-wave signals, comparing the characteristic strain signal $h_c(f)$ to the characteristic strain noise $h_n(f)$. Image reproduced with permission from Janssen et al. (2015), copyright by the authors

The quantity $(2 f T) S_h(f)/S_n(f)$ is the contribution to the square of the signal-to-noise ratio per logarithmic frequency interval. The factor of 2*fT* describes the boost that we get by coherently integrating the signal over many cycles. For deterministic signals the amplitude signal-to-noise ratio grows as $T^{1/2}$. Since sensitivity curves are usually plotted in terms of the amplitude spectral density $h_\mathrm{eff}(f) = \sqrt{S_n(f)}$, it is natural to plot signals in terms of the square-root of the numerator of (10.4). Representative LISA sources are represented in this way in panel (d) of Fig. 79. An alternative choice is to plot both of these quantities multiplied by the square-root of the frequency, which yield the characteristic strain for the signal, $h_c(f)$, as well as for the noise, $h_n(f)$. Examples of characteristic strain sensitivity curves are shown in Fig. 80.

For isotropic stochastic signals, the sky location and polarization-averaged signal-to-noise ratio $\rho$ is

10.6 $$\begin{aligned} \rho ^2 = 2T \int _{f=0}^\infty df\>\sum _{I=1}^M\sum _{J>I}^M \frac{\Gamma _{IJ}^2(f) S_h^2(f)}{P_{n_I}(f)P_{n_J}(f)} = \int _{f=0}^\infty \frac{ (2f T) S_h^2(f)}{S^2_\mathrm{net}(f)}\, d(\ln f) , \end{aligned}$$

where

10.7 $$\begin{aligned} S_\mathrm{net}(f)\equiv \left[ \sum _{I=1}^M\sum _{J>I}^M \frac{\Gamma _{IJ}^2(f)}{P_{n_I}(f)P_{n_J}(f)} \right] ^{-1/2}. \end{aligned}$$

Note that for stochastic signals, $\rho$ is a *power* signal-to-noise ratio. Similar to the amplitude signal-to-noise ratio for deterministic signals, the power signal-to-noise ratio for stochastic signals grows as $T^{1/2}$. (This assumes we are in the weak-signal limit, and that the effective low-frequency cutoff does not change with time. See Siemens et al. (2013) for a more complicated scaling that occurs for pulsar timing arrays). Following the same logic as was applied to deterministic signals, it would be natural to plot $(2f T)^{1/4} \sqrt{S_h(f)}$ against sensitivity curves defined by $\sqrt{S_\mathrm{net}(f)}$. Unfortunately, such conventions are not uniformly applied, and the factor of $(2f T)^{1/4}$ is often applied to $\sqrt{S_\mathrm{net}(f)}$ instead:

10.8 $$\begin{aligned} h_\mathrm{eff}(f) \equiv \frac{1}{(2 T f)^{1/4}} \sqrt{S_\mathrm{net}(f)}. \end{aligned}$$

**Fig. 81:**
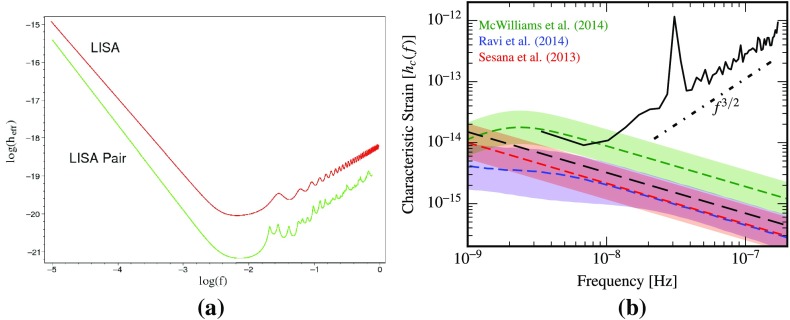
Panel **a** compares $h_\mathrm{eff}(f)$ for an isotropic stochastic background for a single LISA detector to that for a pair of LISA detectors arranged in a crossed-star configuration using an observation time of 1 year. Panel **b** compares the per-frequency-bin $(\Delta f = 1/T)$ upper limits on an isotropic stochastic background derived from the NANOGrav 9-year data set (*solid black line*) to three astrophysical models for the signal from supermassive black hole binaries. The upturn in the bound at low frequencies and the spike at $f=1/\mathrm{year}$ are due to the timing model acting as a filter on the signal. Images reproduced with permission from Cornish (2002), copyright by APS (Panel **a**); and from Arzoumanian et al. (2016), copyright by AAS (Panel **b**)

A plot of $h_\mathrm{eff}(f)$, averaged over a logarithmic frequency interval $\Delta f = f/10$, for a crossed pair of LISA-like detectors is shown in Fig. 81. Also shown in this figure are the related per-frequency-bin upper bounds that are quoted by pulsar timing groups using fixed frequency intervals $\Delta f = 1/T$.

**Fig. 82:**
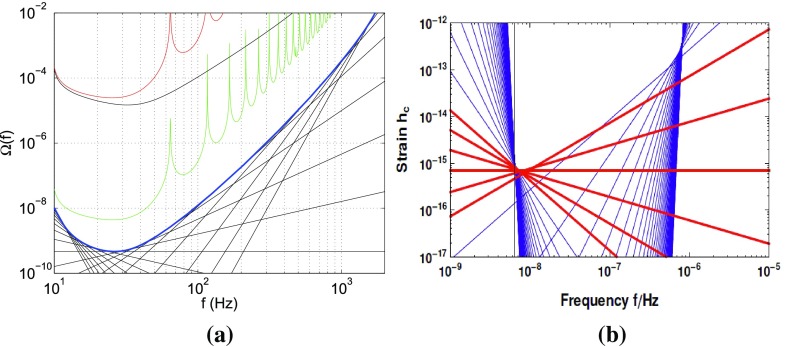
Panel **a** shows the sensitivity of the advanced LIGO Hanford–Livingston detector pair in terms of gravitational-wave energy density $\Omega _\mathrm{gw}(f)$ using a variety of methods. The *blue line* is the sensitivity to isotropic stochastic signals with power-law spectra, formed from the envelope of backgrounds with a wide range of spectral slopes (shown as straight *black lines*). Also shown as a *black curve* is the noise spectral density of a single LIGO detector converted to units of $\Omega (f)$. The *red* and *green lines* are variants of $h_\mathrm{eff}(f)$, again converted to units of $\Omega (f)$. The *lower green curve* is for an observation time of 1 year and $\Delta f = 0.25\,\mathrm{Hz}$. Panel **b** shows the characteristic strain sensitivity for a hypothetical pulsar timing array formed from the envelope of a large number of power law models. The *red lines* show a subset of the power law models used. The upper and lower frequency limits to the sensitivity are set by the observation cadence and the observation time, respectively. Images reproduced with permission from Thrane and Romano (2013), copyright by APS (Panel **a**); and from Moore et al. (2015b), copyright by IOP (Panel **b**)

The most common form of sensitivity curve for stochastic backgrounds compares predictions of the gravitational-wave energy density $\Omega _\mathrm{gw}(f)$ to the equivalent noise energy density $\Omega _n(f) \equiv 2 \pi ^2 f^3 S_n(f) /(3 H_0^2)$. These plots have the advantage of being easy to produce and explain, but they do not fully capture the boost that comes from integrating over frequencies. An alternative form of sensitivity curve that better represents the analysis procedure uses the envelope of limits that can be placed on power-law stochastic backgrounds (Thrane and Romano 2013; Moore et al. 2015b). This method has the advantage of incorporating the integrated nature of the detection statistic. Examples for advanced LIGO and PTAs are shown in Fig. 82.

### Current observational results

#### CMB isotropy

The cosmic microwave background (CMB) provides a snapshot of the Universe ${\approx }400,000$ years after the big bang. During this epoch, the dense, hot plasma that filled the early Universe dilutes and cools to the point where electrons and ions combine to form a neutral gas that is transparent to photons. Maps of the CMB contain a record of the conditions when the CMB photons were last scattered.

Gravitational waves propagating through the early Universe, referred to as tensor perturbations in the CMB literature, can leave an imprint in the temperature and polarization pattern when CMB photons scatter off the tidally-squeezed plasma. The challenge is to separate out the contributions from primordial scalar, vector, and tensor perturbations, and to separate these primordial contributions from subsequent scattering by dust grains and hot gas.

Observations by the *COBE, WMAP* and *Planck* missions, along with a host of ground-based and ballon-borne experiments, have provided strong evidence in support for the inflation paradigm, where the Universe undergoes a short period of extremely rapid expansion driven by some, as yet unknown, *inflaton* field. To keep the discussion brief, we focus our review on the standard single-field “slow-roll” inflation model, and direct the reader to more extensive CMB-focused reviews, e.g., Kamionkowski and Kovetz (2015), that cover more exotic models.

The rapid expansion of some small patch of the very early Universe will erase any initial anisotropy and inhomogeneity, allowing the patch to be modeled by a flat Friedmann–Lemaître–Robertson–Walker (FLRW) metric with scale factor *a*(*t*). The Einstein equations for a FLRW Universe containing an inflaton field $\phi$ with potential $V(\phi )$ are given by^25^

10.9 $$\begin{aligned} H^2 = \left( \frac{\dot{a}}{a}\right) ^2 = \frac{1}{3 M_\mathrm{Pl}^2} \left( \frac{1}{2} \dot{\phi }^2 + V(\phi ) \right) , \end{aligned}$$

and

10.10 $$\begin{aligned} \ddot{\phi }+ 3 H \dot{\phi }+ V_{,\phi } = 0. \end{aligned}$$

In the slow-roll regime, the kinetic energy of the inflaton field $\frac{1}{2} \dot{\phi }^2$ is assumed to be much smaller than the potential energy $V(\phi )$, with $\phi$ having reached “terminal velocity”, such that $\ddot{\phi }\ll H \dot{\phi }$. Thus,

10.11 $$\begin{aligned} 3 H \dot{\phi }\simeq -V_{,\phi } \quad \mathrm{and} \quad H^2 \simeq \frac{V}{3 M_\mathrm{Pl}^2} . \end{aligned}$$

Necessary conditions for these approximations to hold can be expressed in terms of a Taylor-series expansion of the inflaton potential, leading to conditions on the first and second derivatives of the potential:

10.12 $$\begin{aligned} \epsilon _V \equiv \frac{M_\mathrm{Pl}^2 V_{,\phi }^2 }{2 V^2} \ll 1 , \quad \eta _V \equiv \frac{M_\mathrm{Pl}^2 V_{,\phi \phi } }{V} \ll 1 . \end{aligned}$$

The solution of the Einstein equations for slow-roll inflation is well-approximated by an exponentially de Sitter Universe. Quantum fluctuations in the otherwise smooth inflaton field and gravitational field give rise to scalar and tensor perturbations, which leave their imprint in the CMB. On large scales the power spectra for the scalar and tensor fluctuations can be written as

10.13 $$\begin{aligned} P_s(k) = A_s \left( \frac{k}{k_*} \right) ^{n_s(k)-1} \quad \mathrm{and} \quad P_t(k) = A_t \left( \frac{k}{k_*} \right) ^{n_t(k)}, \end{aligned}$$

where the reference wavenumber $k_*=2\pi /\lambda _*$ is typically chosen to correspond to wavelengths $\lambda _* \sim 100 \, \mathrm{Mpc}$. The spectral indices $n_s(k)$ and $n_t(k)$ are usually written in terms of a power-series expansion in $\ln k$:

10.14 $$\begin{aligned} n_s(k) = n_s + \frac{1}{2} \frac{ d n_s}{d \ln k} \ln \left( \frac{k}{k_*} \right) + \frac{1}{6} \frac{ d^2n_s}{d \ln k^2} \ln \left( \frac{k}{k_*} \right) ^2 + \cdots . \end{aligned}$$

The amplitude and spectral indices are related to the energy scale for inflation, *V*, and the slow-roll parameters $\epsilon _V$ and $\eta _V$:

10.15 $$\begin{aligned} A_s \simeq \frac{V}{24 \pi ^2 M_\mathrm{Pl}^4\epsilon _V} \quad \mathrm{and} \quad A_t \simeq \frac{2V}{3 \pi ^2 M_\mathrm{Pl}^4} , \end{aligned}$$

and

10.16 $$\begin{aligned} n_s \simeq 1 + 2 \eta _V - 6\epsilon _V \quad \mathrm{and} \quad n_t \simeq -2 \epsilon _V . \end{aligned}$$

Measuring $A_s$, $A_t$, and $n_s$ fixes the energy scale of inflation, *V*, and the two leading terms in the Taylor-series expansion of the inflaton potential, $V_{,\phi }$ and $V_{,\phi \phi }$. Additionally measuring $n_t$ would provide a consistency check for the slow-roll model.

One challenge in measuring $P_s(k)$ and $P_t(k)$ is that the scalar and tensor perturbations both source temperature and polarization anisotropies in the CMB radiation. Another challenge is that foreground gas and dust can also contribute to the temperature and polarization anisotropies. The various components can be teased apart by observing a wide range of CMB energies across a wide range of angular scales.

The primordial contribution to the CMB follows a black-body spectrum, while the dominant foreground contribution from gas and dust have very different spectra. By observing at multiple CMB wavelengths the primordial and foreground contributions can be separated. Separating the scalar and tensor contributions to the primordial component of the temperature anisotropies can be achieved by making maps that cover a wide range of angular scales, while separating their contributions to the polarization anisotropies can be achieved by decomposing the signal into curl-free *E*-modes and divergence-free *B*-modes, and using measurements made on a wide range of angular scales. For a more in-depth description, see Chapter 27 of the Review of Particle Physics (Olive et al. 2014).

**Fig. 83:**
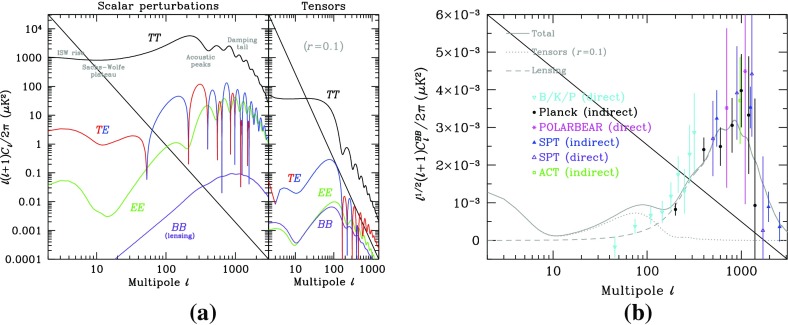
Panel **a** shows the theoretical predictions for the temperature and polarization cross-spectra from scalar and tensor perturbations for the best fit $\Lambda$CDM model from *Planck*, assuming a tensor-to-scalar ratio of $r=0.1$. The *curves* are labeled by type: *TT* labels the temperature power spectrum, while *TE* labels the temperature-*E*-mode cross spectrum and so on. Panel **b** compares recent measurements of the *BB* spectrum to the theoretical prediction. Images reproduced with permission from Olive et al. (2014), copyright by UC Regents

The scalar and tensor contributions to the large-scale temperature anisotropy can be computed using linear perturbation theory. The anisotropy due to tensor fluctuations arises solely from the gravitational potential differences on the last-scattering surface, while the anisotropy due to scalar fluctuations is more complicated, and include contributions from the excitation of sound waves in addition to variations in the gravitational potential. As the co-moving horizon grows, tensor modes that have wavelengths shorter than the horizon size redshift and lose energy. Consequently, the tensor contribution to the CMB anisotropy drops by roughly two orders of magnitude between angular scales $\ell =2$ and $\ell = 200$, while the scalar contribution, after an initial dip, grows until reaching the first acoustic peak at $\ell \simeq 220$. Plots of the predicted scalar and tensor contributions to the temperature (*TT*) power spectra using the best fit $\Lambda$CDM model from *Planck* are shown in panel (a) of Fig. 83. By comparing the CMB anisotropy at very large scales ($\ell \sim 2$–10) and degree scales ($\ell \sim 200$), it is possible to constrain the *tensor-to-scalar ratio* (Knox and Turner 1994):

10.17 $$\begin{aligned} r \equiv \frac{A_t}{A_s}. \end{aligned}$$

In practice, a more sophisticated joint analysis is performed using all available CMB data (often combined with other data sets, such as maps of large-scale structure, weak lensing, and measurements of the expansion history), simultaneously fitting for a large number of cosmological parameters. The *Planck* temperature map, combined with weak lensing data, provide a precise measurement for the amplitude and spectral index of the scalar perturbations:

10.18 $$\begin{aligned} \ln A_s = -19.928 \pm 0.057, \quad n_s = 0.9603 \pm 0.0073, \end{aligned}$$

and a bound on the tensor-to-scalar ratio:

10.19 $$\begin{aligned} r < 0.12 \quad (95\%\ \mathrm{confidence}), \end{aligned}$$

using a pivot scale of $k_* = 0.002\, \mathrm{Mpc}^{-1}$. The *Planck* bound on *r* is the most stringent possible using CMB temperature data (Knox and Turner 1994). (In fact, it beats the theoretical limit slightly since the analysis also used weak lensing and *WMAP* polarization data). In order to improve on this bound, or to detect the tensor contribution, CMB polarization data must also be used.

**Fig. 84:**
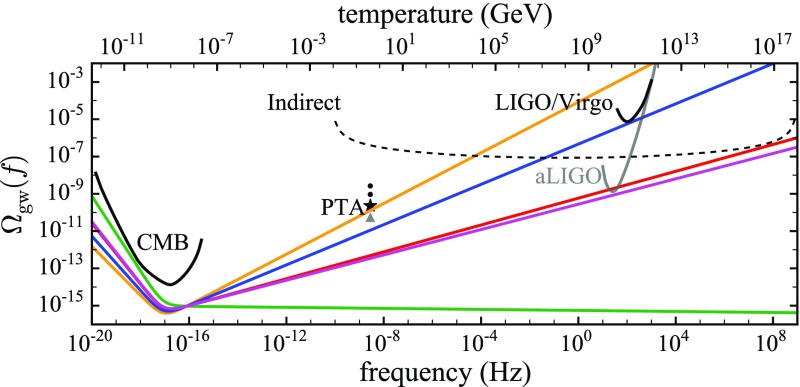
Current and projected bounds on $\Omega _\mathrm{gw}(f)$ from CMB measurements, pulsar timing observations, and ground based interferometers. The curve marked “CMB” shows the projected sensitivity of the *Planck* satellite to primordial *B*-mode polarization anisotropies. The *black star* marked “PTA” is the current 95% upper limit from the Parkes pulsar timing array. The LIGO and aLIGO sensitivity curves were produced using the power-law envelope method (Thrane and Romano 2013). The *curve* labeled “indirect bounds” was produced by converting bounds on the total gravitational-wave energy density from CMB temperature and polarization power spectra, weak lensing, baryon acoustic oscillations, and Big Bang nucleosynthesis to bounds on the energy density per logarithmic frequency interval using power-law models. The colored lines are theoretical predictions for the primordial background assuming $r=0.11$ and for spectral slopes $n_t = 0.68$ (*orange curve*), $n_t= 0.54$ (*blue*), $n_t= 0.36$ (*red*), and $n_t = 0.34$ (*magenta*). The prediction for the simple slow-roll inflation model discussed in this section, $n_t= -r/8$, is shown in *green*. Image reproduced with permission from Lasky et al. (2016), copyright by the authors

The *Planck* bound on *r* can be mapped into constraints on the gravitational-wave energy density via (Turner et al. 1993; Lasky et al. 2016):

10.20 $$\begin{aligned} \Omega _\mathrm{gw}(f) = \frac{3 r A_s \Omega _r}{128} \left( \frac{f}{f_*}\right) ^{n_t} \left[ \frac{1}{2} \left( \frac{f_\mathrm{eq}}{f} \right) ^2 + \frac{16}{9} \right] , \end{aligned}$$

where $f = c k/(2\pi )$, $f_\mathrm{eq} \equiv \sqrt{2} H_0\Omega _m /(2 \pi \sqrt{\Omega _r})$ is the frequency of a horizon-scale mode when matter and radiation have the same density, and $\Omega _m$ and $\Omega _r$ are the matter and radiation density today, in units of the critical density. The projected *Planck* bound from the *B*-mode power spectrum, along with existing and projected bounds from pulsar timing and aLIGO are shown in Fig. 84, which is taken from Lasky et al. (2016). Also shown are curves for theoretical models with a large tensor-to-scalar ratio ($r=0.11$) and a range of spectral tilts $n_t$.

Coherent motion in the primordial plasma can polarize the CMB photons through Thomson scattering. Scalar perturbations source curl-free *E*-mode polarization anisotropies, while the tensor perturbations source divergence-free *B*-mode polarization anisotropies, in addition to *E*-modes. In principle, by decomposing the polarization into *E* and *B* components, and using observations across a range of angular scales, it should be possible to separate the scalar and tensor contributions. In practice, the measurements are extremely challenging due to the weakness of the signals (nano-Kelvin or smaller polarization fluctuations as compared to micro-Kelvin temperature fluctuations) and foreground noise. The main noise contributions come from gravitational lensing, which converts a fraction of the much larger *E*-mode anisotropy into *B*-modes, and scattering by dust grains, which can convert unpolarized CMB radiation into *E* and *B* modes. Both of these potential noise sources have recently been detected (Hanson et al. 2013; Ade et al. 2015c). The detection of *B*-mode polarization on large angular scales by *BICEP2* was originally interpreted as having a primordial origin (Ade et al. 2014), but a joint analysis using *Planck* dust maps (Ade et al. 2015c) showed the signal to be consistent with foreground noise.

While detecting the primordial *B*-mode contribution is very challenging, the pay-off is very large, as measuring the amplitude of the tensor perturbations, $A_t$, fixes the energy scale of inflation, and can be used to strongly constrain models of inflation.

#### Pulsar timing

Pulsar timing observations have made tremendous progress in the past 10 years and are now producing limits that seriously constrain astrophysical models for supermassive black hole mergers. The current observations are most sensitive at $f\sim 10^{-8}\ \mathrm {Hz}$, so we choose a reference frequency of $f_\mathrm{ref} = 10^{-8}\ \mathrm {Hz}$, and quote the latest bounds on $\Omega _\mathrm{gw}(f) = \Omega _\beta (f/f_\mathrm{ref})^\beta$ in terms of bounds on $\Omega _\beta$ for a Hubble constant value of $H_0 = 70~\mathrm{km}\,\mathrm{s}^{-1}\,\mathrm{Mpc}^{-1}$.

For a scale invariant ($n_t=0$) cosmological background, $\beta = 0$. The most recent 95% confidence limits on such a background are (Lentati et al. 2015; Arzoumanian et al. 2016; Shannon et al. 2015; Lasky et al. 2016):

10.21 $$\begin{aligned} \begin{array}{l@{\quad }l} \Omega _0< 1.2 \times 10^{-9} &{}(\mathrm{EPTA}), \\ \Omega _0< 8.5 \times 10^{-10} &{}(\mathrm{NANOGrav}), \\ \Omega _0 < 2.1 \times 10^{-10} &{}(\mathrm{PPTA}). \end{array} \end{aligned}$$

For a stochastic background from a population of black hole binaries on quasi-circular orbits driven by gravitational-wave emission, $\beta =2/3$. The most recent 95% confidence limits on such a background are (Lentati et al. 2015; Arzoumanian et al. 2016; Shannon et al. 2015):

10.22 $$\begin{aligned} \begin{array}{l@{\quad }l} \Omega _{2/3}< 5.4 \times 10^{-9} &{}(\mathrm{EPTA}), \\ \Omega _{2/3}< 1.3 \times 10^{-9} &{}(\mathrm{NANOGrav}), \\ \Omega _{2/3} < 6.0 \times 10^{-10} &{}(\mathrm{PPTA}). \end{array} \end{aligned}$$

#### Spacecraft Doppler tracking

Spacecraft Doppler tracking (Armstrong 2006) operates on the same principles as pulsar timing, with a precision on-board clock and radio telemetry replacing the regular lighthouse-like radio emission of a pulsar. The ${\sim }1$–10 AU Earth-spacecraft separation places spacecraft Doppler tracking between pulsar timing and future LISA-like missions in terms of baseline and gravitational-wave frequency coverage. In principle, a fleet of spacecraft each equipped with accurate clocks and high-power radio transmitters could be used to perform the same type of cross-correlation analysis used in pulsar timing, but to-date the analyses have been limited to single spacecraft studies.

The most stringent bounds come from using the Cassini spacecraft, and place a bound on the strength of a stochastic gravitational-wave background at frequencies of order one over the transit time to the spacecraft (Abbate et al. 2003):

10.23 $$\begin{aligned} \Omega _{\mathrm {gw}}(f)<0.027 \quad \mathrm {for}\quad 10^{-6}< f< 10^{-3}\ \mathrm {Hz} . \end{aligned}$$

#### Interferometer bounds

Data from the initial LIGO and Virgo observation runs, and more recently, from advanced LIGO’s first observing run (O1), have been used to place constraints on the fractional energy density of isotropic stochastic backgrounds across multiple frequency bands between $20-1726$ Hz. The bounds are quoted in terms of $\Omega _\mathrm{gw}(f) =\Omega _\beta (f/f_\mathrm{ref})^\beta$ for $\beta =0$ (flat in energy density), $\beta =3$ (flat in strain spectral density), and $\beta =2/3$ (appropriate for a stochastic signal from a population of inspiralling binaries). The $\beta =0$ bounds are quoted for the lower frequency bands, where the sensitivity is greatest for signals with this slope, while the $\beta =3$ bounds are quoted for the higher frequency bands. The $\beta =2/3$ bound is motivated by the detection of multiple binary black hole mergers during O1, which implies that stellar-remnant black holes may produce a detectable stochastic signal from the superposition of many individually undetected sources (Abbott et al. 2016h). The bounds assume a Hubble constant value of $H_0 = 68~\mathrm{km}\,\mathrm{s}^{-1}\,\mathrm{Mpc}^{-1}$.

*Initial LIGO and Virgo data*

Combining the initial LIGO and Virgo data, the most stringent 95%-confidence upper limits for $\beta =0$ are (Aasi et al. 2014):

10.24 $$\begin{aligned} \begin{array}{l@{\quad }l@{\quad }l} \Omega _{\mathrm {gw}}(f)< 5.6 \times 10^{-6} &{}\mathrm{for}&{}41.5< f< 169.25\ \mathrm {Hz}, \\ \Omega _{\mathrm {gw}}(f)< 1.8 \times 10^{-4} &{}\mathrm{for}&{}170< f< 600\ \mathrm {Hz}. \end{array} \end{aligned}$$

The bounds for $\beta =3$ are (Aasi et al. 2014, 2015):

10.25 $$\begin{aligned} \begin{array}{l@{\quad }l@{\quad }l} \Omega _{\mathrm {gw}}(f)< 7.7 \times 10^{-4}\left( \frac{f}{900 \, \mathrm {Hz}}\right) ^3 &{}\mathrm{for}&{}460< f< 1000\ \mathrm {Hz}, \\ \Omega _{\mathrm {gw}}(f)< 1.0 \times 10^{0}\left( \frac{f}{1300 \, \mathrm {Hz}}\right) ^3 &{}\mathrm{for}&{}1000< f< 1726\ \mathrm {Hz}. \end{array} \end{aligned}$$

We note that the $\beta =3$ bound for the $460< f< 1000\ \mathrm {Hz}$ frequency band comes from a correlation analysis using the colocated 2 km and 4 km Hanford detectors (Aasi et al. 2015).

*Advanced LIGO’s first observing run O1*

The analysis of data from LIGO’s first observing run O1 improves on the above limits for $\beta =0$ and $\beta =3$ at lower frequencies (Abbott et al. 2016b):

10.26 $$\begin{aligned} \begin{array}{l@{\quad }l@{\quad }l} \Omega _{\mathrm {gw}}(f)< 1.7 \times 10^{-7} &{}\mathrm{for}&{}20< f< 85.8\ \mathrm {Hz}, \\ \Omega _{\mathrm {gw}}(f)< 1.7 \times 10^{-8}\left( \frac{f}{25 \, \mathrm {Hz}}\right) ^3 &{}\mathrm{for}&{}20< f< 305\ \mathrm {Hz}. \end{array} \end{aligned}$$

The data was also used to place a limit on stochastic signals with spectral slope $\beta =2/3$, appropriate for stochastic signals from inspiralling binaries (Abbott et al. 2016b):

10.27 $$\begin{aligned} \begin{array}{l@{\quad }l@{\quad }l} \Omega _{\mathrm {gw}}(f)< 1.3 \times 10^{-7}\left( \frac{f}{25 \, \mathrm {Hz}}\right) ^{2/3}&\mathrm{for}&20< f< 98.2\ \mathrm {Hz}. \end{array} \end{aligned}$$

#### Bounds on anisotropic backgrounds

**Fig. 85:**
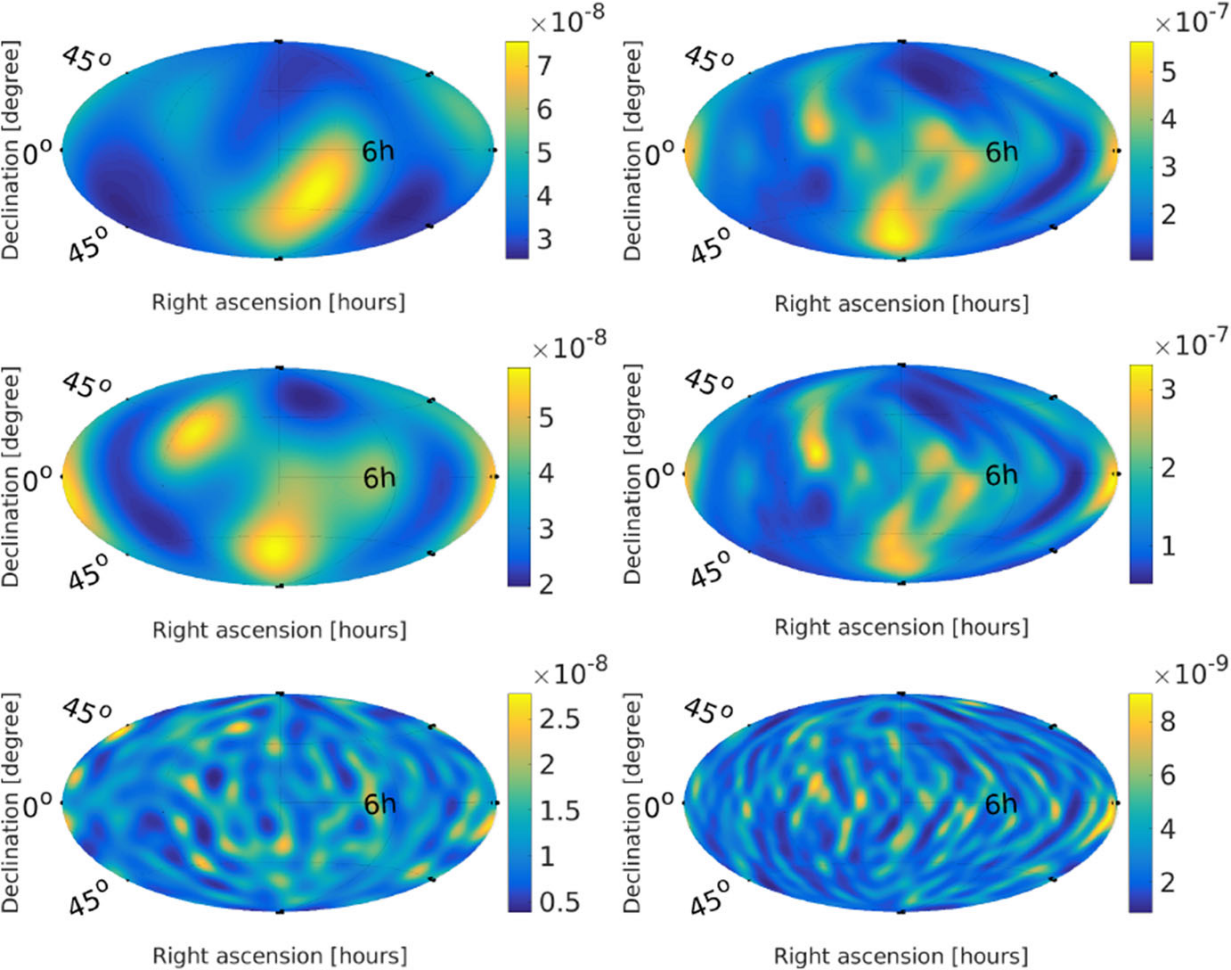
90% confidence-level upper-limit maps on gravitational-wave power for anisotropic backgrounds having spectral indices $\beta = 0$, 2/3, and 3 (*first, second, and third row*, respectively). The data analyzed were from advanced LIGO’s first observational run O1 (Abbott et al. 2016a). *Left column* UL maps on the fractional energy density $\Omega _\beta (\hat{n})$ expressed in units of $\mathrm {sr}^{-1}$, constructed using the spherical harmonic decomposition method out to $l_\mathrm{max}=3$, 4, and 16. *Right column* UL maps on the energy flux $F_{\beta , \hat{n}_0}$ expressed in units of $\mathrm{erg}\,\mathrm{cm}^{-2}\,\mathrm{s}^{-1}\,\mathrm{Hz}^{-1}$, constructed using the radiometer method, which assumes a point-source signal model. Figure adapted from Abbott et al. (2016a)

**Fig. 86:**
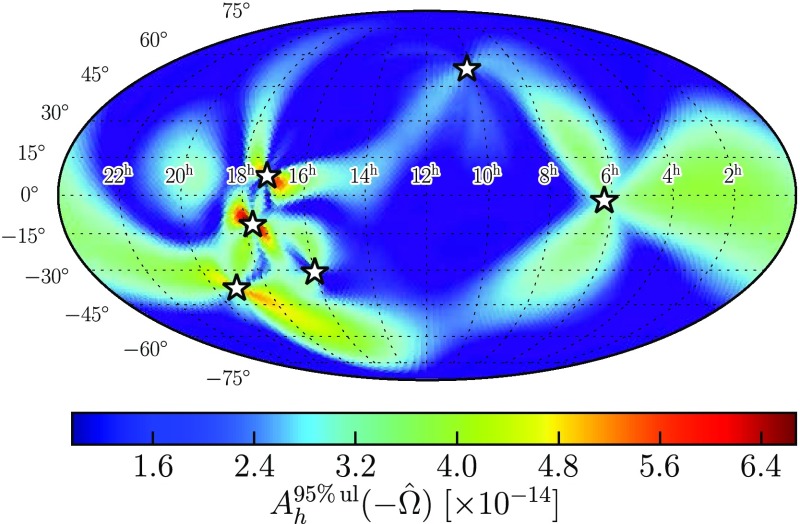
95% confidence-level upper-limit map on the characteristic strain amplitude for an anisotropic background having spectral index $\alpha =-2/3$. The *white stars* show the location of the EPTA pulsars used for the analysis. Image reproduced with permission from Taylor et al. (2015), copyright by APS

Constraints on anisotropic backgrounds have also been set using data from both initial and advanced LIGO (Abadie et al. 2011; Abbott et al. 2016a) and from the European Pulsar Timing Array (Taylor et al. 2015). The corresponding upper-limit maps for advanced LIGO’s first observing run (O1) and from the EPTA data are shown in Figs. 85 and 86, respectively.

The upper-limit maps shown in Fig. 85 are for advanced LIGO’s first observational run (Abbott et al. 2016a). The maps were constructed using both the spherical harmonic decomposition method (left column) and the radiometer method (right column). (These methods are described in Sect. 7.3.6). The three rows correspond to anisotropic backgrounds having spectral indices $\beta =0$, 2/3, and 3, respectively. The spherical harmonic decomposition maps have $l_\mathrm{max}=3$, 4, and 16, respectively, and the upper limits are on

10.28 $$\begin{aligned} \Omega _\beta (\hat{n}) \equiv \frac{2\pi ^2}{3 H_0^2} f_\mathrm{ref}^3\mathcal{P}(\hat{n}), \quad \mathcal{P}(\hat{n}) = \sum _{l=0}^{l_\mathrm{max}} \sum _{m=-l}^l \mathcal{P}_{lm} Y_{lm}(\hat{n}), \end{aligned}$$

expressed in units of fractional energy density per sterardian, $\mathrm{sr}^{-1}$. These limits can be used, for example, to put a constraint on the integrated fractional energy density:

10.29 $$\begin{aligned} \Omega _\mathrm{gw}(f) = \int \! d^2\Omega _{\hat{n}}\> \Omega _{\beta }(\hat{n}) \left( \frac{f}{f_\mathrm{ref}}\right) ^\beta . \end{aligned}$$

The radiometer maps give upper limits on the energy flux

10.30 $$\begin{aligned} F_{\beta , \hat{n}_0} \equiv \frac{c^3 \pi }{4 G}f_\mathrm{ref}^2 \mathcal{P}_{\hat{n}_0}, \quad \mathcal{P}(\hat{n}) = \mathcal{P}_{\hat{n}_0}\,\delta ^2(\hat{n},\hat{n}_0), \end{aligned}$$

expressed in units of $\mathrm{erg}\,\mathrm{cm}^{-2}\,\mathrm{s}^{-1}\,\mathrm{Hz}^{-1}$. Here, *G* is Newton’s gravitational constant, and $\mathcal{P}_{\hat{n}_0}$ is the signal power of a single point source in direction $\hat{n}_0$ (which is the radiometer signal model).^26^ The reference frequency for all the maps is $f_\mathrm{ref}=25~\mathrm{Hz}$, corresponding to the most sensitive part of the frequency band for a stochastic search at advanced LIGO design sensitivity. All the searches include frequencies $20< f < 500~\mathrm{Hz}$, which more than cover the regions of 99% sensitivity for each spectral index.

The upper-limit map shown in Fig. 86 is for the 2015 European Pulsar Timing Array data (Taylor et al. 2015). The map shows the 95% confidence-level upper limits on the (dimensionless) amplitude $A_h$ of the characteristic strain (2.23):

10.31 $$\begin{aligned} h_c(f) = A_h \left( \frac{f}{\mathrm{yr}^{-1}}\right) ^{-2/3}, \end{aligned}$$

for ${\sim } 2< f< 90~\mathrm{nHz}$. The spectral index $\alpha = -2/3$ is appropriate for a stochastic background formed from the superposition of gravitational-wave-driven, circular, inspiraling supermassive black-hole binaries, which is an expected source at the nano-Hz frequencies probed by pulsar timing arrays. The corresponding spectral index for the fractional energy density in gravitational waves, $\Omega _\mathrm{gw}(f)$, is $\beta = 2/3$ (Sect. 2.5).

### Electronic supplementary material

Below is the link to the electronic supplementary material.

Supplementary material 1 (avi 852 KB)

## Figures and Tables

**Fig. 87:**
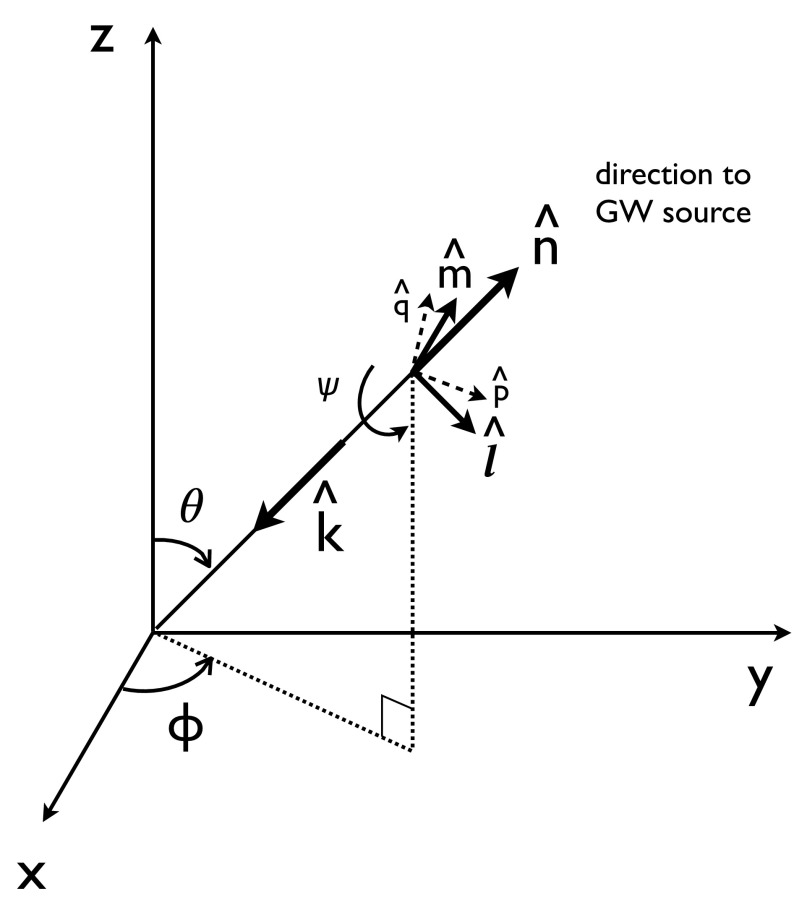
Different choices for the unit vectors perpendicular to $\hat{n}$. By rotating the unit vectors $\hat{l}$, $\hat{m}$ by the angle $\psi$ in the plane orthogonal to $\hat{n}$, one obtains new unit vectors, $\hat{p}$, $\hat{q}$, in terms of which new polarization basis tensors, $\epsilon ^+_{ab}(\hat{n},\psi )$, $\epsilon ^\times _{ab}(\hat{n},\psi )$, are defined

**Fig. 88:**
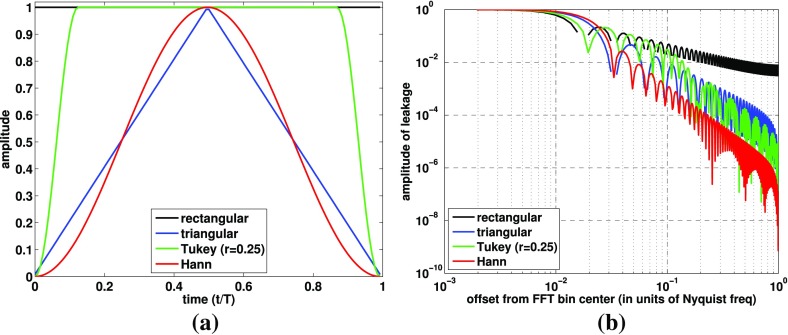
Panel **a** Plots of window functions defined in the main text. Panel **b** Amplitude of spectral leakage for different windows as a function of the offset from the bin center in units of the Nyquist frequency $f_{\mathrm{N}}\equiv 1/(2\Delta t)$. The dips arise because we are using a finite number of samples ($N=128$) to represent the windows

## A Freedom in the choice of polarization basis tensors

### A.1 Linear polarization

In the main text, we chose the $A=+,\times$ polarization basis tensors to be

A.1 $$\begin{aligned} \begin{aligned} e_{ab}^+(\hat{n})&=\hat{l}_a\hat{l}_b - \hat{m}_a\hat{m}_b, \\ e_{ab}^\times (\hat{n})&=\hat{l}_a\hat{m}_b + \hat{m}_a\hat{l}_b, \end{aligned} \end{aligned}$$

where $\hat{n}$ is the direction to the gravitational-wave source, and $\hat{l}$, $\hat{m}$ are unit vectors tangent to the sphere:

A.2 $$\begin{aligned} \begin{aligned} \hat{n}&=\sin \theta \cos \phi \,\hat{x} +\sin \theta \sin \phi \,\hat{y} +\cos \theta \,\hat{z} \equiv \hat{r}, \\ \hat{l}&=\cos \theta \cos \phi \,\hat{x} +\cos \theta \sin \phi \,\hat{y} -\sin \theta \,\hat{z} \equiv \hat{\theta }, \\ \hat{m}&=-\sin \phi \,\hat{x} +\cos \phi \,\hat{y} \equiv \hat{\phi }. \end{aligned} \end{aligned}$$

This particular choice for the vectors $\hat{l}$, $\hat{m}$, perpendicular to $\hat{n}$ is somewhat arbitrary, as one can rotate these vectors by an angle $\psi$ in the plane orthogonal to ${\hat{n}}$, preserving the triple as a right-handed orthonormal triad. (For a gravitational-wave source with a symmetry axis, such as a binary system or rotating neutron star, the angle $\psi$ can be interpreted as the *polarization angle* of the source). See Fig. 87. Under such a rotation, $\hat{l}$ and $\hat{m}$ transform to new unit vectors

A.3 $$\begin{aligned} \begin{aligned}&\hat{p} \equiv \cos \psi \,\hat{l} + \sin \psi \,\hat{m}, \\&\hat{q} \equiv -\sin \psi \,\hat{l} + \cos \psi \,\hat{m}, \end{aligned} \end{aligned}$$

leading to new polarization tensors

A.4 $$\begin{aligned} \begin{aligned} \epsilon _{ab}^+(\hat{n},\psi )&\equiv \hat{p}_a\hat{p}_b - \hat{q}_a\hat{q}_b, \\ \epsilon _{ab}^\times (\hat{n},\psi )&\equiv \hat{p}_a\hat{q}_b + \hat{q}_a\hat{p}_b. \end{aligned} \end{aligned}$$

The new polarization tensors are related to the original ones via

A.5 $$\begin{aligned} \begin{aligned} \epsilon _{ab}^+({\hat{n}},\psi )&=\cos 2\psi \,e_{ab}^+({\hat{n}}) + \sin 2\psi \,e_{ab}^\times ({\hat{n}}), \\ \epsilon _{ab}^\times ({\hat{n}},\psi )&=-\sin 2\psi \,e_{ab}^+({\hat{n}}) + \cos 2\psi \,e_{ab}^\times ({\hat{n}}). \end{aligned} \end{aligned}$$

### A.2 Circular polarization

The form of the above transformation suggests a more convenient basis of polarization tensors. Namely, if we define the complex combinations

A.6 $$\begin{aligned} \begin{aligned} {e}_{ab}^R&\equiv \frac{1}{\sqrt{2}} \left( e_{ab}^+ + i\,e_{ab}^\times \right) , \\ {e}_{ab}^L&\equiv \frac{1}{\sqrt{2}} \left( e_{ab}^+ - i\,e_{ab}^\times \right) , \end{aligned} \end{aligned}$$

or, equivalently,

A.7 $$\begin{aligned} \begin{aligned} {e}_{ab}^R&\equiv \frac{1}{\sqrt{2}}(\hat{l}_a+i\hat{m}_a)(\hat{l}_b+i\hat{m}_b) , \\ {e}_{ab}^L&\equiv \frac{1}{\sqrt{2}}(\hat{l}_a-i\hat{m}_a)(\hat{l}_b-i\hat{m}_b), \end{aligned} \end{aligned}$$

then under the above rotation by $\psi$,

A.8 $$\begin{aligned} \begin{aligned} \epsilon _{ab}^R({\hat{n}},\psi )&=e^{-i 2\psi }\, {e}_{ab}^R({\hat{n}}), \\ \epsilon _{ab}^L({\hat{n}},\psi )&=e^{i 2\psi }\, {e}_{ab}^L({\hat{n}}). \end{aligned} \end{aligned}$$

The tensors $e^R_{ab}$, $e^L_{ab}$ correspond to *right* and *left* circularly polarized waves when looking down on the $\{\hat{l},\hat{m}\}$ plane in the $-\hat{n}$-direction. (The deformation ellipse for $e^R_{ab}$ would rotate to the right, i.e., clockwise, when viewed in this direction). The fact that the right and left circularly polarized waves transform by a simple phase factor involving $2\psi$ is a manifestation of the spin-two nature of the graviton (Weinberg 1972). Indeed, one can show that the scalar field $e^R_{ab}(\hat{n})h^{ab}(f,\hat{n})$ can be written as a linear combination of spin-weight $+2$ spherical harmonics ${}_2Y_{lm}(\hat{n})$, while $e^L_{ab}(\hat{n})h^{ab}(f,\hat{n})$ can be written as a linear combination of spin-weight $-2$ spherical harmonics ${}_{-2}Y_{lm}(\hat{n})$. (See Appendices E, F, G for more details regarding spin-weighted and vector and tensor spherical harmonics).

The Fourier components $h_{ab}(f,\hat{n})$ of the metric perturbations $h_{ab}(t,{\vec {x}})$ can be expanded in terms of either the linear polarization basis tensors:

A.9 $$\begin{aligned} h_{ab}(f,\hat{n}) = h_+(f,\hat{n})e^+_{ab}(\hat{n})+ h_\times (f,\hat{n})e^\times _{ab}(\hat{n}), \end{aligned}$$

or the circular polarization basis tensors:

A.10 $$\begin{aligned} h_{ab}(f,\hat{n}) = h_R(f,\hat{n})e^R_{ab}(\hat{n})+ h_L (f,\hat{n})e^L_{ab}(\hat{n}). \end{aligned}$$

The expansion coefficients $h_R$, $h_L$ are related to $h_+$, $h_\times$ via:

A.11 $$\begin{aligned} \begin{aligned} h_R&= \frac{1}{\sqrt{2}} \left( h_+ - i h_\times \right) , \\ h_L&= \frac{1}{\sqrt{2}} \left( h_+ + i h_\times \right) . \end{aligned} \end{aligned}$$

Note the sign change on the right-hand side of (A.11) compared to (A.6).

### A.3 Polarization matrix and Stokes’ parameters

For a single monochromatic plane wave, the expansion coefficients $h_+$, $h_\times$ or $h_R$, $h_L$ are (complex-valued) constants. The polarization content of the plane wave is encoded in terms of the $2\times 2$ (Hermitian) polarization matrix

A.12 $$\begin{aligned} J_{BB'} \equiv h_B h_{B'}^*, \end{aligned}$$

where *B* labels either the linear polarization components $A\equiv \{+,\times \}$ or circular polarization components $C\equiv \{R,L\}$. For linear polarization, the matrix elements have the form

A.13 $$\begin{aligned} J_{AA'} = \frac{1}{2}\left[ \begin{array}{c@{\quad }c} I+Q &{} U-iV\\ U+iV &{} I-Q\\ \end{array} \right] , \end{aligned}$$

where *I*, *Q*, *U*, *V* are the *Stokes’ parameters* (Jackson 1998):

A.14 $$\begin{aligned} \begin{aligned} I&=|h_+|^2 + |h_\times |^2, \\ Q&=|h_+|^2 - |h_\times |^2, \\ U&=h_+h_\times ^* + h_\times h_+^*, \\ V&=i(h_+ h_\times ^* - h_\times h_+^*). \end{aligned} \end{aligned}$$

For circular polarization, we have

A.15 $$\begin{aligned} J_{CC'} = \frac{1}{2}\left[ \begin{array}{c@{\quad }c} I+V &{} Q-iU\\ Q+iU &{} I-V\\ \end{array} \right] , \end{aligned}$$

where

A.16 $$\begin{aligned} \begin{aligned} I&=|h_R|^2 + |h_L|^2, \\ Q&=h_Rh_L^* + h_L h_R^*, \\ U&=i(h_R h_L^* - h_L h_R^*), \\ V&=|h_R|^2 - |h_L|^2. \end{aligned} \end{aligned}$$

Note that *I* is the total intensity of the wave, *Q* is a measure of linear polarization, $|h_+|^2-|h_\times |^2$, and *V* is a measure of circular polarization, $|h_R|^2 - |h_L|^2$. Since a stochastic gravitational-wave background is a linear *superposition* of plane waves having different frequencies and coming from different directions on the sky, the matrix elements of *J* will be replaced by quadratic *expectation values*, e.g., $\langle h_+(f,\hat{n}) h_\times ^*(f',\hat{n}')\rangle$, which will also depend on whether the background is stationary or anisotropic, etc.

Given the transformation properties (A.8) of $e^R_{ab}$, $e^L_{ab}$, and the definition (A.11) of $h_R$, $h_L$, it follows that $h_R$, $h_L$ transform to

A.17 $$\begin{aligned} \begin{aligned} \bar{h}_R&= e^{i2\psi } h_R, \\ \bar{h}_L&= e^{-i2\psi } h_L, \end{aligned} \end{aligned}$$

under a rotation of the basis vectors $\{\hat{l},\hat{m}\}$ by $\psi$. From these equations and expressions (A.16) for the Stokes parameters, we can further show that *I*, *Q*, *U*, *V* transform to

A.18 $$\begin{aligned} \begin{aligned} \bar{I}&= I, \\ \bar{V}&= V, \\ \bar{Q}+i\bar{U}&= e^{-i4\psi }(Q+iU), \\ \bar{Q}-i\bar{U}&= e^{i4\psi }(Q-iU), \end{aligned} \end{aligned}$$

under a rotation by $\psi$. Thus, *I* and *V* are ordinary scalar (spin 0) functions on the sphere, while $Q\pm iU$ are spin 4 fields, and can be written as linear combinations of spin-weight $\pm 4$ spherical harmonics ${}_{\pm 4}Y_{lm}(\hat{n})$. This has relevance for searches for circularly or linearly polarized stochastic backgrounds, as circular polarization, *V*, is present in the isotropic component of the background, while linear polarization, *Q*, is not (Seto 2008).

## B Some standard results for Gaussian random variables

The statistical properties of a random variable *X* are completely determined by its probability distribution $p_X(x)$. The *moments* of the distribution $\langle X\rangle$, $\langle X^2\rangle$, $\langle X^3\rangle , \ldots$, are defined by

B.1 $$\begin{aligned} \langle X^n\rangle \equiv \int _{-\infty }^\infty dx\, x^n p_X(x). \end{aligned}$$

The first moment $\langle X\rangle$ is the expected (or mean) value of *X*, and is often denoted by $\mu$; the second moment is related to the variance $\sigma ^2$ via the formula $\langle X^2\rangle = \sigma ^2 + \langle X\rangle ^2$. The *characteristic function* of the probability distribution is defined by the Fourier transform:

B.2 $$\begin{aligned} \varphi _X(t)\equiv \int _{-\infty }^\infty dx\,e^{itx}\,p_X(x). \end{aligned}$$

Note that by expanding the exponential

B.3 $$\begin{aligned} \varphi _X(t) = 1 +it\langle X\rangle +\frac{i^2t^2}{2!}\langle X^2\rangle +\cdots . \end{aligned}$$

This means that the moments $\langle X^n\rangle$ can be obtained by simply differentiating $\varphi _X(t)$:

B.4 $$\begin{aligned} \langle X^n\rangle = i^{-n}\left[ \frac{d^n}{dt^n}\varphi _X(t)\right] \bigg |_{t=0}. \end{aligned}$$

If the moments are all finite and the expansion (B.3) is absolutely convergent near the origin, then the probability distribution $p_X(x)$ is simply the inverse Fourier transform of $\varphi _X(t)$:

B.5 $$\begin{aligned} p_X(x) = \frac{1}{2\pi }\int _{-\infty }^\infty dt\,e^{-itx}\,\varphi _X(t). \end{aligned}$$

A similar result can be obtained for a *one-sided* probability distribution $p_X(x)$ (e.g., defined only for $x\ge 0$) by working with Laplace transformations instead.

If *X* is a *Gaussian* random variable, then

B.6 $$\begin{aligned} p_X(x) = \frac{1}{\sqrt{2\pi }\sigma }\, e^{-\frac{1}{2}\frac{(x-\mu )^2}{\sigma ^2}}. \end{aligned}$$

The parameters $\mu$ and $\sigma ^2$ are just the mean and variance of *X*:

B.7 $$\begin{aligned} \mu =\langle X\rangle , \quad \sigma ^2 =\langle X^2\rangle -\langle X\rangle ^2. \end{aligned}$$

A nice property of Gaussian distributions is that all third and higher-order moments can be expressed as a sum of products of the first two moments. For example, for a single Gaussian random variable *X*,

B.8 $$\begin{aligned} \begin{aligned} \langle X^3\rangle&= 3 \langle X\rangle \langle X^2\rangle -2 \langle X\rangle ^3, \\ \langle X^4\rangle&= 4\langle X\rangle \langle X^3\rangle +3\langle X^2\rangle ^2 -12\langle X\rangle ^2 \langle X^2\rangle +6\langle X\rangle ^4, \\&\vdots \end{aligned} \end{aligned}$$

More generally, for $n\ge 3$ these relations can be obtained by solving the equations

B.9 $$\begin{aligned} \left[ \frac{d^n}{dt^n}\ln \varphi _X(t)\right] \bigg |_{t=0} = 0 \end{aligned}$$

for $\langle X^n\rangle$, where $\varphi _X(t)$ is given by the right-hand side of (B.3). The fact that the derivatives are actually equal to zero follows from the specific form for the characteristic function for a Gaussian distribution:

B.10 $$\begin{aligned} \varphi _X(t)=\exp \left[ i\mu t-\frac{\sigma ^2 t^2}{2}\right] . \end{aligned}$$

Since $\ln \varphi _X(t)$ is quadratic in *t*, all third and higher-order derivatives vanish.

A *multivariate Gaussian* distribution is a generalization of (B.6) to a set of random variable $\mathbf {X}\equiv \{X_1, X_2,\ldots , X_N\}$. The joint probability density function is given by

B.11 $$\begin{aligned} p_{\mathbf {X}}(x_1,x_2,\ldots , x_N) = \frac{1}{\sqrt{\det (2\pi C)}}\, e^{-\frac{1}{2}\sum _{i,j} (x_i-\mu _i) \left( C^{-1}\right) _{ij} (x_j-\mu _j)}, \end{aligned}$$

where $\mu _i=\langle X_i\rangle$ are the mean values, and

B.12 $$\begin{aligned} C_{ij}=\langle X_i X_j\rangle - \langle X_i\rangle \langle X_j\rangle \end{aligned}$$

are the elements of the *covariance matrix* *C*. For a zero-mean multivariate Gaussian distribution, all of the odd-ordered moments are identically zero. In addition,

B.13 $$\begin{aligned} \langle X_1 X_2 X_3 X_4\rangle = \langle X_1 X_2\rangle \langle X_3 X_4\rangle + \langle X_1 X_3\rangle \langle X_2 X_4\rangle + \langle X_1 X_4\rangle \langle X_2 X_3\rangle . \end{aligned}$$

We will use several of the above results repeatedly throughout the main text, as most of the probability distributions that we work with are multivariate-Gaussian.

## C Definitions and tests for stationarity and Gaussianity

Here we provide definitions of what it means for data to be stationary and Gaussian, and highlight some tests for these properties. Ascertaining whether or not data are stationary and Gaussian can be challenging as the tests rely on comparison with alternative models, and some models are better at picking up certain forms of non-stationarity and non-Gaussianity than others.

### C.1 Definition of stationarity

A stationary stochastic process has statistical properties that do not depend on time: the joint statistical properties of the sample $\{x_{t_1},\ldots ,x_{t_k}\}$ are identical to the joint statistical properties of the sample $\{x_{t_1+\tau },\ldots ,x_{t_k+\tau }\}$ for all $\tau$ and *k*. In particular, the joint distribution of $(x_t,x_s)$ depends only on the lag $|t-s|$, and not on *t* or *s*, and all higher-order moments are strictly independent of time. A less restrictive, and more practical notion, is that of *weak or second-order stationarity*, which asserts that the mean and variance are constant, and that the auto-covariance $\mathrm{cov}(x_t,x_{t+\tau })$ depends only on the lag $\tau$.

### C.2 Definition of Gaussianity

A continuous random variable *X* is said to be a Gaussian, or *normal*, random variable $X\sim N(\mu ,\sigma ^2)$ if its probability density function is given by

C.1 $$\begin{aligned} p_X(x) = \frac{1}{\sqrt{2\pi } \sigma } e^{-\frac{1}{2}\frac{(x-\mu )^2}{\sigma ^2}} . \end{aligned}$$

The multivariate generalization to a collection of continuous random variables ${\mathbf {X}}\equiv \{X_1,X_2,\ldots ,X_N\}$ is given in terms of a Gaussian probability density function with covariance matrix *C*:

C.2 $$\begin{aligned} p_{\mathbf {X}}(x_1,x_2,\ldots ,x_N) = \frac{1}{\sqrt{\mathrm{det}(2\pi C)} }e^{-\frac{1}{2} \sum _{i,j} (x_i -\mu _i)\left( C^{-1}\right) _{ij} (x_j-\mu _j)} . \end{aligned}$$

See Appendix B for additional statistical properties of Gaussian random variables.

### C.3 Tests for stationarity

There exists a vast literature on tests of non-stationarity of time-series data. The simplest tests for non-stationarity are qualitative in nature and involve looking at plots of the mean, variance, and auto-correlation as a function of time (for example, by using a sliding window of some duration to select the samples used to compute these quantities). The difficulty with this approach is deciding on what constitutes acceptable levels of variation. The concept of time-varying correlations and time-varying spectral densities are well defined and useful concepts for *locally-stationary* processes (Dahlhaus 2011), but less so for other forms of non-stationarity (Sect. 9.2.1).

It is unclear whether many of the more powerful quantitative tests for non-stationarity are useful for gravitational-wave data analysis. For example, commonly used tests, such as the augmented Dickey-Fuller test and the Phillips-Perron test, which test to see if the data follow a “unit root” auto-regressive process, do not appear to be particularly applicable since the noise encountered in gravitational-wave experiments usually exhibits high auto-correlation, and thus has roots that are naturally close to unity, which poses a challenge for these tests (Müller 2005).

The most useful tests, at least for evenly-sampled gravitational-wave data, are those based on evolutionary spectral estimates, or correlations in the Fourier coefficients. The Priestley-Subba Rao test (Priestley and Rao 1969), and modern variants based on wavelets (Nason et al. 2000), use window functions to compute spectral estimates as a function of time. A statistical test is then used to assess if the spectral estimates are consistent with stationarity. The second type of test is based on the fact that second-order stationary time series produce uncorrelated Fourier series (which is why most gravitational-wave analyses are performed in the Fourier domain). Statistical tests can be used to decide whether the level of correlation between Fourier coefficients indicates that the data are non-stationary (Dwivedi and Subba Rao 2009).

### C.4 Tests for Gaussianity

There are a large number of tests for Gaussianity described in the literature that are in regular use. These tests are based on different properties of the Gaussian distribution, and the power of the tests differ depending on the nature of the non-Gaussianity.

Three of the most widely used frequentist tests are the Shapiro–Wilk test, the Anderson–Darling test, and the Lilliefors test (a modified Kolmogorov–Smirnov test). The Shapiro–Wilk test is a regression test that out-performs other tests on small data sets, but is challenging to apply to the large data sets encountered in gravitational-wave data analysis. Both the Anderson–Darling and the Lilliefors test are based on the distance between the hypothesized cumulative distribution function (in this case, that of a Gaussian distribution) and the cumulative distribution function of the data. The Anderson–Darling test performs almost as well, and sometimes better, than the Shapiro–Wilk test (Seier 2011), and can be used on large data sets.

Bayesian tests for Gaussianity can be performed by computing the Bayes factors between competing models for the data, in this case the Gaussian distribution and some more general alternative such as Student’s *t*-distribution (Spiegelhalter 1980; Kruschke 2013). This approach has been applied to gravitational-wave data analysis (Littenberg and Cornish 2010; Cornish and Romano 2015).

## D Discretely-sampled data

In this appendix, we describe the relationship between continuous functions of time and frequency (used throughout most of the article) and their discretely-sampled counterparts. This is needed to cast a theoretical analysis into one that can be run on a digital computer, which naturally works with a finite number of discrete samples. Although we will focus attention on topics that are most-relevant to searches for stochastic gravitational-wave backgrounds, much of what we say here is general and relevant to many other signal processing applications. We refer interested readers to e.g., Oppenheim and Schafer (1999), Press et al. (1992) and Gregory (2005) for more thorough discussions of these topics.

### D.1 Discretely-sampled time-series

In the majority of the text, we represented the output of a detector by a time-series, e.g., *x*(*t*), which was a function of a *continuous* time parameter *t*. Usually, the range of *t* was infinite (from $-\infty$ to $\infty$), although sometimes we would restrict attention to a finite duration, $t\in [t_0,t_0+T]$, where $t_0$ was some initial time (usually $t_0=0$), and *T* was the length of an analysis segment or the total duration of an observation. The Fourier transform of *x*(*t*) (assumed to be defined for all *t*) was defined as

D.1 $$\begin{aligned} \tilde{x}(f)\equiv \int _{-\infty }^\infty dt\> x(t)\,e^{-i 2\pi f t}, \end{aligned}$$

with inverse Fourier transform

D.2 $$\begin{aligned} x(t)=\int _{-\infty }^\infty df\> \tilde{x}(f)\,e^{i 2\pi f t}. \end{aligned}$$

The signal power in the frequency band *f* to $f+df$ is proportional to $|\tilde{x}(f)|^2\,df$.

In practice, any real time-series will be *discretely-sampled*. This means that a continuous function of time *x*(*t*) will be represented by a set of discrete values

D.3 $$\begin{aligned} x_k\equiv x(t_k), \end{aligned}$$

where

D.4 $$\begin{aligned} t_k=t_0+k\Delta t, \quad k=0,\pm 1,\pm 2,\ldots . \end{aligned}$$

For now we allow *k* to take on an infinite set of values, corresponding to time-series having an infinite duration; shortly, we will restrict attention to a discretely-sampled time-series having a *finite* duration. Here we have assumed regularly-sampled data (i.e., the time interval between adjacent samples $x_k$ and $x_{k+1}$ is a constant $\Delta t$), although for some cases (e.g., pulsar timing) the data samples $x_k$ will correspond to irregularly-spaced times. Although it is more difficult to compute power spectra for irregularly-spaced time series, there do exist algorithms—like the Lomb–Scargle algorithm (Lomb 1976; Scargle 1982)—which can be used for this purpose. Also, to simplify the analysis slightly in what follows, we will set $t_0=0$.

A convenient way of representing a discretely-sampled time-series is to multiply the continuous function *x*(*t*) by an infinite sum of Dirac delta functions, called the *Dirac comb*:

D.5 $$\begin{aligned} \Delta _{\Delta t}(t)\equiv \sum _{k=-\infty }^\infty \delta (t-k\Delta t). \end{aligned}$$

The function

D.6 $$\begin{aligned} x_d(t) \equiv \Delta t\,\sum _{k=-\infty }^\infty x_k\,\delta (t-k\Delta t) \end{aligned}$$

is a continuous time-series representation of the discretely-sampled data $x_k=x(k\Delta t)$. (The multiplicative factor $\Delta t$ is included so that $x_d(t)$ has the same dimensions as *x*(*t*)). Using the above expression, it immediately follows that the Fourier transform of $x_d(t)$ is

D.7 $$\begin{aligned} \tilde{x}_d(f) =\Delta t\sum _{k=-\infty }^\infty x_k\, e^{-i2\pi fk\Delta t}. \end{aligned}$$

Alternatively, since $x_d(t)$ is a product of two functions in the time domain, its Fourier transform is the *convolution* of the Fourier transforms $\tilde{x}(f)$ and $\widetilde{\Delta }_{\Delta t}(f)$ in the frequency domain:

D.8 $$\begin{aligned} \tilde{x}_d(f)=\Delta t\int _{-\infty }^\infty df'\> \tilde{x}(f-f')\widetilde{\Delta }_{\Delta t}(f'). \end{aligned}$$

But since the Fourier transform of the Dirac comb is another Dirac comb,

D.9 $$\begin{aligned} \widetilde{\Delta }_{\Delta t}(f')=\frac{1}{\Delta t} \sum _{k=-\infty }^\infty \delta \left( f'-k/\Delta t\right) , \end{aligned}$$

it follows that

D.10 $$\begin{aligned} \tilde{x}_d(f) =\sum _{k=-\infty }^\infty \tilde{x}(f-k/\Delta t). \end{aligned}$$

This is the relation between the Fourier transforms of the continuous and discretely-sampled time-series. Note that $\tilde{x}_d(f)$ is periodic in *f* with period $1/\Delta t$.

One can interpret (D.10) as follows: If *x*(*t*) and $\Delta t$ are such that $\tilde{x}(f)=0$ outside $[-f_{\mathrm{N}}, f_{\mathrm{N}}]$, where $f_{\mathrm{N}}\equiv 1/(2\Delta t)$ is the *Nyquist* critical frequency, then the Fourier transform of the discretely-sampled data is *identical* to that of the original continuous time-series for $f\in [-f_{\mathrm{N}}, f_{\mathrm{N}}]$. Otherwise, there is *aliasing* of power from outside the Nyquist band making $|\tilde{x}_d(f)|>|\tilde{x}(f)|$ for $f\in [-f_{\mathrm{N}}, f_{\mathrm{N}}]$. In other words, $\tilde{x}_d(f)=\tilde{x}(f)$ for $f\in [-f_{\mathrm{N}}, f_{\mathrm{N}}]$ if and only if the following two conditions hold:

(i) *x*(*t*) is *band-limited*—i.e., $\tilde{x}(f)=0$ for $|f|\ge f_\mathrm{max}$, where $f_\mathrm{max}$ is some finite frequency,
(ii) the sampling rate $1/\Delta t$ is sufficiently large that $f_\mathrm{max}<f_{\mathrm{N}}$, or, equivalently, $\Delta t < 1/(2 f_\mathrm{max})$.

For the special case where *x*(*t*) happens to be periodic, the condition for no aliasing is to sample at least twice per period.

For a band-limited signal sampled so that $f_\mathrm{max}<f_{\mathrm{N}}$, we can recover the continuous time-series *x*(*t*) from the discrete samples $x_k$. The explicit reconstruction formula is obtained by taking the inverse Fourier transform of $\tilde{x}(f)$, replacing $\tilde{x}(f)$ by $\tilde{x}_d(f)$ for $f\in [-f_{\mathrm{N}},f_{\mathrm{N}}]$, and then using (D.7) to get an expression involving $x_k$. The final result is

D.11 $$\begin{aligned} x(t) = \sum _{k=-\infty }^\infty x_k\, \mathrm {sinc}\left[ \pi (t-k\Delta t)/\Delta t\right] , \end{aligned}$$

where $\mathrm {sinc}\,(x)\equiv \sin (x)/x$. Note that the reconstruction formula involves a sum over an *infinite* set of $x_k$. This is a consequence of *x*(*t*) being band-limited, since a function of compact support in the frequency domain must have infinite support in the time domain.

### D.2 Windowing

In addition to being discretely-sampled, real-world signals are non-zero for only a finite duration *T*. Mathematically, the simplest way to do this is to multiply an infinite-duration time-series *x*(*t*) by a *rectangular* (or *top-hat*) function

D.12 $$\begin{aligned} w(t)\equiv \bigg \{ \begin{array}{c@{\quad }c} 1 &{} 0\le t\le T\\ 0 &{} \mathrm {otherwise} \end{array}, \end{aligned}$$

which simply sets *x*(*t*) to zero outside the interval [0, *T*]. The rectangular function is a special case of a more general class of so-called *window* functions (or *tapers*), all of which set the signal to zero outside the interval [0, *T*]. Other examples include:

*Triangular window*

D.13 $$\begin{aligned} w(t)\equiv 1-|2t/T-1|, \end{aligned}$$

*Tukey window*

D.14 $$\begin{aligned} w(t)\equiv \left\{ \begin{array}{l@{\quad }l} \frac{1}{2} \left[ 1-\cos \left( \frac{2\pi }{r}\frac{t}{T}\right) \right] , &{} 0\le \frac{t}{T} \le \frac{r}{2} \\ 1, &{} \frac{r}{2} \le \frac{t}{T} \le 1 - \frac{r}{2} \\ \frac{1}{2} \left[ 1+\cos \left( \frac{2\pi }{r}\left[ \frac{t}{T}-\left( 1-\frac{r}{2}\right) \right] \right) \right] , &{} 1-\frac{r}{2} \le \frac{t}{T} \le 1 \\ \end{array} \right. , \end{aligned}$$

*Hann window*

D.15 $$\begin{aligned} w(t)\equiv \frac{1}{2}(1-\cos (2\pi t/T)). \end{aligned}$$

All of these windows taper the signal so that it “ramps-up” and “ramps-down” at the start and end of the interval. (See Fig. 88, panel a). For example, the Tukey window (D.14) is defined by a parameter *r*, which specifies the fraction of time that the window ramps-up to unity and then back down to zero, with a cosine-like taper. For $r=1$, the Tukey window becomes a Hann window (D.15). Several other common window functions are also used in signal processing applications; see e.g., Oppenheim and Schafer (1999) and Press et al. (1992) for more details.

Given a time-series *x*(*t*) and a choice of window function *w*(*t*), we define the *windowed time-series* by

D.16 $$\begin{aligned} x_w(t)\equiv w(t)x(t). \end{aligned}$$

Since $x_w(t)$ is just a product of two functions in the time domain, its Fourier transform $\tilde{x}(f)$ is the convolution of the Fourier transforms $\tilde{x}(f)$ and $\tilde{w}(f)$ in the frequency domain:

D.17 $$\begin{aligned} \tilde{x}_w(f)=\int _{-\infty }^\infty df'\> \tilde{x}(f-f')\tilde{w}(f'). \end{aligned}$$

Since *w*(*t*) has compact support in the time domain, $\tilde{w}(f)$ has infinite support in the frequency domain, meaning that the power in the windowed time-series $\tilde{x}_w(f)$ will contain power in $\tilde{x}(f')$ from frequencies $f'~(\ne f)$ as well. This *smearing* or *leakage* of power exists for *any* type of window, although the extent of the leakage depends on the shape of the window as shown in Fig. 88, panel (b).^27^ The rectangular window has the largest spectral leakage of all the windows, while the Hann window has the smallest leakage (several orders of magnitude suppression) for large frequency offsets, due to its smooth turn-on and turn-off. In general, there is a trade-off between spectral leakage and the loss of time-domain data due to the windowing. The Tukey window provides a nice balance in that spectral leakage can been strongly suppressed while only affecting a small fraction of the time domain samples. If one needs greater suppression but cannot afford to lose more data, one can use Hann windows that overlap by 50% (see, e.g., Lazzarini and Romano, 2004 in the context of stochastic background searches using LIGO data).

Windowing can also be applied to a discretely-sampled time-series, leading to a time-series which is represented by a *finite* number of discrete samples $x_k$, where $k=0,1,\ldots , N-1$ and $T=N\Delta t$. Similar to what we saw in the previous subsection, this finite, discretely-sampled time-series can be conveniently represented by a continuous time-series by multiplying by the Dirac comb. Explicitly,

D.18 $$\begin{aligned} x_{dw}(t) =\Delta t\sum _{k=0}^{N-1}w_k x_k \,\delta (t-k\Delta t), \end{aligned}$$

where $x_k=x(k\Delta t)$ and $w_k=w(k\Delta t)$. Note that this function can also be written as

D.19 $$\begin{aligned} x_{dw}(t) = w(t)x_d(t) = w_d(t)x(t), \end{aligned}$$

from which it immediately follows that

D.20 $$\begin{aligned} \tilde{x}_{dw}(f) =\int _{-\infty }^\infty df'\> \tilde{x}_d(f-f')\tilde{w}(f') =\int _{-\infty }^\infty df'\> \tilde{x}(f-f')\tilde{w}_d(f') . \end{aligned}$$

As a specific example, let *w*(*t*) be the rectangular window defined by (D.12). Then it is easy to show that

D.21 $$\begin{aligned} \tilde{w}(f) = e^{-i\pi fT}\,T\mathrm {sinc}(\pi fT). \end{aligned}$$

In addition, one can show that the discretized-version of the rectangular window has Fourier transform^28^

D.22 $$\begin{aligned} \tilde{w}_d(f) =\Delta t\sum _{k=0}^{N-1} e^{-i2\pi f k\Delta t} =e^{-i\pi (N-1)f\Delta t}\,T\mathcal {D}_N(f\Delta t), \end{aligned}$$

where

D.23 $$\begin{aligned} \mathcal {D}_N(x) \equiv \frac{1}{N}\frac{\sin (N\pi x)}{\sin (\pi x)} =\sum _{k=-\infty }^\infty \mathrm {sinc}[\pi (x-k)N] \end{aligned}$$

is the *Dirichlet kernel* (Percival and Walden 1993). Hence, for a rectangular window, (D.20) has the explicit form

D.24 $$\begin{aligned} \tilde{x}_{dw}(f)=\int _{-\infty }^\infty df'\ \tilde{x}(f-f') e^{-i\pi (N-1) f'\Delta t}\, T\mathcal {D}_N(f'\Delta t), \end{aligned}$$

which relates the Fourier transform of the infinite-duration, continuous time-series *x*(*t*) to the Fourier transform of the finite-duration, discretely-sampled time-series $x_{dw}(t)$.

### D.3 Discrete Fourier transform

Just as any real-world signal processing algorithm must deal with finite-duration, discretely-sampled time-series data $x_k$, where $k=0,1,\ldots , N-1$, so too must frequency-series (like $\tilde{x}_{dw}(f)$) be represented by a finite set of discrete values. From our earlier discussion (Sect. D.1) about aliasing, we know that the Nyquist frequency, $f_{\mathrm{N}}=1/(2\Delta t)$, is the maximum frequency of a band-limited signal that can be faithfully represented with discrete samples $x_k$ taken with sampling period $\Delta t$. In addition, the frequency resolution $\Delta f$ of the Fourier transform of a finite-duration signal is limited to $\Delta f\equiv 1/T$, where *T* is the total duration of the signal, since it is meaningless to talk about the Fourier components corresponding to periods greater than the total observation time. Thus, the best we can do in practice is to evaluate the Fourier transform of the finite set of discretely-sampled time-series data $x_k$ at the discrete frequencies

D.25 $$\begin{aligned} f_j \equiv j\Delta f =\frac{j}{N\Delta t}, \quad j=-N/2,-N/2+1,\ldots , N/2-1, \end{aligned}$$

which lie in the frequency band $[-f_{\mathrm{N}},f_{\mathrm{N}} - \Delta f]$. If *N* is odd, the index *j* runs from $-(N-1)/2$ to $(N-1)/2$. (In what follows, we will assume that *N* is even).

The *discrete Fourier transform* (DFT) of $x_k$, where $k=0,1,\ldots ,N-1$, is defined to be

D.26 $$\begin{aligned} \mathrm {DFT}(x_j) \equiv \sum _{k=0}^{N-1}x_k\,e^{-i2\pi jk/N}, \quad j=-N/2,-N/2+1,\ldots , N/2-1. \end{aligned}$$

Note that the kernel of this transformation

D.27 $$\begin{aligned} U_{jk} \equiv \frac{1}{\sqrt{N}}e^{-i2\pi jk/N}, \end{aligned}$$

is a *unitary* matrix, and thus satisfies

D.28 $$\begin{aligned} U^{-1} = U^\dagger , \quad |\mathrm{det}(U)| =1, \end{aligned}$$

as a consequence of the identity

D.29 $$\begin{aligned} \frac{1}{N}\sum _{l=0}^{N-1} e^{-i 2\pi (j-k)l/N}=\delta _{jk}. \end{aligned}$$

The inverse transformation (from the $\mathrm {DFT}(x_j)$ back to $x_k$) is thus

D.30 $$\begin{aligned} x_k =\frac{1}{N}\sum _{j=-N/2}^{N/2-1} \mathrm {DFT}(x_j)\, e^{2\pi jk/N}, \quad k=0,1,\ldots ,N-1. \end{aligned}$$

Using the above results, one can also show that

D.31 $$\begin{aligned} \sum _{k=0}^{N-1} |x_k|^2 = \frac{1}{N}\sum _{j=-N/2}^{N/2-1}|\mathrm{DFT}(x_j)|^2, \end{aligned}$$

which is called *Parseval’s theorem*. Parseval’s theorem is what tells us that the total power in a signal is the same when calculated in either the time domain or the frequency domain. (More on this below).

### D.4 DFTs and discretely-sampled Fourier transforms

To make the connection between the DFT of a set of discrete samples $x_k = x(k\Delta t)$ and the Fourier transform $\tilde{x}(f)$ of the underlying continuous time-series *x*(*t*), we first define

D.32 $$\begin{aligned} \tilde{x}_j \equiv \Delta t\, \mathrm {DFT}(x_j). \end{aligned}$$

The factor of $\Delta t$ gives $\tilde{x}_j$ and $\tilde{x}(f)$ the same units. Using (D.7), one can show that

D.33 $$\begin{aligned} \tilde{x}_j=\tilde{x}_{dw}(f_j), \end{aligned}$$

where the window function *w*(*t*) entering the definition of $\tilde{x}_{dw}(f)$ is the (trivial) rectangular window on [0, *T*]. Thus, up to a factor of $\Delta t$, the DFT of a finite set of discretely-sampled data is just the Fourier transform of the discretized, rectangular-windowed data evaluated at the discrete frequencies $f_j$.

An explicit relation between $\tilde{x}_j$ and the Fourier transform $\tilde{x}(f)$ of the infinite-duration, continuous time-series *x*(*t*) is more complicated than (D.33), due to the leakage of power from $\tilde{x}(f)$ into $\tilde{x}_{dw}(f)$, as discussed in the previous section. From (D.24) it follows that

D.34 $$\begin{aligned} \tilde{x}_j=\int _{-\infty }^\infty df'\ \tilde{x}(f_j-f') e^{-i\pi (N-1) f'\Delta t}\, T\mathcal {D}_N(f'\Delta t). \end{aligned}$$

But since $T\mathcal {D}_N(f'\Delta t)$ is typically well-approximated by the Dirac delta function $\delta (f')$, we have the *approximate* relation

D.35 $$\begin{aligned} \tilde{x}_j\simeq \tilde{x}(f_j). \end{aligned}$$

Finally, note that if the $x_k$ are real, as they will be if they are discrete samples of a real-valued time-series *x*(*t*), then

D.36 $$\begin{aligned} \tilde{x}_{-j} = \tilde{x}_j^*, \quad \mathrm{for}\quad j=0,1,\ldots ,N/2-1. \end{aligned}$$

So no information is lost if we restrict attention to non-negative frequencies

D.37 $$\begin{aligned} f_j = j\Delta f = \frac{j}{N\Delta t}, \quad \mathrm{\ where}\quad j=0,1,\ldots , N/2-1. \end{aligned}$$

### D.5 Discrete power spectra

Suppose we are given *N* samples $n_k$, $k=0,1,\ldots , N-1$, of a real-valued, stationary random process, e.g., detector noise or a stochastic signal. Then we define its discrete power spectrum as

D.38 $$\begin{aligned} {S_n}_j\equiv \frac{2}{T}|\tilde{n}_j|^2, \quad j=0,1,\ldots , N/2-1, \end{aligned}$$

where $\tilde{n}_j\equiv \Delta t\,\mathrm {DFT}(n_j)$. The factor of 2 has been included to make it a *one-sided* power spectrum, for which Parseval’s theorem (D.31) takes the form:

D.39 $$\begin{aligned} \sum _{j=0}^{N/2-1} \Delta f\, {S_n}_j =\frac{1}{N}\sum _{k=0}^{N-1}\, |n_k|^2. \end{aligned}$$

Using the approximate relation (D.35), it follows that ${S_n}_j\simeq {S_n}(f_j)$, where $S_n(f)$ is the power spectrum of the underlying continuous time series *n*(*t*). With this correspondence, we see that (D.39) is the discretized version of

D.40 $$\begin{aligned} \int _0^{f_{\mathrm{N}}}df\> S_n(f) = \frac{1}{T}\int _{0}^{T} dt\> |n(t)|^2, \end{aligned}$$

which is the continuous version of Parseval’s theorem. Similarly, the expectation values

D.41 $$\begin{aligned} \langle \tilde{n}(f)\tilde{n}^*(f')\rangle =\frac{1}{2}\delta (f-f')\,S_n(f) \end{aligned}$$

for the continuous functions become

D.42 $$\begin{aligned} \langle \tilde{n}_j \tilde{n}_{j'}^*\rangle \simeq \frac{T}{2}\delta _{jj'}{S_n}_{j'}, \end{aligned}$$

where we used

D.43 $$\begin{aligned} \delta (f_j-f_{j'}) = \delta ((j-j')\Delta f) = \frac{1}{\Delta f}\delta _{jj'} =T\delta _{jj'}. \end{aligned}$$

### D.6 Discrete and continuous probability distributions

Suppose further that the *N* samples $n_k$, $k=0,1,\ldots ,N-1$, are Gaussian distributed with zero mean and covariance matrix

D.44 $$\begin{aligned} (C_n)_{kk'} \equiv \langle n_k n_{k'}\rangle . \end{aligned}$$

Then the probability distribution for $n\equiv (n_0,n_1,\ldots , n_{N-1})^T$ is

D.45 $$\begin{aligned} p(n) = \frac{1}{\sqrt{\det (2\pi C_n)}} \exp \left[ -\frac{1}{2} n^\dagger C_n^{-1} n\right] , \end{aligned}$$

with volume element

D.46 $$\begin{aligned} d^Nn = \prod _{k=0}^{N-1} dn_k. \end{aligned}$$

Using the above results, one can show that in the limit of large *N*, the DFT (approximately) diagonalizes the covariance matrix $C_n$:

D.47 $$\begin{aligned} U C_n U^{-1} \simeq \frac{1}{2\Delta t}\mathrm{diag}({S_n}_{k}). \end{aligned}$$

From this, one can then show that the probability distribution for the discrete frequency components $\tilde{n}\equiv \left( \tilde{n}_0, \tilde{n}_1,\ldots , \tilde{n}_{N/2-1}\right) ^T$ is given by

D.48 $$\begin{aligned} p(\tilde{n}) \simeq \prod _{j=0}^{N/2-1} \frac{2}{\pi T {S_n}_{j}} \exp \left[ -\frac{2|\tilde{n}_j|^2}{T {S_n}_{j}}\right] = \prod _{j=0}^{N/2-1} \frac{2}{\pi T {S_n}_{j}} \exp \left[ -\frac{1}{2}\frac{(\mathfrak {R}\tilde{n}_j)^2 + (\mathfrak {I}\tilde{n}_j)^2}{T {S_n}_{j}/4}\right] , \end{aligned}$$

with volume element

D.49 $$\begin{aligned} d^{N/2} \tilde{n} \equiv \prod _{j=0}^{N/2-1} d(\mathfrak {R}{\tilde{n}}_j)d(\mathfrak {I}{\tilde{n}}_j). \end{aligned}$$

In the continuum limit:

D.50 $$\begin{aligned} -\frac{1}{2} n^\dagger C_n^{-1} n \simeq - \sum _{j=0}^{N/2-1} \frac{2|\tilde{n}_j|^2}{T {S_n}_{j}} \simeq -\frac{1}{2}(\tilde{n}|\tilde{n}), \end{aligned}$$

where

D.51 $$\begin{aligned} (\tilde{g}|\tilde{k}) \equiv 2\int _0^\infty df\> (S_n(f))^{-1}\left[ \tilde{g}^*(f)\tilde{k}(f)+ \tilde{g}(f)\tilde{k}^*(f)\right] \end{aligned}$$

is the noise-weighted inner product of $\tilde{g}(f)$, $\tilde{k}(f)$. See Cutler (1998) for more details regarding the noise-weighted inner product in the continuum limit.

## E Ordinary (scalar) and spin-weighted spherical harmonics

This appendix, adapted from Gair et al. (2015), summarizes some useful relations involving spin-weighted and ordinary spherical harmonics, ${}_sY_{lm}(\hat{n})$ and $Y_{lm}(\hat{n})$. For more details, see e.g., Goldberg et al. (1967) and del Castillo (2003). Note that for our analyses, we can restrict attention to spin-weighted spherical harmonics having *integral* spin weight *s*, even though spin-weighted spherical harmonics with half-integral spin weight do exist.

Ordinary spherical harmonics:

E.1 $$\begin{aligned} Y_{lm}(\hat{n})\equiv Y_{lm}(\theta ,\phi ) = N_l^m P_l^m(\cos \theta )e^{im\phi }, \quad \mathrm{where}\ N_l^m = \sqrt{\frac{2l+1}{4\pi }\frac{(l-m)!}{(l+m)!}}. \end{aligned}$$

Relation of spin-weighted spherical harmonics to ordinary spherical harmonics:

E.2 $$\begin{aligned} \begin{aligned} {}_sY_{lm}(\theta ,\phi )&=\sqrt{\frac{(l-s)!}{(l+s)!}}\, \check{\partial }^s Y_{lm}(\theta ,\phi ) \quad \mathrm{for}\quad 0\le s\le l, \\ {}_sY_{lm}(\theta ,\phi )&=\sqrt{\frac{(l+s)!}{(l-s)!}}\, (-1)^s \overline{\check{\partial }}{}^{-s} Y_{lm}(\theta ,\phi ) \quad \mathrm{for}\quad -l\le s\le 0, \end{aligned} \end{aligned}$$

where

E.3 $$\begin{aligned} \begin{aligned} \check{\partial }\eta&=-(\sin \theta )^s \left[ \frac{\partial }{\partial \theta } +i\csc \theta \frac{\partial }{\partial \phi }\right] (\sin \theta )^{-s}\eta , \\ \overline{\check{\partial }} \eta&=-(\sin \theta )^{-s} \left[ \frac{\partial }{\partial \theta } -i\csc \theta \frac{\partial }{\partial \phi }\right] (\sin \theta )^{s}\eta , \end{aligned} \end{aligned}$$

and $\eta =\eta (\theta ,\phi )$ is a spin-*s* scalar field.

Complex conjugate:

E.4 $$\begin{aligned} {}_sY_{lm}^*(\theta ,\phi ) = (-1)^{m+s}\,{}_{-s}Y_{l,-m}(\theta , \phi ). \end{aligned}$$

Relation to Wigner rotation matrices:

E.5 $$\begin{aligned} D^l{}_{m'm}(\phi ,\theta ,\psi )= (-1)^{m'} \sqrt{\frac{4\pi }{2l+1}}\,{}_mY_{l,-m'}(\theta ,\phi )e^{-im\psi }, \end{aligned}$$

or

E.6 $$\begin{aligned} \left[ D^l{}_{m'm}(\phi ,\theta ,\psi )\right] ^*= (-1)^{m} \sqrt{\frac{4\pi }{2l+1}}\,{}_{-m}Y_{l,m'}(\theta ,\phi )e^{im\psi }. \end{aligned}$$

Parity transformation:

E.7 $$\begin{aligned} {}_sY_{lm}(\pi -\theta ,\phi +\pi ) = (-1)^l\,{}_{-s}Y_{lm}(\theta , \phi ). \end{aligned}$$

Orthonormality (for fixed *s*):

E.8 $$\begin{aligned} \int d^2\Omega _{\hat{n}}\> {}_sY_{lm}(\hat{n}) \,{}_{s}Y_{l'm'}^*(\hat{n}) \equiv \int _0^{2\pi } d\phi \int _0^\pi \sin \theta \, d\theta \> {}_sY_{lm}(\theta ,\phi ) \,{}_sY_{l'm'}^*(\theta ,\phi ) = \delta _{ll'}\delta _{mm'}. \end{aligned}$$

Addition theorem for spin-weighted spherical harmonics:

E.9 $$\begin{aligned} \sum _{m=-l}^l {}_sY_{lm}(\theta _1,\phi _1)\,{}_{s'}Y_{lm}^{*}(\theta _2,\phi _2) =(-1)^{-s'}\sqrt{\frac{2l+1}{4\pi }}\,{}_{-s'}Y_{ls}(\theta _3,\phi _3) e^{is'\chi _3}, \end{aligned}$$

where

E.10 $$\begin{aligned} \cos \theta _3 = \cos \theta _1\cos \theta _2 + \sin \theta _1\sin \theta _2\cos (\phi _2-\phi _1), \end{aligned}$$

and

E.11 $$\begin{aligned} \begin{aligned} e^{-i(\phi _3+\chi _3)/2}&=\frac{\cos \frac{1}{2}(\phi _2-\phi _1)\cos \frac{1}{2}(\theta _2-\theta _1) -i\sin \frac{1}{2}(\phi _2-\phi _1)\cos \frac{1}{2}(\theta _1+\theta _2)}{\sqrt{\cos ^2\frac{1}{2}(\phi _2-\phi _1)\cos ^2\frac{1}{2}(\theta _2-\theta _1) +\sin ^2\frac{1}{2}(\phi _2-\phi _1)\cos ^2\frac{1}{2}(\theta _1+\theta _2)}}, \\ e^{i(\phi _3-\chi _3)/2}&=\frac{\cos \frac{1}{2}(\phi _2-\phi _1)\sin \frac{1}{2}(\theta _2-\theta _1) +i\sin \frac{1}{2}(\phi _2-\phi _1)\sin \frac{1}{2}(\theta _1+\theta _2)}{\sqrt{\cos ^2\frac{1}{2}(\phi _2-\phi _1)\sin ^2\frac{1}{2}(\theta _2-\theta _1) +\sin ^2\frac{1}{2}(\phi _2-\phi _1)\sin ^2\frac{1}{2}(\theta _1+\theta _2)}}. \end{aligned} \end{aligned}$$

Addition theorem for ordinary spherical harmonics:

E.12 $$\begin{aligned} \sum _{m=-l}^l Y_{lm}(\hat{n}_1) Y_{lm}^*(\hat{n}_2) =\frac{2l+1}{4\pi }\,P_l(\hat{n}_1\cdot \hat{n}_2). \end{aligned}$$

Integral of a product of spin-weighted spherical harmonics:

E.13 $$\begin{aligned}&\int d^2\Omega _{\hat{n}}\>\> {}_{s_1}Y_{l_1m_1}(\hat{n}) \, {}_{s_2}Y_{l_2m_3}(\hat{n}) \, {}_{s_3}Y_{l_3m_3}(\hat{n}) \, \nonumber \\&\quad = \sqrt{\frac{(2l_1+1)(2l_2+1)(2l_3+1)}{4\pi }} \left( \begin{array}{ccc}l_1&{}l_2&{}l_3\\ m_1&{}m_2&{}m_3 \end{array} \right) \left( \begin{array}{ccc}l_1&{}l_2&{}l_3\\ -s_1&{}-s_2&{}-s_3\end{array} \right) , \end{aligned}$$

where $$\left( \begin{array}{ccc}l_1&{}l_2&{}l_3\\ m_1&{}m_2&{}m_3 \end{array} \right) $$ is a Wigner 3-*j* symbol (Wigner 1959; Messiah 1962).

## F Gradient and curl rank-1 (vector) spherical harmonics

The gradient and curl rank-1 (vector) spherical harmonics are defined for $l\ge 1$ by:

F.1 $$\begin{aligned} \begin{aligned} Y^{G}_{(lm)a}&\equiv \frac{1}{2} {}^{(1)}\!N_l\partial _a Y_{lm} =\frac{1}{2} {}^{(1)}\!N_l\left( \frac{\partial Y_{lm}}{\partial \theta }\,\hat{\theta }_a + \frac{1}{\sin \theta }\frac{\partial Y_{lm}}{\partial \phi }\,\hat{\phi }_a\right) , \\ Y^{C}_{(lm)a}&\equiv \frac{1}{2} {}^{(1)}\!N_l(\partial _b Y_{lm})\epsilon ^b{}_a =\frac{1}{2} {}^{(1)}\!N_l\left( -\frac{1}{\sin \theta }\frac{\partial Y_{lm}}{\partial \phi }\,\hat{\theta }_a +\frac{\partial Y_{lm}}{\partial \theta }\,\hat{\phi }_a\right) , \end{aligned} \end{aligned}$$

where $\hat{\theta }$ and $\hat{\phi }$ are the standard unit vectors tangent to the 2-sphere

F.2 $$\begin{aligned} \begin{aligned} \hat{\theta }&=\cos \theta \cos \phi \,\hat{x}+ \cos \theta \sin \phi \,\hat{y}- \sin \theta \,\hat{z}, \\ \hat{\phi }&=-\sin \phi \,\hat{x}+ \cos \phi \,\hat{y}, \end{aligned} \end{aligned}$$

${}^{(1)}\!N_l$ is a normalization constant

F.3 $$\begin{aligned} {}^{(1)}\!N_l = \sqrt{\frac{2(l-1)!}{(l+1)!}}, \end{aligned}$$

and $\epsilon _{ab}$ is the Levi-Civita anti-symmetric tensor

F.4 $$\begin{aligned} \epsilon _{ab} = \sqrt{g} \left( \begin{array}{c@{\quad }c}0&{}1\\ -1&{}0\end{array}\right) , \quad g\equiv \mathrm{det}(g_{ab}). \end{aligned}$$

Following standard practice, we use the metric tensor $g_{ab}$ on the 2-sphere and its inverse $g^{ab}$ to “lower” and “raise” tensor indices—e.g., $\epsilon ^{c}{}_b \equiv g^{ca}\epsilon _{ab}$. In standard spherical coordinates $(\theta ,\phi )$,

F.5 $$\begin{aligned} g_{ab}=\left( \begin{array}{c@{\quad }c} 1&{}0\\ 0&{}\sin ^2\theta \\ \end{array} \right) , \quad \sqrt{g}=\sin \theta . \end{aligned}$$

The gradient and curl spherical harmonics are related to the spin-weight $\pm 1$ spherical harmonics

F.6 $$\begin{aligned} {}_{\pm 1}Y_{lm}(\theta , \phi ) =\sqrt{\frac{(l-1)!}{(l+1)!}} \frac{N_l^m}{\sqrt{1-x^2}} \left( \pm (1-x^2) \frac{dP_l^m}{dx} + m P_l^m(x)\right) \mathrm{e}^{im\phi }, \end{aligned}$$

where $$x=\cos \theta $$, via

F.7 $$\begin{aligned} Y^G_{(lm)a} \pm iY^C_{(lm)a} =\pm \frac{1}{\sqrt{2}}(\hat{\theta }_a \pm i \hat{\phi }_a)\,{}_{\mp 1}Y_{lm}, \end{aligned}$$

or, equivalently,

F.8 $$\begin{aligned} \begin{aligned} Y^{G}_{(lm)a}&=\frac{1}{2\sqrt{2}}\left[ \left( {}_{-1}Y_{lm} - {}_{1}Y_{lm}\right) \hat{\theta }_a +i \left( {}_{-1}Y_{lm} + {}_{1}Y_{lm}\right) \hat{\phi }_a\right] , \\ Y^{C}_{(lm)a}&=\frac{1}{2\sqrt{2}}\left[ \left( {}_{-1}Y_{lm} - {}_{1}Y_{lm}\right) \hat{\phi }_a -i \left( {}_{-1}Y_{lm} + {}_{1}Y_{lm}\right) \hat{\theta }_a\right] . \end{aligned} \end{aligned}$$

For decompositions of vector-longitudinal backgrounds, as discussed in the main text, it will be convenient to construct rank-2 tensor fields

F.9 $$\begin{aligned} \begin{aligned} Y^{V_G}_{(lm)ab}&= Y^G_{(lm)a}\hat{n}_b+ Y^G_{(lm)b}\hat{n}_a, \\ Y^{V_C}_{(lm)ab}&= Y^C_{(lm)a}\hat{n}_b+ Y^C_{(lm)b}\hat{n}_a, \end{aligned} \end{aligned}$$

where $\hat{n}$ is the unit radial vector orthogonal to the surface of the 2-sphere:

F.10 $$\begin{aligned} \hat{n} = \sin \theta \cos \phi \,\hat{x}+\sin \theta \sin \phi \,\hat{y} +\cos \theta \,\hat{z}. \end{aligned}$$

These fields satisfy the following orthonormality relations

F.11 $$\begin{aligned} \begin{aligned} \int \! d^2\Omega _{\hat{n}}\> Y^{V_G}_{(lm)ab} (\hat{n}) Y^{V_G}_{(l'm')}{}^{ab\,*}(\hat{n})&=\delta _{ll'}\delta _{mm'}, \\ \int \! d^2\Omega _{\hat{n}}\> Y^{V_C}_{(lm)ab} (\hat{n}) Y^{V_C}_{(l'm')}{}^{ab\,*}(\hat{n})&=\delta _{ll'}\delta _{mm'}, \\ \int \! d^2\Omega _{\hat{n}}\> Y^{V_G}_{(lm)ab} (\hat{n}) Y^{V_C}_{(l'm')}{}^{ab\,*}(\hat{n})&=0. \end{aligned} \end{aligned}$$

## G Gradient and curl rank-2 (tensor) spherical harmonics

The gradient and curl rank-2 (tensor) spherical harmonics are defined for $l\ge 2$ by:

G.1 $$\begin{aligned} \begin{aligned} Y^G_{(lm)ab}&= {}^{(2)}\!N_l \left( Y_{(lm);ab} - \frac{1}{2} g_{ab} Y_{(lm);c}{}^{c} \right) , \\ Y^C_{(lm)ab}&= \frac{{}^{(2)}\!N_l}{2} \left( Y_{(lm);ac}\epsilon ^c{}_b + Y_{(lm);bc} \epsilon ^c{}_a \right) , \end{aligned} \end{aligned}$$

where a semicolon denotes covariant derivative on the 2-sphere, $\epsilon _{ab}$ is the Levi-Civita anti-symmetric tensor (F.4), $g_{ab}$ is the metric tensor on the 2-sphere (F.5), and ${}^{(2)}\!N_l$ is a normalization constant

G.2 $$\begin{aligned} {}^{(2)}\!N_l = \sqrt{\frac{2 (l-2)!}{(l+2)!}}. \end{aligned}$$

Using the standard polarization tensors on the 2-sphere:

G.3 $$\begin{aligned} \begin{aligned} e^+_{ab}(\hat{n})&= \hat{\theta }_a\hat{\theta }_b -\hat{\phi }_a\hat{\phi }_b, \\ e^\times _{ab}(\hat{n})&= \hat{\theta }_a\hat{\phi }_b +\hat{\phi }_a\hat{\theta }_b, \end{aligned} \end{aligned}$$

where $\hat{\theta }$, $\hat{\phi }$ are given by (F.2) and $\hat{n}$ by (F.10), we have (Hu and White 1997):

G.4 $$\begin{aligned} \begin{aligned} Y^G_{(lm)ab}(\hat{n})&= \frac{{}^{(2)}\!N_l}{2} \left[ W_{(lm)}(\hat{n}) e_{ab}^+(\hat{n}) + X_{(lm)}(\hat{n}) e_{ab}^\times (\hat{n}) \right] , \\ Y^C_{(lm)ab}(\hat{n})&= \frac{{}^{(2)}\!N_l}{2} \left[ W_{(lm)}(\hat{n})e_{ab}^\times (\hat{n}) - X_{(lm)}(\hat{n}) e_{ab}^+(\hat{n}) \right] , \end{aligned} \end{aligned}$$

where

G.5 $$\begin{aligned} \begin{aligned} W_{(lm)}(\hat{n})&= \left( \frac{\partial ^2}{\partial \theta ^2} - \cot \theta \frac{\partial }{\partial \theta } +\frac{m^2}{\sin ^2\theta } \right) Y_{lm}(\hat{n}) = \left( 2 \frac{\partial ^2}{\partial \theta ^2} + l(l+1) \right) Y_{lm}(\hat{n}), \\ X_{(lm)}(\hat{n})&= \frac{2 i m}{\sin \theta } \left( \frac{\partial }{\partial \theta } - \cot \theta \right) Y_{lm}(\hat{n}). \end{aligned} \end{aligned}$$

These functions enter the expression for the spin-weight $\pm 2$ spherical harmonics (Newman and Penrose 1966; Goldberg et al. 1967):

G.6 $$\begin{aligned} {}_{\pm 2}Y_{lm}(\hat{n}) =\frac{{}^{(2)}\!N_l}{\sqrt{2}} \left[ W_{(lm)}(\hat{n})\pm i X_{(lm)}(\hat{n})\right] , \end{aligned}$$

which are related to the gradient and curl spherical harmonics via

G.7 $$\begin{aligned} Y^G_{(lm)ab}(\hat{n}) \pm i Y^C_{(lm)ab}(\hat{n})&=\frac{1}{\sqrt{2}} \left( e_{ab}^+(\hat{n}) \pm i e_{ab}^\times (\hat{n})\right) \,{}_{\mp 2}Y_{lm}(\hat{n}). \end{aligned}$$

Note that the gradient and curl spherical harmonics satisfy the orthonormality relations

G.8 $$\begin{aligned} \begin{aligned} \int _{S^2} d^2\Omega _{\hat{n}}\> Y^{G}_{(lm)ab} (\hat{n}) Y^{G}_{(l'm')}{}^{ab\,*}(\hat{n})&=\delta _{ll'}\delta _{mm'}, \\ \int _{S^2} d^2\Omega _{\hat{n}}\> Y^{C}_{(lm)ab} (\hat{n}) Y^{C}_{(l'm')}{}^{ab\,*}(\hat{n})&=\delta _{ll'}\delta _{mm'}, \\ \int _{S^2} d^2\Omega _{\hat{n}}\> Y^{G}_{(lm)ab} (\hat{n}) Y^{C}_{(l'm')}{}^{ab\,*}(\hat{n})&=0. \end{aligned} \end{aligned}$$

## H Translation between $\hat{n}$ and $\hat{k}$ conventions

Numerous papers on detecting stochastic gravitational-wave backgrounds have adopted the convention where the polarization tensors and detector response functions are functions of the *direction of propagation* of the gravitational wave, $\hat{k}$, where $\hat{k}$ points radially *outward*. In this article, we have adopted instead the convention where plane wave expansions, polarization tensors, and response functions are written in terms of the *direction to the source* of the gravitational wave, $\hat{n}$, where again $\hat{n}$ points radially outward. In both approaches, the unit vectors $\hat{l}$ and $\hat{m}$, which are perpendicular to $\hat{k}$ (or $\hat{n}$) and are used to define the polarization tensors, are typically chosen to be the standard spherical polar coordinate unit vectors $\hat{\theta }$ and $\hat{\phi }$. Thus, the polarization tensors $e^{+,\times }_{ab}(\hat{n})$ and $e^{+,\times }_{ab}(\hat{k})$ are the same for both conventions. What is different is the expression for an individual plane wave—either $e^{i2\pi f(t-\hat{k}\cdot {\vec {x}}/c)}$ or $e^{i2\pi f(t+\hat{n}\cdot {\vec {x}}/c)}$—as the direction of propagation of the wave is opposite the direction to the source.

In this appendix, we summarize how the expressions for the response functions $R^{ab}(f,\hat{n})$, $R^A(f, \hat{n})$, and $R^P_{(lm)}(f)$, given in previous sections are related to similar quantities calculated in other papers that use the $\hat{k}$-convention. For completeness, we will write down expressions for the vector and scalar polarization modes (Section 8.3) in addition to the standard tensor ($+$, $\times$ or grad and curl) modes in general relativity. We will denote quantities calculated using the $\hat{k}$-convention with an overbar, e.g., $\bar{R}^A(f,\hat{k})$.

### H.1 General relationship between the response functions

Plane wave expansion:

H.1 $$\begin{aligned} h_{ab}(t,{\vec {x}}) = \int _{-\infty }^\infty \int d^2\Omega _{\hat{n}}\> h_{ab}(f,\hat{n}) e^{i2\pi f(t+\hat{n}\cdot {\vec {x}}/c)}. \end{aligned}$$

Detector response:

H.2 $$\begin{aligned} \begin{aligned} h(t)&= \int _{-\infty }^\infty d\tau \int d^3 y\> R^{ab}(\tau , \vec {y}) h_{ab}(t-\tau , {\vec {x}}-\vec {y}) \\&=\int _{-\infty }^\infty df\int d^2\Omega _{\hat{n}}\> R^{ab}(f,\hat{n}) h_{ab}(f,\hat{n})e^{i2\pi ft}, \end{aligned} \end{aligned}$$

where

H.3 $$\begin{aligned} R^{ab}(f,\hat{n}) = e^{i2\pi f\hat{n}\cdot {\vec {x}}/c} \int _{-\infty }^\infty d\tau \int d^3 y\> R^{ab}(\tau , \vec {y}) e^{-i2\pi f(\tau +\hat{n}\cdot \vec {y}/c)}. \end{aligned}$$

Note that compared to an expansion in terms of the direction of propagation $\hat{k}$, we have:

H.4 $$\begin{aligned} R^{ab}(f,\hat{n}) = \bar{R}^{ab}(f,\hat{k})\big |_{\hat{k}=-\hat{n}}. \end{aligned}$$

This is the general relationship between the response functions for the two approaches.

### H.2 Polarization basis response functions

The response functions in the polarization basis are given by:

H.5 $$\begin{aligned} R^A(f,\hat{n}) = R^{ab}(f,\hat{n}) e^A_{ab}(\hat{n}), \end{aligned}$$

where $A=\{+,\times , X, Y, B, L\}$ label the tensor, vector, and scalar polarization modes (two for each). Since the polarization basis tensors $e^A_{ab}(\hat{n})$ are the same for the two approaches, it follows from (H.4) that

H.6 $$\begin{aligned} R^A(f,\hat{n}) = \bar{R}^{ab}(f,\hat{k}) e^A_{ab}(\hat{n})\big |_{\hat{k}=-\hat{n}}. \end{aligned}$$

If we further use the transformation properties of the polarization basis tensors $e^A_{ab}(\hat{n})$ under a parity transformation (i.e., $\hat{n}\rightarrow -\hat{n}$) we have:

H.7 $$\begin{aligned} \begin{aligned} R^+(f,\hat{n})&=\bar{R}^+(f,\hat{k})\big |_{\hat{k}=-\hat{n}},\\ R^\times (f,\hat{n})&=-\bar{R}^\times (f,\hat{k})\big |_{\hat{k}=-\hat{n}},\\ R^X(f,\hat{n})&=-\bar{R}^X(f,\hat{k})\big |_{\hat{k}=-\hat{n}},\\ R^Y(f,\hat{n})&=\bar{R}^Y(f,\hat{k})\big |_{\hat{k}=-\hat{n}},\\ R^B(f,\hat{n})&=\bar{R}^B(f,\hat{k})\big |_{\hat{k}=-\hat{n}},\\ R^L(f,\hat{n})&=\bar{R}^L(f,\hat{k})\big |_{\hat{k}=-\hat{n}}.\\ \end{aligned} \end{aligned}$$

Note that in terms of standard angular coordinates $(\theta ,\phi )$ on the sphere, the substitution $\hat{k}= -\hat{n}$ corresponds to

H.8 $$\begin{aligned} \theta \rightarrow \pi -\theta , \quad \phi \rightarrow \phi +\pi , \end{aligned}$$

for which

H.9 $$\begin{aligned} \begin{aligned}&\sin \theta \rightarrow \sin \theta , \\&\cos \theta \rightarrow -\cos \theta , \\&\sin \phi \rightarrow -\sin \phi , \\&\cos \phi \rightarrow -\cos \phi . \end{aligned} \end{aligned}$$

### H.3 Spherical harmonic basis response functions

The response functions in the spherical harmonic basis are given by:

H.10 $$\begin{aligned} R^P_{(lm)}(f) = \int d^2\Omega _{\hat{n}}\> R^{ab}(f,\hat{n}) Y^P_{(lm)ab}(\hat{n}), \end{aligned}$$

where $P=\{G,C,V_G,V_C, B,L\}$ label the tensor, vector, and scalar spherical harmonic modes. If we use the transformation properties of the spherical harmonics $Y^P_{(lm)ab}(\hat{n})$ under a parity transformation, it follows that:

H.11 $$\begin{aligned} \begin{aligned} R^G_{(lm)}(f)&=(-1)^l\bar{R}^G_{(lm)}(f),\\ R^C_{(lm)}(f)&=(-1)^{l+1}\bar{R}^C_{(lm)}(f),\\ R^{V_G}_{(lm)}(f)&=(-1)^l\bar{R}^{V_G}_{(lm)}(f),\\ R^{V_C}_{(lm)}(f)&=(-1)^{l+1}\bar{R}^{V_C}_{(lm)}(f),\\ R^B_{(lm)}(f)&=(-1)^l\bar{R}^B_{(lm)}(f),\\ R^L_{(lm)}(f)&=(-1)^l\bar{R}^L_{(lm)}(f). \end{aligned} \end{aligned}$$

Thus, the curl modes (both tensor and vector) involve a factor of $(-1)^{l+1}$, while all the other modes involve a factor of $(-1)^l$.

## Footnotes

1 The coalescence rate is expected to vary significantly with redshift *z*, so this simple calculation, which assumes a constant coalescence rate, provides only a rough estimate.

2 Actually, even if the gravity-gradient and seismic noise were zero, one couldn’t go below ${\sim }1~\mathrm {Hz}$ with the current generation of ground-based laser interferometers, since the suspended mirrors (i.e., the test masses) are no longer freely floating when you go below their resonant frequencies: ${\sim }1~\mathrm {Hz}$.

3 Signals may be separable even when overlapping in time and frequency if the detector has good sky resolution, or if the signals have additional complexities due to effects such as orbital evolution and precession.

4 The gravitational-wave propagation direction, which we will denote by $\hat{k}$, is given by $\hat{k}=-\hat{n}$.

5 In some treatments, the Bayesian interpretation is equated to philosophical schools such as Berkeley’s empiricist idealism, or subjectivism, which holds that things only exist to the extent that they are perceived, while the frequentist interpretation is equated to Platonic realism, or metaphysical objectivism, holding that things exist objectively and independently of observation. These equivalences are false. A physical object can have a definite, Platonic existence, and Bayesians can still assign probabilities to its attributes since our ability to measure is limited by imperfect equipment.

6 Since the model $\bar{h}(t)$ will differ from the actual *h*(*t*), we use an overbar for the model to distinguish the two.

7 Our convention for Fourier transform is $\tilde{h}(f)= \int _{-\infty }^\infty dt \>e^{-i2\pi f t} h(t)$.

8 A flat prior for $S_h$ yields more conservative (i.e., larger) upper limits for $S_h$ than a Jeffrey’s prior, since there is more prior weight at larger values of $S_h$ for a flat prior than for a Jeffrey’s prior.

9 See Creighton et al. (2009) and Koop and Finn (2014) for an alternative derivation of the response of a detector to gravitational waves, which is done in terms of the curvature tensor and not the metric perturbations.

10 Some authors (Christensen 1990, 1992; Flanagan 1993; Allen and Romano 1999; Cornish and Larson 2001; Finn et al. 2009), including us in the past, have defined the response function $R^{ab}(f,\hat{n})$ *without* the factor of $e^{i 2\pi f\hat{n}\cdot {\vec {x}}/c}$. If one chooses coordinates so that the measurement is made at ${\vec {x}}={\vec {0}}$, then these two definitions agree. Just be aware of this possible difference when reading the literature. To distinguish the two definitions, we will use the symbol $\bar{R}^{ab}(f,\hat{n})$ to denote the expression without the exponential term, i.e., $R^{ab}(f,\hat{n}) = e^{i 2\pi f\hat{n}\cdot {\vec {x}}/c}\bar{R}^{ab}(f,\hat{n})$.

11 There is a factor of $(-1)^l$ difference between $R^G_{(lm)}(f)$ in (5.23) and (92) in Gair et al. (2014). The difference is due to the change in expressing the response functions in terms of the direction to the gravitational-wave source, $\hat{n}$, as opposed to the direction of gravitational-wave propagation, $\hat{k}=-\hat{n}$. Appendix H provides expressions relating the response functions calculated using these two different conventions.

12 Although Fig. 28 shows $\hat{u}$ and $\hat{v}$ making right angles with one another, the following calculation is valid for $\hat{u}$ and $\hat{v}$ separated by an *arbitrary* angle.

13 Recall from Footnote 10 that the phase factors $e^{i 2\pi f\hat{n}\cdot {\vec {x}}_{I,J}/c}$ are already contained in our definition of the response functions $R^A_{I,J}(f,k)$. If we explicitly display this dependence then

$$\begin{aligned} \Gamma _{IJ}(f) \equiv \frac{1}{8\pi } \int d^2\Omega _{\hat{n}} \sum _A \bar{R}_I^A(f,\hat{n})\bar{R}_J^{A}{}^*(f,\hat{n}) e^{i 2\pi f\hat{n}\cdot ({\vec {x}}_I -{\vec {x}}_J)/c}, \nonumber \end{aligned}$$

where $\bar{R}^A_{I,J}(f,\hat{n}) \equiv \bar{R}^{ab}_{I,J}(f,\hat{n})e^A_{ab}(\hat{n})$. One often sees this latter expression for $\Gamma _{IJ}(f)$ in the literature.

14 This is needed, for example, to calculate the overlap functions for an array of seismometers in the small-antenna limit (Coughlin and Harms 2014). For this case, the detector tensors are simply $D_I^{ab} \equiv u_I^a u_I^b$, where $\hat{u}_I$ is a unit vector pointing along the sensitive direction of the *I*th seismometer.

15 Actually, the bin size for a typical LIGO search for a stochastic background is *larger* than the ${\sim } 10^{-2}~\mathrm{Hz}$ Doppler shift due to the Earth’s orbital motion around the Sun.

16 In equatorial coordinates, the galactic center is located at $(\mathrm{ra},\mathrm{dec}) = (-6^\mathrm{h} 15^\mathrm{m}, -29^\circ )$.

17 For correlated measurements, $Q_i = \sum _j(\bar{C}^{-1})_{ij}/h_i$ where $\bar{C}^{-1}$ is the inverse of the re-scaled covariance matrix $\bar{C}_{ij}\equiv C_{ij}/(h_i h_j)$.

18 The last equality in (6.23) follows from (5.38) with the Dirac delta function $\delta (f-f')$ replaced by its finite-time version $\delta _T(f-f') = T\mathrm {sinc}[\pi (f-f')T]$, which equals *T* when $f=f'$.

19 The number of terms in the expansion is given by $2+\mathrm{floor}(1+l/2)$.

20 The functions here are a factor of 1 / 2 smaller than those in Fig. 8 in Gair et al. (2014), due to different definitions of $\gamma (t; f, \hat{n})$. Compare (115) in that paper to (7.8) and (7.10) above.

21 The multiplications inside the exponential are *matrix* multiplications—either summations over sky directions $\hat{n}$ or summations over discrete times and frequencies, *t* and *f*.

22 What to do when the noise is non-stationary or non-Gaussian is discussed in Sects. 9.2 and 9.3.

23 At times it will be convenient to think of *M* as an $N_f\times N_f$ block diagonal matrix with $N_b\times 2$ blocks, one for each frequency. At other times, it will be convenient to think of *M* as an $N_b\times 2$ block matrix with diagonal $N_f\times N_f$ blocks. The calculations we need to do usually determine which representation is most appropriate. (Similar statements can be made for the vectors $\hat{C}$ and $\mathcal{S}$).

24 This corresponds to the direction to the pulsar and the direction to the source of the gravitational wave being the same. For this case, the radio pulse from the pulsar and the gravitational wave travel in phase with one another from the pulsar to Earth. It is as if the radio pulse “surfs” the gravitational wave (Chamberlin and Siemens 2012).

25 For our discussion of inflation, we will work in *particle physics units* where both $c=1$ and $\hbar =1$. In place of using Newton’s gravitational constant *G*, we will use the *reduced* Planck mass $M_\mathrm{Pl} \equiv (\hbar c/8\pi G)^{1/2} = 2.435\times 10^{18}~\mathrm{GeV}/c^2$. In these units $M_\mathrm{Pl}^{2} = 1/8\pi G$, which simplifies several of the formulae. If you want to reinstate all of the relevant factors of $\hbar$ and *c*, note that the inflaton field $\phi$ has dimensions of energy and the inflaton potential $V(\phi )$ has dimensions of energy density.

26 One should think of a radiometer upper-limit map as a convenient way of representing upper limits for a *collection* of individual point-source signal models, one for each point on the sky. As described in Sect. 7.3.6, the radiometer analysis ignores correlations between neighboring pixels on the sky, completely side-stepping the deconvolution problem associated with a non-trivial point spread function for the search. In other words, each pixel of a radiometer map corresponds to a separate analysis.
